# Diverse Secondary Metabolites from the Marine-Derived Fungus *Dichotomomyces cejpii* F31-1

**DOI:** 10.3390/md15110339

**Published:** 2017-11-01

**Authors:** Yan-Xiu Chen, Meng-Yang Xu, Hou-Jin Li, Kun-Jiao Zeng, Wen-Zhe Ma, Guo-Bao Tian, Jun Xu, De-Po Yang, Wen-Jian Lan

**Affiliations:** 1 School of Pharmaceutical Sciences, Sun Yat-sen University, Guangzhou 510006, China; chenyx239@mail2.sysu.edu.cn (Y.-X.C.); xumy3@mail2.sysu.edu.cn (M.-Y.X.); junxu@biochemomes.com (J.X.); lssydp@mail.sysu.edu.cn (D.-P.Y.); 2 Guangdong Technology Research Center for Advanced Chinese Medicine, Guangzhou 510006, China; 3 School of Chemistry, Sun Yat-sen University, Guangzhou 510275, China; ceslhj@mail.sysu.edu.cn; 4 Zhongshan School of Medicine, Sun Yat-sen University, Guangzhou 510080, China; zkj3880@163.com (K.-J.Z.); tiangb@mail.sysu.edu.cn (G.-B.T.); 5 State Key Laboratory of Quality Research in Chinese Medicine, Macau Institute for Applied Research in Medicine and Health, Macau University of Science and Technology, Avenida Wai Long, Taipa 519020, Macau (SAR), China; wzma@must.edu.mo

**Keywords:** *Dichotomomyces cejpii*, diverse secondary metabolites, amino acid-directed strategy, nitrogen-containing compounds, bioactivity

## Abstract

By adding l-tryptophan and l-phenylalanine to GPY medium, twenty-eight compounds, including amides, polyketides, a sesquiterpenoid, a diterpenoid, a meroterpenoid, diketopiperazines, β-carbolines, fumiquinazolines, and indole alkaloids, were discovered from the marine-derived fungus *Dichotomomyces cejpii* F31-1, demonstrating the tremendous biosynthetic potential of this fungal strain. Among these compounds, four amides dichotomocejs A–D (**1**–**4**), one polyketide dichocetide A (**5**), and two diketopiperazines dichocerazines A–B (**15** and **16**) are new. The structures of these new compounds were determined by interpreting detailed spectroscopic data as well as calculating optical rotation values and ECD spectra. Obviously, *Dichotomomyces cejpii* can effectively use an amino acid-directed strategy to enhance the production of nitrogen-containing compounds. Dichotomocej A (**1**) displayed moderate cytotoxicity against the human rhabdomyosarcoma cell line RD with an IC_50_ value of 39.1 µM, and pityriacitrin (**22**) showed moderate cytotoxicity against the human colon carcinoma cell line HCT116 with an IC_50_ value of 35.1 µM.

## 1. Introduction

**Figure 1:**
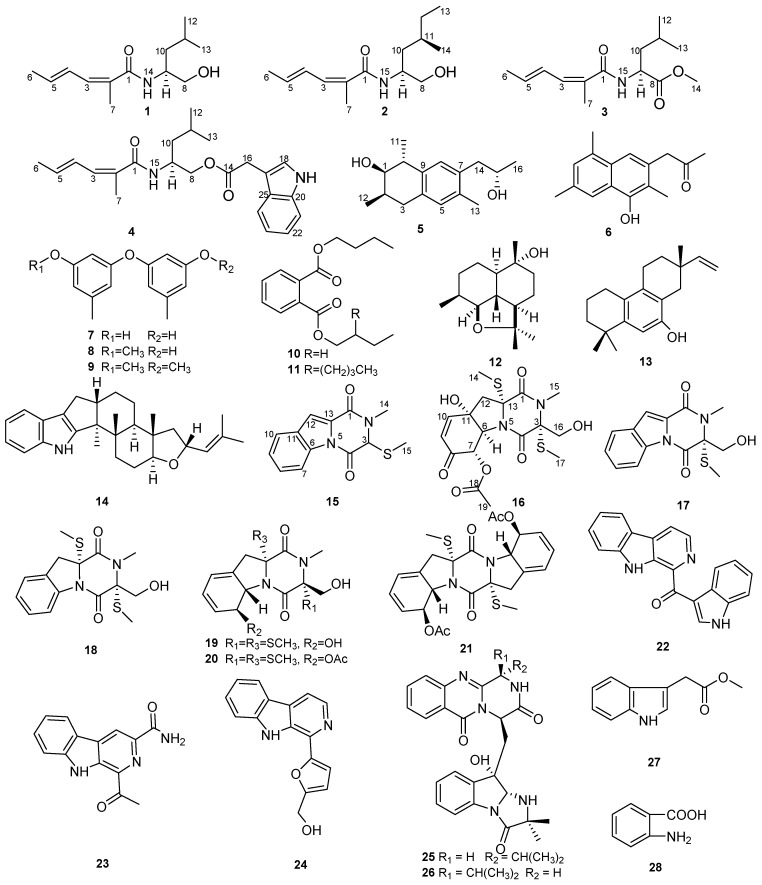
Chemical structures of compounds **1**–**28**.

The ascomycete *Dichotomomyces cejpii* is a common fungus known for its heat-resistant properties, that allow it to survive at 70 °C for 60 min [1]. *Dichotomomyces cejpii* is also representative of the fungus found in the soil under decomposing corpses, which highlights its potential as a forensic tool [2]. Extracts from this fungus display ciliostatic activity, cytotoxic activity, and broad-spectrum antimicrobial activity. In addition, *Dichotomomyces cejpii* has a substantial inhibitory effect on some drug-resistant bacterium [3,4,5]. The major metabolites of the fungus are diketopiperazines, indoloditerpenes, polyketides, and steroids. These secondary metabolites exhibited various bioactivities. For example, Henrik et al., isolated indoloditerpenes with antagonistic activities at GPR18 and cannabinoid receptors [6], one polyketide and three diketopiperazines with NF-κB inhibitory potentials [7], and one xanthocillin derivative and three steroids which can be aβ-42 lowering agents [8]. In our effort to discover chemically diverse alkaloids of fungal origin with significant bioactivities, the metabolite profile of the fungus *Dichotomomyces cejpii* F31-1 associated with the soft coral *Lobophytum crassum* collected in the South China Sea caught our attention. To encourage the fungus to generate alkaloids, we adopted the amino acid–directed strategy described previously [9,10]. By adding l-tryptophan and l-phenylalanine to GPY medium (20 g/L glucose, 5 g/L peptone, 2 g/L yeast extract, 30 g/L sea salt, and 1L H_2_O at pH 7.5), seven new compounds, including four aliphatic amides dichotomocejs A–D (**1**–**4**), one polyketide dichocetide A (**5**), and two diketopiperazines dichocerazines A–B (**15** and **16**), together with twenty-one known compounds (**6**–**14**, **17**–**28**), were isolated from the EtOAc extract of the culture broth (Figure 1). The cytotoxicities of compounds **1**, **7**, **8**, **11**, **15**, **22**, **23**, and **27** were evaluated against the four tumor cell lines HCT116, RD, ACHN, and A2780T, and the antimicrobial activities of compounds **4**, **8**, **13**, **14**, **22**, and **24** were evaluated against the four bacteria ATCC29213, ATCC25922, ATCC27853, and ATCC19606. In this paper, we report the isolation, structural determination, and bioactivities of these compounds.

## 2. Results and Discussion

### 2.1. Structural Elucidation

**Figure 2:**
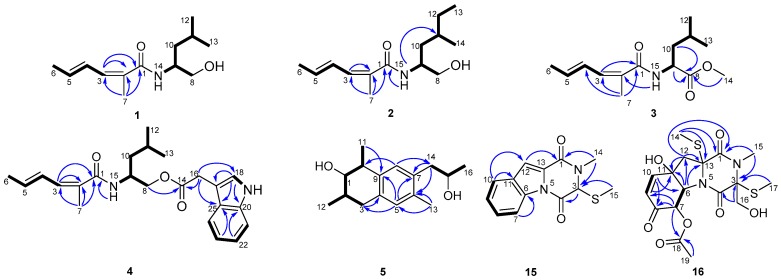
The ^1^H-^1^H COSY (bold line) and key HMBC correlations (arrows) of compounds **1**–**5** and **15**–**16**.

Dichotomocej A (**1**) was afforded as a yellowish oil. The molecular formula was deduced to be C_13_H_23_NO_2_ from the HRESIMS quasi-molecular ion [M + H]^+^ peak at *m/z* 226.1809 (calcd. for 226.1802) (Supplementary Figure S2), indicating three sites of unsaturation. The ^13^C NMR spectra (Table 1 and Supplementary Figure S4) showed thirteen carbons, including four methyls, two methylenes, two sp^3^ methines, three olefinic methines, one olefinic quaternary carbon, and one carbonyl. Therefore, the presence of two pairs of double bonds and one carbonyl accounts for the degrees of unsaturation. In addition, both the methine at *δ*_C_ 50.7 and the carbonyl at *δ*_C_ 170.4 were attached to a nitrogen atom, and the methylene at *δ*_C_ 67.1 was bonded to an oxygen atom. The ^1^H NMR data and HMQC spectra (Table 2 and Supplementary Figure S5) showed signals indicative of four methyl groups at *δ*_H_ 0.94 (d, 6.8), 0.95 (d, 6.8), 1.86 (d, 6.4), and 1.94 (s), two methylenes at *δ*_H_ 1.41 (dt, 8.4, 6.0) and 3.57 (dd, 10.8, 6.0)/3.72 (dd, 10.8, 3.2), two sp^3^ methines at *δ*_H_ 1.66 (m) and 4.12 (m), three olefinic methines at *δ*_H_ 6.04 (dq, 13.2, 6.8), 6.33 (ddq, 13.2, 11.2, 1.6), and 6.88 (d, 11.2), and one broad signal at *δ*_H_ 5.77 (brd, 6.4).The ^1^H-^1^H COSY correlations (Figure 2) of H-3 with H-4, H-4 with H-5, H-5 with H-6, and the key HMBC cross peaks (Figure 2) of H-7 with C-1/C-2/C-3 and H-3 with C-1/C-2 indicated the presence of a CH_3_CH=CH-CH=C(CH_3_)COX fragment. The ^1^H-^1^H COSY correlations of H-8 with H-9, H-9 with H-10, H-9 with H-14, H-10 with H-11, and H-11 with H-12/H-13 supported the right-hand aliphatic alcohol fragment of our proposed molecular structure. Thus, the structure of **1** was established as shown in Figure 1, which is similar to that of 2-methyl-hexa-2,4-dienoic acid, isoleucinol amide [11]. However the methylene of **1** at C-10 was not consistent with the aliphatic alcohol fragment present in the 2-methyl-hexa-2,4-dienoic acid, isoleucinol amide. Additionally in **1**, the geminal methyls at C-11 replaced a methyl and an ethyl fragment in the above mentioned analog. The double bond at C-4 of **1** was in the *E* configuration based on the large *J*_H4-H5_ coupling constant (13.2) and the NOESY correlation of H-3 with H-5. However, the double bond at C-2 was in the *Z* configuration based on the NOESY cross peaks of H_3_-7 with H-3/H-5. The absolute configuration of **1** was determined to be 9*S* based on the good match of the experimental optical rotation (−41.9) with our calculated value (−42.1) (Supplementary Table S1).

Dichotomocej B (**2**) was obtained as a pale-yellow oil. This compound had a molecular formula of C_14_H_25_NO_2_ based on the HRESIMS peak at *m/z* 240.1955 [M + H]^+^ (calcd. for 240.1958) (Supplementary Figure S9) and had the same number of degrees of unsaturation as **1**. Careful inspection of the NMR spectra (Table 1 and Table 2, Supplementary Figures S10–S16) of **2** suggested that its NMR spectra resembled those of **1**. The only difference was a methyl and an ethyl fragment at C-11 in **2** instead of the geminal methyls seen in **1**. This was confirmed by the ^1^H-^1^H COSY cross peak (Figure 2) of H-12 with H-13 in **2**, and these substituents are consistent with the molecular formula of **2**. The double bond at C-4 of **2** was in the *E* configuration inferred by the large *J*_H4-H5_ coupling constant (14.8) and the NOESY correlation of H-3 with H-5, and the double bond at C-2 was in the *Z* configuration based on the NOESY correlations between H-3 and H-15 and between H_3_-7 and H-3/H-15.

The relative stereochemistry was inferred by the NOESY data. The NOESY correlations of H_3_-7 and H_3_-13 with H-9/H-11 revealed that H-9 and H-11 were located on the same side of the molecule. A comparison of the experimental optical rotation (−4.4) of **2** with the calculated value (−7.1) (Supplementary Table S1) suggested the stereochemistry of **2** was 9*S*, 11*R* since **2** only has two possible absolute configurations with opposite optical activities.

Dichotomocej C (**3**) was isolated as a yellowish oil. The HRESIMS spectrum of compound **3** gave a molecular ion peak at *m/z* 254.1750 [M + H]^+^ (calcd. for 254.1751) (Supplementary Figure S17), which suggested a molecular formula of C_14_H_23_NO_3_ with four degrees of unsaturation. The NMR data (Table 1 and Table 2, Supplementary Figures S18–S24) of **3** were similar to those of **1**. The only significant difference was the presence of a methyl formate group at the C-8 position in **3** in place of the hydroxymethyl group seen in **1**. This finding was supported by the HMBC correlations (Figure 2) from H-9, H-10 and H-14 to C-8 and was consistent with the one additional degree of unsaturation in **3** relative to **1**. In addition, the configurations of the two double bonds in **3** were the same as those in **1**. This observation was based on the large *J*_H4-H5_ coupling constant (15.2) and the NOESY cross peaks of H-3 with H-5/H-15 and H_3_-7 with H-3/H-15. The experimental ${\left[\alpha \right]}_{D}^{25}$ value (−51.6) of **3** showed the same direction of rotation as the calculated optical rotation (−48.4) (Supplementary Table S1), thus, **3** was assigned an absolute configuration of 9*S*.

Dichotomocej D (**4**) was afforded as a yellowish oil. Compound **4** showed a molecular ion peak at *m/z* 383.2296 [M + H]^+^ (calcd for 383.2329) in the HRESIMS spectrum (Supplementary Figure S25), which led us to give a molecular formula of C_23_H_30_N_2_O_3_, corresponding to ten double bond equivalents. The comparison of the NMR data (Table 1 and Table 2, Supplementary Figures S26–S32) of **4** with those of **1** displayed that the alkyl chain of **4** was the same as that of **1**. The major difference was an indole acetoxyl in **4** replacing the hydroxyl group at C-8 in **1**, and the presence of that fragment accounts for the remaining degrees of unsaturation. The cross peaks of H-18 with H-19, H-21 with H-22, H-22 with H-23, and H-23 with H-24 in the ^1^H-^1^H COSY experiment (Figure 2) and the HMBC correlations (Figure 2) from H-8 to C-14, from H-16 to C-14/C-18/C-25, from H-18 to C-17/C-20/C-25, from H-22 to C-20, and from H-23 to C-25 further supported the indole acetoxyl group in **4**. Therefore, the proposed structure of **4** was shown in Figure 1. According to the large *J*_H4-H5_ coupling constant (14.8) and the NOESY cross peaks of H-3 with H-5/H-15 and H_3_-7 with H-3/H-15, the configurations of the double bonds in **4** were also identified as 2*Z*,4*E*. A calculated ${\left[\alpha \right]}_{D}^{25}$ value (−14.5) of **4** was in consonance with the experimental value (−10.6) (Supplementary Table S1), indicating that the stereochemistry of **4** was 9*S*.

Dichocetide A (**5**) was isolated as a colorless oil and gave an HRESIMS ion peak at *m/z* 271.16654 [M + Na]^+^ (calcd. for 271.16685) (Supplementary Figure S33) that is indicative of the molecular formula of C_16_H_24_O_2_Na with five sites of unsaturation. The ^1^H, ^13^C NMR, DEPT and HMQC spectra (Table 1 and Table 3, Supplementary Figures S34–S40) displayed signals for four methyls, two methylenes, six methines, and four quaternary carbons. Both C-1 and C-15 are connected to hydroxyl groups based on their downfield chemical shifts and the molecular formula of **5**. The CH_3_CHCH(OH)CH(CH_3_)CH_2_ fragment was built from the ^1^H-^1^H COSY correlations (Figure 2) of H-2 with H-1/H-3/H-12 and of H-10 with H-1/H-11, and the CH_3_CH(OH)CH_2_ fragment was established based on the cross peaks of H-14 with H-15 and H-15 with H-16 in the ^1^H-^1^H COSY spectrum. Thorough analysis of the key HMBC cross peaks (Figure 2) from H-3 to C-4, from H-5 to C-3/C-7/C-9, from H-8 to C-10/C-14, from H-11 to C-9, from H-13 to C-5/C-6 and from H-14 to C-6/C-7 allowed us to connect the abovementioned fragments. Thus, the planar structure of **5** was established as shown in Figure 1, and the partially reduced naphthalene ring core of **5** accounts for the five degrees of unsaturation.

**Figure 3:**
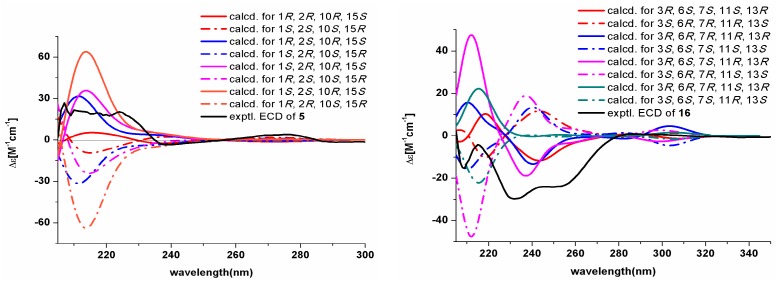
Comparison of the experimental and calculated ECD spectra of **5** and **16**.

The relative stereochemistry of **5** was confirmed by a NOESY experiment. The NOESY correlations of H-8 with H-10/H-15 suggested that H-10 and H-15 were located on the same side of the molecule as H-8. The experimental ECD spectrum (Figure 3) of **5** was identical to the curve calculated for (1*R*, 2*R*, 10*R*, 15*S*). Furthermore, the experimental optical rotation (23.0) is in accordance with the calculated value (25.1) (Supplementary Table S1), which supports the 1*R*, 2*R*, 10*R*, 15*S*-configuration of **5**.

Dichocerazine A (**15**) was isolated as a yellowish solid. The molecular formula of compound **15** was determined to be C_13_H_12_N_2_O_2_S from the HRESIMS data, which showed a molecular ion peak at *m/z* 261.0686 [M + H]^+^ (calcd. for 261.0692) (Supplementary Figure S59). This formula suggests nine degrees of unsaturation. The ^1^H NMR spectrum (Table 3 and Supplementary Figure S60) displayed signals indicative of two singlet methyls at *δ*_H_ 2.05 (s) and 3.24 (s), one sp^3^ methine at *δ*_H_ 5.04 (s), one aromatic proton at *δ*_H_ 7.46 (s) and a 1,2-disubstituted benzene ring at *δ*_H_ 7.40 (t, 8.0), 7.53 (t, 8.0), 7.70 (d, 8.0), and 8.42 (d, 8.0). The ^13^C NMR, in combination with the DEPT experiment (Table 1 and Supplementary Figures S61 and S62) showed two methyls, six methines, and five quaternary carbons. Careful analysis of the 1D NMR data of **15** revealed characteristic signals of a diketopiperazine that were similar to the characteristic signals of 1,2,3,4-tetrahydro-2,3-dimethyl-1,4-dioxopyrazino[1,2-a]indole [12], except for an S-methyl at *δ*_C_ 12.7 in **15** instead of the methyl at *δ*_C_ 19.8 of the latter. Detailed 2D NMR analyses validated the planar structure of **15**, which was depicted in Figure 1. The HMBC correlations (Figure 2) from H_3_-14 to C-1/C-3, from H-3 to C-4 and from H_3_-15 to C-3 supported the diketopiperazine framework. The ^1^H-^1^H COSY correlations (Figure 2) of H-7 with H-8, H-8 with H-9 and H-9 with H-10 combined with the HMBC cross peaks of H-12 with C-1, H-10 with C-11/C-12 and H-7 with C-6 allowed us to determine the structure of the remaining fragments. Compound **15** didn’t show optical activity in the optical rotation experiment, thus, this compound occurs as a racemate. The exhaustive effort to separate the enantiomers with HPLC using a Chiralcel OD column (250 mm × 10 mm) was unsuccessful.

Dichocerazine B (**16**) was acquired as a viscous yellow oil that gave an [M + Na]^+^ ion in the HRESIMS at *m/z* 453.0727 (calcd. for 453.0761) (Supplementary Figure S67). Its molecular formula was determined to be C_17_H_22_N_2_O_7_S_2_, which implies eight double bond equivalents. From the NMR data (Table 1 and Table 3, Supplementary Figures S68–S74), compound **16** was found to possess the same diketopiperazine skeleton as the 6-acetylbis (methylthio) gliotoxin previously isolated from *Neosartorya pseudofischeri* [12] based on the characteristic α-carbon signals of amino acid residues at *δ*_C_ 69.4 and 71.7 and the two amide carbonyls at *δ*_C_ 164.4 and 165.8. The presence of one *N*-methyl (*δ*_C_ 29.6), one methylene connected to an oxygen atom (*δ*_C_ 64.0), two S-methyls (*δ*_C_ 13.7 and 15.3), and the cross peaks from H-14 to C-1/C-13, from H-15 to C-1/C-3, from H-16 to C-3/C-4, and from H-17 to C-3 in the HMBC spectrum verified the diketopiperazine fragment. Further inspection of the remaining data in the 1D NMR and HMQC experiments displayed one singlet methyl, one methylene, two sp^2^ methines, two sp^3^ methines attached to heteroatoms, one quaternary carbon linked to a heteroatom, one ester carbonyl, and one keto-carbonyl. Based on the ^1^H-^1^H COSY correlations of H-9 with H-10 and H-6 with H-7, the two sp^2^ methines were a pair of olefinic methines (*δ*_C_ 127.2 and 148.4), and the two sp^3^ methines were adjacent aliphatic methines (*δ*_C_ 70.9 and 75.2). Detailed analyses of the HMBC correlations from H-6 to C-11/C-12/C-13, from H-12 to C-11/C-13, from H-7 to C-8/C-9/C-18, from H-9 to C-11, from H-10 to C-6/C-8, and from H-19 to C-18 confirmed the presence of a 6,5-fused ring system. In addition, the HMBC correlation of H-12 with C-1 explained the link between the diketopiperazine fragment and the 6,5-fused ring system. Consequently, the planar structure of **16** was constructed as shown in Figure 1.

The relative configuration of **16** was assigned by the magnitude of the coupling constant and the analysis of the NOESY spectrum (Supplementary Figure S74). The large *J*_H-6/H-7_ (11.2) coupling constants suggested that both H-6 and H-7 are axial. The NOESY correlations of H-6 with H-12′ and H-7 with H-12′ indicated that H-6 and H-7 are trans to each other. Comparing the experimental CD curve and the ${\left[\alpha \right]}_{D}^{25}$ value (−60.5) of **16** with the calculated ECD spectrum (Figure 3) and the optical rotation (−59.6) (Supplementary Table S1), respectively, the stereochemistry of **16** was confirmed to be 3*R*, 6*S*, 7*S*, 11*S*, 13*R*.

According to a comparison of the spectroscopic data of compounds **6**–**14** and **17**–**28** (Supplementary Figures S41–S58 and S75–S98) with literature reports, their chemical structures were identified as dichotone A (**6**) [13], diorcinol (**7**) [14], 3-*O*-methyldiorcinol (**8**) [14], 5,5′-oxybis(1-methoxy-3-methylbenzene) (**9**) [15], dibutyl phthalate (**10**) [16], butyl (2-ethylhexyl) phthalate (**11**) [17], (2a*R*, 5*R*, 5a*R*, 8*S*, 8a*S*)-2,2,5,8-tetramethyldecahydro-2H-naphtho[1,8-bc]furan-5-ol (**12**) [18], aspewentin A (**13**) [19], JBIR-03 (**14**) [20], dichotocejpin A (**17**) [21], didehydrobisdethiobis (methylthio) gliotoxin (**18**) [12], bisdethiobis (methylthio) gliotoxin (**19**) [10], 6-acetylbis (methylthio) gliotoxin (**20**) [12], haematocin (**21**) [10], pityriacitrin (**22**) [22], stellarine A (**23**) [23], perlolyrine (**24**) [24], fiscalin C (**25**) [25], epi-fiscalin C (**26**) [25], indolyl-3-acetic acid methyl ester (**27**) [26], and anthranilic acid (**28**) [27].

### 2.2. Biological Activity

The cytotoxicities of compounds **1**, **7**, **8**, **11**, **15**, **22**, **23**, and **27** were evaluated against the human colon cancer cell line HCT116, human rhabdomyosarcoma cell line RD, human renal carcinoma cell line ACHN, and human ovarian cancer cell line A2780T. Dichotomocej A (**1**) exhibited a moderate inhibitory effect against RD with an IC_50_ value of 39.1 µM, and pityriacitrin (**22**) exhibited a moderate inhibitory effect against HCT116 with an IC_50_ value of 35.1 µM.

The antibacterial activities of compounds **4**, **8**, **13**, **14**, **22**, and **24** were screened against *Staphylococcus aureus* ATCC29213, *Escherichia coli* ATCC25922, *Pseudomonas aeruginosa* ATCC27853, and *Bauman's acinetobacter* ATCC19606. However, no significant inhibitory effects were observed for these compounds against these four bacterial strains.

## 3. Materials and Methods

### 3.1. General Experimental Procedures

Column chromatography was carried out on silica gel (SiO_2_, 200–300 mesh, Qingdao Marine Chemical Inc., Qingdao, Shandong, China) and Sephadex LH-20 (green herbs, Beijing, China). Preparative HPLC was performed using a Shim-pack PRC-ODS HPLC column (250 × 20 mm, Shimadzu Corporation, Nakagyo-ku, Kyoto, Japan) and a Shimadzu LC-20AT HPLC pump (Shimadzu Corporation, Nakagyo-ku, Kyoto, Japan) installed with an SPD-20A dual λ absorbance detector (Shimadzu Corporation, Nakagyo-ku, Kyoto, Japan). 1D and 2D NMR spectra were measured on Bruker Avance II 400 spectrometers (Bruker BioSpin AG, Industriestrasse 26, Fällanden, Switzerland), and the chemical shifts are relative to the residual solvent signals (CDCl_3_: *δ*_H_ 7.260 and *δ*_C_ 77.160; Acetone-*d*_6_: *δ*_H_ 2.050 and *δ*_C_ 29.840; DMSO-*d*_6_: *δ*_H_ 2.500 and *δ*_C_ 39.520). Mass spectra were performed on Thermo DSQ ESI low-resolution and Thermo MAT95XP ESI high-resolution mass spectrometers (Thermo Fisher Scientific Inc., Waltham, MA, USA). UV spectra were acquired on a Shimadzu UV-Vis-NIR spectrophotometer (Shimadzu Corporation, Nakagyo-ku, Kyoto, Japan). IR spectra were recorded on a PerkinElmer Frontier FT-IR spectrophotometer (PerkinElmer Inc., Waltham, MA, USA). Optical rotations were recorded on a Schmidt and Haensch Polartronic HNQW5 optical rotation spectrometer (SCHMIDT + HAENSCH GmbH & Co., Berlin, Germany). CD spectra were obtained using a JASCO J-810 circular dichroism spectrometer (JASCO International Co. Ltd., Hachioji, Tokyo, Japan).

### 3.2. Fungal Material

The marine fungus *Dichotomomyces cejpii* F31-1 was obtained from the inner tissue of the soft coral *Lobophytum crassum* collected from Hainan Sanya National Coral Reef Reserve, China. This fungal strain was conserved in 15% (*v*/*v*) glycerol aqueous solution at −80 °C. A voucher specimen was deposited in the School of Pharmaceutical Sciences, Sun Yat-sen University, Guangzhou, China. Analysis of the ITS rDNA (GenBank EF669956) by BLAST database screening provided a 100% match to *Dichotomomyces cejpii*.

### 3.3. Culture, Extraction, and Isolation

The marine fungus *Dichotomomyces cejpii* was cultured in the medium which contained 20 g/L glucose, 5 g/L peptone, 2 g/L yeast extract, 2 g/L Trp, 2 g/L Phe, 30 g/L sea salt, and 1 L H_2_O at pH 7.5. Fungal mycelia were cut and transferred aseptically to 1 L Erlenmeyer flasks, each adding 600 mL of sterilized liquid medium. The flasks were incubated at 25 °C for 60 days. Ninety liters of liquid culture were filtered through cheesecloth to separate the culture broth and the mycelia. The culture broth was successively extracted three times with EtOAc (90 L) and then was concentrated by low-temperature rotary evaporation to give a crude extract (35 g).

The extract was chromatographed on a silica gel column (diameter: 8 cm, length: 80 cm, silica gel: 300 g) with a gradient of petroleum ether-EtOAc (10:0–0:10, *v*/*v*) followed by EtOAc-MeOH (10:0–0:10, *v*/*v*) to afford 12 fractions (Fr. 1–Fr. 12). Fr. 2 was purified by silica gel column using a step gradient elution with petroleum ether-EtOAc (10:0–0:10, *v*/*v*) to get 10 subfractions (Fr. 2-1–Fr. 2-10) after gathering the similar fractions as monitored by TLC analyses. Fr. 2-8 was seperated via Sephadex LH-20 (MeOH) to give compounds **4** (17.2 mg), **8** (7.3 mg), and **22** (23.8 mg). Compounds **2** (2.0 mg) and **3** (3.5 mg) were obtained from Fr. 4 with a preparative RP HPLC with MeOH-H_2_O (61:39, *v*/*v*). Fr. 5 was purified by the recrystallization in the CHCl_3_-acetone (2:1, *v*/*v*) solution to afford compounds **23** (90.0 mg) and **24** (55.6 mg). Fr. 6 and Fr. 7 were subjected to a Sephadex LH-20 column and eluted with CH_2_Cl_2_-MeOH (1:1, *v*/*v*) to give three sub-fractions (Fr. 6-1–Fr. 6-3 and Fr. 7-1–Fr. 7-3) respectively. Then compounds **1** (7.2 mg), **13**(13.3 mg)**, 14** (0.6 mg), **15** (11.0 mg), and **27** (17.5 mg) were obtained from Fr. 6-2, which is chromatographed on silica gel column using a step gradient elution with CHCl_3_-EtOAc (10:0–0:10, *v*/*v*). Fr. 7-1 was further purified with a preparative RP HPLC (MeCN-H_2_O, 35:65, *v*/*v*) to acquire compounds **11** (11.0 mg), **16** (7.6mg), **5** (0.9 mg), and **26** (8.7 mg). Fr. 8 was recrystallized from MeOH to yield compound **18** (100.0 mg), while Fr. 9 was recrystallized from CHCl_3_ to produce compound **28** (32.0 mg). The mother liquid of Fr. 8 was further purified using reversed phase preparative HPLC with a mobile phase of MeOH-H_2_O (50:50, *v*/*v*) to obtain compounds **7** (70.8 mg), **19** (98.0 mg), **20** (110.2 mg), and **21** (6.8 mg). The mother liquid of Fr. 9 was isolated using Sephadex LH-20 (MeOH) to yield compounds **10** (27.5 mg) and **6** (1.8 mg). HPLC purification of Fr. 10 with solvent system MeCN-H2O (26:74, *v*/*v*) gave compounds **12** (10.2 mg) and **25** (4.5 mg). Finally, compounds **9** (6.9 mg) and **17** (7.0 mg) were separated by RP-HPLC with MeOH-H_2_O (66:34, *v*/*v*) of Fr. 10.

Dichotomocej A (**1**): pale yellow oil; ${\left[\alpha \right]}_{D}^{25}$ = −41.9 (*c* 0.30, CHCl_3_). UV (MeCN) *λ*_max_ nm (log *ε*): 192 (3.87), 253 (3.90). IR (KBr) *ν*_max_ 3367, 3233, 2957, 2926, 2870, 1652, 1629, 1530, 1378, 1262, 1050, 881 cm^−1^. ^1^H and ^13^C NMR data see Table 1 and Table 2. HRESIMS *m/z* 226.1809 [M + H]^+^ (calcd. for C_13_H_23_NO_2_, 226.1802).

Dichotomocej B (**2**): yellowish oil; ${\left[\alpha \right]}_{D}^{25}$ = −4.4 (*c* 0.20, CHCl_3_). UV (MeCN) *λ*_max_ nm (log *ε*): 193 (3.95), 245 (3.67). IR (KBr) *ν*_max_ 3377, 3223, 2959, 2926, 2855, 1652, 1529, 1378, 1261, 1051, 968, 804 cm^−1^. ^1^H and ^13^C NMR data see Table 1 and Table 2. HRESIMS *m/z* 240.1955 [M + H]^+^ (calcd. for C_14_H_25_NO_2_, 240.1958).

Dichotomocej C (**3**): yellowish oil; ${\left[\alpha \right]}_{D}^{25}$ = −51.6 (*c* 0.40, CHCl_3_). UV (MeCN) *λ*_max_ nm (log *ε*): 194 (4.35), 252 (4.05). IR (KBr) *ν*_max_ 3354, 2956, 2926, 2855, 1740, 1657, 1207, 1160 cm^−1^. ^1^H and ^13^C NMR data see Table 1 and Table 2. HRESIMS *m/z* 254.1750 [M + H]^+^ (calcd. for C_14_H_23_NO_3_, 254.1751).

Dichotomocej D (**4**): yellowish oil; ${\left[\alpha \right]}_{D}^{25}$ = −10.6 (*c* 0.20, CHCl_3_). UV (MeCN) *λ*_max_ nm (log *ε*): 192 (4.59), 220 (4.69), 257 (4.50). IR (KBr) *ν*_max_ 3389, 3233, 2957, 2926, 2870, 1727, 1633, 1514, 1457, 1260, 1157, 969, 737 cm^−1^. ^1^H and ^13^C NMR data see Table 1 and Table 2. HRESIMS *m/z* 383.2296 [M + H]^+^ (calcd. for C_23_H_30_N_2_O_3_, 383.2329).

Dichocetide A (**5**): colorless oil; ${\left[\alpha \right]}_{D}^{25}$ = 23.0 (*c* 0.10, MeOH). CD (MeOH): 217 (Δ*ε* +21.4), 235 (Δ*ε* 0), 239 (Δ*ε* −4.8), 257 (Δ*ε* 0). UV (MeOH) *λ*_max_ nm (log *ε*): 202 (4.46), 270 (3.06), 280 (3.01). IR (KBr) *ν*_max_ 3201, 2970, 2926, 2907, 2857, 1739, 1376, 1263, 1051, 803 cm^−1^. ^1^H and ^13^C NMR data see Table 1 and Table 3. HRESIMS *m/z* 271.16654 [M + Na]^+^ (calcd. for C_16_H_24_O_2_Na, 271.16685).

(±)-Dichocerazine A (**15**): yellowish solid; ${\left[\alpha \right]}_{D}^{25}$ = 0 (*c* 0.20, MeOH). UV (MeOH) *λ*_max_ nm (log *ε*): 202 (4.32), 245 (4.14), 272 (3.87), 298 (4.00). IR (KBr) *ν*_max_ 2926, 1712, 1651, 1588, 1569, 1429, 1385, 1359, 1333, 1256, 1207, 1019, 845, 749, 734 cm^−1^.^1^H and ^13^C NMR data see Table 1 and Table 3. HRESIMS *m/z* 261.0686 [M + H]^+^ (calcd. for C_13_H_12_N_2_O_2_S, 261.0692).

Dichocerazine B (**16**): viscous yellow oil; ${\left[\alpha \right]}_{D}^{25}$ = −60.5 (*c* 0.20, MeOH). CD (MeOH): 217 (Δ*ε* −4.6), 231 (Δ*ε* −29.8), 282 (Δ*ε* 0). UV (MeOH) *λ*_max_ nm (log *ε*): 202 (4.21), 285 (3.39). IR (KBr) *ν*_max_ 3370, 2957, 2926, 2854, 1743, 1651, 1419, 1377, 1222, 1039 cm^−1^. ^1^H and ^13^C NMR data see Table 1 and Table 3. HRESIMS *m/z* 453.0727 [M + Na]^+^ (calcd. for C_17_H_22_N_2_O_7_S_2_, 453.0761).

### 3.4. Computational Methods

The absolute configurations of compounds **1**–**5** and **16** were determined by calculations of optical rotation values and ECD spectra. Both geometry analyses and all calculations of optical properties have been carried out using the Gaussian 09 software [28,29] and the theory of Boltzmann weights at room temperature. The stationary conformers with the lowest energy of compounds **1**–**5** and **16** were geometrically optimized by the DFT method at the B3LYP/6-31+G (d) level. The calculations of optical rotation values were performed by the TDDFT method at the B3LYP/6-31+G (d) level in chloroform and methanol [29]. The ECD spectra of the different conformers were obtained using the TDDFT method at the PBE1PBE/6-311++G (d, p) level in methanol [30]. Additionally, the ECD spectra were generated from dipole-length dipolar and rotational strengths using a Gaussian band shape with a 0.3 eV exponential half-width and elaborated using the SpecDis program [31].

### 3.5. Cytotoxic Assay

The cytotoxic activities of the tested compounds against cancer cell lines were determined using sulforhodamine B (SRB) colorimetric method. Firstly, cells were seeded in 96 well plates in a volume of 100 μL/well (5000–40,000 cells per well). After 24 h incubation at 37 °C in a humidified incubator with 5% CO_2_, the cells were treated with 100 μL medium containing tested compounds (2X indicated concentrations) for 72 h. Secondly, 50 μL cold 50% (*w*/*v*) trichloroacetic acid (TCA) was applied to fix the attached cells for 1 h at 4 °C, and then 100 μL 0.4% (*w*/*v)* SRB was used for a stain of the attached cells. Finally, the protein-bound dye was solubilized with 200 μL 10 mM Tris base solution (pH 10.5) for absorbency determination at 515 nm by using SpectraMax 190 microplate reader (Molecular Devices). When the concentration was displayed as a 50% reduction in the process of cell growth, the IC_50_ value was defined.

### 3.6. Antimicrobial Activity

According to the National Committee for Clinical Laboratory Standards (NCCLS) standard, the antimicrobial experiments were performed using a broth dilution method (Mueller-Hinton broth). The tested bacteria were grown in liquid MH medium (2 g/L beef powder, 1.5 g/L soluble starch, 17.5 g/L acid hydrolyzed casein, PH = 7.4), and 50 µL of bacterial suspension (1.5 × 10^6^ CFU/mL) were seeded in 96 well plates. Then the test compounds (50 µL) with different concentrations were added into each well, 256 μg/mL was a starting concentration to screen the potential antimicrobial activities of the tested compounds. The bacterial suspension without compounds was used as a positive control, while the MH medium was used as the negative control. After incubation at 37 °C in an electro-heating standing-temperature cultivator, the growth of the test organisms was inhibited completely with a lowest concentration. In this way, the minimum inhibitory concentration (MIC) of the tested compounds was defined. What’s more, the OD determination at 595 nm were measured by a multifunction microplate reader (PowerWaveTMXS2, BioTek^®^ Instruments Inc., Winooski, VT, USA).

## 4. Conclusions

In this study, twenty-eight compounds in total were obtained from the marine-derived fungus *Dichotomomyces cejpii* F31-1. Their structures included amides, polyketides, a sesquiterpenoid, a diterpenoid, a meroterpenoid, diketopiperazines, β-carbolines, fumiquinazolines and indole alkaloids, which demonstrated the tremendous biosynthetic potential of the investigated fungal strain. Seven diketopiperazines (**15**–**21**), four indole-related alkaloids (**22**–**24**, **27**), and seven polyketides (**5**–**11**) had been previously reported from *Dichotomomyces cejpii* in the literature, but four novel aliphatic amides (**1**–**4**) and two fumiquinazoline (**25**–**26**) alkaloids were also obtained. It was proposed that the fumiquinazolines are related to amino acids supplementation in the medium, since *Scedosporium apiospermum* F41-1 produced predominately fumiquinazolines when the medium was doped with exogeneous amino acids [9]. Obviously, an amino acid–directed strategy is effective for promoting the production of nitrogen-containing compounds by *Dichotomomyces cejpii*. Anthranilic acid, a common biosynthetic precursor of fumiquinazolines, was also isolated with the fumiquinazolines. Additionally, *Dichotomomyces cejpii* also afforded indolyl-3-acetic acid methyl ester, which is apparently derived from tryptophan. Overall, the amino acids Trp and Phe in the culture medium of F31-1 may contribute to the generation and diversity of the nitrogen-containing compounds. Furthermore, the terpenoids (**12** and **13**) were the first of their chemical class reported from the genus *Dichotomomyces*.

## Figures and Tables

**Table 1:** ^13^C NMR data for compounds **1**–**5** and **15**–**16** (100 MHz, CDCl_3_).

No.	1	2	3	4	5	15	16
1	170.4, C	170.3, C	169.1, C	168.9, C	75.8, CH	156.7, C	165.8, C
2	127.3, C	127.2, C	127.3, C	127.3, C	31.0, CH	N	N
3	134.6, CH	134.6, CH	134.5, CH	134.1, CH	36.3, CH_2_	66.8, CH	71.7, C
4	127.2, CH	127.1, CH	127.2, CH	127.1, CH	133.4, C	161.8, C	164.4, C
5	136.7, CH	136.7, CH	136.6, CH	136.2, CH	130.5, CH	N	N
6	18.9, CH_3_	19.0, CH_3_	18.9, CH_3_	18.9, CH_3_	134.6, C	129.2, C	70.9, CH
7	13.0, CH_3_	13.0, CH_3_	12.9, CH_3_	12.6, CH_3_	134.4, C	116.5, CH	75.2, CH
8	67.1, CH_2_	66.4, CH_2_	174.0, C	66.6, CH_2_	130.6, CH	128.3, CH	190.9, C
9	50.7, CH	50.5, CH	51.1, CH	47.0, CH	138.6, C	125.7, CH	127.2, CH
10	40.5, CH_2_	38.3, CH_2_	42.1, CH_2_	40.8, CH_2_	37.8, CH	122.8, CH	148.4, CH
11	25.3, CH	31.5, CH	25.1, CH	24.9, CH	17.2, CH_3_	134.9, C	76.4, C
12	23.2, CH_3_	29.1, CH_2_	22.9, CH_3_	23.0, CH_3_	18.2, CH_3_	115.1, CH	51.1, CH_2_
13	22.4, CH_3_	11.2, CH_3_	22.3, CH_3_	22.4, CH_3_	19.3, CH_3_	127.9, C	69.4, C
14	NH	19.6, CH_3_	52.4, CH_3_	172.3, C	43.0, CH_2_	32.2, CH_3_	15.3, CH_3_
15		NH	NH	NH	68.1, CH	12.7, CH_3_	29.6, CH_3_
16				31.5, CH_2_	23.2, CH_3_		64.0, CH_2_
17				108.4, C			13.7, CH_3_
18				123.3, CH			170.1, C
19				NH			20.7, CH_3_
20				136.3, C			
21				111.5, CH			
22				122.3, CH			
23				119.8, CH			
24				118.8, CH			
25				127.4, C			

**Table 2:** ^1^H NMR data for compounds **1**–**4** (400 MHz, CDCl_3_).

No.	1	2	3	4
3	6.88 (d, 11.2)	6.88 (d, 11.2)	6.88 (d, 10.8)	6.76 (d, 11.2)
4	6.33 (ddq, 13.2, 11.2, 1.6)	6.31 (ddq, 14.8, 11.2, 1.6)	6.32 (ddq, 15.2, 10.8, 1.6)	6.27 (ddq, 14.8, 11.2, 1.6)
5	6.04 (dq, 13.2, 6.8)	6.02 (dq, 14.8, 6.8)	6.03 (dq, 15.2, 6.8)	5.97 (dq, 14.8, 6.8)
6	1.86 (d, 6.4)	1.85 (d, 6.8)	1.85 (d, 6.4)	1.85 (d, 6.8)
7	1.94 (s)	1.93 (s)	1.95 (s)	1.72 (s)
8	3.57 (dd, 10.8, 6.0); 3.72 (dd, 10.8, 3.2)	3.55 (dd, 10.8, 6.0); 3.71 (dd, 10.8, 3.2)		4.09 (dd, 11.2, 4.0); 4.20 (dd, 11.2, 5.2)
9	4.12 (m)	4.11 (m)	4.71 (td, 8.4, 5.2)	4.33 (m)
10	1.41 (dt, 8.4, 6.0)	1.31 (dd, 13.2, 6.0); 1.54 (dt, 13.2, 6.4)	1.58 (m); 1.70 (m)	1.24 (m)
11	1.66 (m)	1.44 (m)	1.67 (m)	1.52 (m)
12	0.95 (d, 6.8)	1.14 (t, 6.8); 1.39 (m)	0.95 (d, 6.4)	0.85 (d, 6.4)
13	0.94 (d, 6.8)	0.86 (t, 6.8)	0.95 (d, 6.4)	0.85 (d, 6.4)
14	5.77 (brd, 6.4)	0.92 (d, 6.4)	3.74 (s)	
15		5.87 (brd, 7.2)	6.10 (d, 8.0)	5.54 (d, 8,8)
16				3.79 (s)
18				7.11 (s)
19				8.38 (brs)
21				7.34 (d, 8.0)
22				7.18 (dd, 8.0, 8.0)
23				7.11 (dd, 8.0, 8.0)
24				7.61 (d, 8.0)

**Table 3:** ^1^H NMR data for compounds **5** and **15–16** (400MHz, CDCl_3_).

No.	5	15	16
1	3.71 (dd, 9.2, 4.8)		
2	2.07, m		
3	2.41 (dd, 16.8, 9.6); 2.92 (dd, 16.8, 6.4)	5.04 (s)	
5	6.87 (s)		
6			5.14 (d, 11.2)
7		8.42 (d, 8.0)	5.89 (d, 11.2)
8	6.94 (s)	7.53 (t, 8.0)	
9		7.40 (t, 8.0)	6.10 (d, 10.4)
10	3.03 (m)	7.70 (d, 8.0)	6.92 (d, 10.4)
11	1.25 (d, 7.2)		
12	1.11 (d, 6.4)	7.46 (s)	2.80 (d, 16.0); 3.42 (d, 16.0)
13	2.26 (s)		
14	2.67 (dd, 13.6, 8.4); 2.76 (dd, 13.6, 4.4)	3.24 (s)	2.23 (s)
15	4.00, m	2.05 (s)	3.10 (s)
16	1.27 (d, 6.4)		3.85 (d, 12.0); 4.31 (d, 12.0)
17			2.19 (s)
19			2.17 (s)

## Supplementary Materials

The following are available online at www.mdpi.com/1660-3397/15/11/339/s1. Table S1: Comparison of the experimental optical rotatation values with the calculated OR values of compounds **1**–**5** and **16**; Figure S1: The most stable conformers of **1**–**5**, **16**; Figure S2: HR-ESI-MS spectrum of compound **1**; Figure S3–S8: 1D and 2D NMR spectra of compound **1**; Figure S9: HR-ESI-MS spectrum of compound **2**; Figure S10–S16: 1D and 2D NMR spectra of compound **2**; Figure S17: HR-ESI-MS spectrum of compound **3**; Figure S18–S24: 1D and 2D NMR spectra of compound **3**; Figure S25 HR-ESI-MS spectrum of compound **4**;Figure S26–S32: 1D and 2D NMR spectra of compound **4**; Figure S33: HR-ESI-MS spectrum of compound **5**; Figure S34–S40: 1D and 2D NMR spectra of compound **5**; Figures S41 and S42: 1D NMR spectra of compound **6**; Figures S43 and S44: 1D NMR spectra of compound **7**; Figures S45 and S46: 1D NMR spectra of compound **8**; Figures S47 and S48: 1D NMR spectra of compound **9**; Figures S49 and S50: 1D NMR spectra of compound **10**; Figures S51 and S52: 1D NMR spectra of compound **11**; Figures S53 and S54: 1D NMR spectra of compound **12**; Figures S55 and S56: 1D NMR spectra of compound **13**; Figures S57 and S58: 1D NMR spectra of compound **14**; Figure S59: HR-ESI-MS spectrum of compound **15**; Figures S60–S66: 1D and 2D NMR spectra of compound **15**; Figure S67: HR-ESI-MS spectrum of compound **16**; Figures S68–S74: 1D and 2D NMR spectra of compound **16**; Figures S75 and S76: 1D NMR spectra of compound **17**; Figures S77 and S78: 1D NMR spectra of compound **18**; Figures S79 and S80: 1D NMR spectra of compound **19**; Figures S81 and S82: 1D NMR spectra of compound **20**; Figures S83 and S84: 1D NMR spectra of compound **21**; Figures S85 and S86: 1D NMR spectra of compound **22**; Figures S87 and S88: 1D NMR spectra of compound **23**; Figures S89 and S90: 1D NMR spectra of compound **24**; Figures S91 and S92: 1D NMR spectra of compound **25**; Figures S93 and S94: 1D NMR spectra of compound **26**; Figures S95 and S96: 1D NMR spectra of compound **27**; Figures S97 and S98: 1D NMR spectra of compound **28**.
