# Supplementary material for: Diverse Secondary Metabolites from the Marine-Derived Fungus Dichotomomyces cejpii F31-1
Source: Mar Drugs. 2017 Nov 1;15(11):339. doi: 10.3390/md15110339 (PMC5706029; doi:10.3390/md15110339)

Supporting Information

Diverse secondary metabolites from the marine-derived fungus *Dichotomomyces cejpii* F31-1

Yan-Xiu Chen [1,5], Meng-Yang Xu [1], Hou-Jin Li [2], Kun-Jiao Zeng [3], Wen-Zhe Ma [4], Guo-Bao Tian [3], Jun Xu [1], De-Po Yang [1,5], Wen-Jian Lan [1,5,*]

[1] School of Pharmaceutical Sciences, Sun Yat-sen University, Guangzhou 510006, China; chenyx239@mail2.sysu.edu.cn; xumy3@mail2.sysu.edu.cn; junxu@biochemomes.com;lssydp@mail.sysu.edu.cn; lanwj@mail.sysu.edu.cn
[2] School of Chemistry, Sun Yat-sen University, Guangzhou 510275, China; ceslhj@mail.sysu.edu.cn
[3] Zhongshan School of Medicine, Sun Yat-sen University, Guangzhou 510080, China; zkj3880@163.com; tiangb@mail.sysu.edu.cn
[4] State Key Laboratory of Quality Research in Chinese Medicine, Macau Institute for Applied Research in Medicine and Health, Macau University of Science and Technology, Avenida Wai Long, Taipa 519020, Macau (SAR), China; wzma@must.edu.mo
[5] Guangdong Technology Research Center for Advanced Chinese Medicine, Guangzhou 510006, China
Correspondence: lanwj@mail.sysu.edu.cn; Tel.: +86-020-3994-3042

List of Contents

Table S1. Comparison of the experimental optical rotatation values with the calculated OR values of compounds **1–5** and **16**........S1
Figure S1. The most stable conformers of **1–5**, **16**.........S2
Figure S2. HR-ESI-MS spectrum of dichotomocej A (**1**)........S18
Figure S3. ^{1}H NMR spectrum of dichotomocej A (**1**) in $CDCl_3$ (400MHz)........S19
Figure S4. ^{13}C NMR spectrum of dichotomocej A (**1**) in $CDCl_3$ (100MHz)........S20
Figure S5. HMQC spectrum of dichotomocej A (**1**) in $CDCl_3$........S21
Figure S6. ^{1}H-^{1}H COSY spectrum of dichotomocej A (**1**) in $CDCl_3$........S22
Figure S7. HMBC spectrum of dichotomocej A (**1**) in $CDCl_3$........S23
Figure S8. NOESY spectrum of dichotomocej A (**1**) in $CDCl_3$........S24

Figure S9. HR-ESI-MS spectrum of dichotomocej B (**2**)....S25
Figure S10. ^{1}H NMR spectrum of dichotomocej B (**2**) in $CDCl_3$ (400MHz)....S26
Figure S11. ^{13}C NMR spectrum of dichotomocej B (**2**) in $CDCl_3$ (100MHz)....S27
Figure S12. DEPT 135 spectum of dichotomocej B (**2**) in $CDCl_3$ (100MHz)....S28
Figure S13. HMQC spectrum of dichotomocej B (**2**) in $CDCl_3$....S29
Figure S14. ^{1}H-^{1}H COSY spectrum of dichotomocej B (**2**) in $CDCl_3$....S30
Figure S15. HMBC spectrum of dichotomocej B (**2**) in $CDCl_3$....S31
Figure S16. NOESY spectrum of dichotomocej B (**2**) in $CDCl_3$....S32
Figure S17. HR-ESI-MS spectrum of dichotomocej C (**3**)....S33
Figure S18. ^{1}H NMR spectrum of dichotomocej C (**3**) in $CDCl_3$ (400MHz)....S34
Figure S19. ^{13}C NMR spectrum of dichotomocej C (**3**) in $CDCl_3$ (100MHz)....S35
Figure S20. DEPT 135 spectrum of dichotomocej C (**3**) in $CDCl_3$ (100MHz)....S36
Figure S21. HMQC spectrum of dichotomocej C (**3**) in $CDCl_3$....S37
Figure S22. ^{1}H-^{1}H COSY spectrum of dichotomocej C (**3**) in $CDCl_3$....S38
Figure S23. HMBC spectrum of dichotomocej C (**3**) in $CDCl_3$....S39
Figure S24. NOESY spectrum of dichotomocej C (**3**) in $CDCl_3$....S40
Figure S25. HR-ESI-MS spectrum of dichotomocej D (**4**)....S41
Figure S26. ^{1}H NMR spectrum of dichotomocej D (**4**) in $CDCl_3$ (400MHz)....S42
Figure S27. ^{13}C NMR spectrum of dichotomocej D (**4**) in $CDCl_3$ (100MHz)....S43
Figure S28. DEPT 135 spectum of dichotomocej D (**4**) in $CDCl_3$ (100MHz)....S44
Figure S29. HMQC spectrum of dichotomocej D (**4**) in $CDCl_3$....S45
Figure S30. ^{1}H-^{1}H COSY spectrum of dichotomocej D (**4**) in $CDCl_3$....S46
Figure S31. HMBC spectrum of dichotomocej D (**4**) in $CDCl_3$....S47
Figure S32. NOESY spectrum of dichotomocej D (**4**) in $CDCl_3$....S48
Figure S33. HR-ESI-MS spectrum of dichocetide A (**5**)....S49
Figure S34. ^{1}H NMR spectrum of dichocetide A (**5**) in $CDCl_3$ (400MHz)....S50
Figure S35. ^{13}C NMR spectrum of dichocetide A (**5**) in $CDCl_3$ (100MHz)....S51
Figure S36. DEPT 135 spectrum of dichocetide A (**5**) in $CDCl_3$ (100MHz)....S52
Figure S37. HMQC spectrum of dichocetide A (**5**) in $CDCl_3$....S53
Figure S38. ^{1}H-^{1}H COSY spectrum of dichocetide A (**5**) in $CDCl_3$....S54
Figure S39. HMBC spectrum of dichocetide A (**5**) in $CDCl_3$....S55
Figure S40. NOESY spectrum of dichocetide A (**5**) in $CDCl_3$....S56
Figure S41. ^{1}H NMR spectrum of dichotone A (**6**) in $CDCl_3$ (400MHz)....S57
Figure S42. ^{13}C NMR spectrum of dichotone A (**6**) in $CDCl_3$ (100MHz)....S58
Figure S43. ^{1}H NMR spectrum of diorcinol (**7**) in $CDCl_3$ (400MHz)....S59
Figure S44. ^{13}C NMR spectrum of diorcinol (**7**) in $CDCl_3$ (100MHz)....S60
Figure S45 ^{1}H NMR spectrum of 3-O-methyldiorcinol (**8**) in $CDCl_3$ (400MHz)....S61
Figure S46. ^{13}C NMR spectrum of 3-O-methyldiorcinol (**8**) in $CDCl_3$ (100MHz)....S62
Figure S47. ^{1}H NMR spectrum of 5,5'-oxybis (1-methoxy-3-methylbenzene) (**9**) in $CDCl_3$ (400MHz)....S63
Figure S48. ^{13}C NMR spectrum of 5,5'-oxybis (1-methoxy-3-methylbenzene) (**9**) in $CDCl_3$ (100MHz)....S64
Figure.S49. ^{1}H NMR spectrum of dibutyl phthalate (**10**) in $CDCl_3$ (400MHz)....S65
Figure S50. ^{13}C NMR spectrum of dibutyl phthalate (**10**) in $CDCl_3$ (100MHz)....S66

Figure S51. ^{1}H NMR spectrum of butyl (2-ethylhexyl) phthalate (**11**) in $CDCl_3$ (400MHz).......S67
Figure S52. ^{13}C NMR spectrum of butyl (2-ethylhexyl) phthalate (**11)** in $CDCl_3$ (100MHz)......S68
Figure S53. ^{1}H NMR spectrum of (2a*R*,5*R*,5a*R*,8*S*,8a*S*)-2,2,5,8-tetramethyldecahydro-2H-naphtho[1,8-bc]furan-5-ol (**12**) in $CDCl_3$ (400MHz)……...S69
Figure S54. ^{13}C NMR spectrum of (2a*R*,5*R*,5a*R*,8*S*,8a*S*)-2,2,5,8-tetramethyldecahydro-2H-naphtho[1,8-bc]furan-5-ol (**12**) in $CDCl_3$ (100MHz)..S70
Figure S55. ^{1}H NMR spectrum of aspewentin A (**13**) in $CDCl_3$ (400MHz).............................S71
Figure S56. ^{13}C NMR spectrum of aspewentin A (**13**) in $CDCl_3$ (100MHz).............................S72
Figure S57. ^{1}H NMR spectrum of JBIR-03 (**14**) in $CDCl_3$ (400MHz)..S73
Figure S58. ^{13}C NMR spectrum of JBIR-03 (**14**) in $CDCl_3$ (100MHz).......................................S74
Figure S59. HR-ESI-MS spectrum of dichocerazine A (**15**)..S75
Figure S60. ^{1}H NMR spectrum of dichocerazine A (**15**) in $CDCl_3$ (400MHz)............................S76
Figure S61. ^{13}C NMR spectrum of dichocerazine A (**15**) in $CDCl_3$ (100MHz)...........................S77
Figure S62. DEPT 135 spectrum of dichocerazine A (**15**) in $CDCl_3$ (100MHz).........................S78
Figure S63. HMQC spectrum of dichocerazine A (**15**) in $CDCl_3$...S79
Figure S64. ^{1}H-^{1}H COSY spectrum of dichocerazine A (**15**) in $CDCl_3$......................................S80
Figure S65. HMBC spectrum of dichocerazine A (**15**) in CDCl3...S81
Figure S66. NOESY spectrum of dichocerazine A (**15**) in CDCl3..S82
Figure S67. HR-ESI-MS spectrum of dichocerazine B (**16**)...S83
Figure S68. ^{1}H NMR spectrum of dichocerazine B (**16**) in CDCl3 (400MHz)...........................S84
Figure S69. ^{13}C NMR spectrum of dichocerazine B (**16**) in CDCl3 (100MHz)..........................S85
Figure S70. DEPT 135 spectrum of dichocerazine B (**16**) in CDCl3 (100MHz)...S86
Figure S71. HMQC spectrum of dichocerazine B (**16**) in CDCl3..S87
Figure S72. ^{1}H-^{1}H COSY spectrum of dichocerazine B (**16**) in CDCl3......................................S88
Figure S73. HMBC spectrum of dichocerazine B (**16**) in CDCl3..S89
Figure S74. NOESY spectrum of dichocerazine B (**16**) in CDCl3..S90
Figure S75. ^{1}H NMR spectrum of dichotocejpin A (**17**) in $CDCl_3$ (400MHz)............................S91
Figure S76. ^{13}C NMR spectrum of dichotocejpin A (**17**) in $CDCl_3$ (100MHz)...........................S92
Figure S77. ^{1}H NMR spectrum of didehydrobisdethiobis (methy1thio) gliotoxin (**18**) in $CDCl_3$ (400MHz)...S93
Figure S78. ^{13}C NMR spectrum of didehydrobisdethiobis (methy1thio) gliotoxin (**18**) in $CDCl_3$ (100MHz)...S94
Figure S79. ^{1}H NMR spectrum of bisdethiobis (methylthio) gliotoxin (**19**) in $CDCl_3$ (400MHz)...S95
Figure S80. ^{13}C NMR spectrum of bisdethiobis(methylthio)gliotoxin (**19**) in $CDCl_3$ (100MHz)...S96
Figure S81. ^{1}H NMR spectrum of 6-acetylbis(methylthio) gliotoxin (**20**) in $CDCl_3$ (400MHz)...S97
Figure S82. ^{13}C NMR spectrum of 6-acetylbis (methylthio) gliotoxin (**20**) in $CDCl_3$ (100MHz)...S98
Figure S83. ^{1}H NMR spectrum of haematocin (**21**) in $CDCl_3$ (400MHz)...........................S99
Figure S84. ^{13}C NMR spectrum of haematocin (**21**) in $CDCl_3$ (100MHz)..........................S100
Figure S85. ^{1}H NMR spectrum of pityriacitrin (**22**) in Acetone-d_6 (400MHz).........................S101

Figure S86. ^{13}C NMR spectrum of pityriacitrin (**22**) in Acetone-d_6 (100MHz)........................S102
Figure S87. ^{1}H NMR spectrum of stellarine A (**23**) in DMSO-d_6 (400MHz)............................S103
Figure S88. ^{13}C NMR spectrum of stellarine A (**23**) in DMSO-d_6 (100MHz)...........................S104
Figure S89. ^{1}H NMR spectrum of perlolyrine (**24**) in $CDCl_3$ (400MHz).............................S105
Figure S90 ^{13}C NMR spectrum of perlolyrine (**24**) in $CDCl_3$ (100MHz)............................S106
Figure S91. ^{1}H NMR spectrum of fiscalin C (**25**) in $CDCl_3$ (400MHz)..............................S107
Figure S92. ^{13}C NMR spectrum of fiscalin C (**25**) in $CDCl_3$ (100MHz)............................S108
Figure S93. ^{1}H NMR spectrum of epi-fiscalin C (**26**) in $CDCl_3$ (400MHz).............................S109
Figure S94. ^{13}C NMR spectrum of epi-fiscalin C (**26**) in $CDCl_3$ (100MHz)............................S110
Figure S95. ^{1}H NMR spectrum of indolyl-3-acetic acid methyl ester (**27**) in $CDCl_3$ (400MHz)..S111
Figure S96. ^{13}C NMR spectrum of indolyl-3-acetic acid methyl ester (**27**) in $CDCl_3$ (100MHz)……………………………………………………………………………………S112
Figure S97. ^{1}H NMR spectrum of anthranilic acid (**28**) in Acetone-d_6 (400MHz)…..................S113
Figure S98. ^{13}C NMR spectrum of anthranilic acid (**28**) in Acetone-d_6 (100MHz)...................S114

Table S1. Comparison of the experimental optical rotatation values with the calculated OR values of compounds **1–5** and **16**

compounds	calculated OR values	experimental OR value
9*S*-**1**	-42.1	-41.9
9*R*-**1**	45.4	
9*S*,11*R*-**2**	-7.1	-4.4
9*R*,11*S*-**2**	7.4	
9*S*-**3**	-48.4	-51.6
9*R*-**3**	45.4	
9*S*-**4**	-14.5	-10.6
9*R*-**4**	14.6	
1*R*,2*R*,10*R*,15*S*-**5**	25.1	23.0
1*S*,2*S*,10*S*,15*R*-**5**	-24.5	
1*R*,2*S*,10*R*,15*S*-**5**	132.1	
1*S*,2*R*,10*S*,15*R*-**5**	-132.5	
1*S*,2*R*,10*R*,15*S*-**5**	52.9	
1*R*,2*S*,10*S*,15*R*-**5**	-50.0	
1*S*,2*S*,10*R*,15*S*-**5**	122.3	
1*R*,2*R*,10*S*,15*R*-**5**	-122.4	
3*R*,6*S*,7*S*,11*S*,13*R*-**16**	-59.6	-60.5
3*S*,6*R*,7*R*,11*R*,13*S*-**16**	56.3	
3*R*,6*R*,7*R*,11*R*,13*R*-**16**	133.3	
3*S*,6*S*,7*S*,11*S*,13*S*-**16**	160.8	
3*R*,6*S*,7*S*,11*R*,13*R*-**16**	-120.3	
3*S*,6*R*,7*R*,11*S*,13*S*-**16**	119.0	
3*R*,6*R*,7*R*,11*S*,13*R*-**16**	-78.2	
3*S*,6*S*,7*S*,11*R*,13*S*-**16**	78.6	

Figure S1. The most stable conformers of **1–5**, **16** calculated at the B3LYP/6-31+G(d) level. Relative populations are in parentheses.

(9*S*)-1

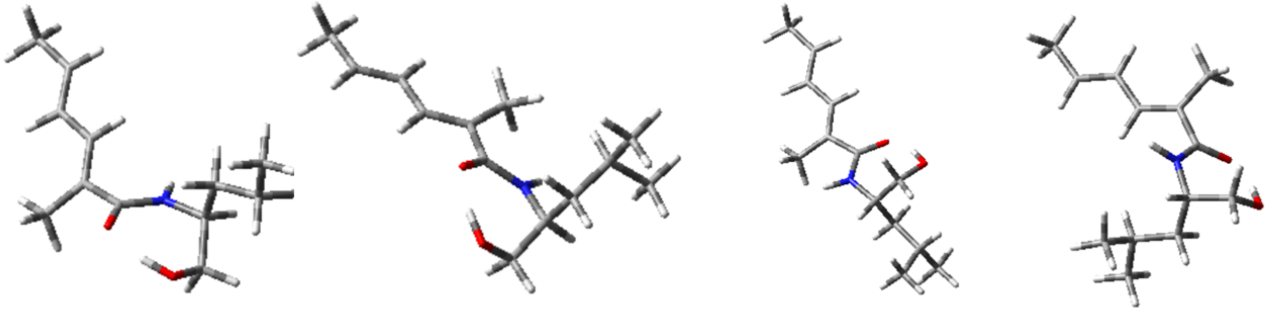

1（56.82%）　2（22.18%）　3（15.01%）　4（5.98%）

(9*R*)-1

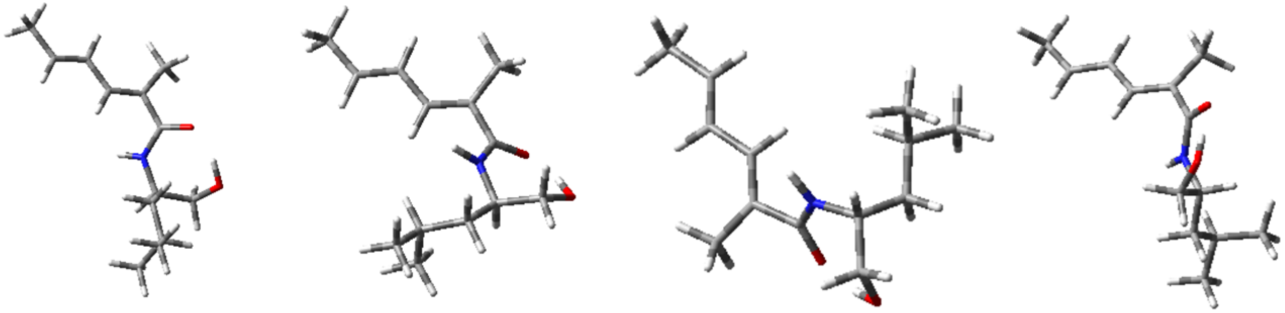

1（66.47%）　2（17.03%）　3（12.52%）　4（5.95%）

(9*S*,11*R*)**-2**

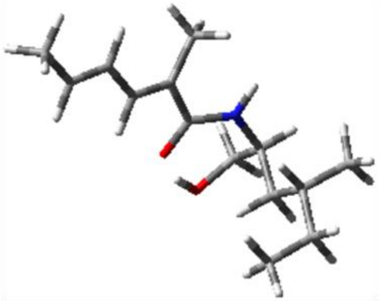

1（42.8%）

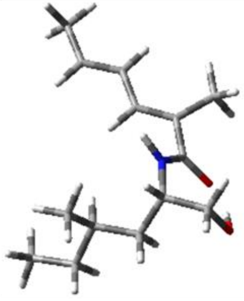

2（28.83%）

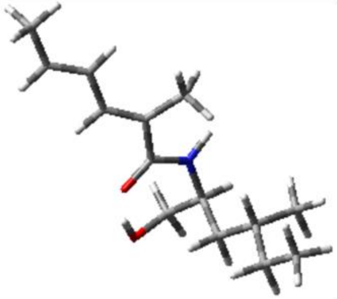

3（15.59%）

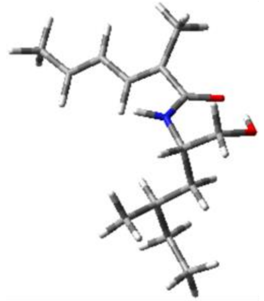

4（12.77%）

(9*R*,11*S*)**-2**

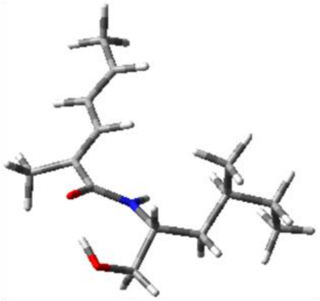

1（40.05%）

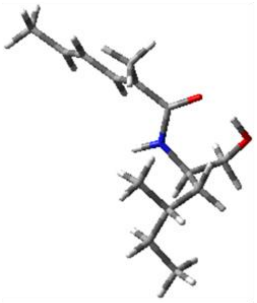

2（28.57%）

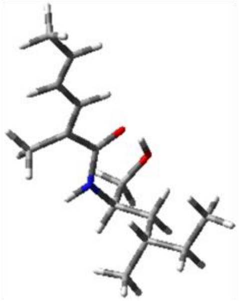

3（16.08%）

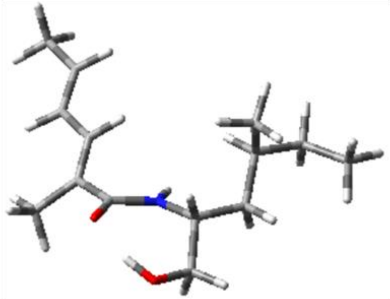

4（15.29%）

(9*S*)-**3**

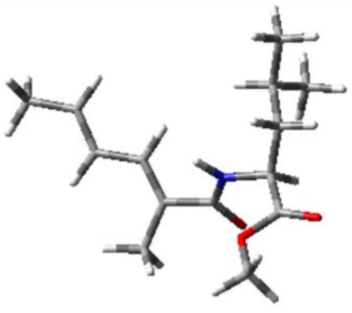

1（55.04%）

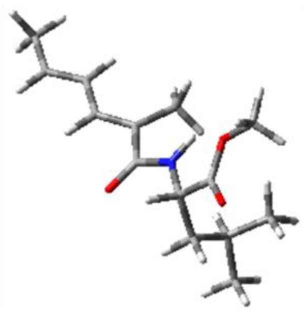

2（23.36%）

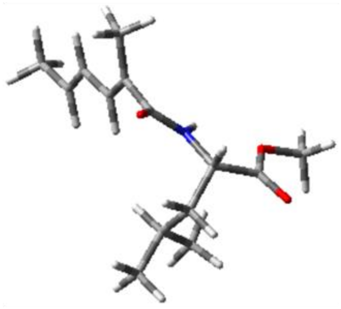

3（11.11%）

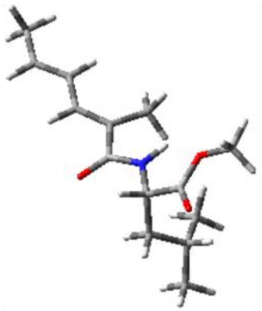

4（10.48%）

(9*R*)-**3**

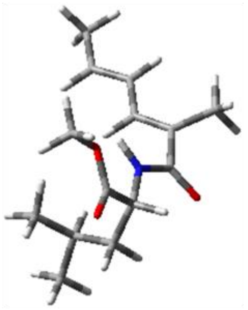

1（50.52%）

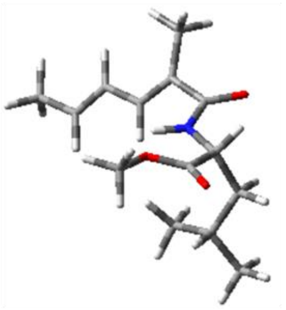

2（25.52%）

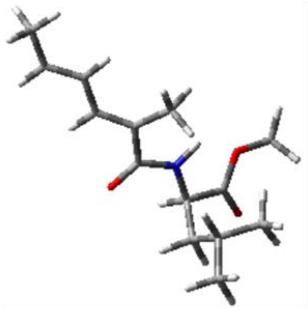

3（13.65%）

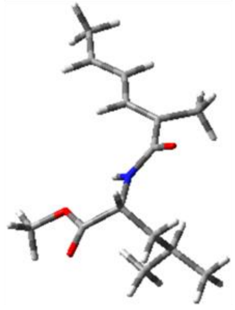

4（10.3%）

(9*S*)-**4**

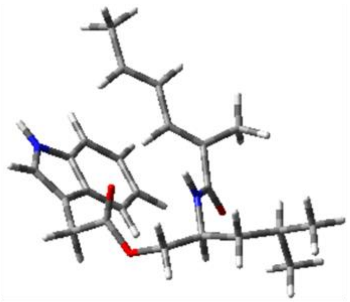

1（31.68%）

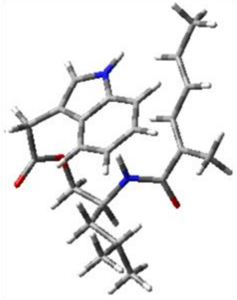

2（25.87%）

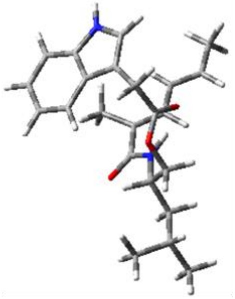

3（25.14%）

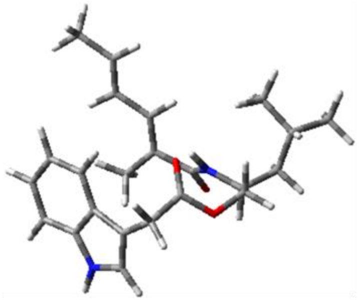

4（17.3%）

(9*R*)-**4**

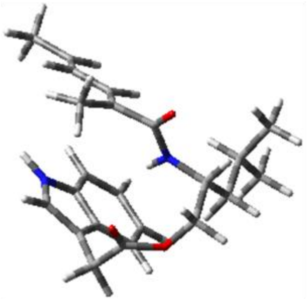

1（30.16%）

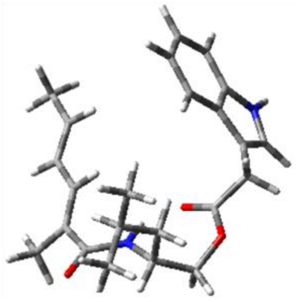

2（24.42%）

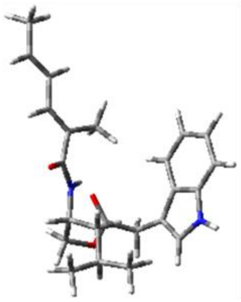

3（23.77%）

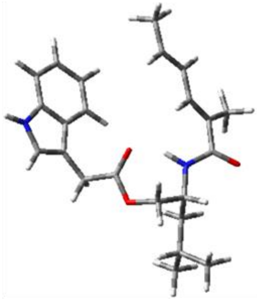

4（21.63%）

(1*R*,2*R*,10*R*,15*S*)-**5**

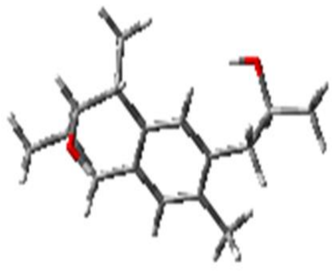
1(63%)

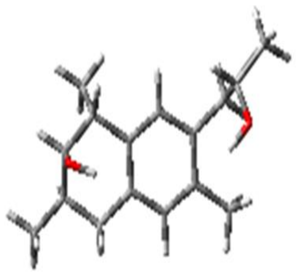
2(24.1%)

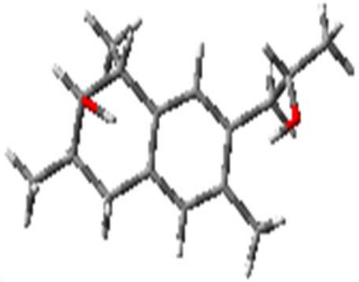
3(4.23%)

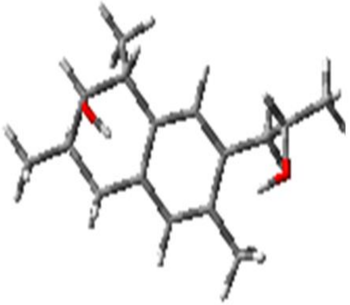
4(4.01%)

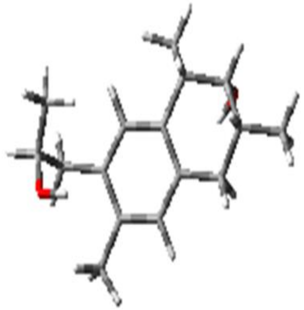
5(2.21%)

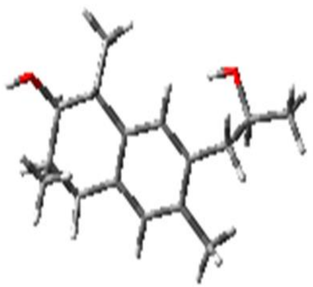
6(1.67%)

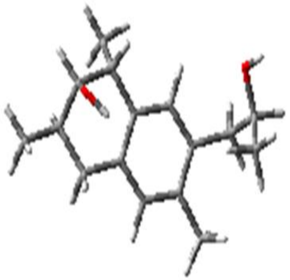
7(0.31%)

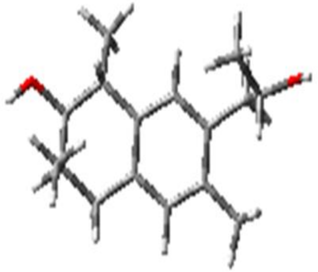
8(0.2%)

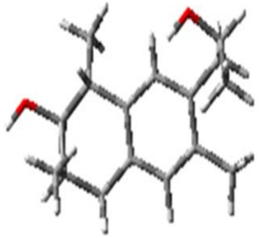
9(0.17%)

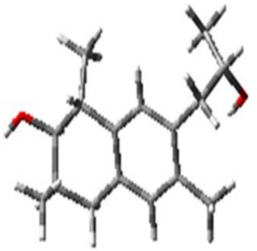
10(0.1%)

(1*S*,2*S*,10*S*,15*R*)-**5**

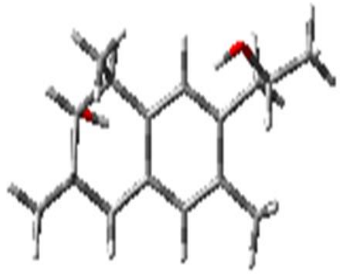

1(64.21%)

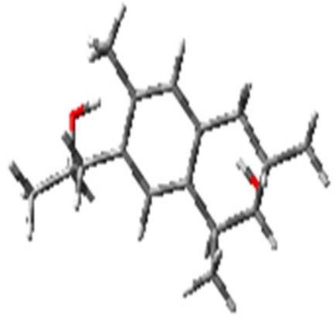

2(22.9%)

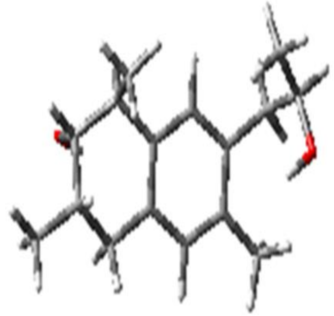

3(3.03%)

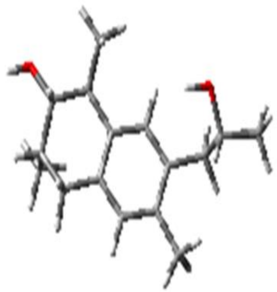

4(2.81%)

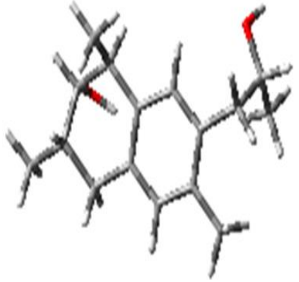

5(3.32%)

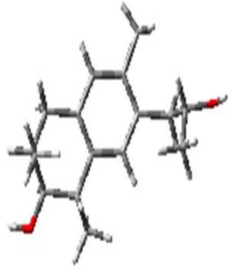

6(2.91%)

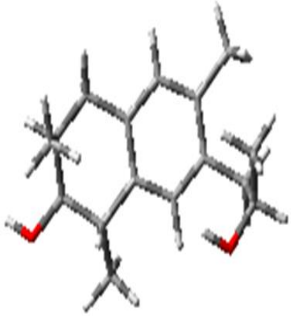

7(0.45%)

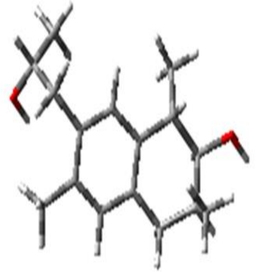

8(0.17%)

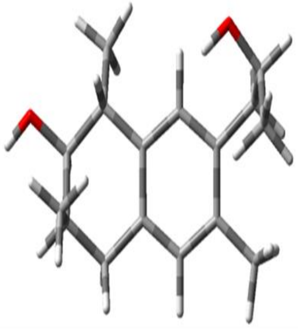

9(0.1%)

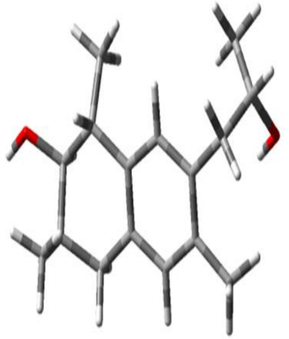

10(0.1%)

(1*R*,2*S*,10*R*,15*S*)-**5**

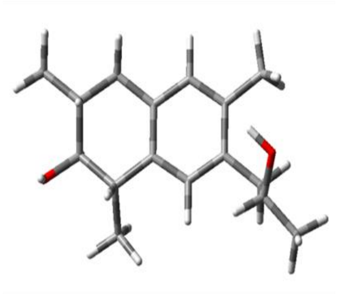

1(73.17%)

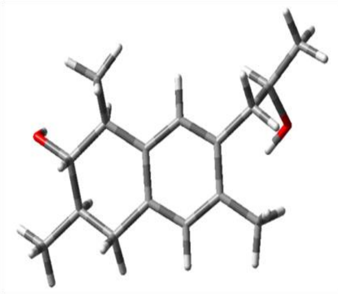

2(10.34%)

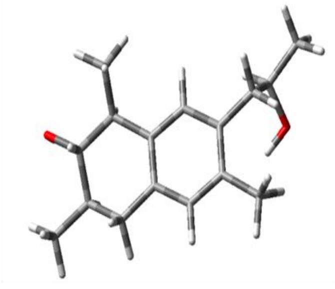

3(9.3%)

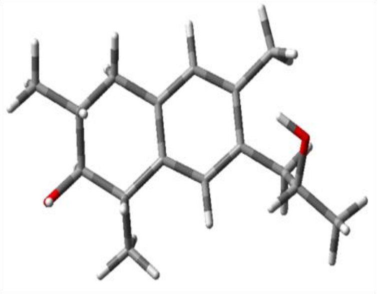

4(5.47%)

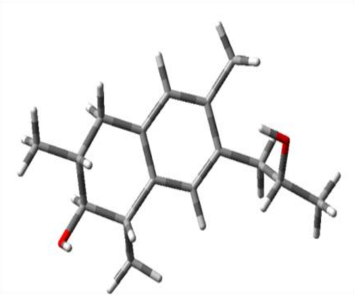

5(1.4%)

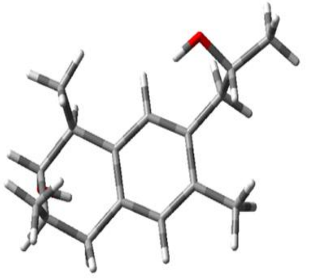

6(0.13%)

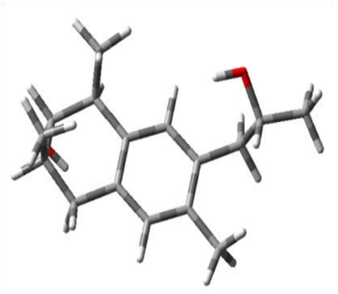

7(0.11%)

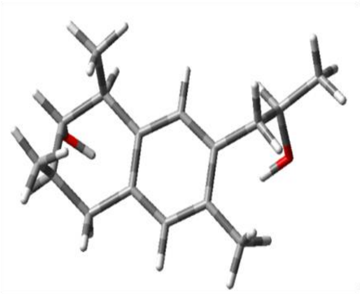

8(0.06%)

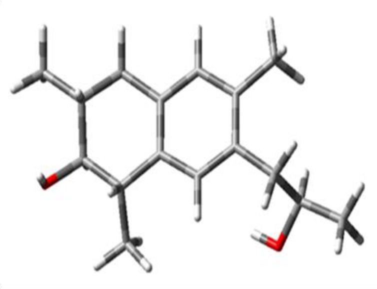

9(0.01%)

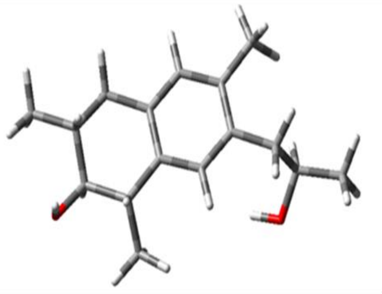

10(0.01%)

(1*S*,2*R*,10*S*,15*R*)-**5**

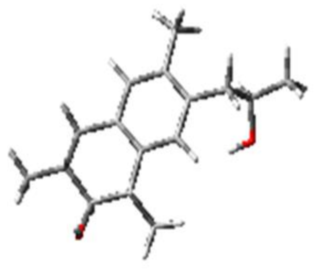

1(71%)

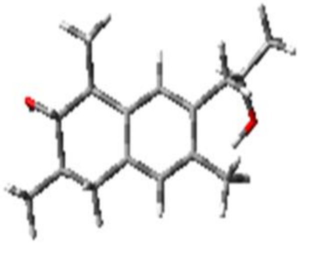

2(9.31%)

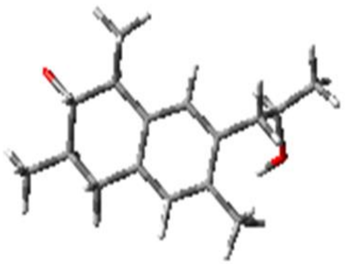

3(8.02%)

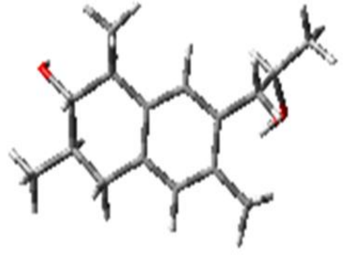

4(7.6%)

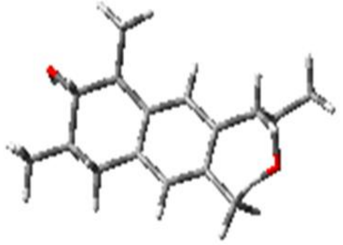

5(3.43%)

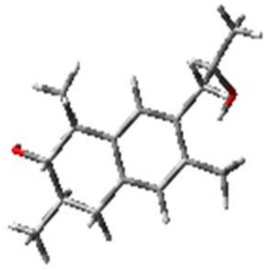

6(0.35%)

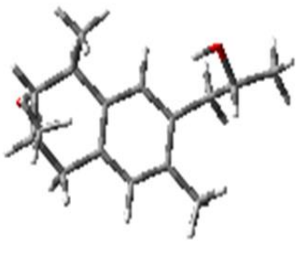

7(0.14%)

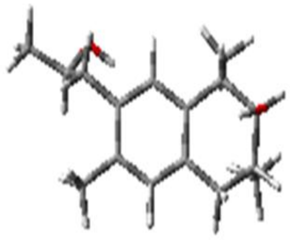

8(0.1%)

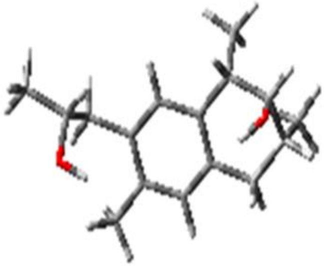

9(0.04%)

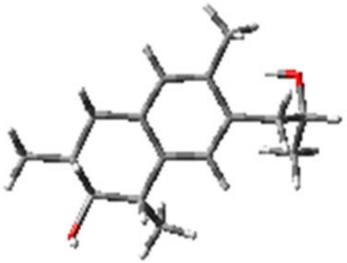

10(0.01%)

(1*S*,2*R*,10*R*,15*S*)-**5**

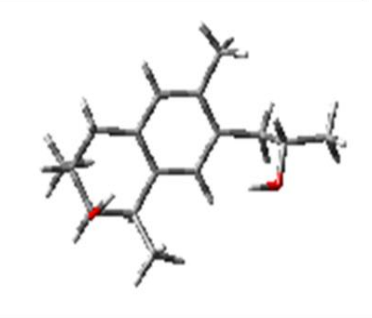
1(50.65%)

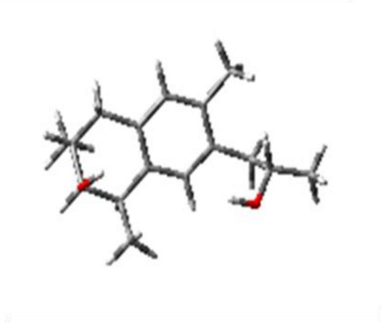
2(41.39%)

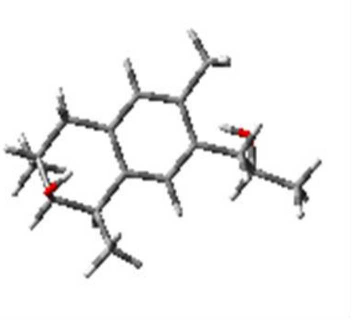
3(4%)

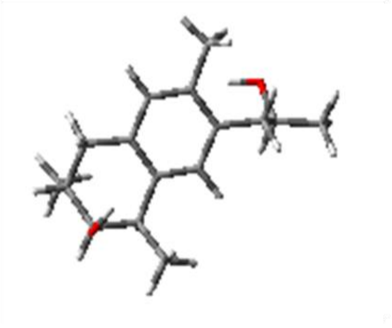
4(1.14%)

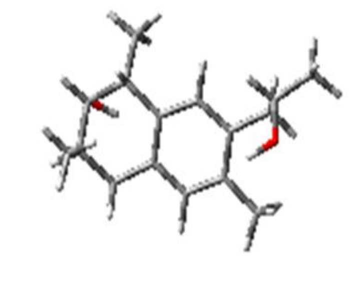
5(0.93%)

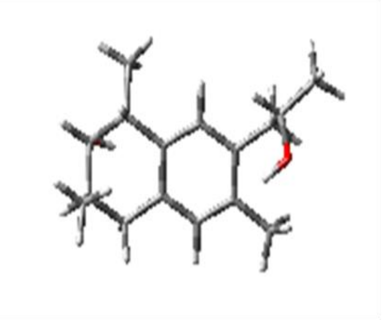
6(0.84%)

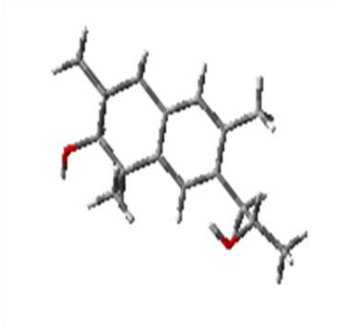
7(0.41%)

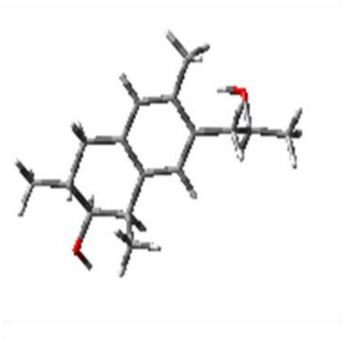
8(0.29%)

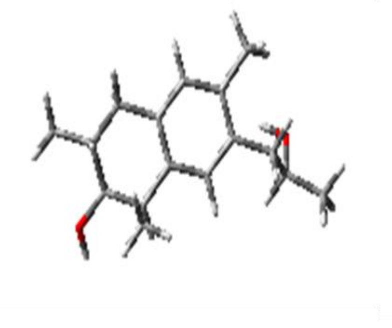
9(0.25%)

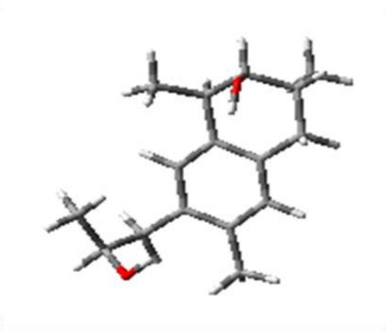
10(0.01%)

(1*R*,2*S*,10*S*,15*R*)-**5**

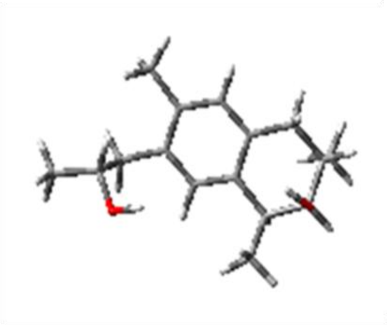

1(75.37%)

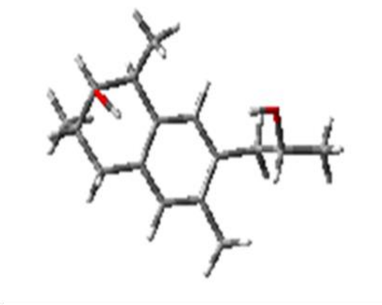

2(13.2%)

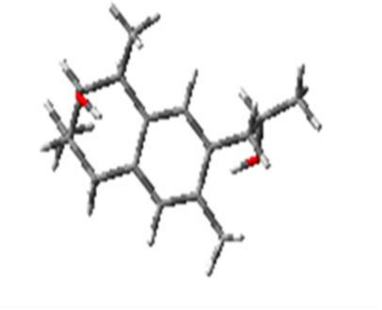

3(7.64%)

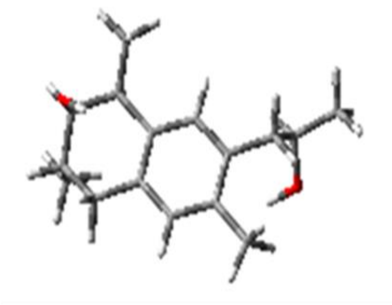

4(2.68%)

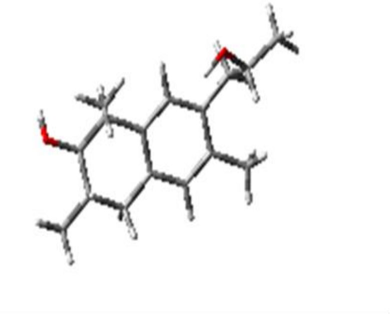

5(0.58%)

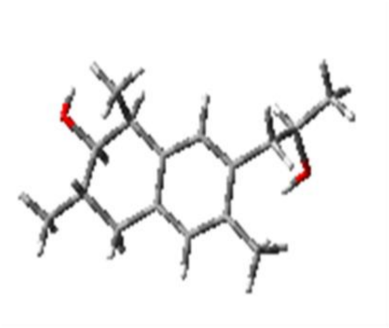

6(0.27%)

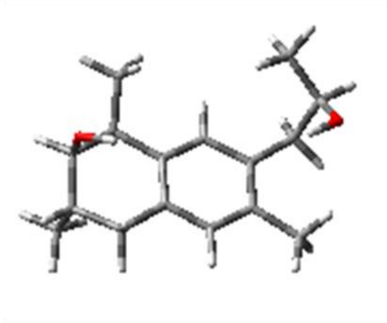

7(0.16%)

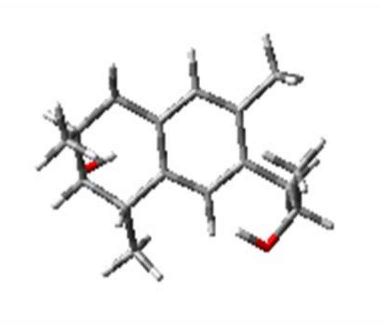

8(0.08%)

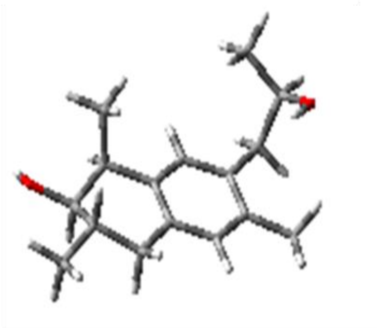

9(0.01%)

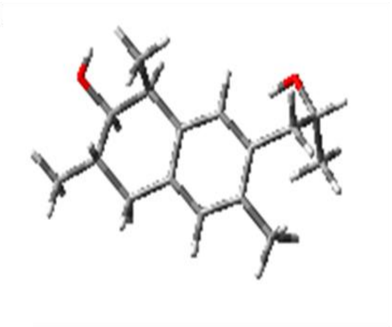

10(0.01%)

(1*S*,2*S*,10*R*,15*S*)-**5**

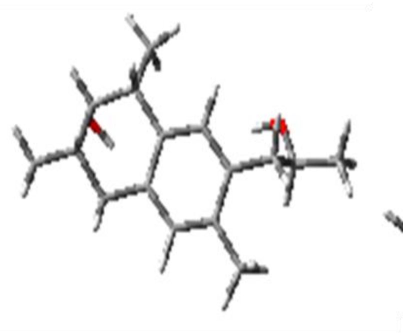

1(59.46%)

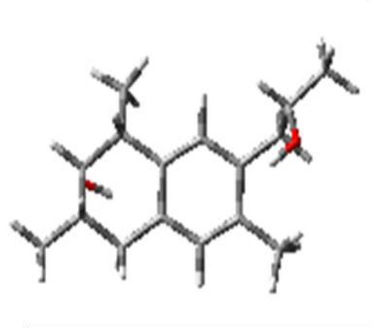

2(26.21%)

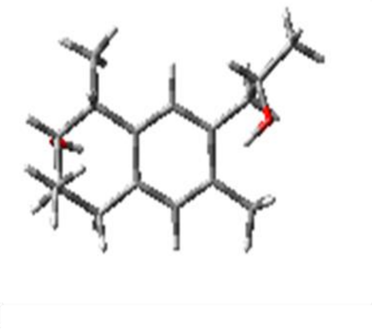

3(3.96%)

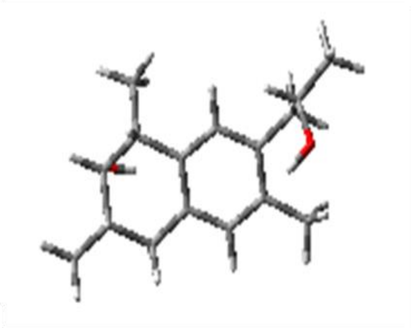

4(2.44%)

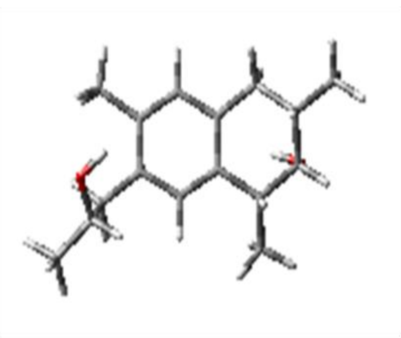

5(4.38%)

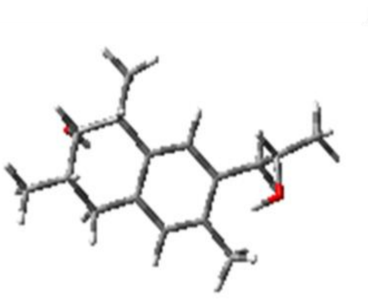

6(2.71%)

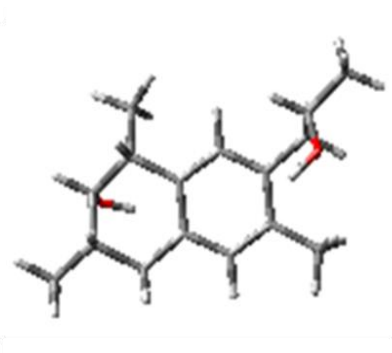

7(0.43%)

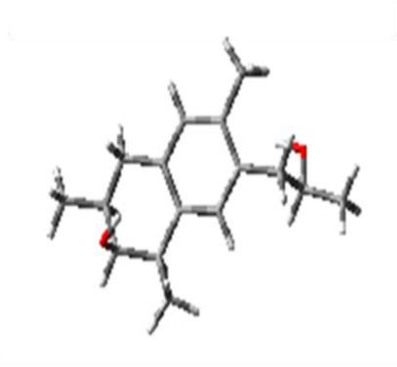

8(0.33%)

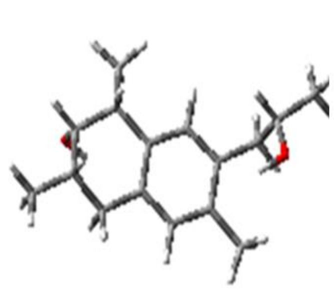

9(0.07%)

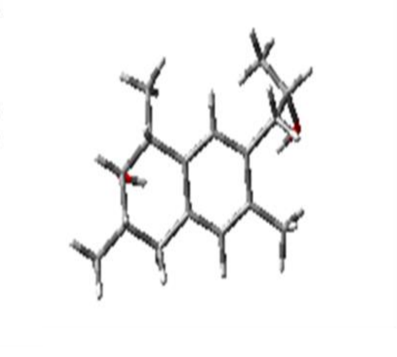

10(0.01%)

(1*R*,2*R*,10*S*,15*R*)-**5**

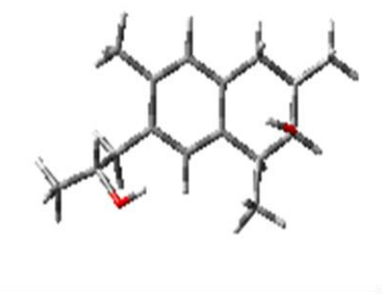

1(61.33%)

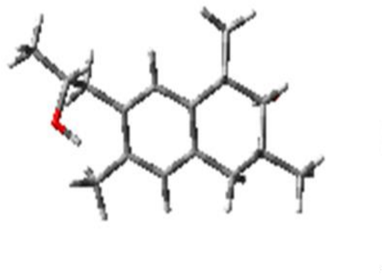

2(24.34%)

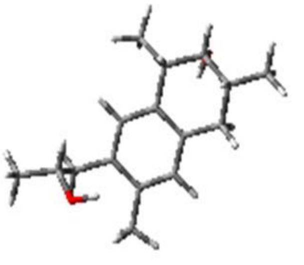

3(5.83%)

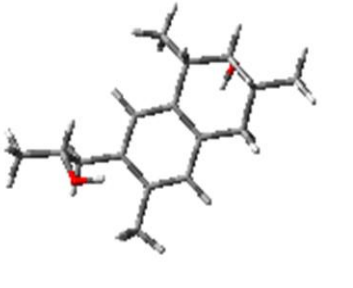

4(4.25%)

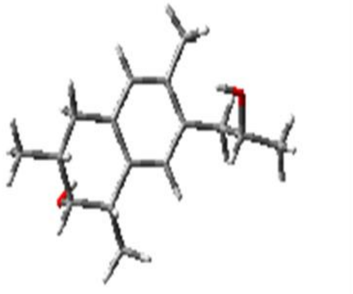

5(2.51%)

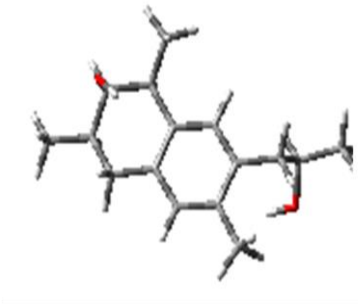

6(0.84%)

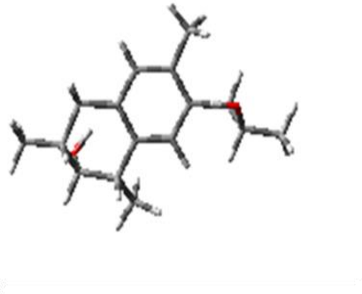

7(0.55%)

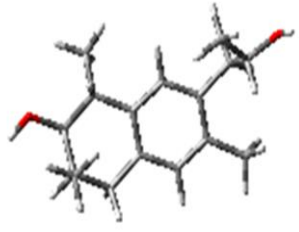

8(0.21%)

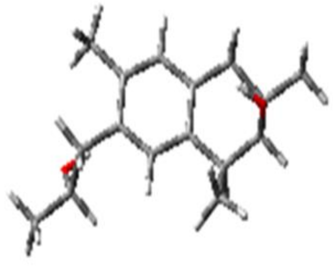

9(0.09%)

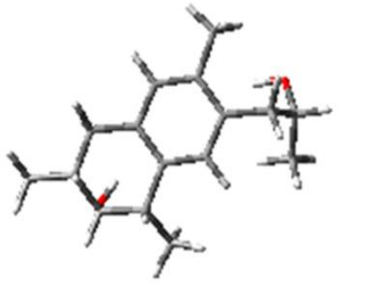

10(0.05%)

(3*R*,6*S*,7*S*,11*S*,13*R*)**-16**

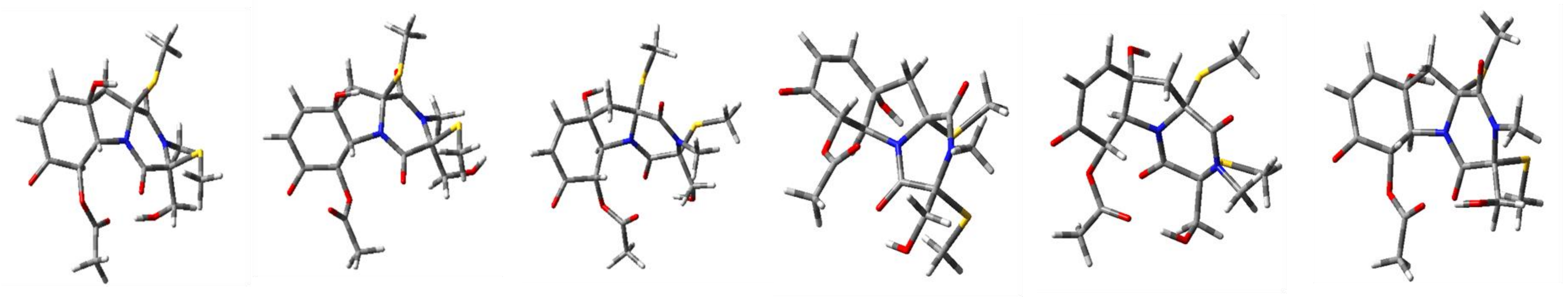

1(48.26%) 2(41.99%) 3(5.03%) 4(3.94%) 5(0.71%) 6(0.07%)

(3*S*,6*R*,7*R*,11*R*,13*S*)**-16**

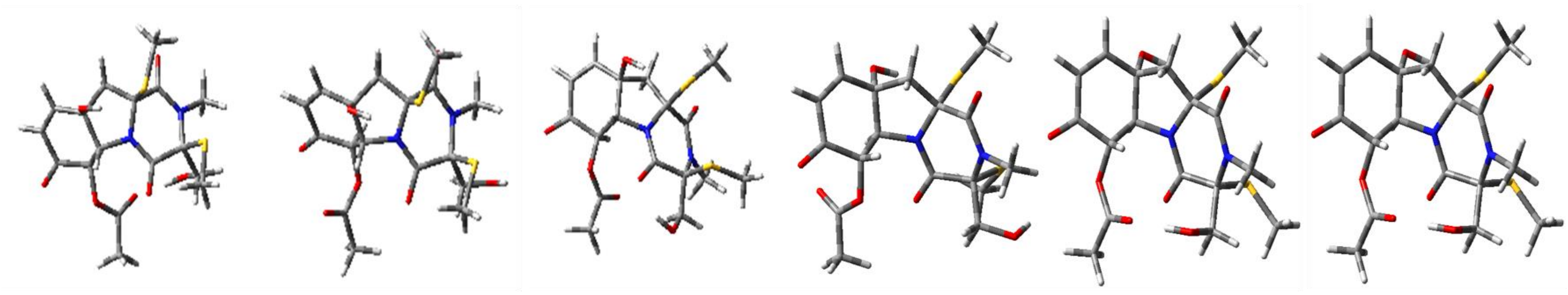

1(49.12%) 2(42.85%) 3(4.17%) 4(3.08 %) 5(0.57%) 6(0.21%)

(3*R*,6*R*,7*R*,11*R*,13*R*)**-16**

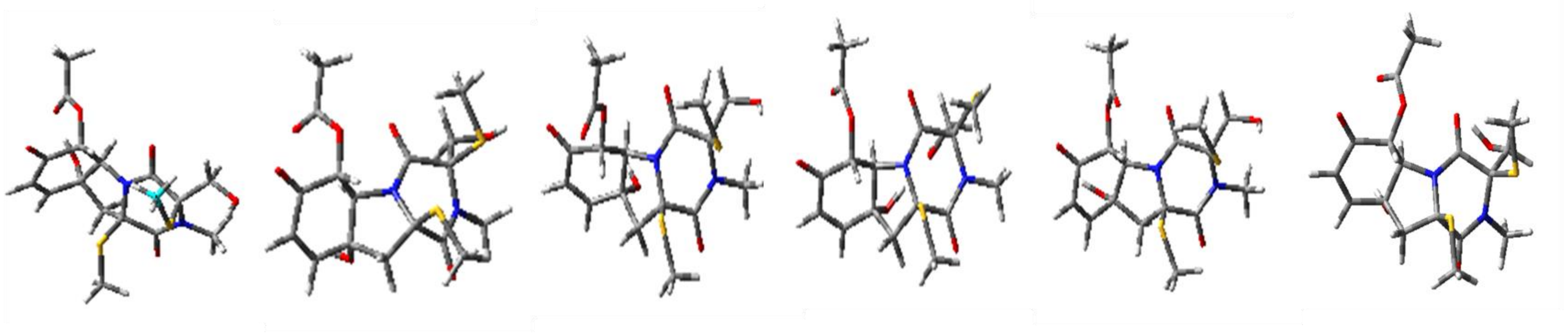

1(49.52%) 2(45.31%) 3(3.44%) 4(1.24%) 5(0.41%) 6(0.08%)

(3*S*,6*S*,7*S*,11*S*,13*S*)**-16**

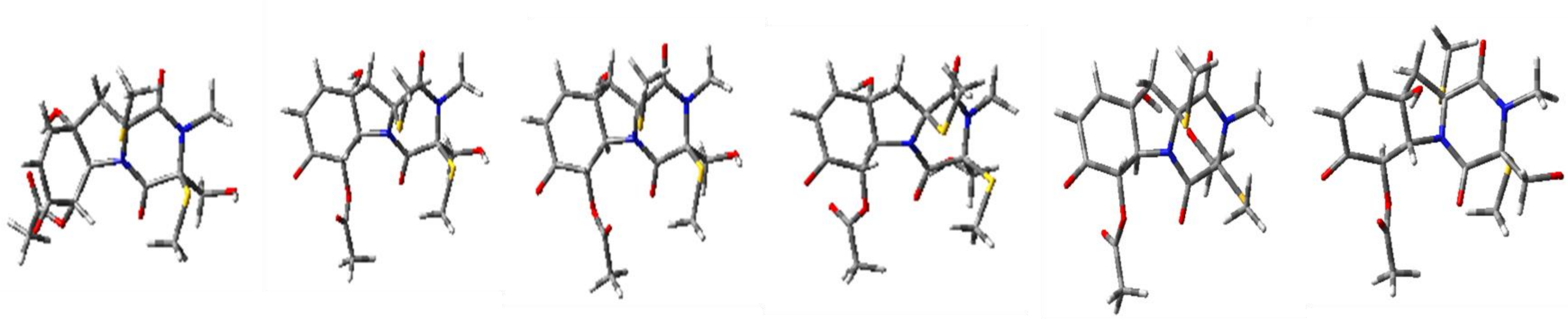

1(50.21%) 2(43.00%) 3(4.44%) 4(1.45%) 5(0.62%) 6(0.28%)

(3*R*,6*S*,7*S*,11*R*,13*R*)**-16**

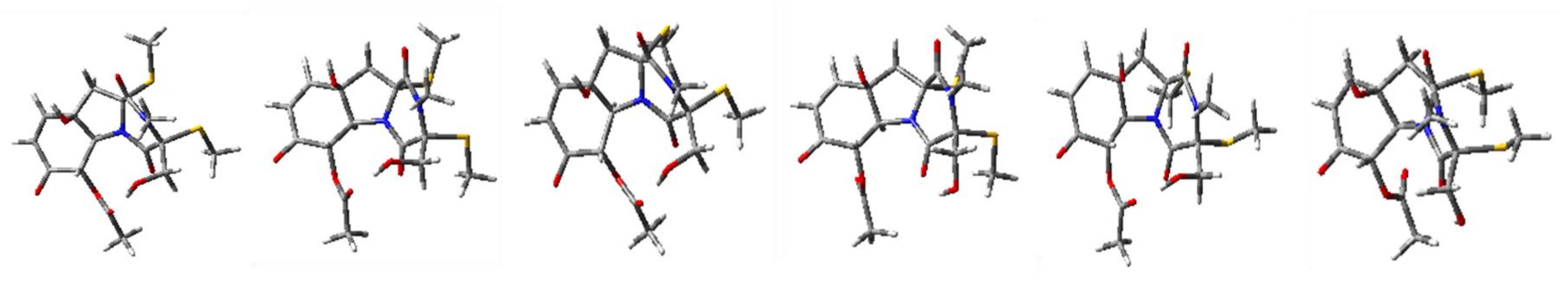

1(49.64%) 2(45.19%) 3(3.56%) 4(1.12%) 5(0.51%) 6(0.02%)

(3*S*,6*R*,7*R*,11*S*,13*S*)**-16**

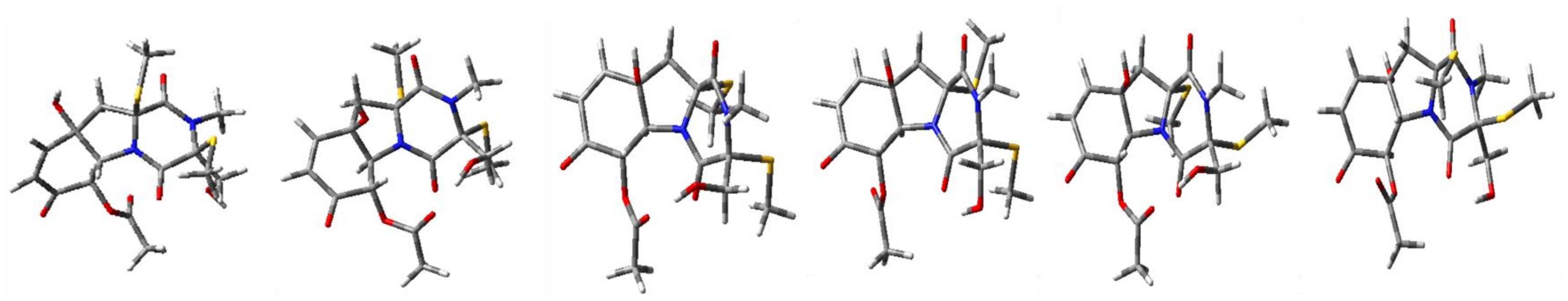

1(47.53%) 2(47.38%) 3(2.58%) 4(2.58%) 5(0.81%) 6(0.61%)

(3*R*,6*R*,7*R*,11*S*,13*R*)**-16**

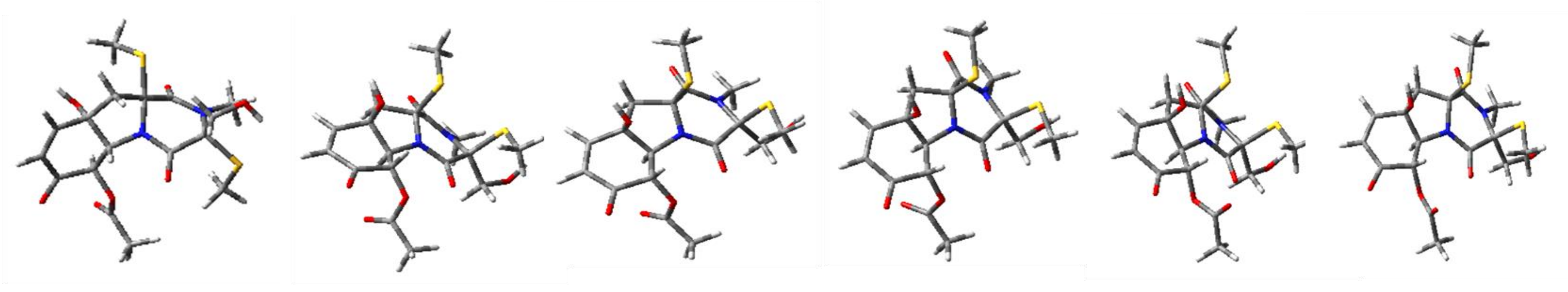

1(50.21%) 2(38.1%) 3(6.13%) 4(4%) 5(1.46%) 6(0.01%)

(3*S*,6*S*,7*S*,11*R*,13*S*)**-16**

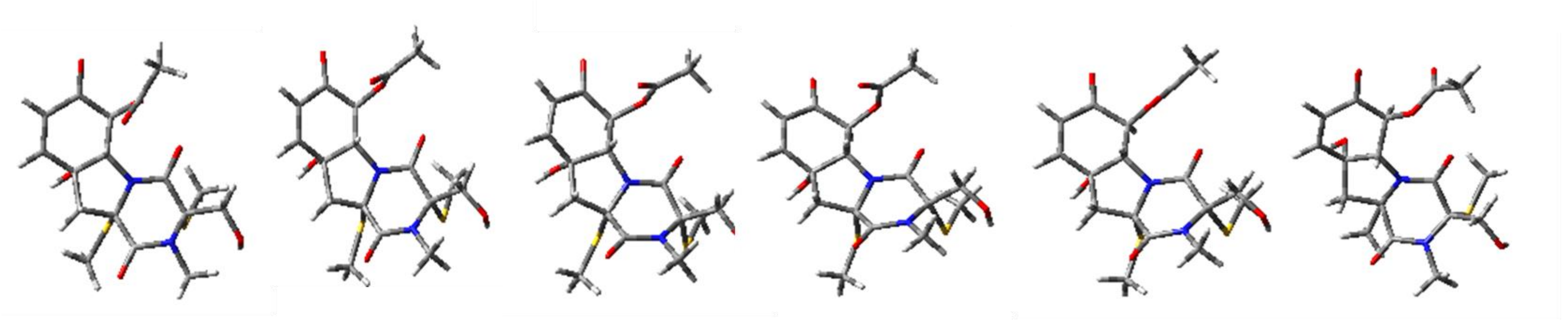

1(49.25%) 2(41%) 3(6.02%) 4(3.08%) 5(0.51%) 6(0.14%)

Figure S2. HR-ESI-MS spectrum of dichotomocej A (**1**)

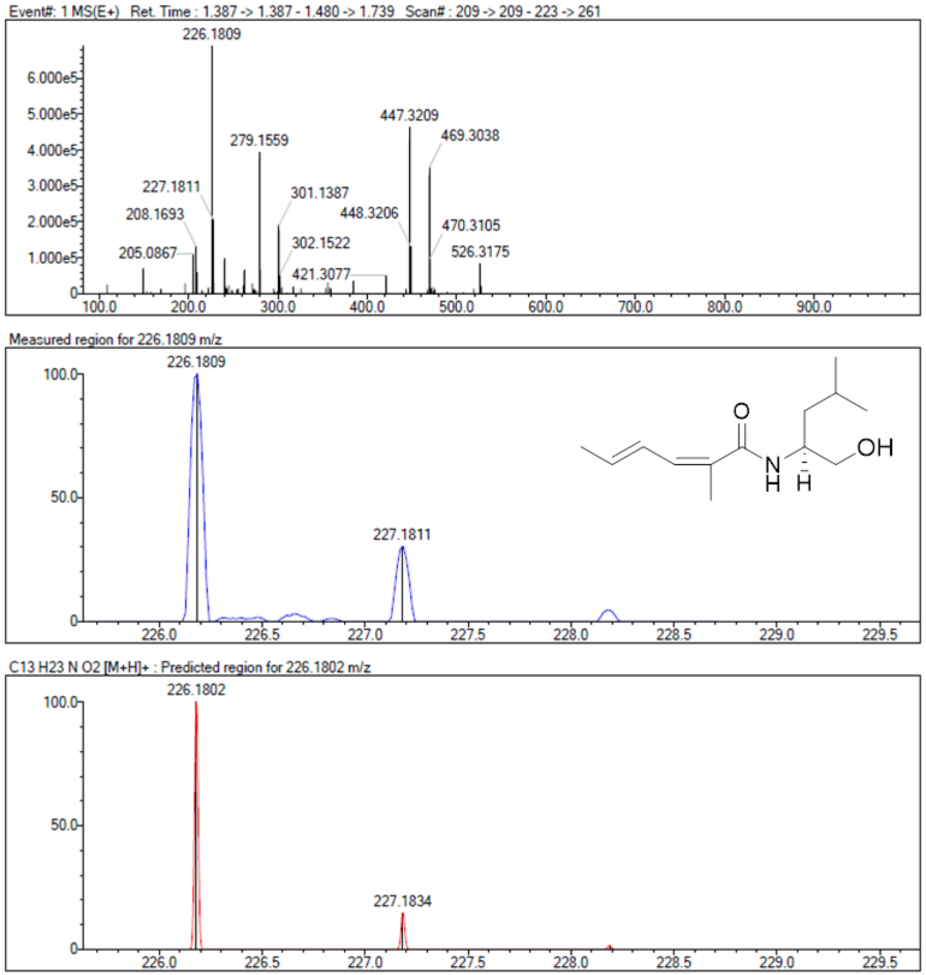

Rank	Score	Formula (M)	Ion	Meas. m/z	Pred. m/z	Df. (mDa)	Df. (ppm)	Iso	DBE
> 1	31.95	C13 H23 N O2	[M+H]+	226.1809	226.1802	0.7	3.09	33.72	3.0

Figure S3. ^{1}H NMR spectrum of dichotomocej A (**1**) in $CDCl_3$ (400MHz)

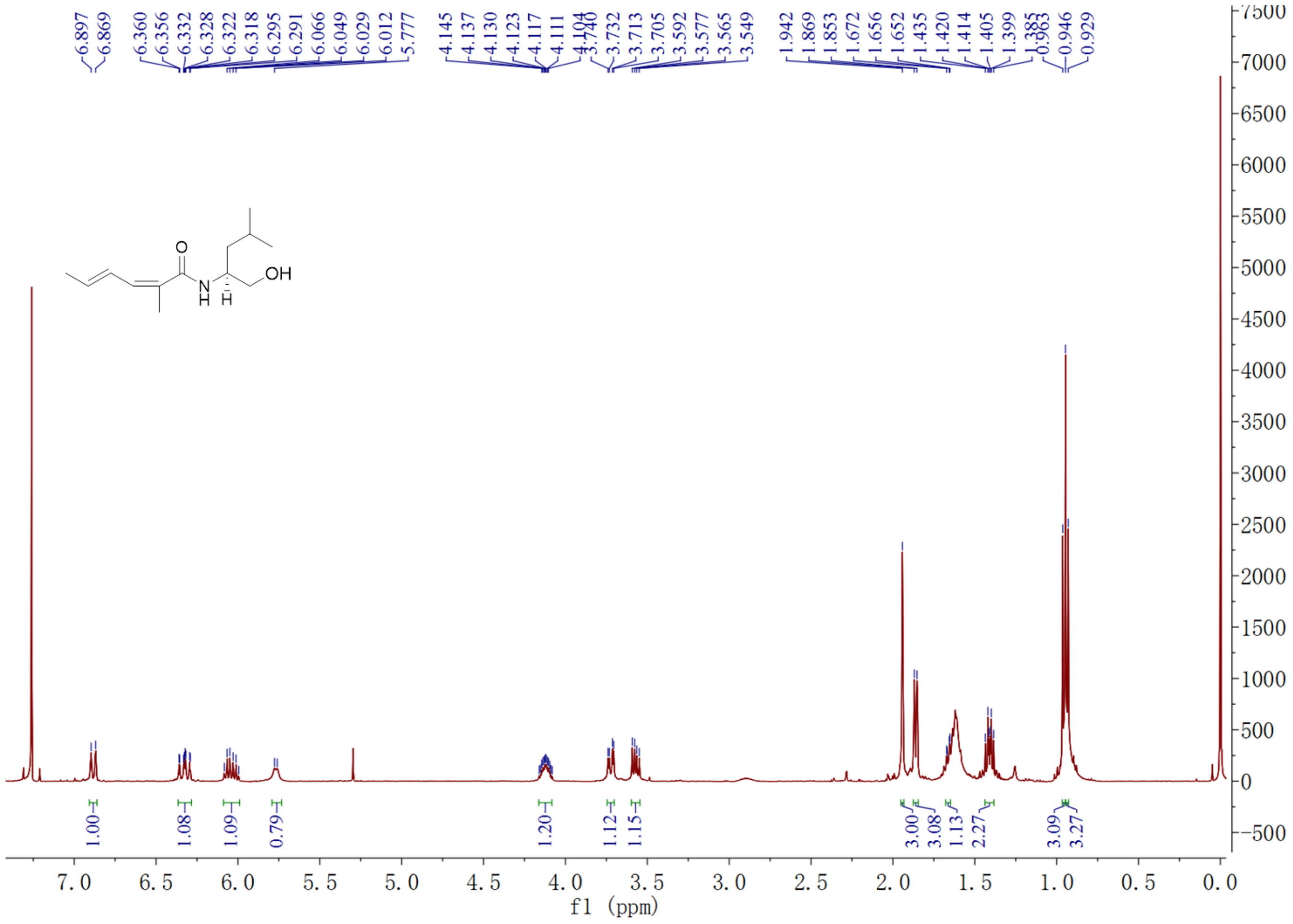

Figure S4. ^{13}C NMR spectrum of dichotomocej A (**1**) in $CDCl_3$ (100MHz)

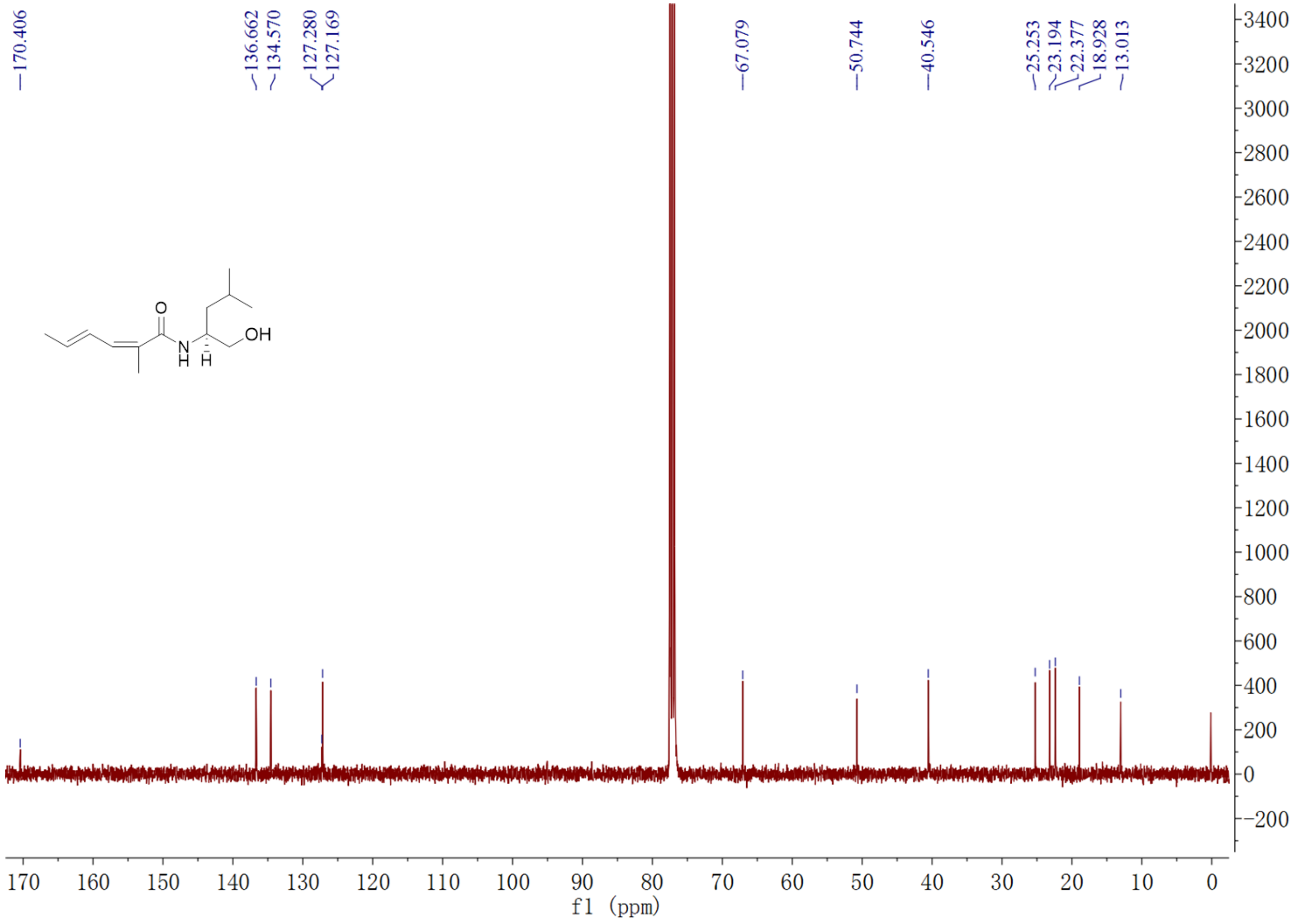

Figure S5. HMQC spectrum of dichotomocej A (**1**) in $CDCl_3$

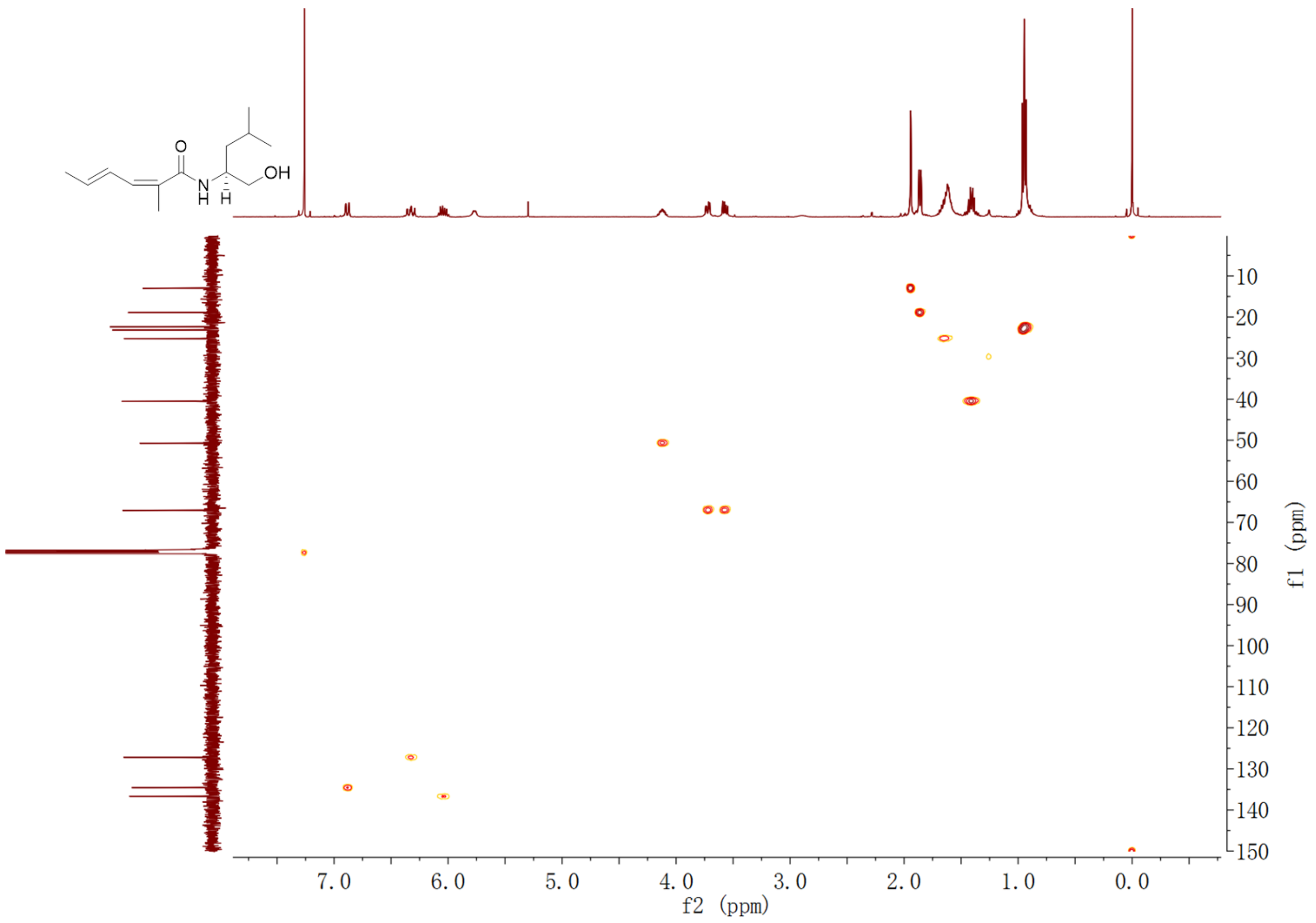

Figure S6. ^{1}H-^{1}H COSY spectrum of dichotomocej A (**1**) in $CDCl_3$

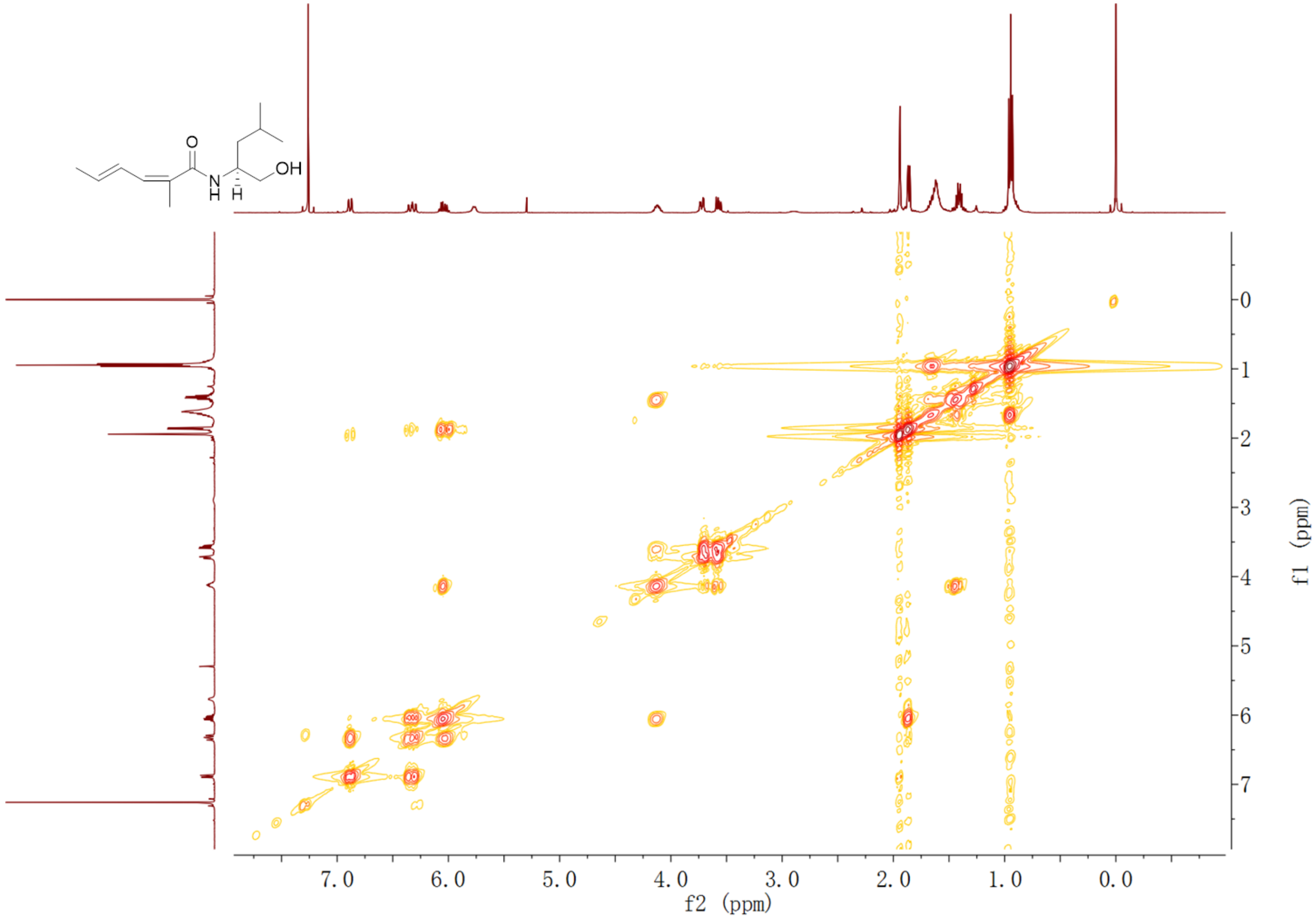

Figure S7. HMBC spectrum of dichotomocej A (**1**) in $CDCl_3$

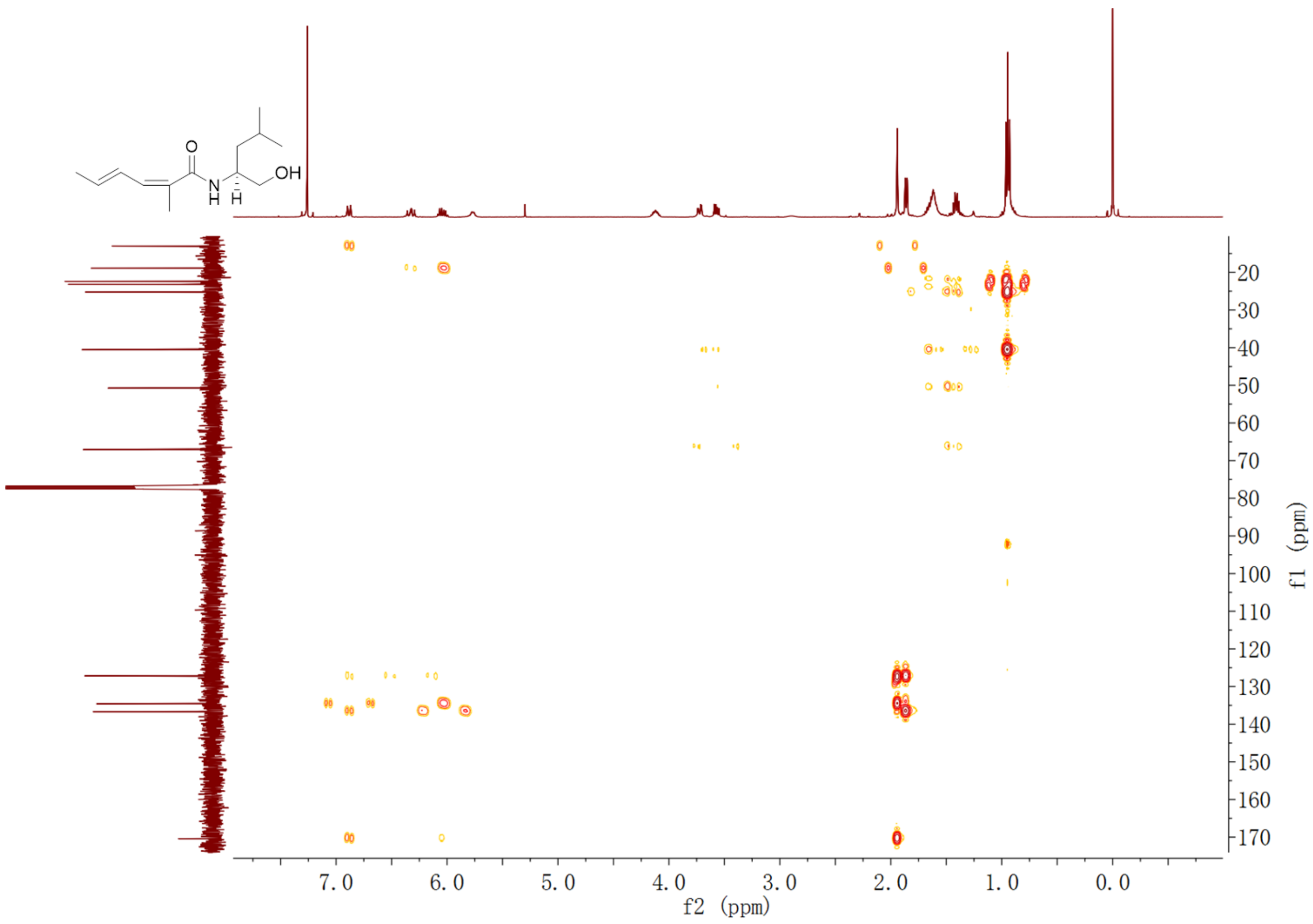

Figure S8. NOESY spectrum of dichotomocej A (**1**) in $CDCl_3$

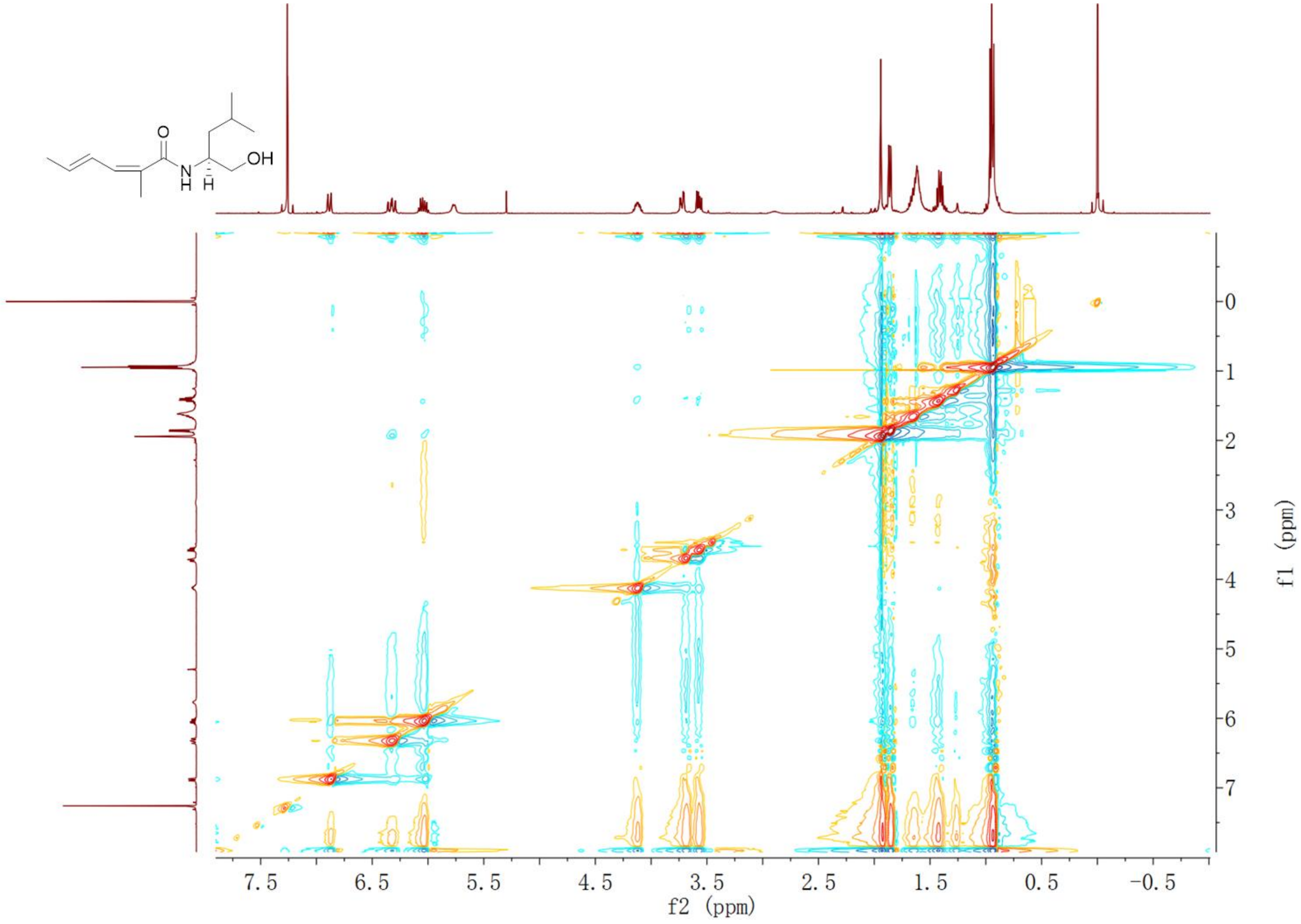

Figure S9. HR-ESI-MS spectrum of dichotomocej B (**2**)

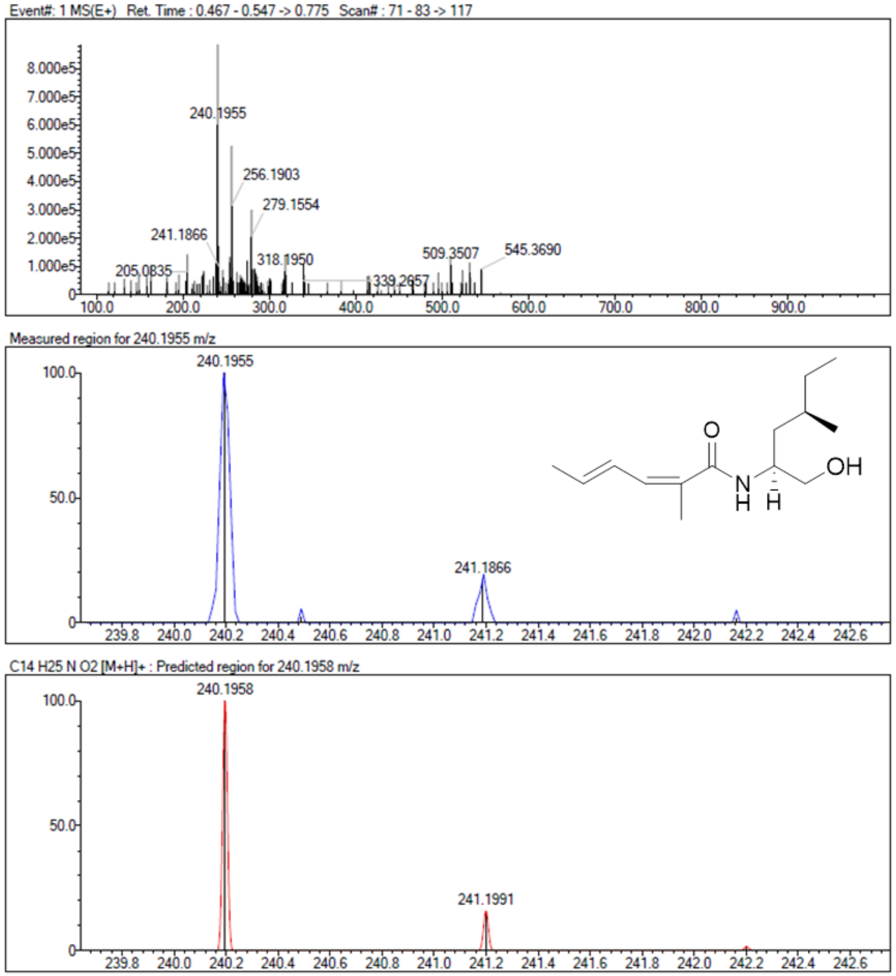

Rank	Score	Formula (M)	Ion	Meas. m/z	Pred. m/z	Df. (mDa)	Df. (ppm)	Iso	DBE
1	51.67	C14 H25 N O2	[M+H]+	240.1955	240.1958	-0.3	-1.25	51.99	3.0

Figure S10. ^{1}H NMR spectrum of dichotomocej B (**2**) in $CDCl_3$ (400MHz)

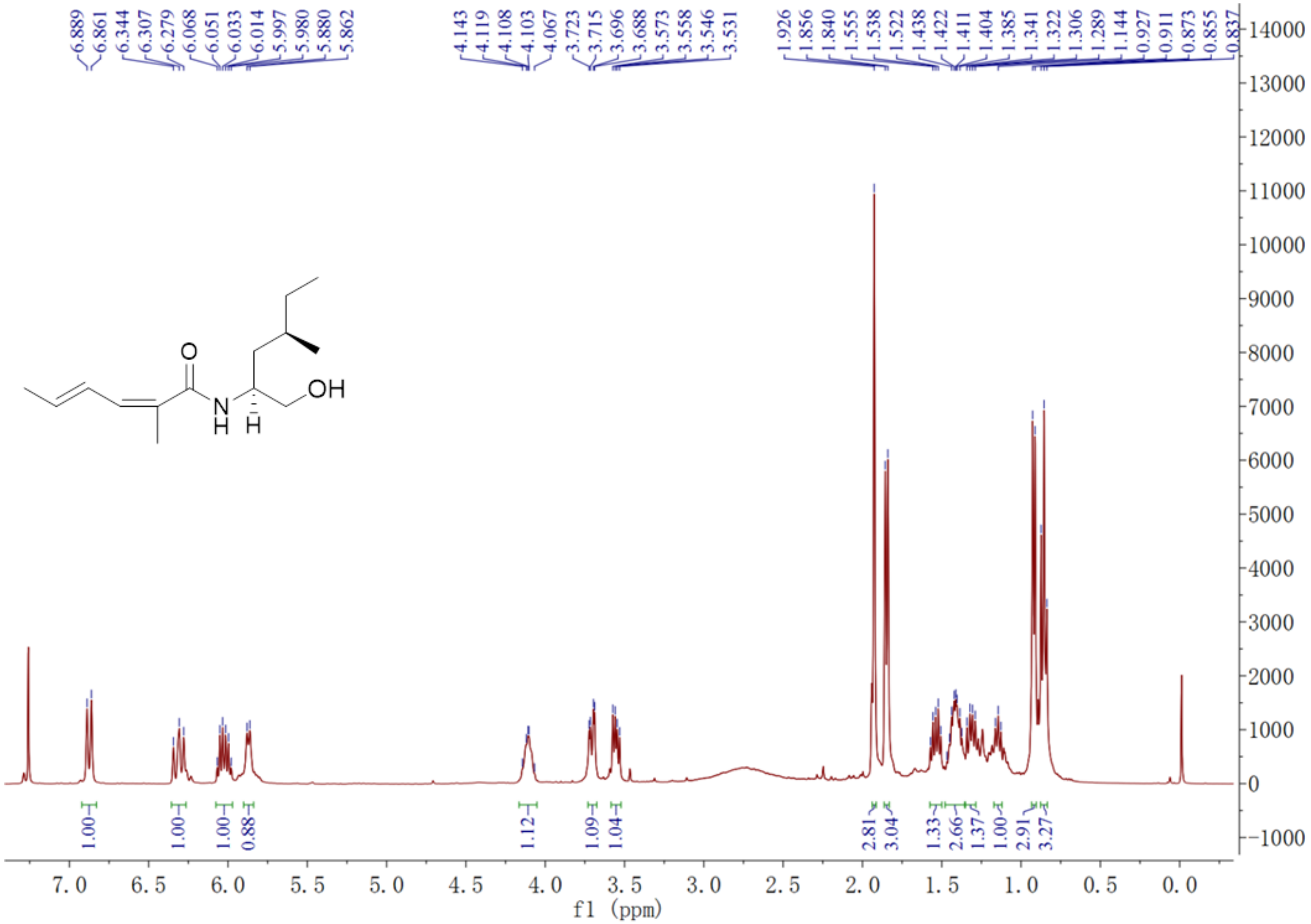

Figure S11. ^{13}C NMR spectrum of dichotomocej B (**2**) in $CDCl_3$ (100MHz)

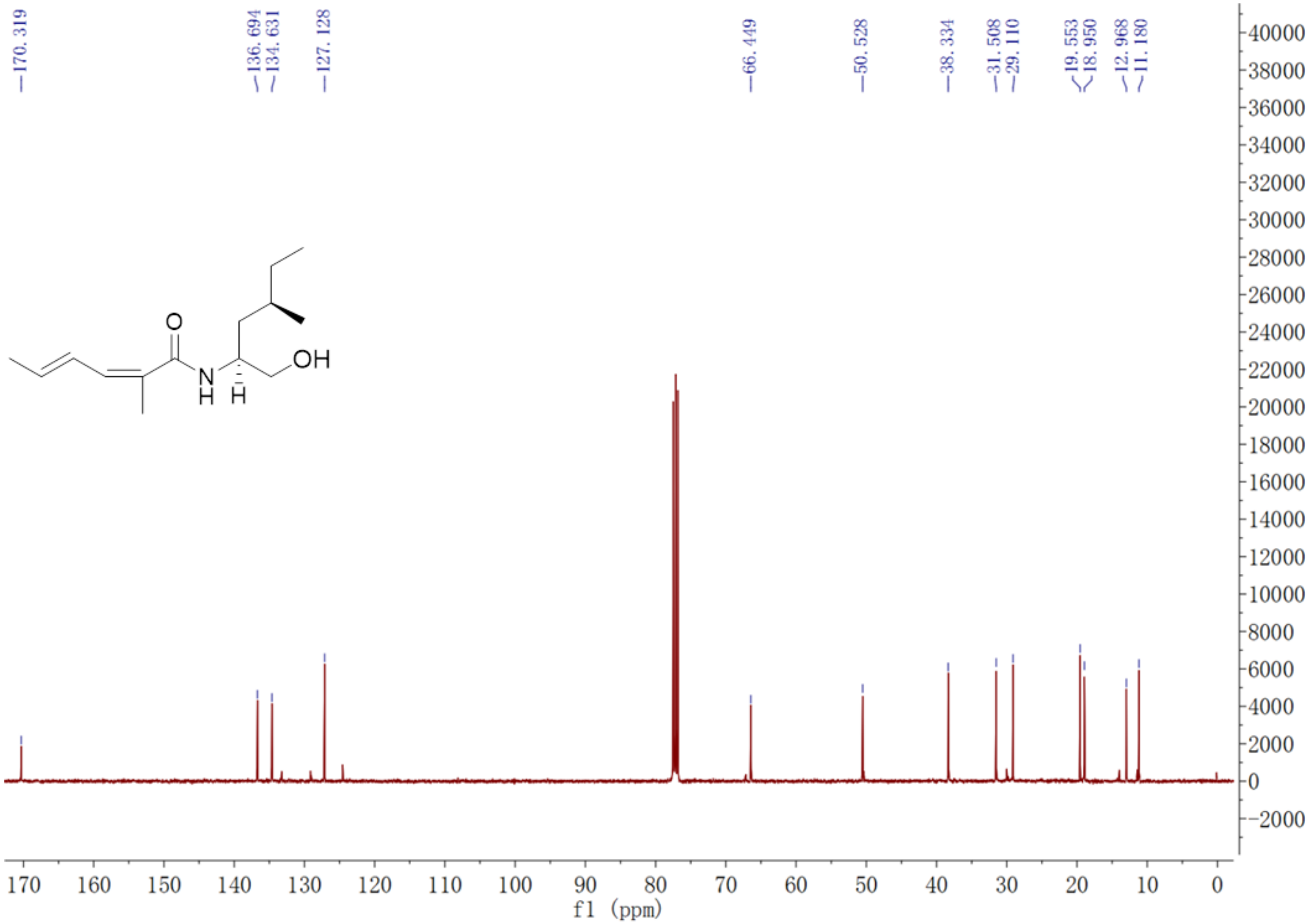

Figure S12. DEPT 135 spectum of dichotomocej B (**2**) in $CDCl_3$ (100MHz)

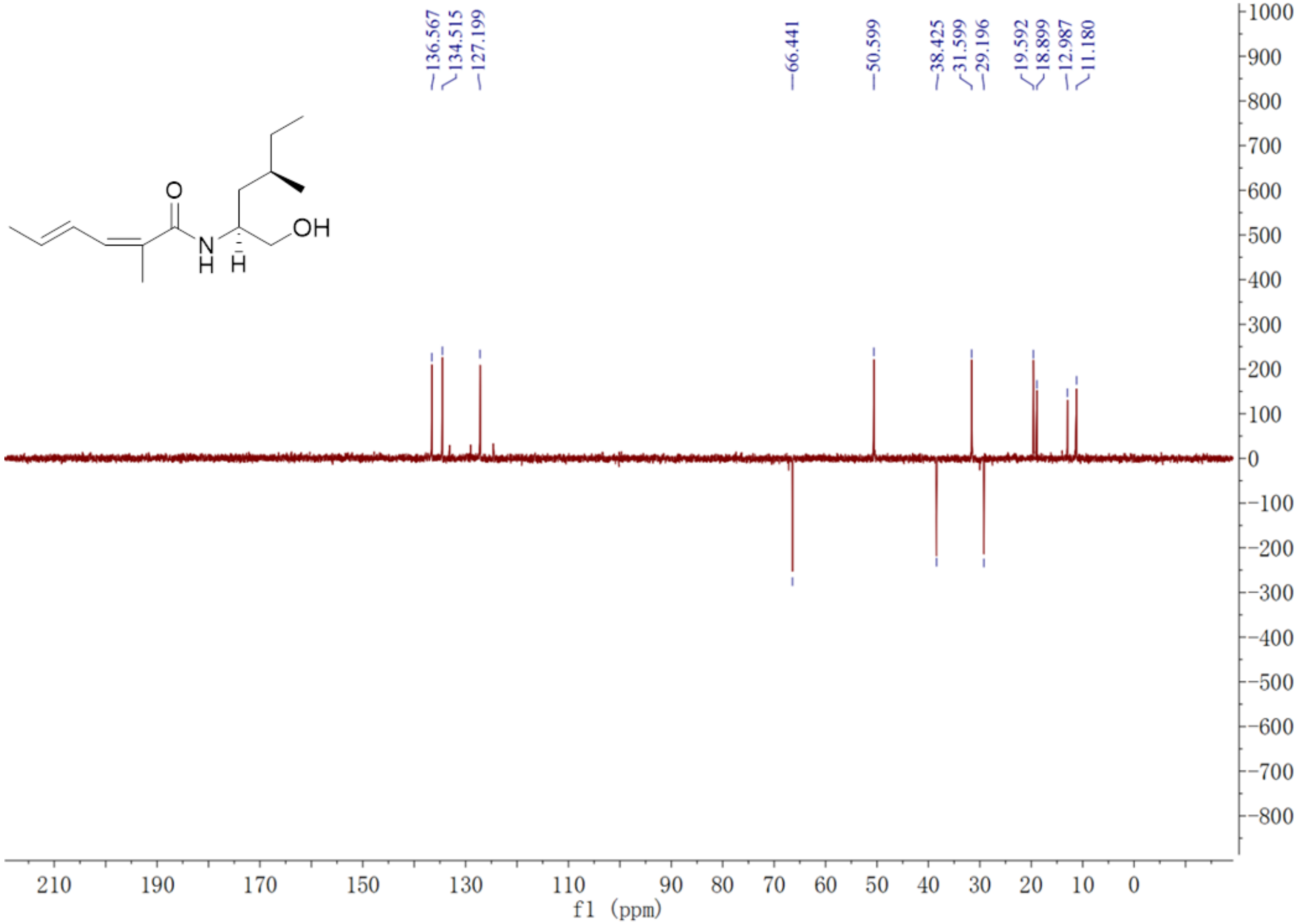

Figure S13. HMQC spectrum of dichotomocej B (**2**) in $CDCl_3$

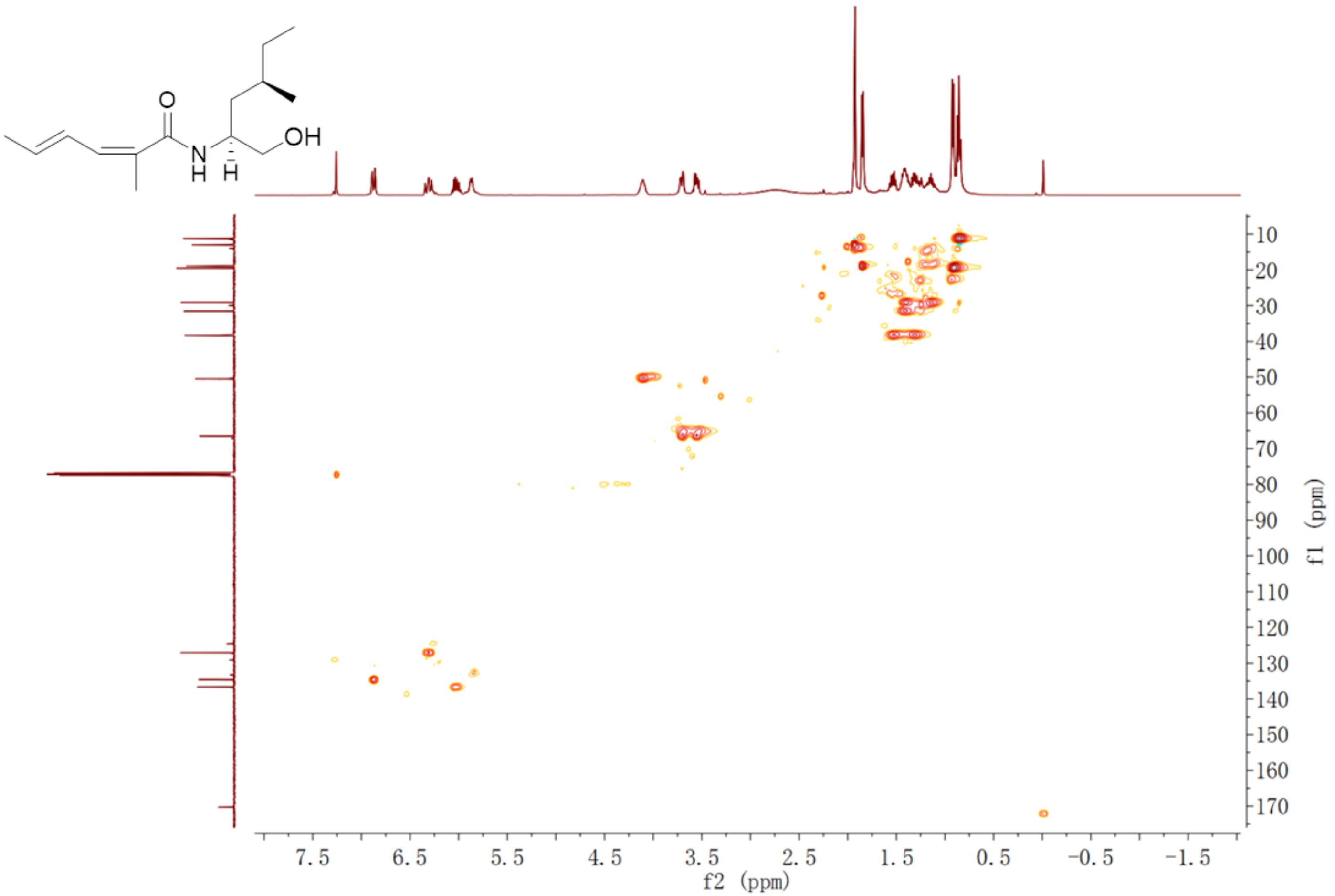

Figure S14. ^{1}H-^{1}H COSY spectrum of dichotomocej B (**2**) in $CDCl_3$

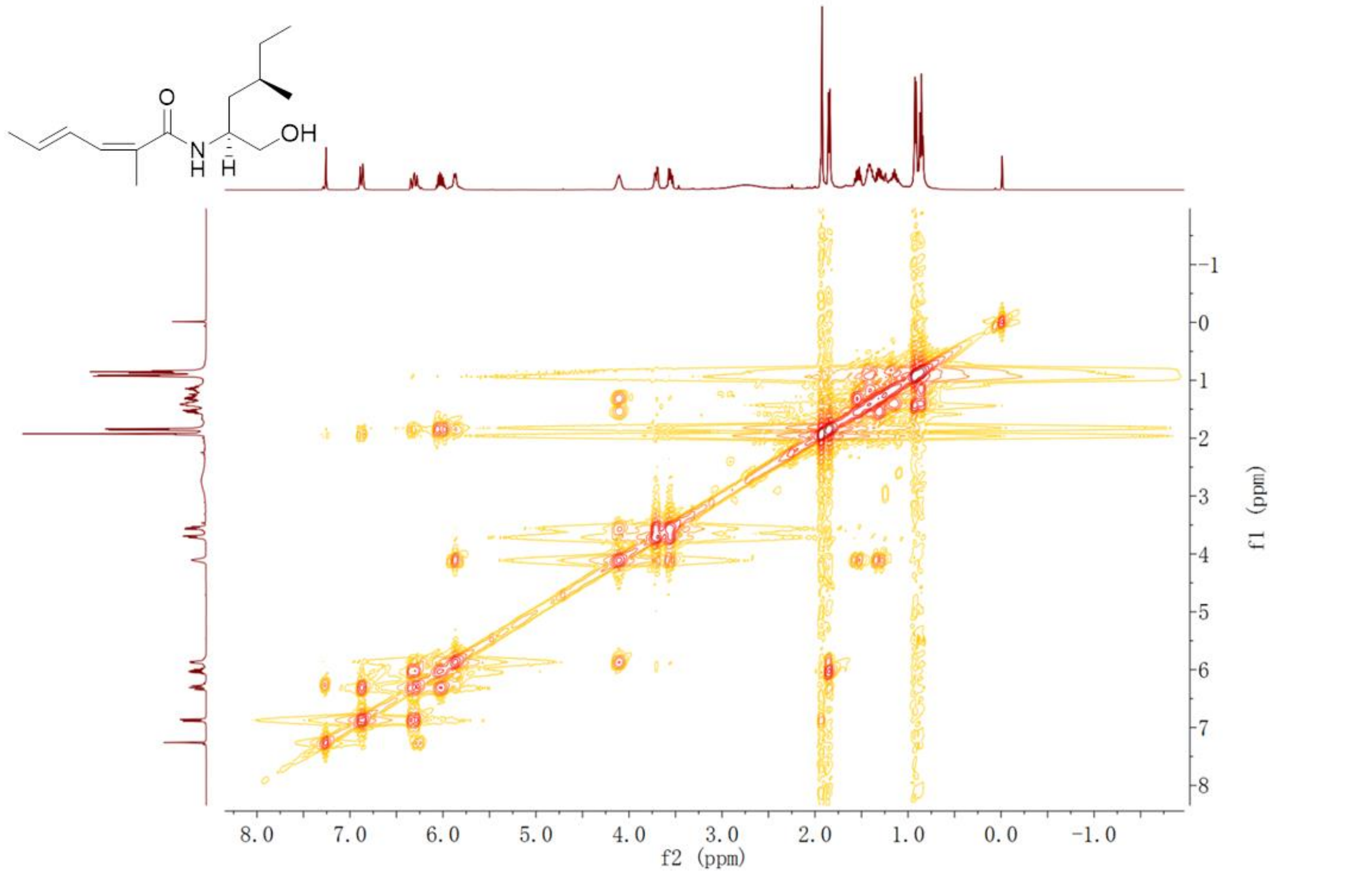

Figure S15. HMBC spectrum of dichotomocej B (**2**) in $CDCl_3$

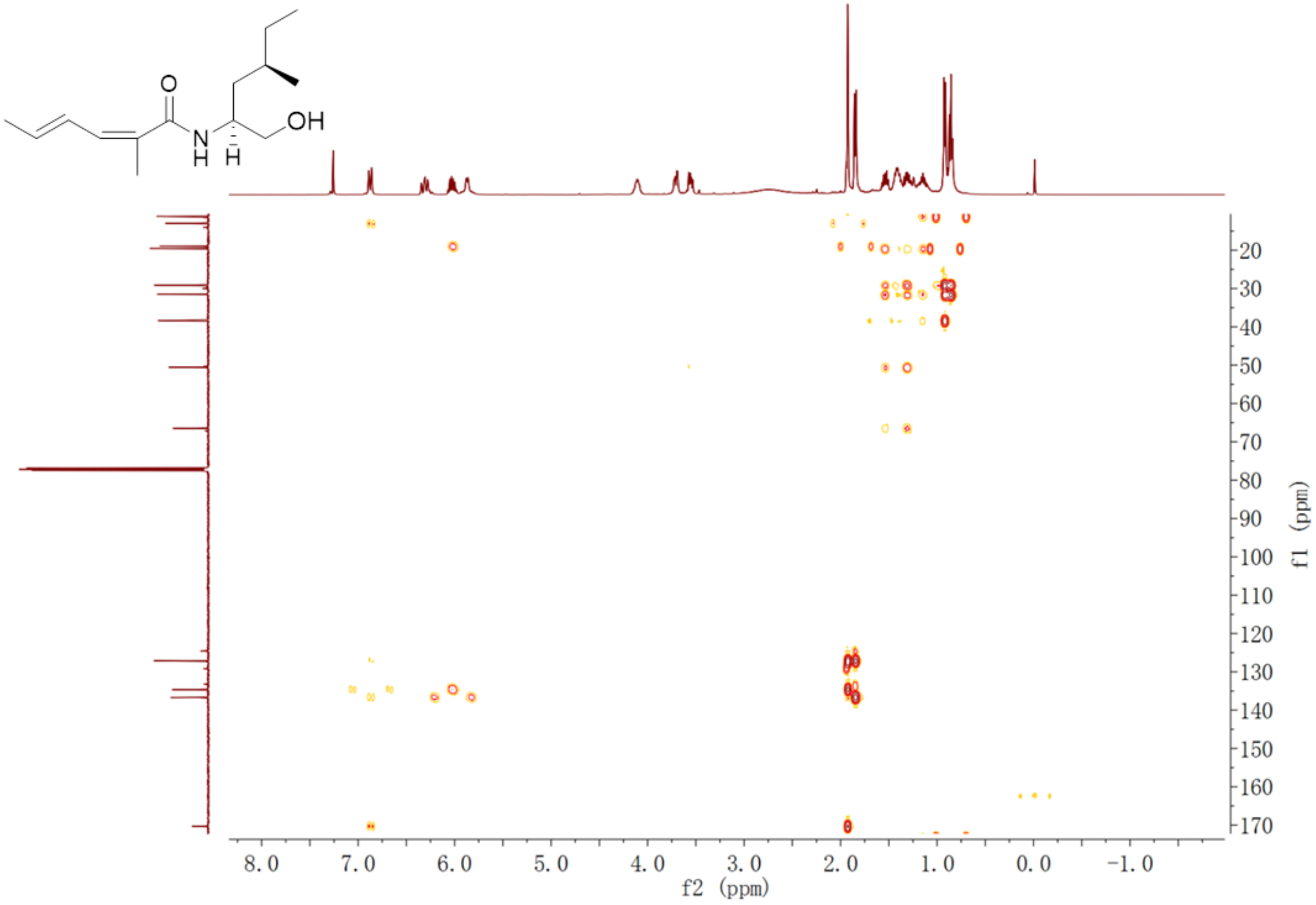

Figure S16. NOESY spectrum of dichotomocej B (**2**) in $CDCl_3$

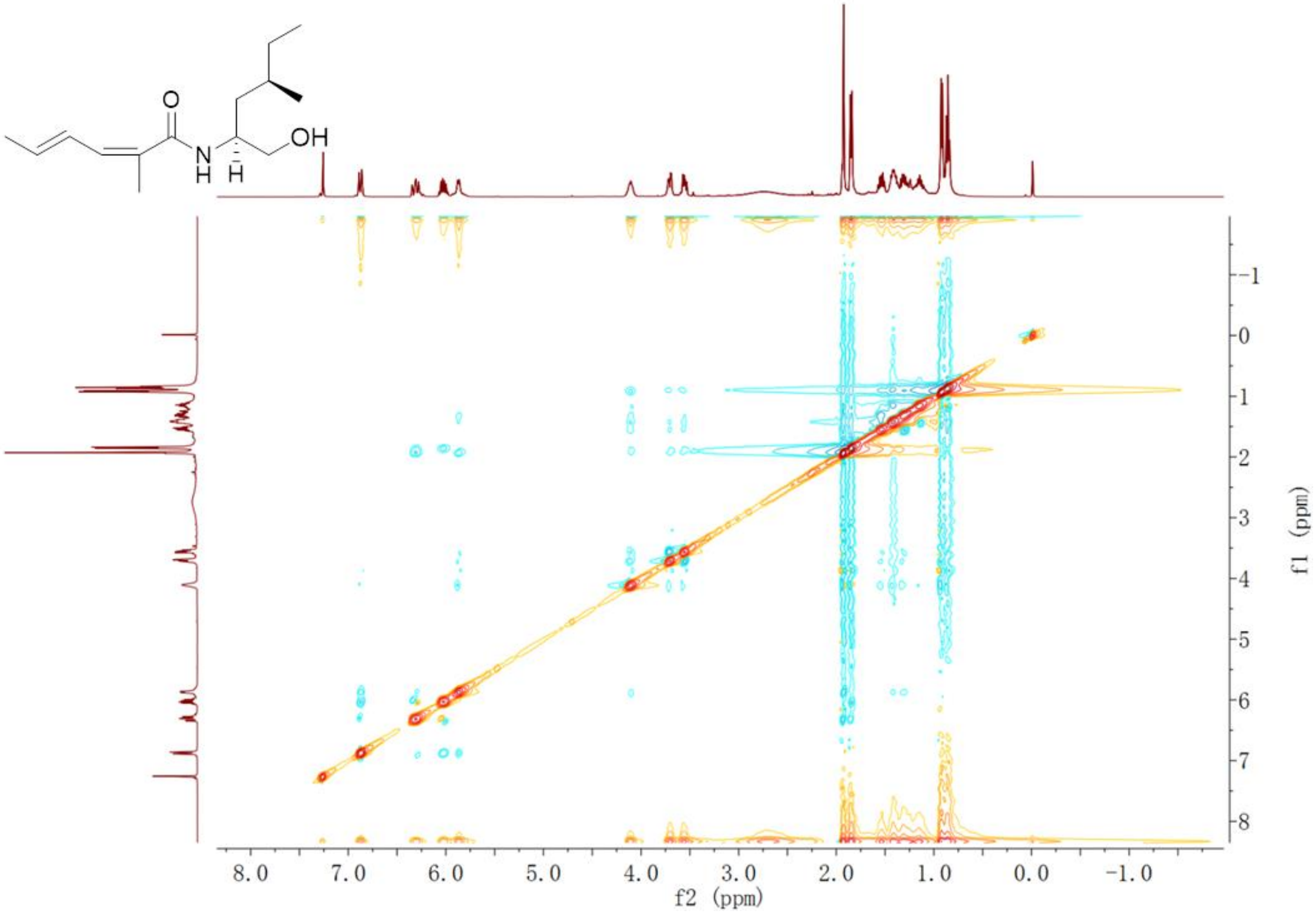

Figure S17. HR-ESI-MS spectrum of dichotomocej C (**3**)

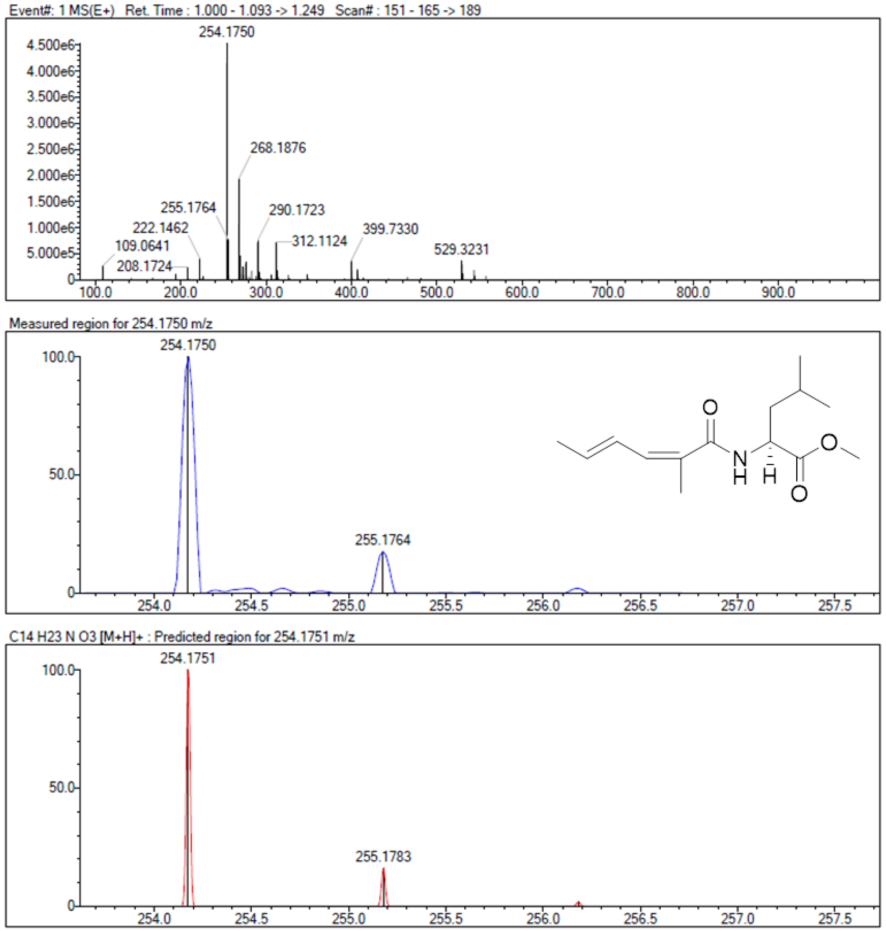

Rank	Score	Formula (M)	Ion	Meas. m/z	Pred. m/z	Df. (mDa)	Df. (ppm)	Iso	DBE
> 1	71.89	C14 H23 N O3	[M+H]+	254.1750	254.1751	-0.1	-0.39	71.89	4.0

Figure S18. ^{1}H NMR spectrum of dichotomocej C (**3**) in $CDCl_3$ (400MHz)

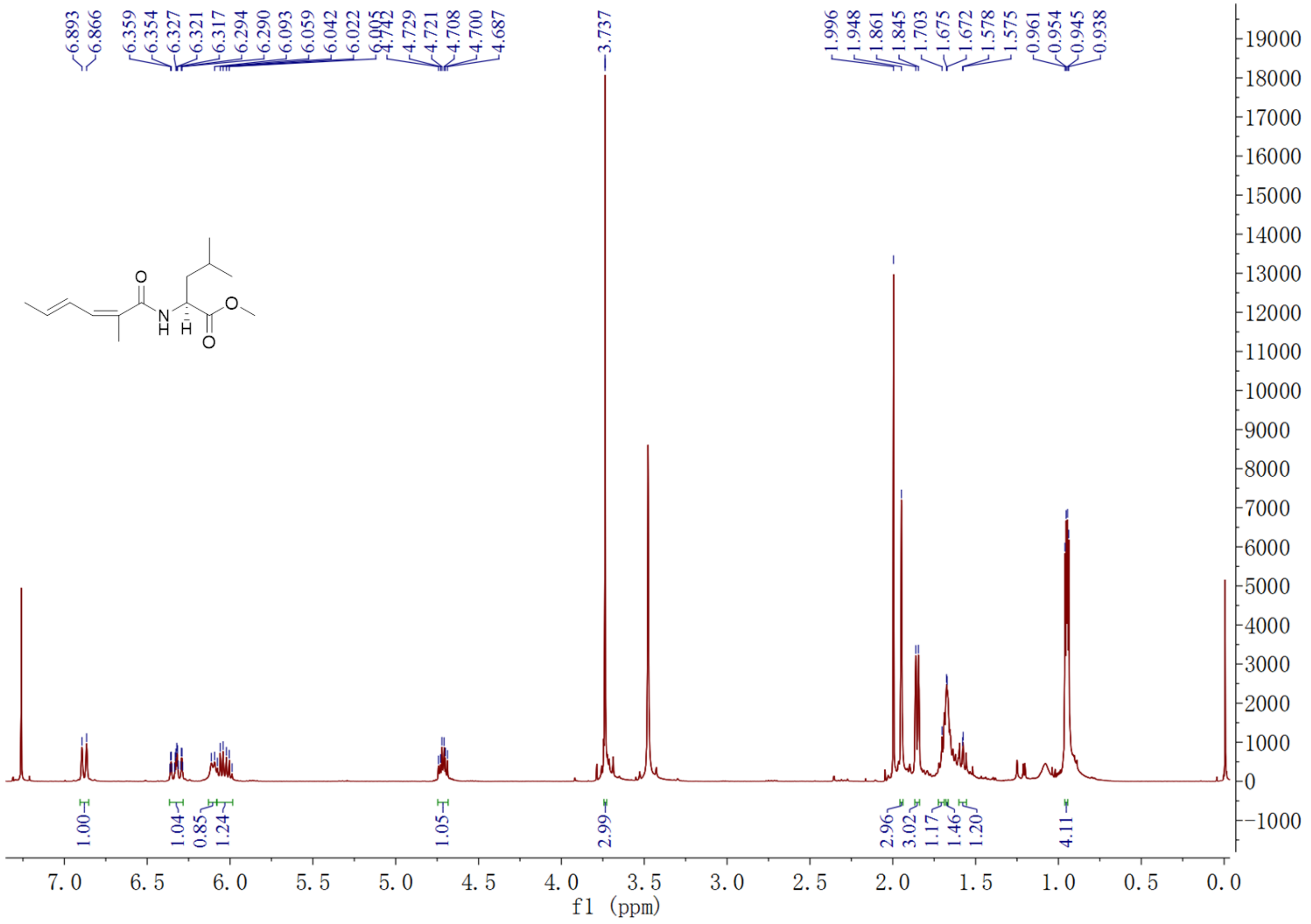

Figure S19. ^{13}C NMR spectrum of dichotomocej C (**3**) in $CDCl_3$ (100MHz)

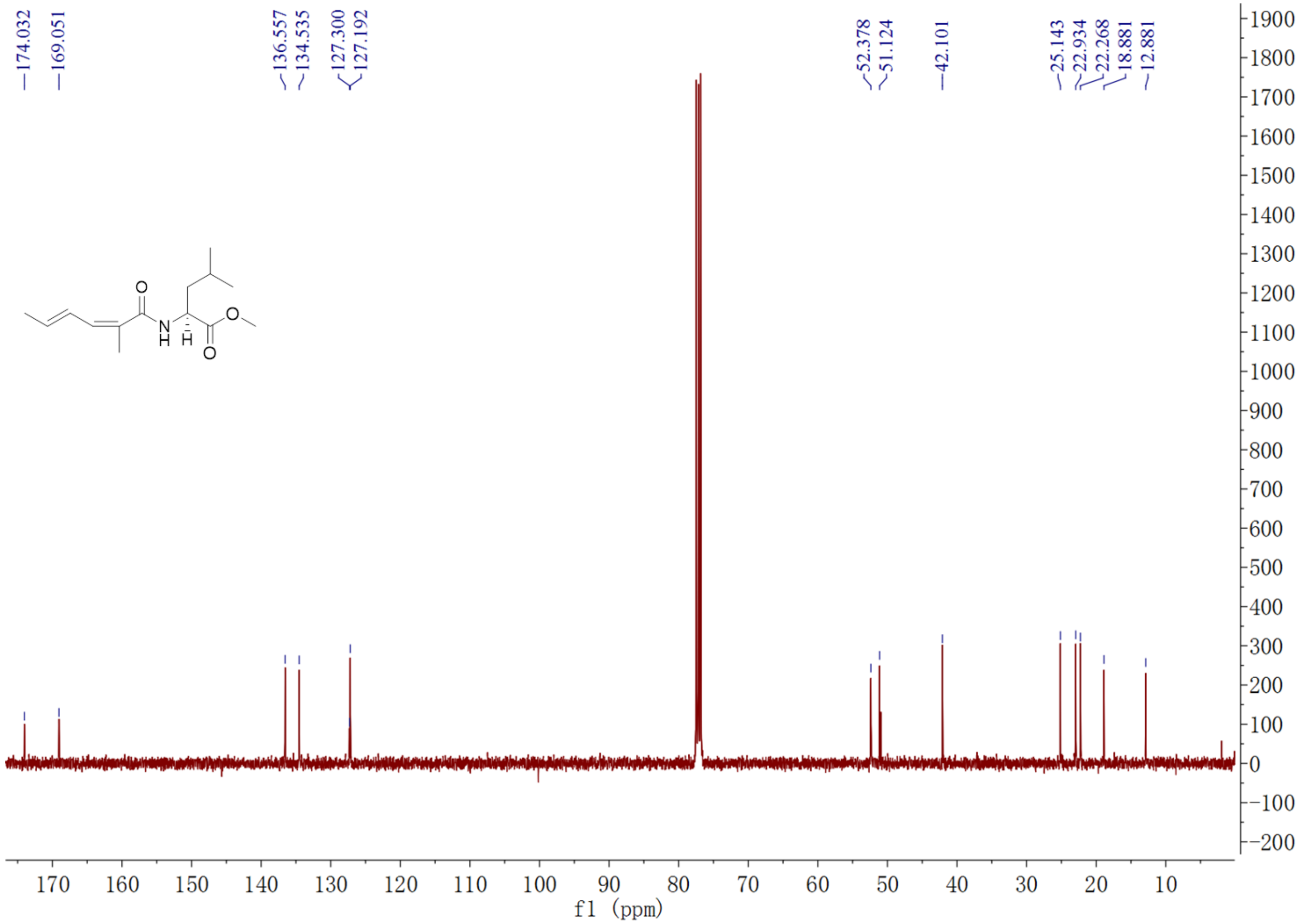

Figure S20. DEPT 135 spectrum of dichotomocej C (**3**) in $CDCl_3$ (100MHz)

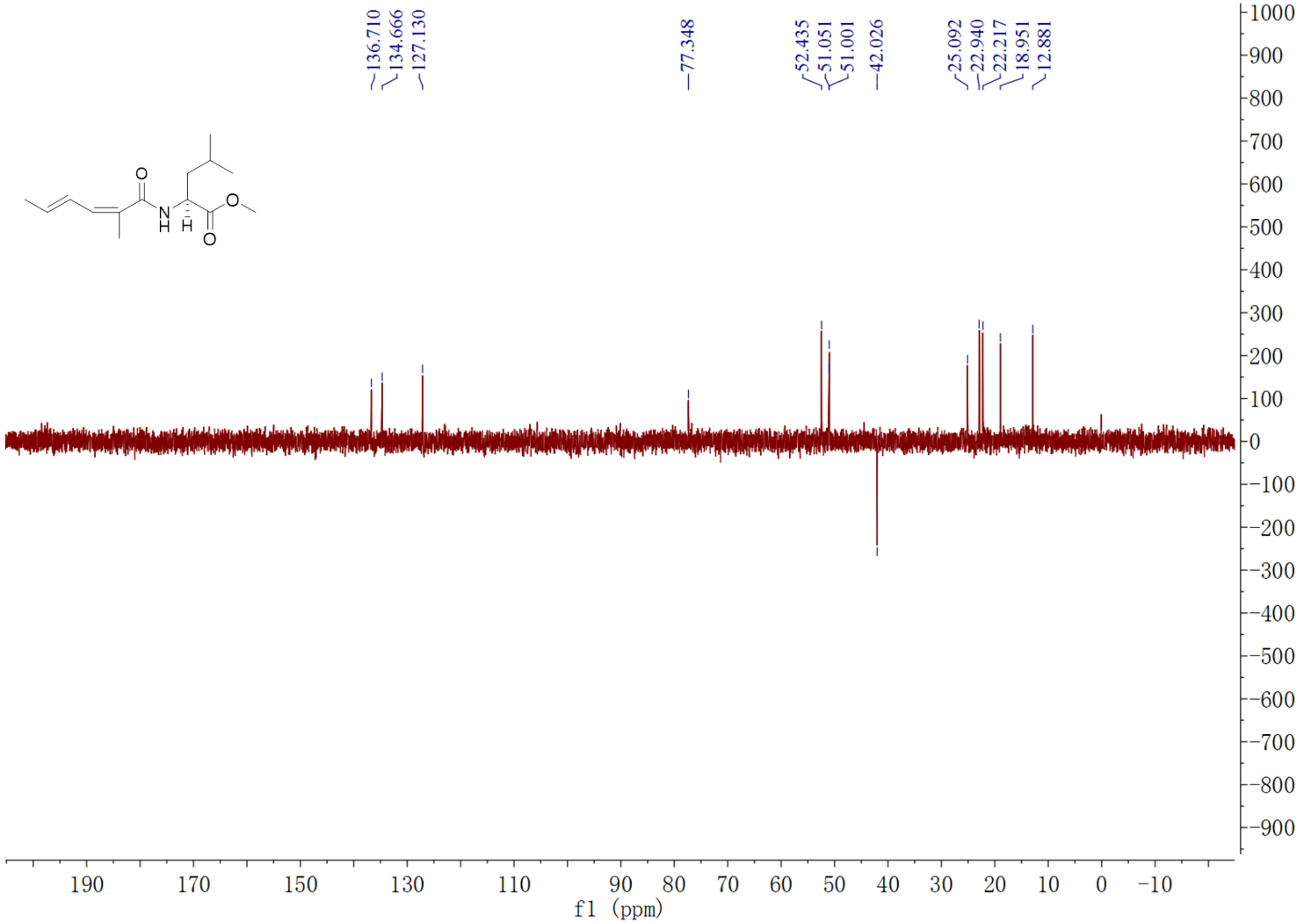

Figure S21. HMQC spectrum of dichotomocej C (**3**) in $CDCl_3$

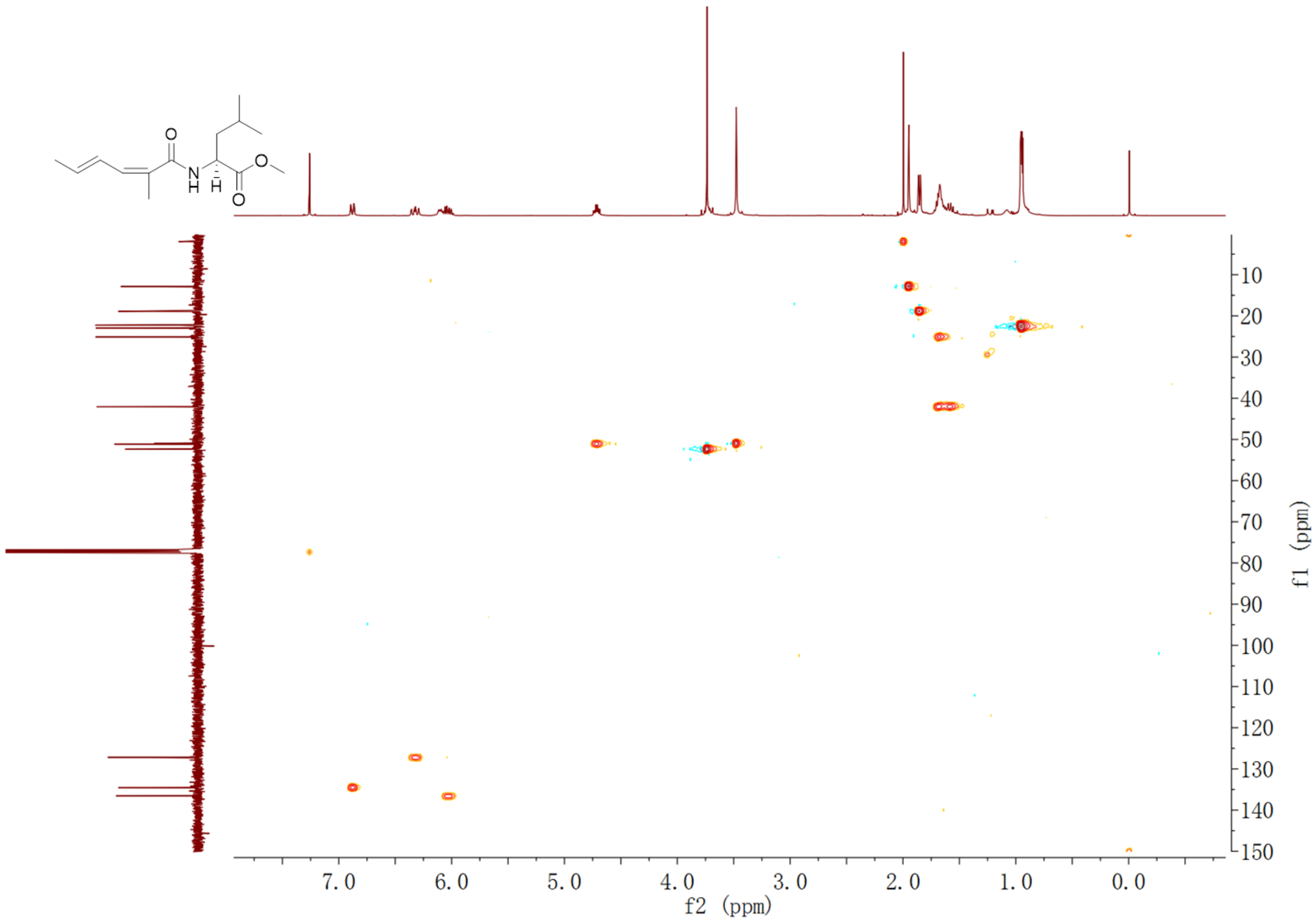

Figure S22. ^{1}H-^{1}H COSY spectrum of dichotomocej C (**3**) in $CDCl_3$

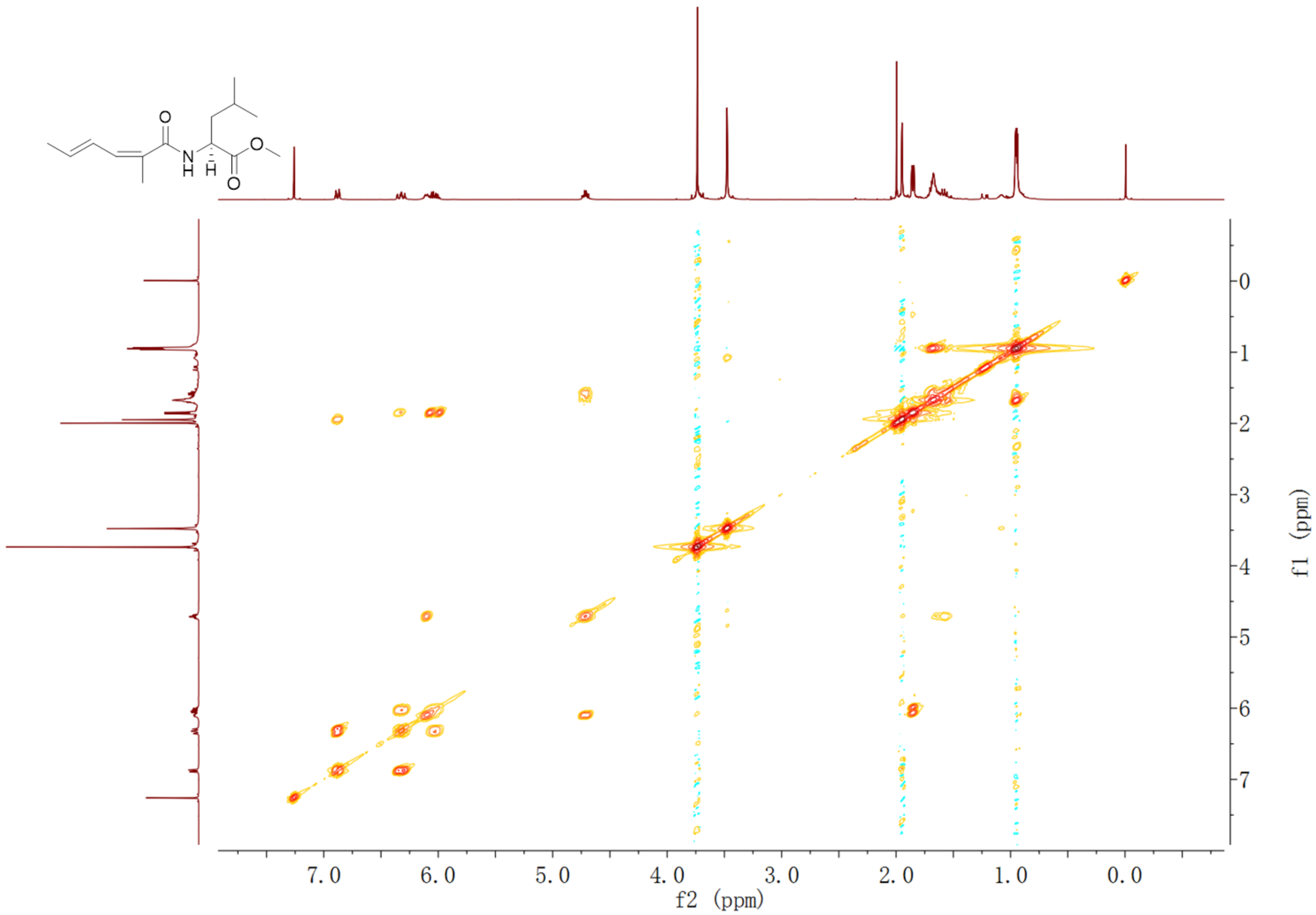

Figure S23. HMBC spectrum of dichotomocej C (**3**) in $CDCl_3$

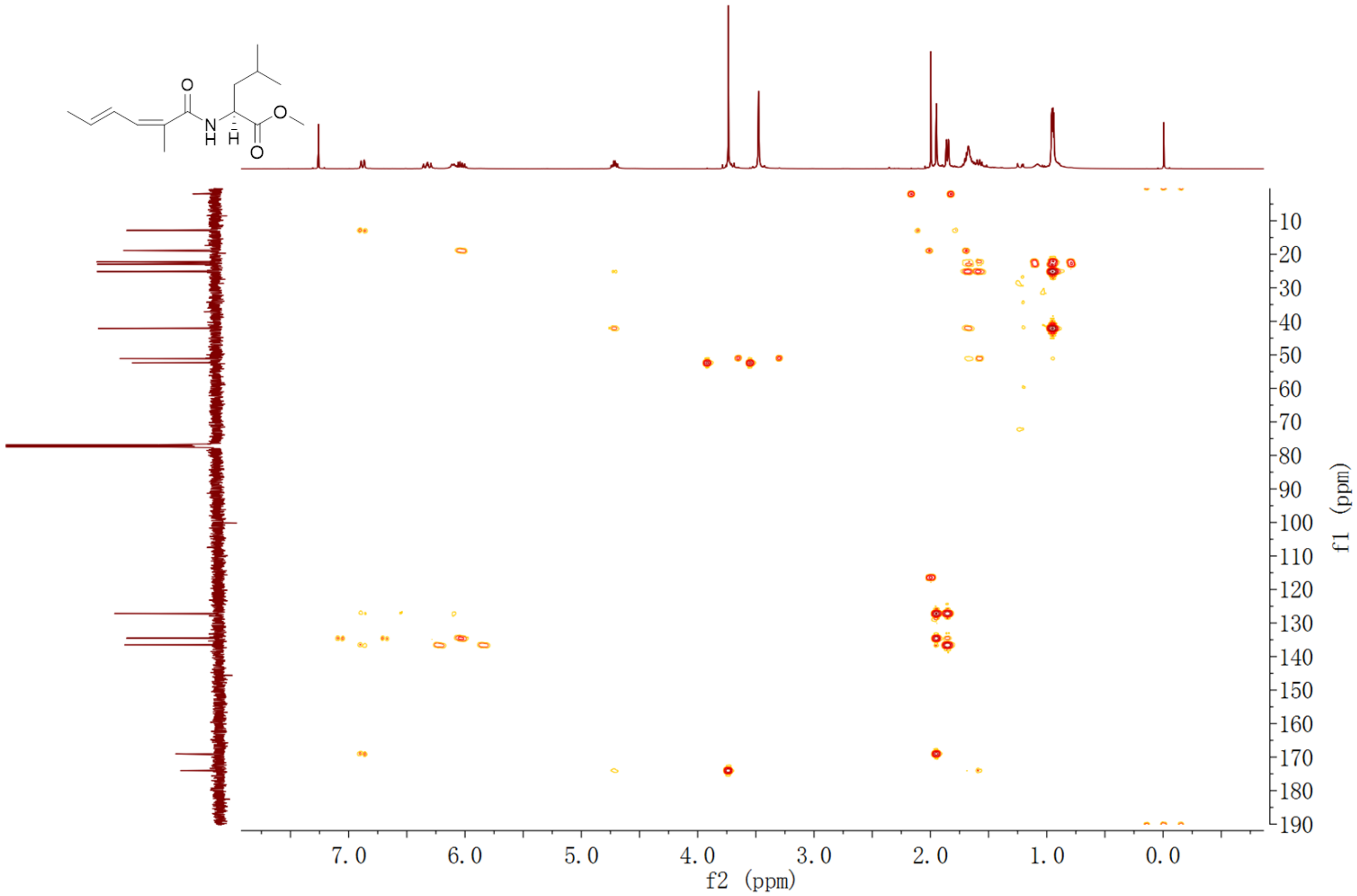

Figure S24. NOESY spectrum of dichotomocej C (**3**) in $CDCl_3$

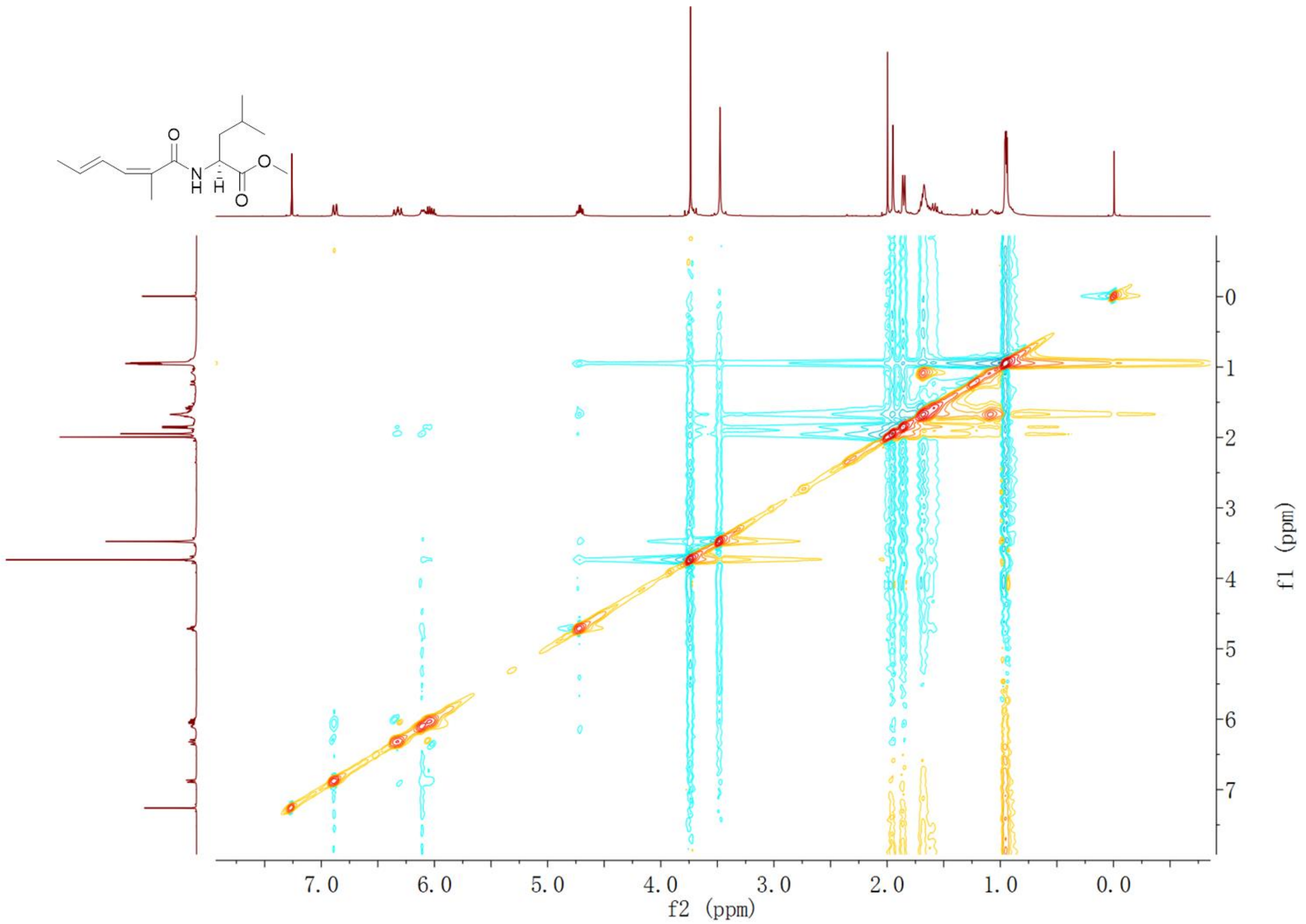

Figure S25. HR-ESI-MS spectrum of dichotomocej D (**4**)

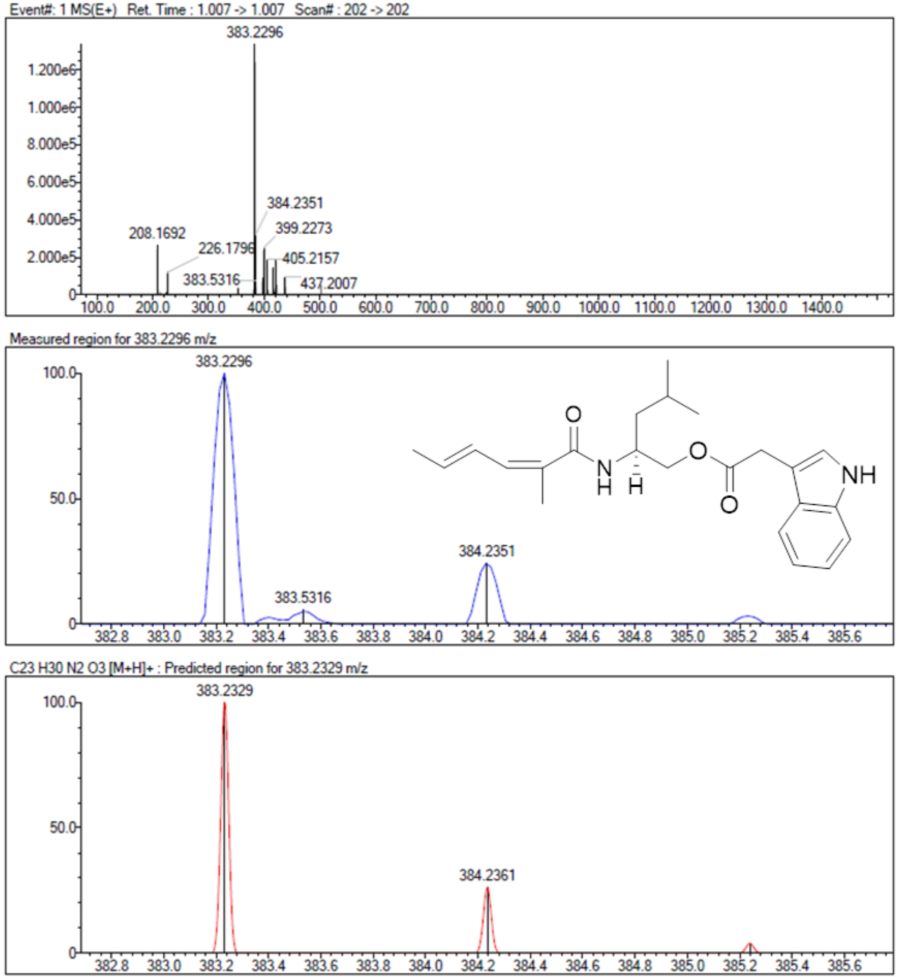

Rank	Score	Formula (M)	Ion	Meas. m/z	Pred. m/z	Df. (mDa)	Df. (ppm)	Iso	DBE
1	45.36	C23 H30 N2 O3	[M+H]+	383.2296	383.2329	-3.3	-8.61	84.16	10.0

Figure S26. ^{1}H NMR spectrum of dichotomocej D (**4**) in $CDCl_3$ (400MHz)

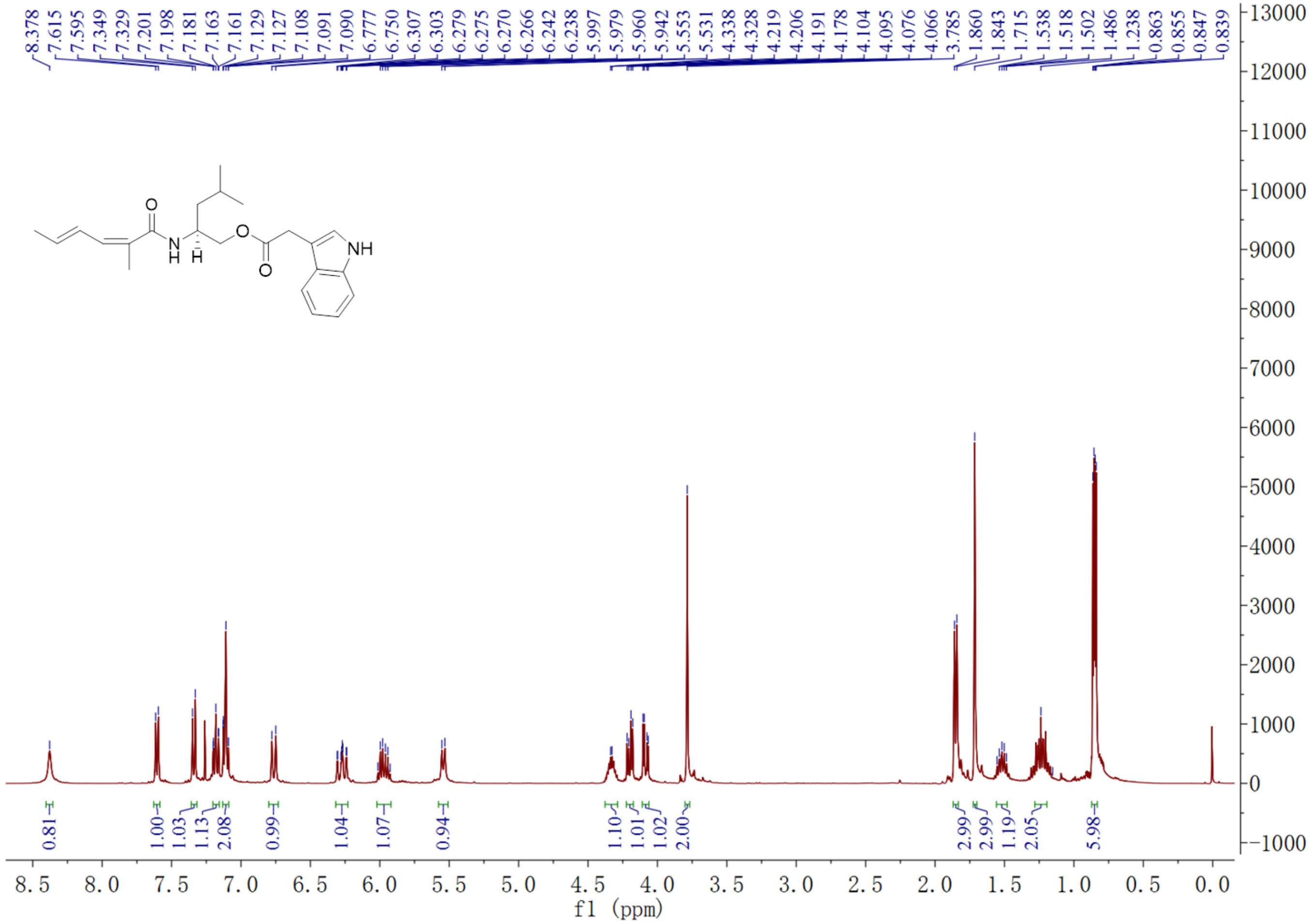

Figure S27. ^{13}C NMR spectrum of dichotomocej D (**4**) in $CDCl_3$ (100MHz)

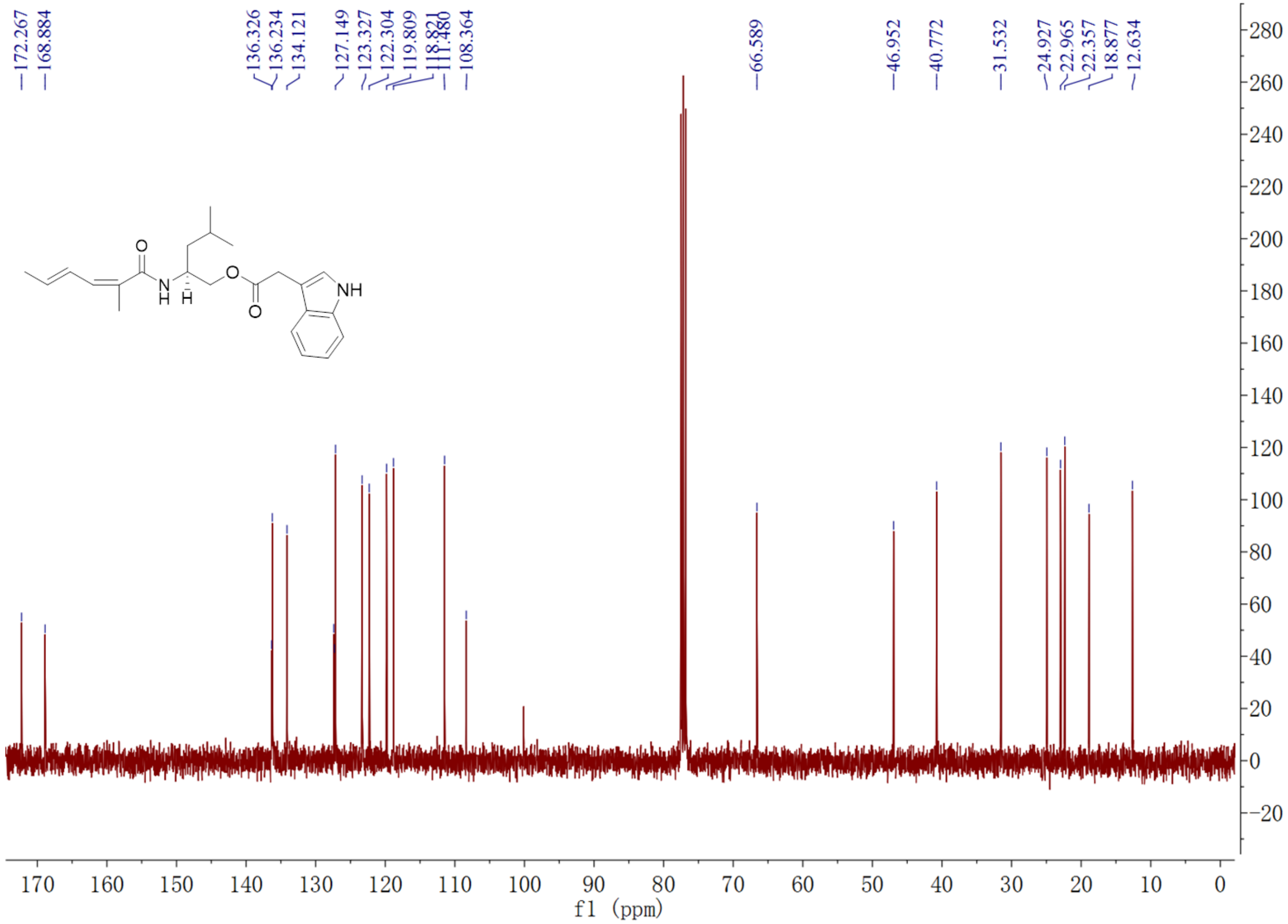

Figure S28. DEPT 135 spectum of dichotomocej D (**4**) in $CDCl_3$ (100MHz)

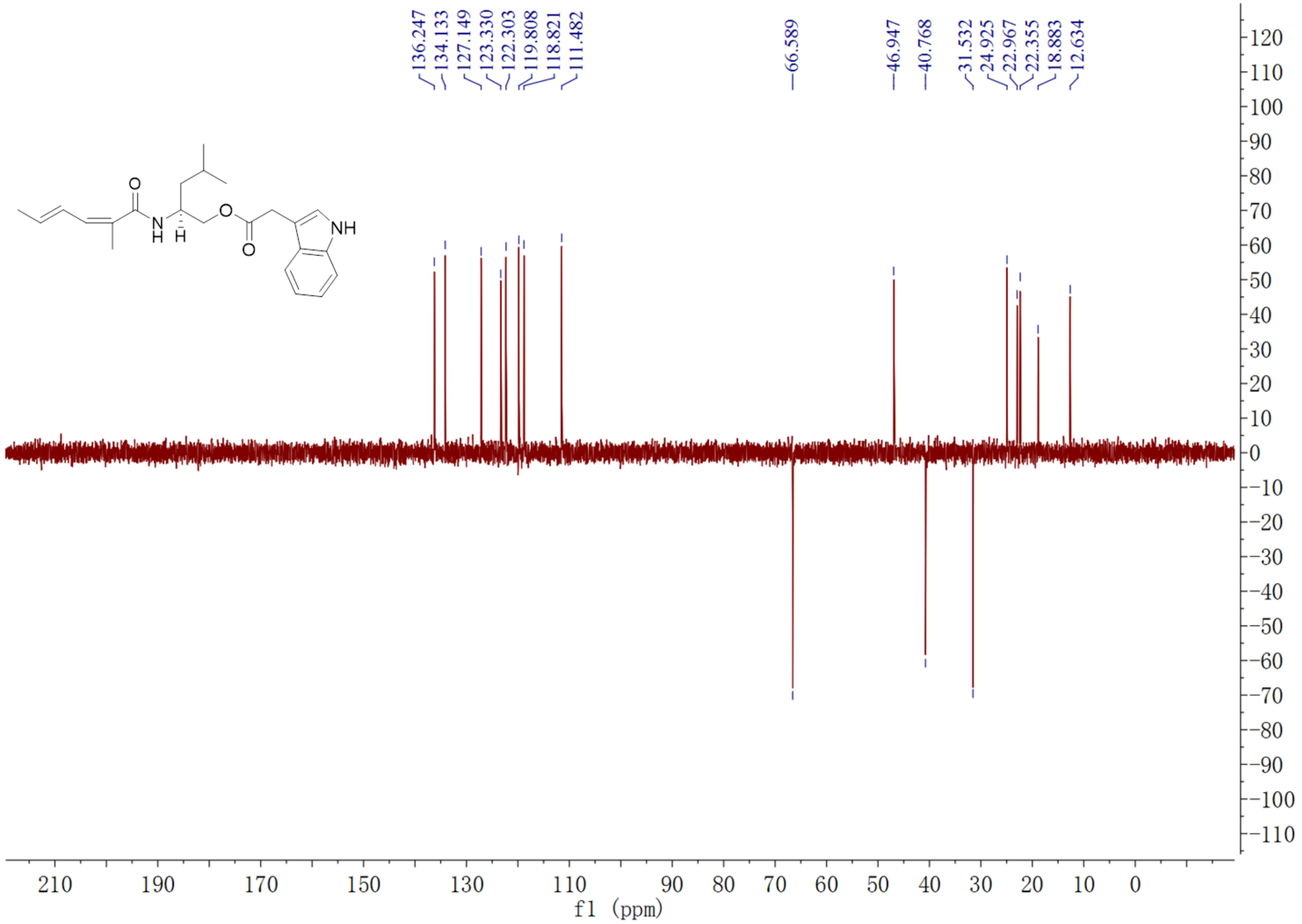

Figure S29. HMQC spectrum of dichotomocej D (**4**) in $CDCl_3$

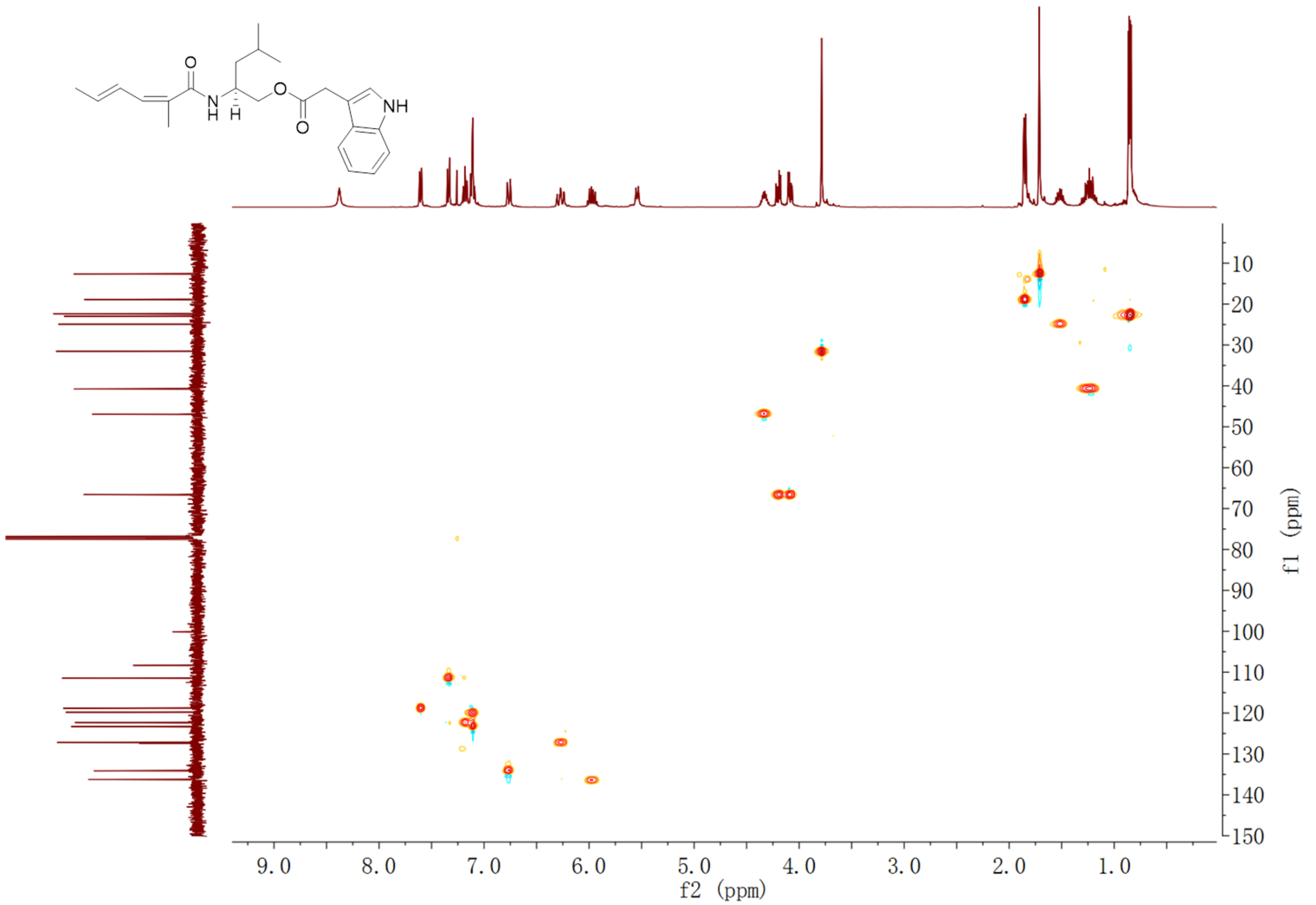

Figure S30. ^{1}H-^{1}H COSY spectrum of dichotomocej D (**4**) in $CDCl_3$

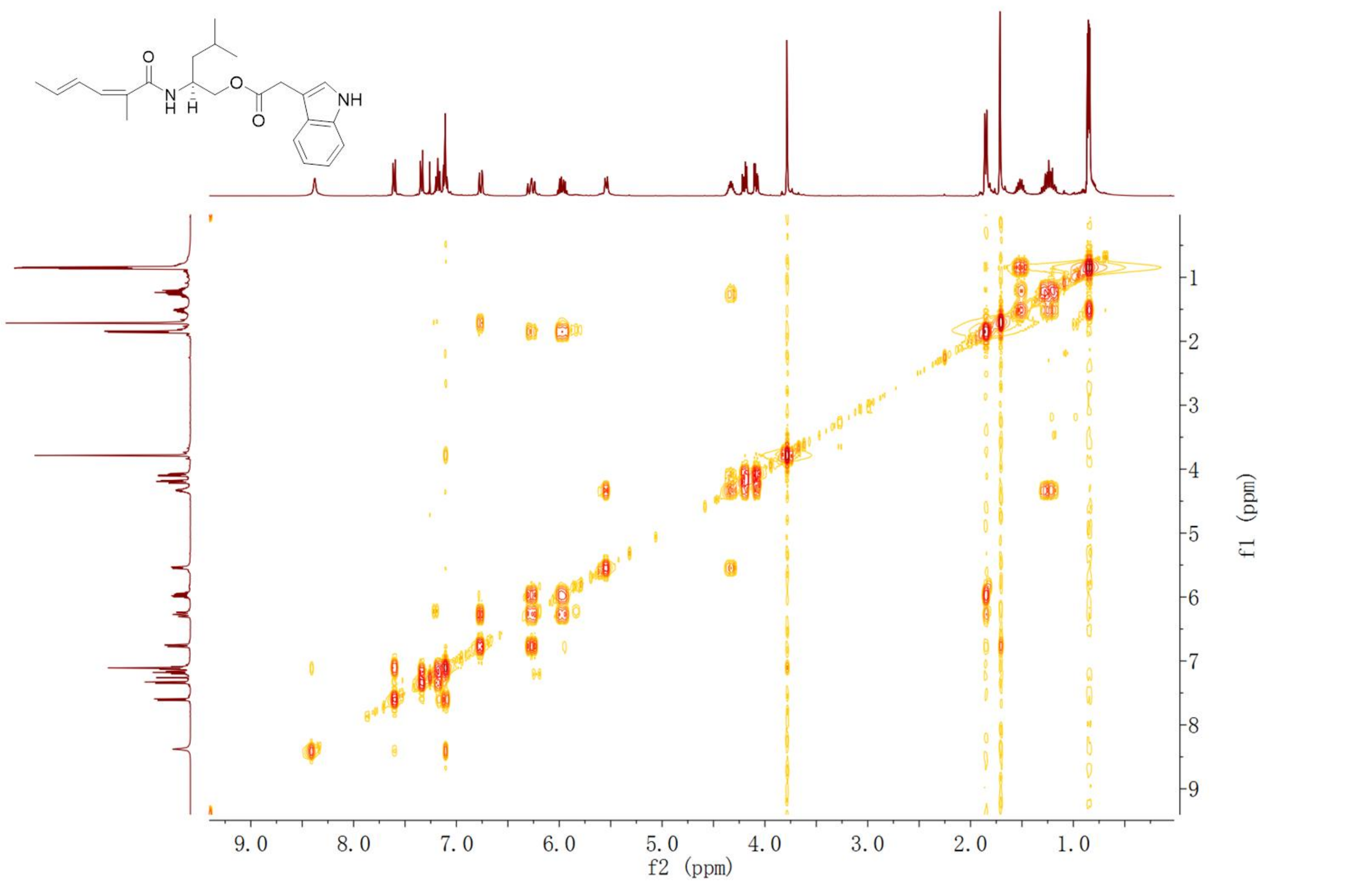

Figure S31. HMBC spectrum of dichotomocej D (**4**) in $CDCl_3$

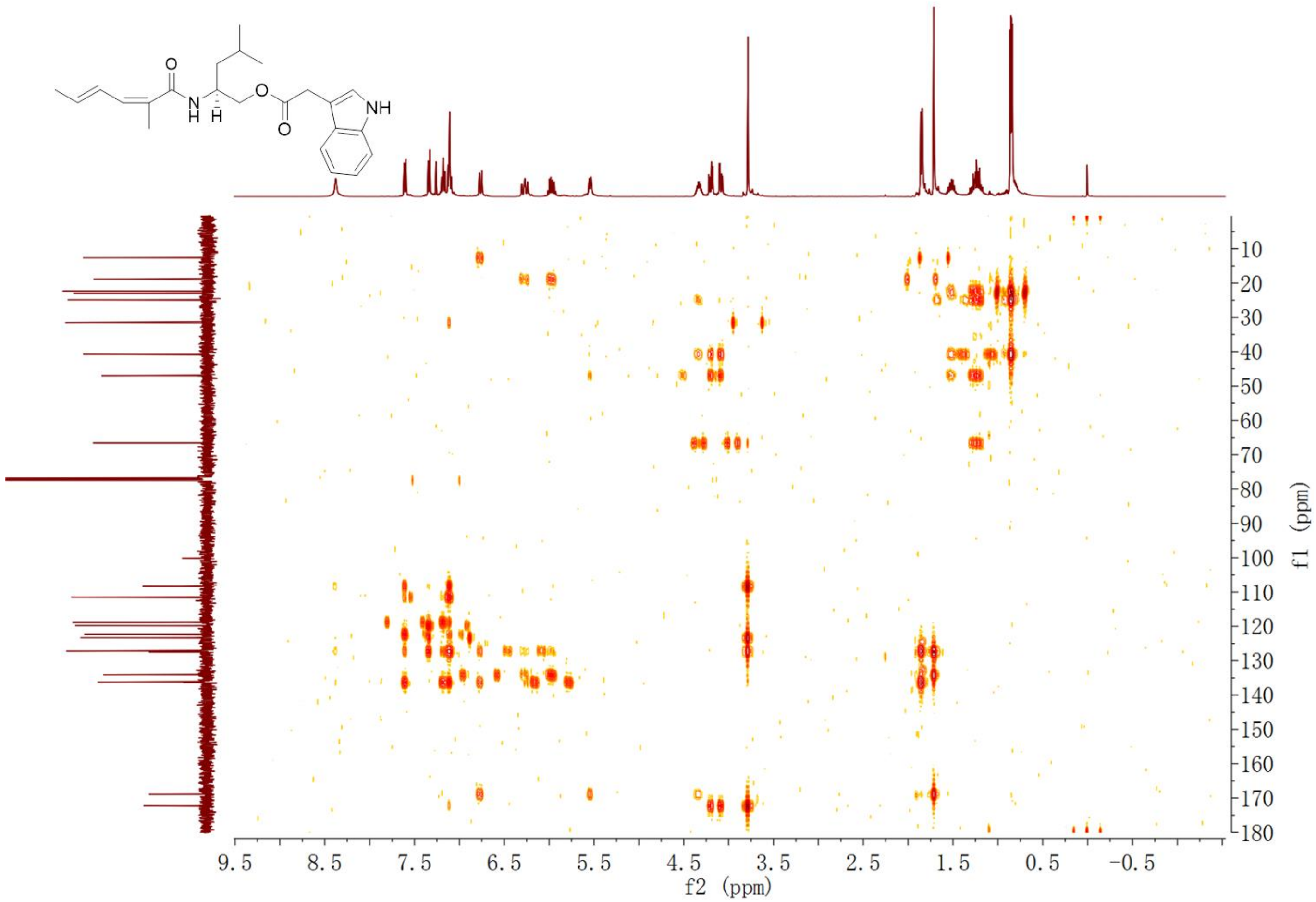

Figure S32. NOESY spectrum of dichotomocej D (**4**) in $CDCl_3$

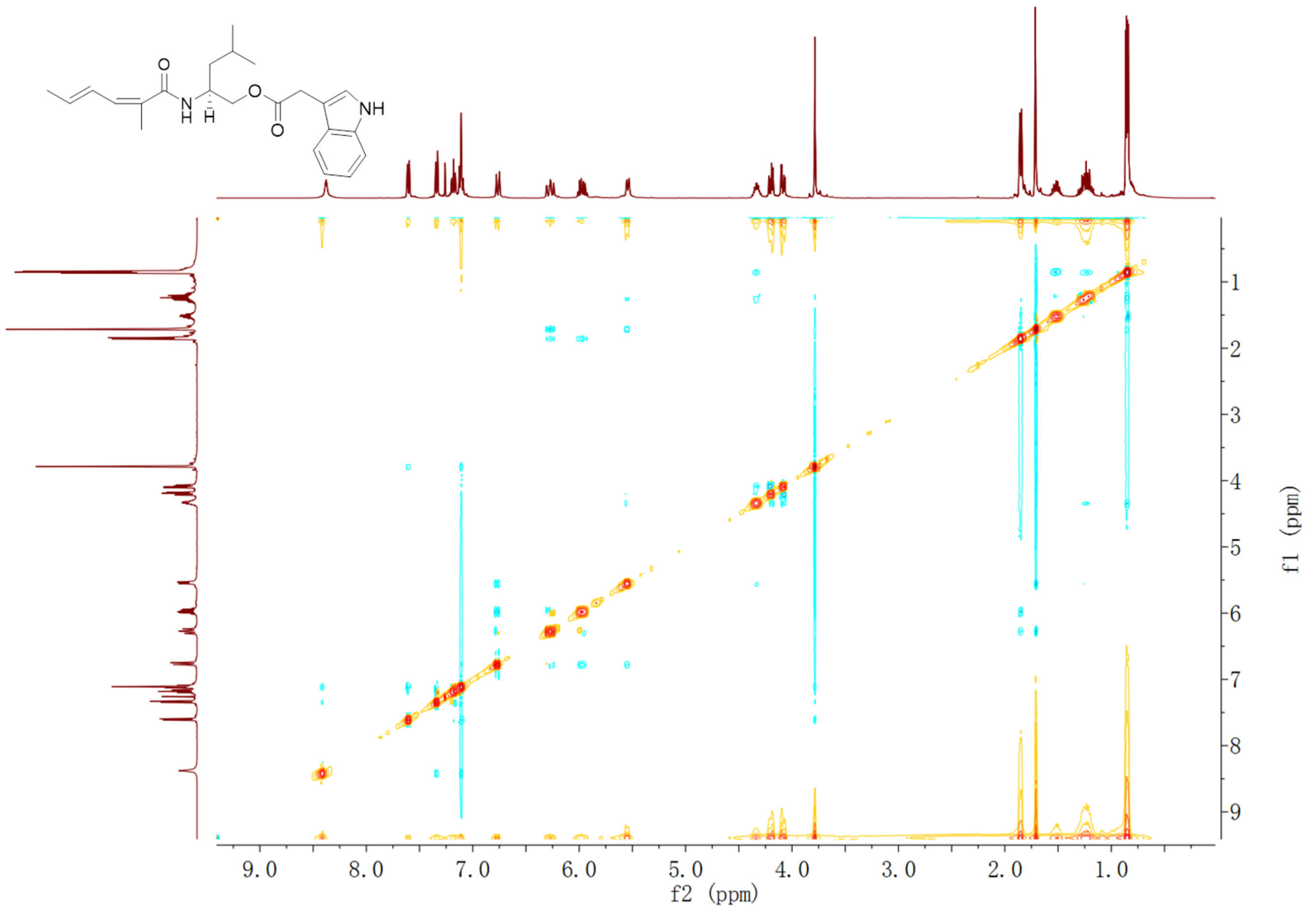

Figure S33. HR-ESI-MS spectrum of dichocetide A (**5**)

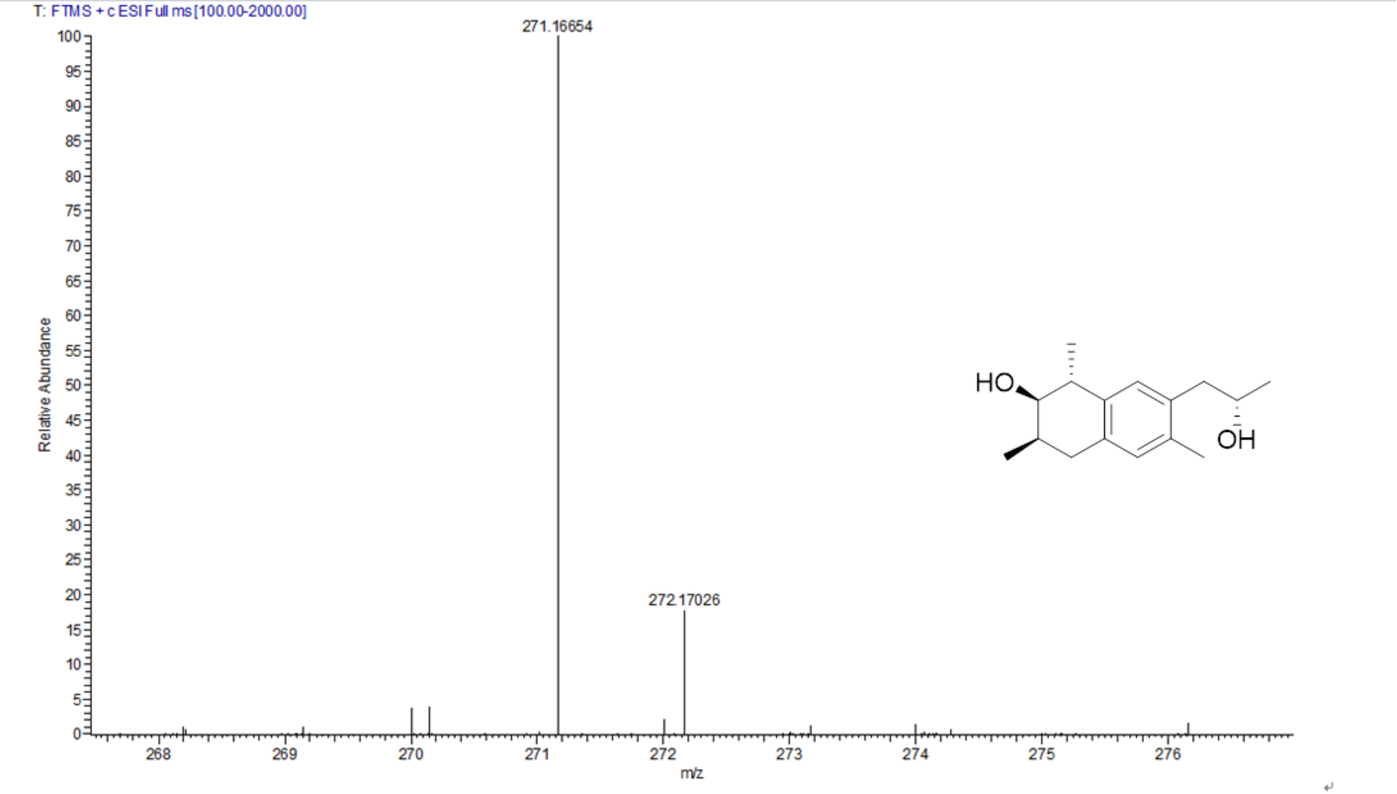

SPECTRUM - simulation :				
m/z	Theo. Mass	Delta (ppm)	RDB equiv.	Composition
271.16654	271.16685	-1.15	4.5	C16 H24 O2 Na

Figure S34. ^{1}H NMR spectrum of dichocetide A (**5**) in $CDCl_3$ (400MHz)

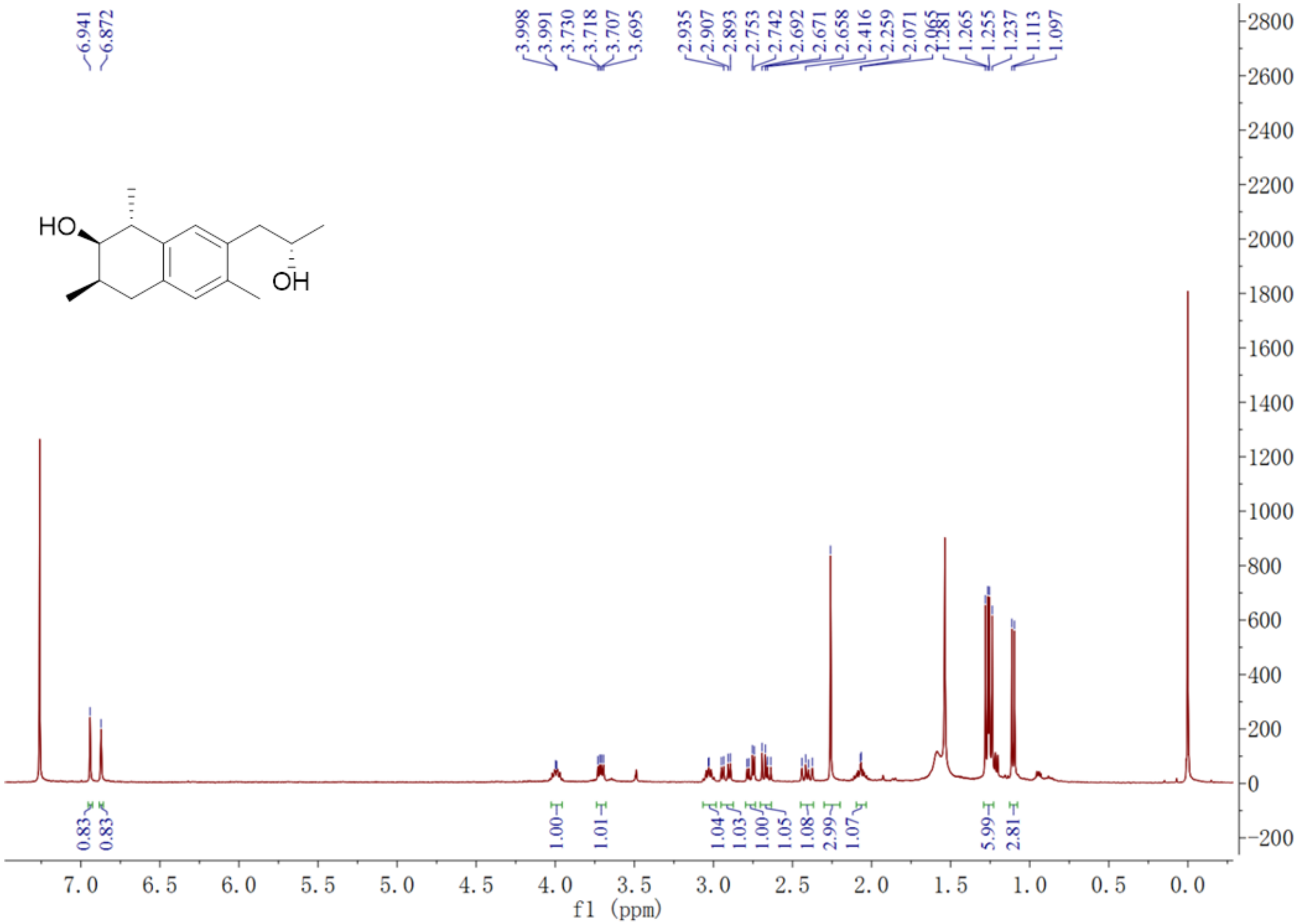

Figure S35. ^{13}C NMR spectrum of dichocetide A (**5**) in $CDCl_3$ (100MHz)

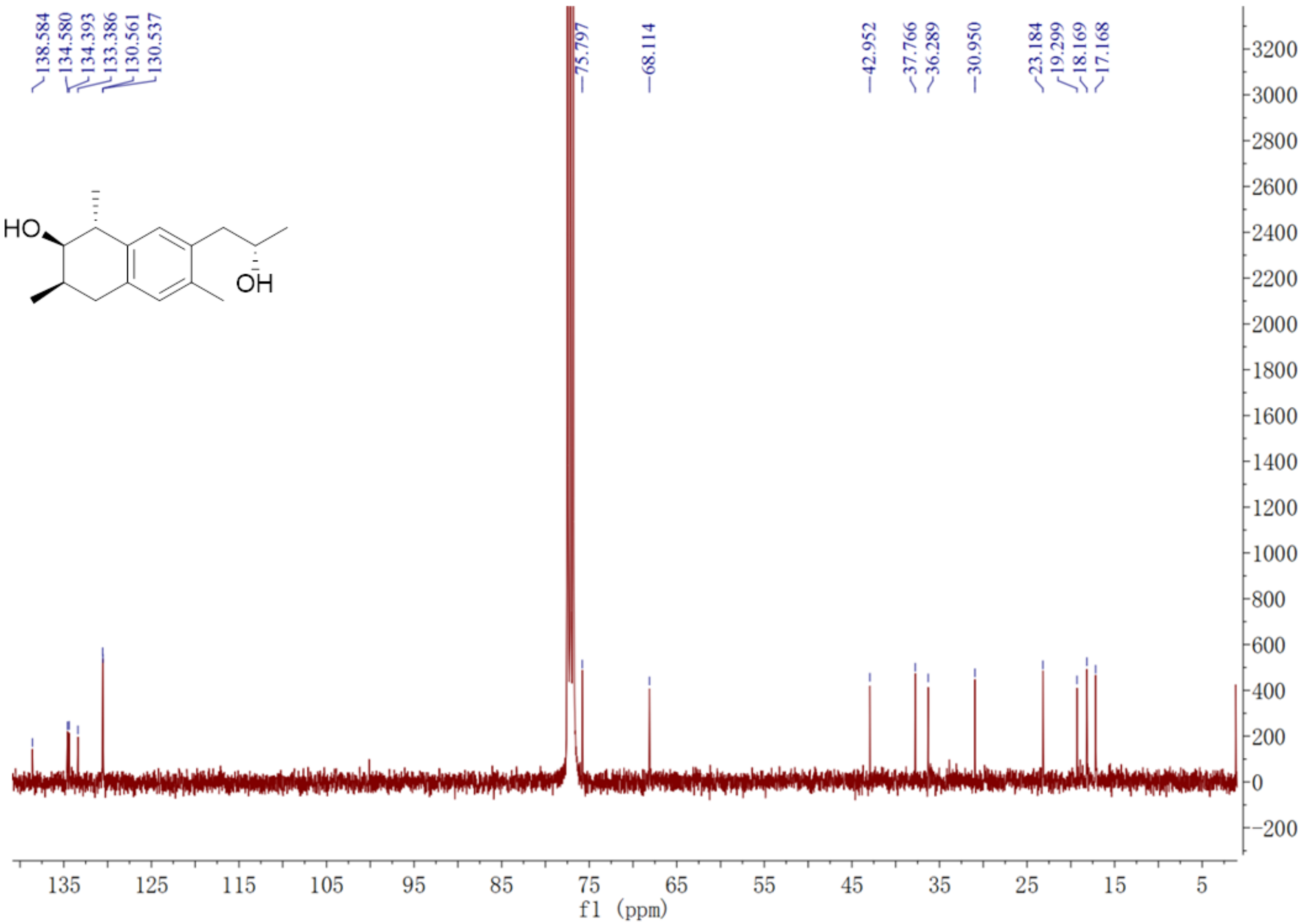

Figure S36. DEPT 135 spectrum of dichocetide A (**5**) in $CDCl_3$ (100MHz)

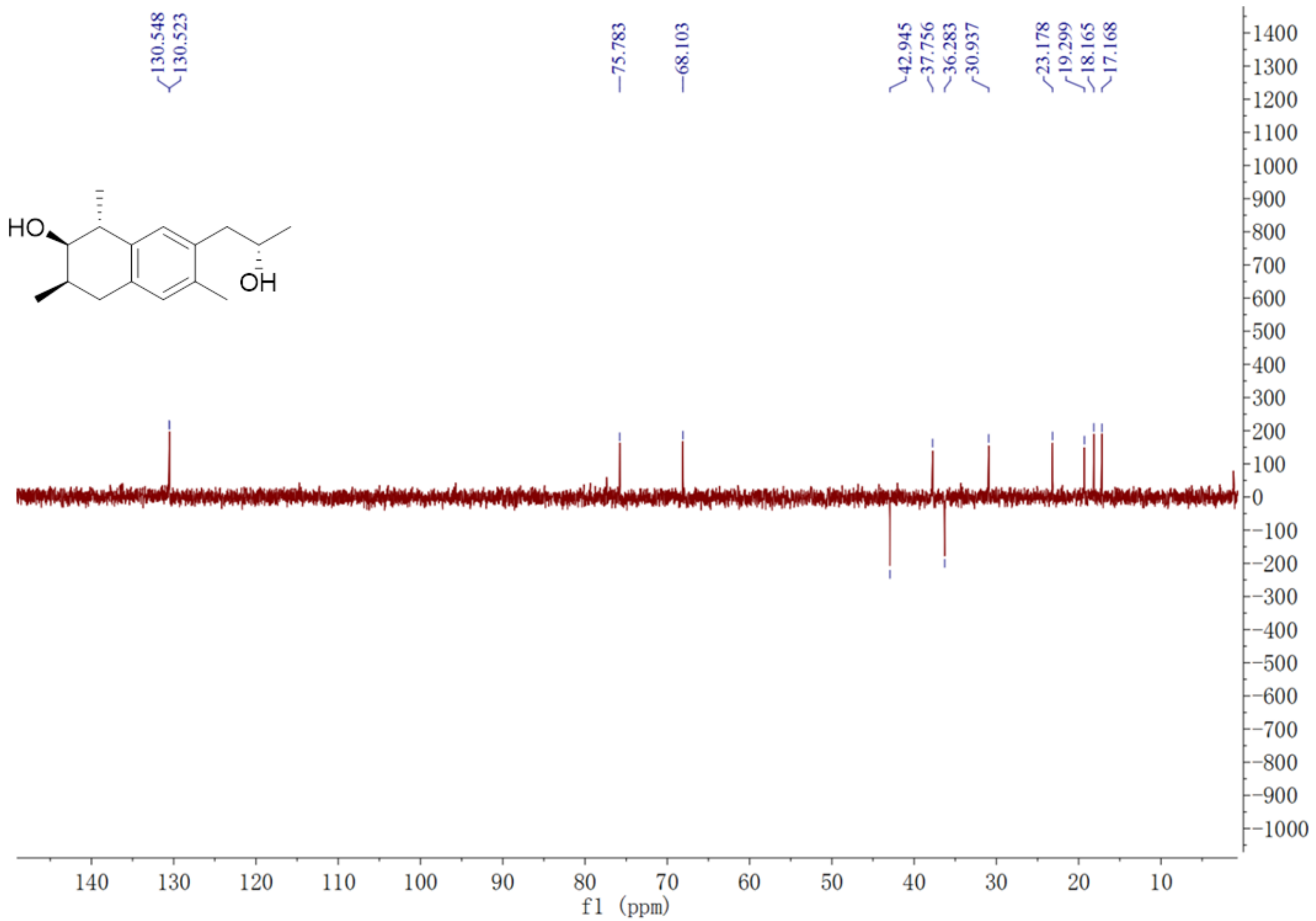

Figure S37. HMQC spectrum of dichocetide A (**5**) in $CDCl_3$

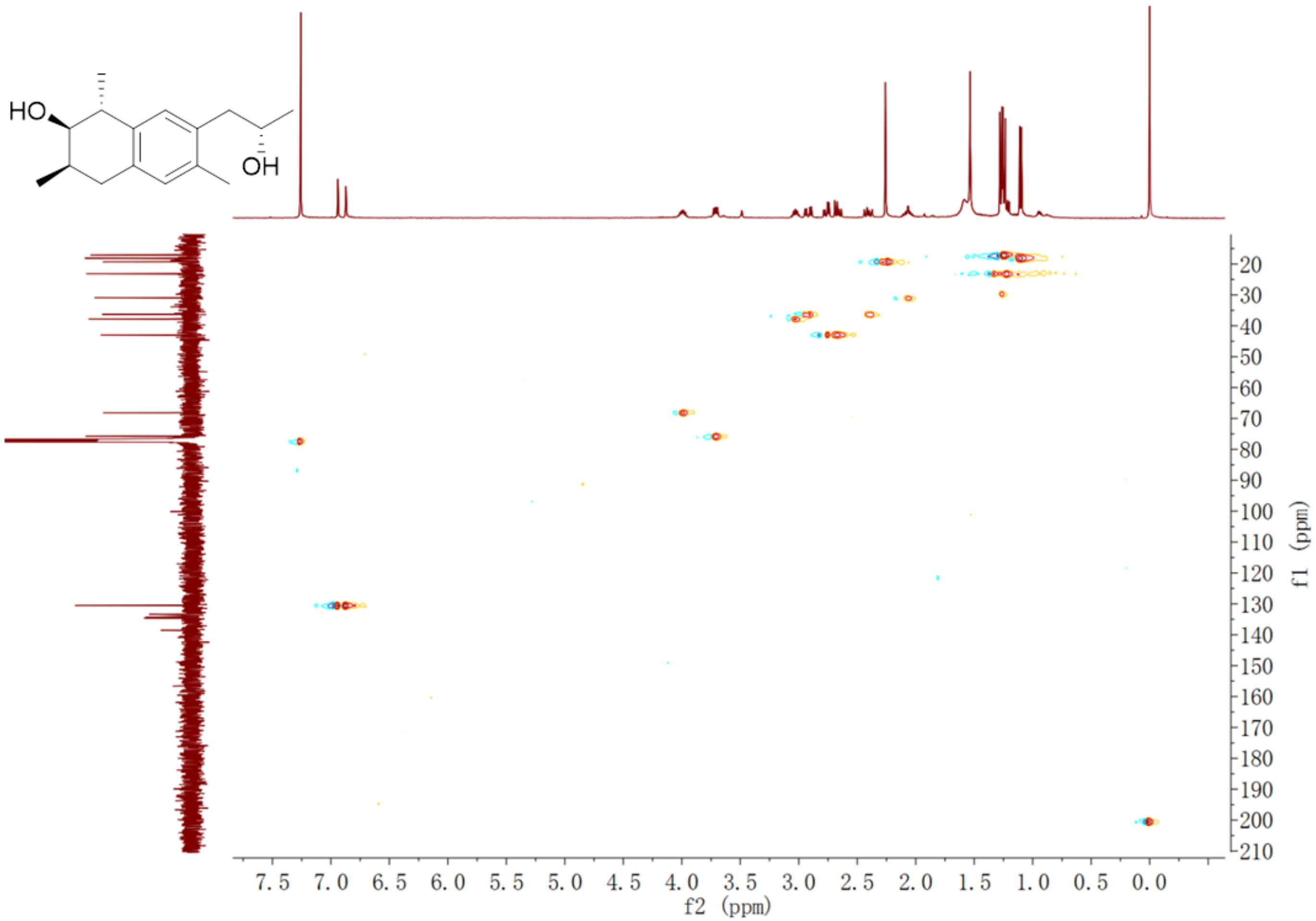

Figure S38. ^{1}H-^{1}H COSY spectrum of dichocetide A (**5**) in $CDCl_3$

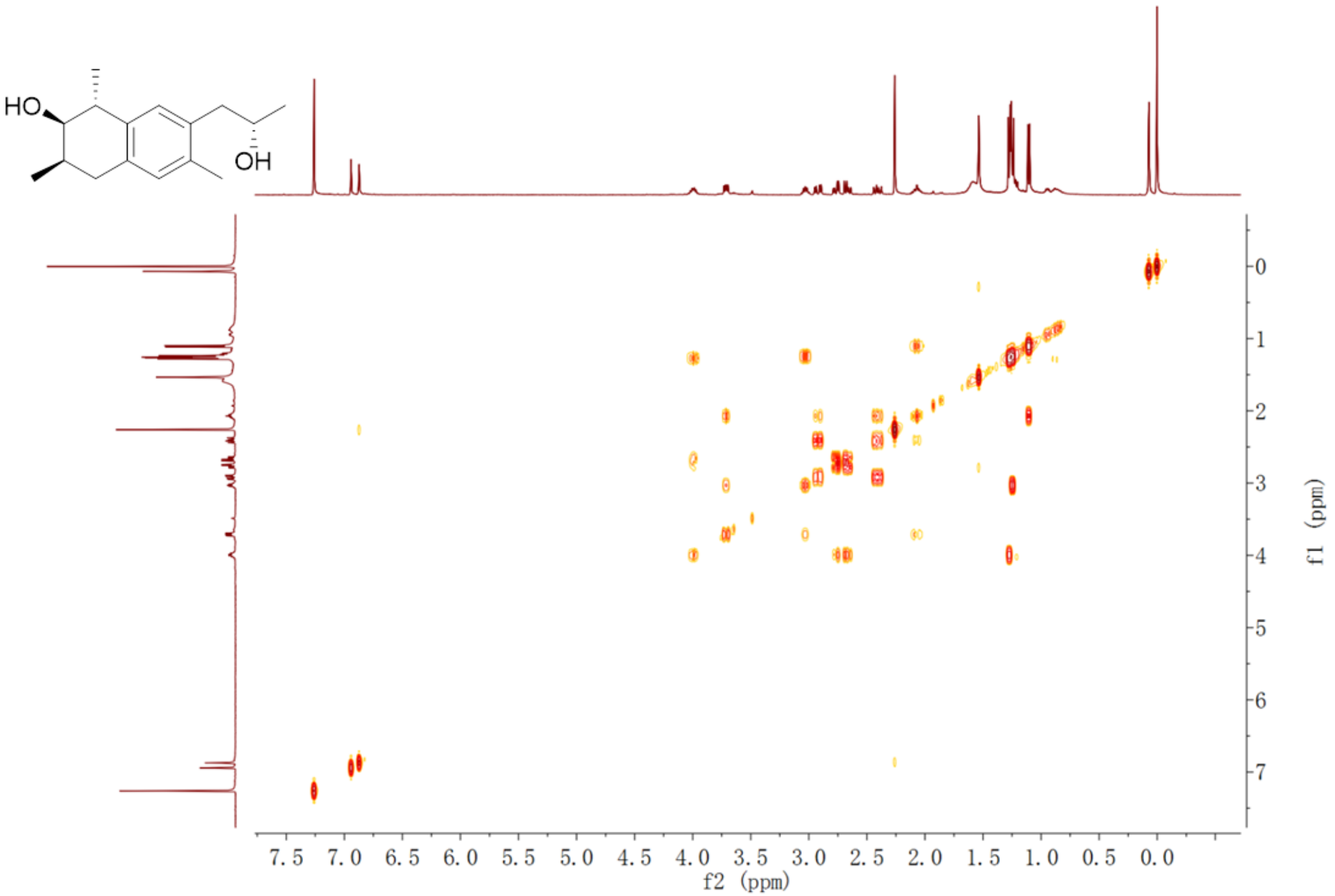

Figure S39. HMBC spectrum of dichocetide A (**5**) in $CDCl_3$

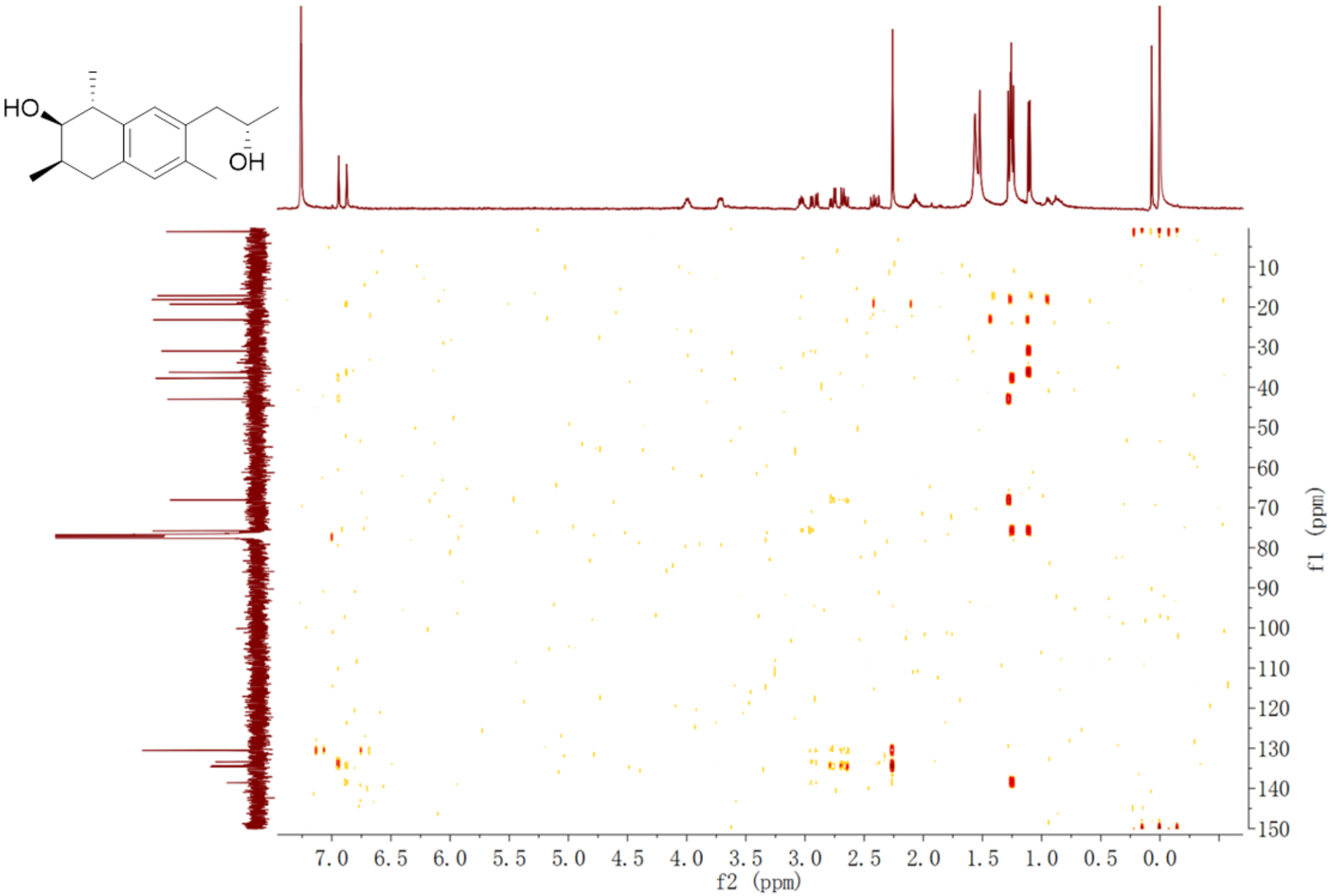

Figure S40. NOESY spectrum of dichocetide A (**5**) in $CDCl_3$

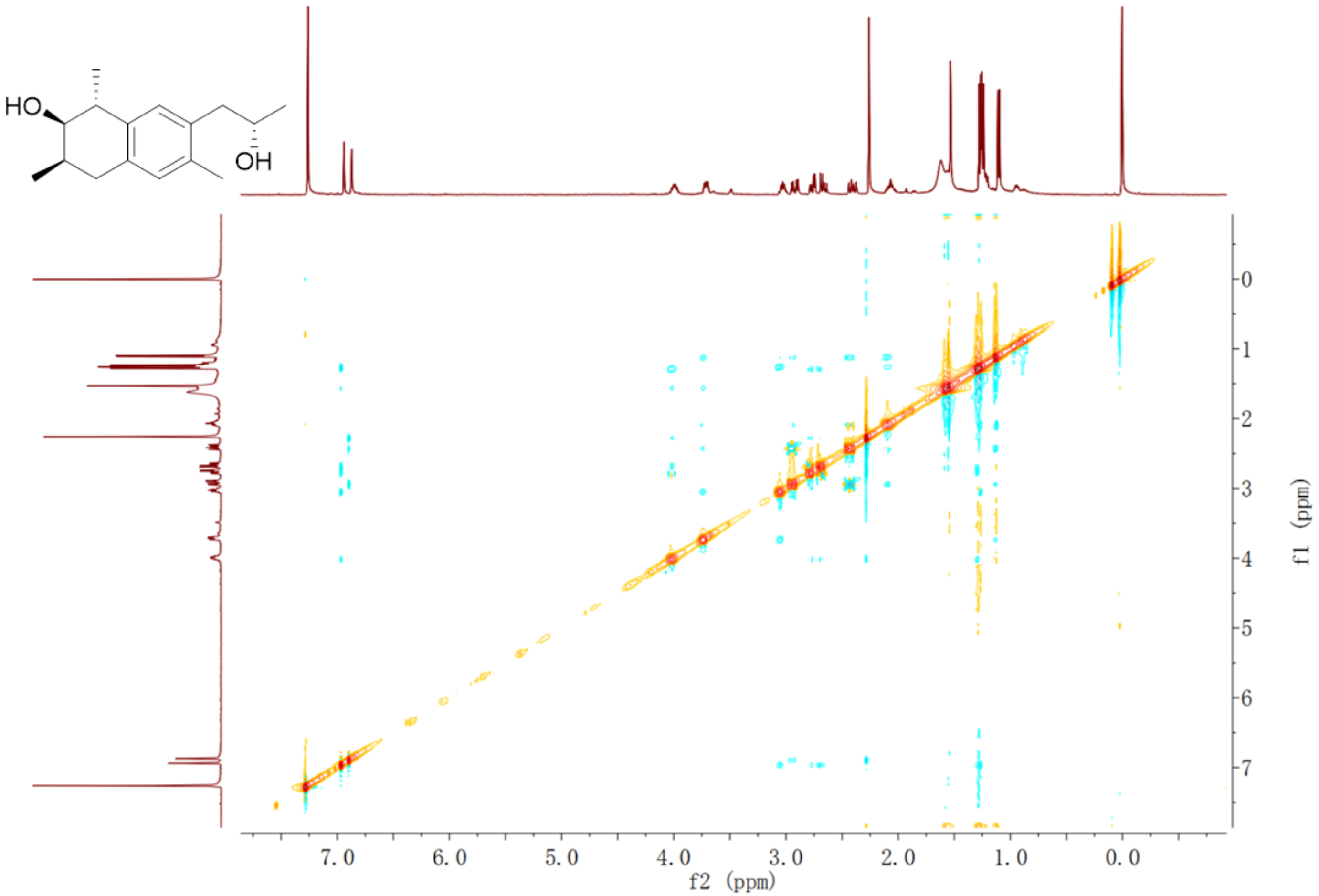

Figure S41. ^{1}H NMR spectrum of dichotone A (**6**) in $CDCl_3$ (400MHz)

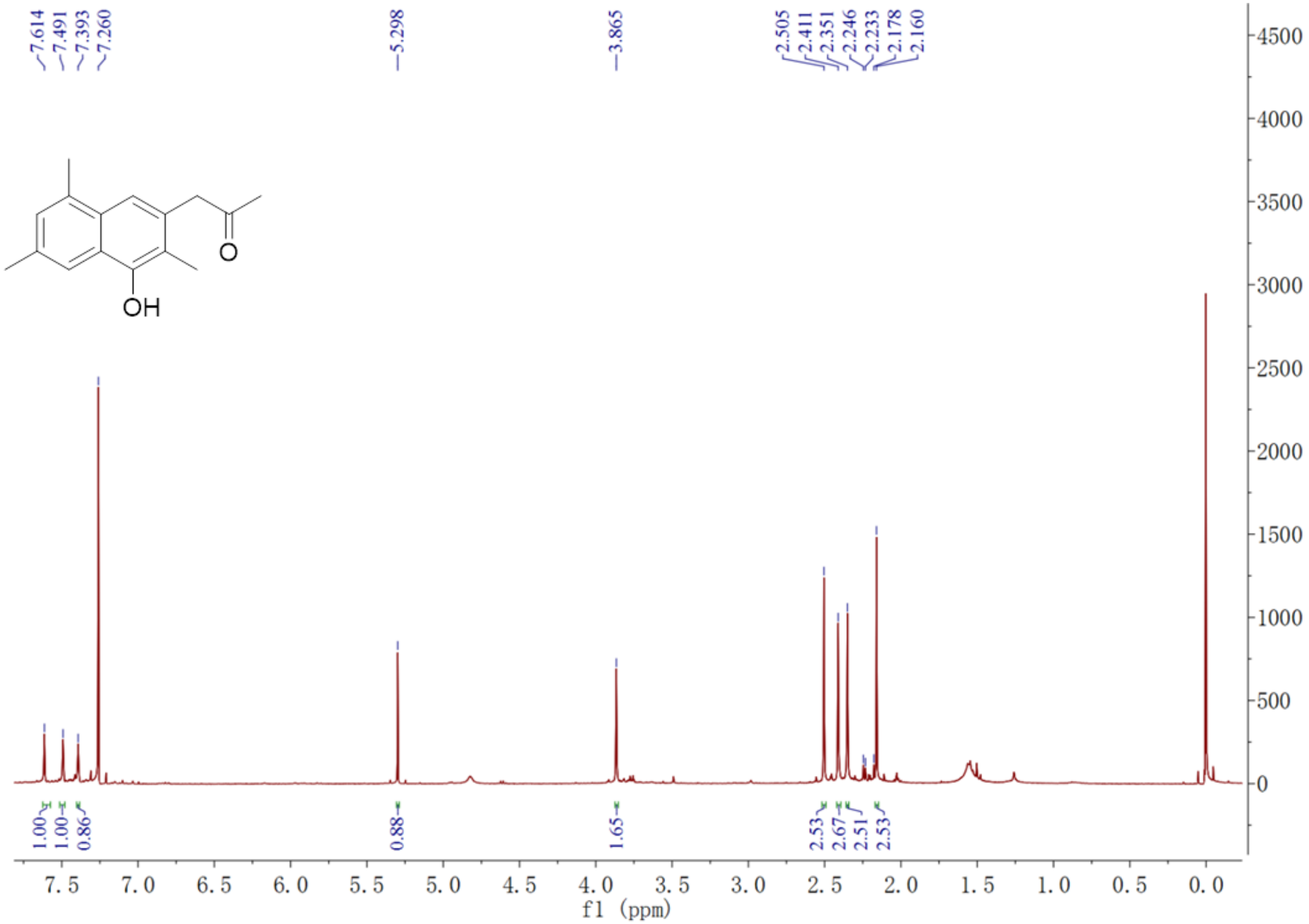

Figure S42. ^{13}C NMR spectrum of dichotone A (**6**) in $CDCl_3$ (100MHz)

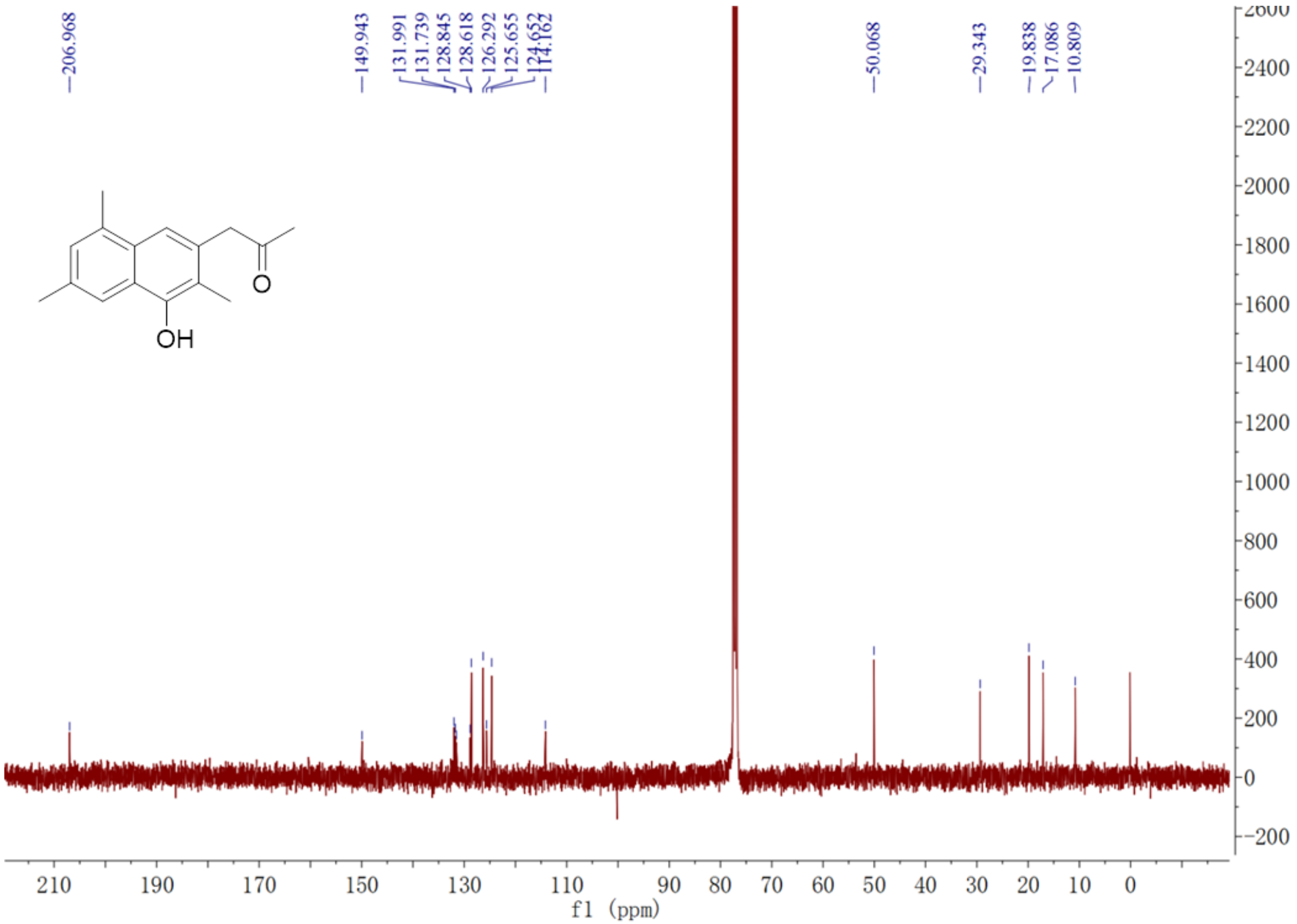

Figure S43. ^{1}H NMR spectrum of diorcinol (**7**) in $CDCl_3$ (400MHz)

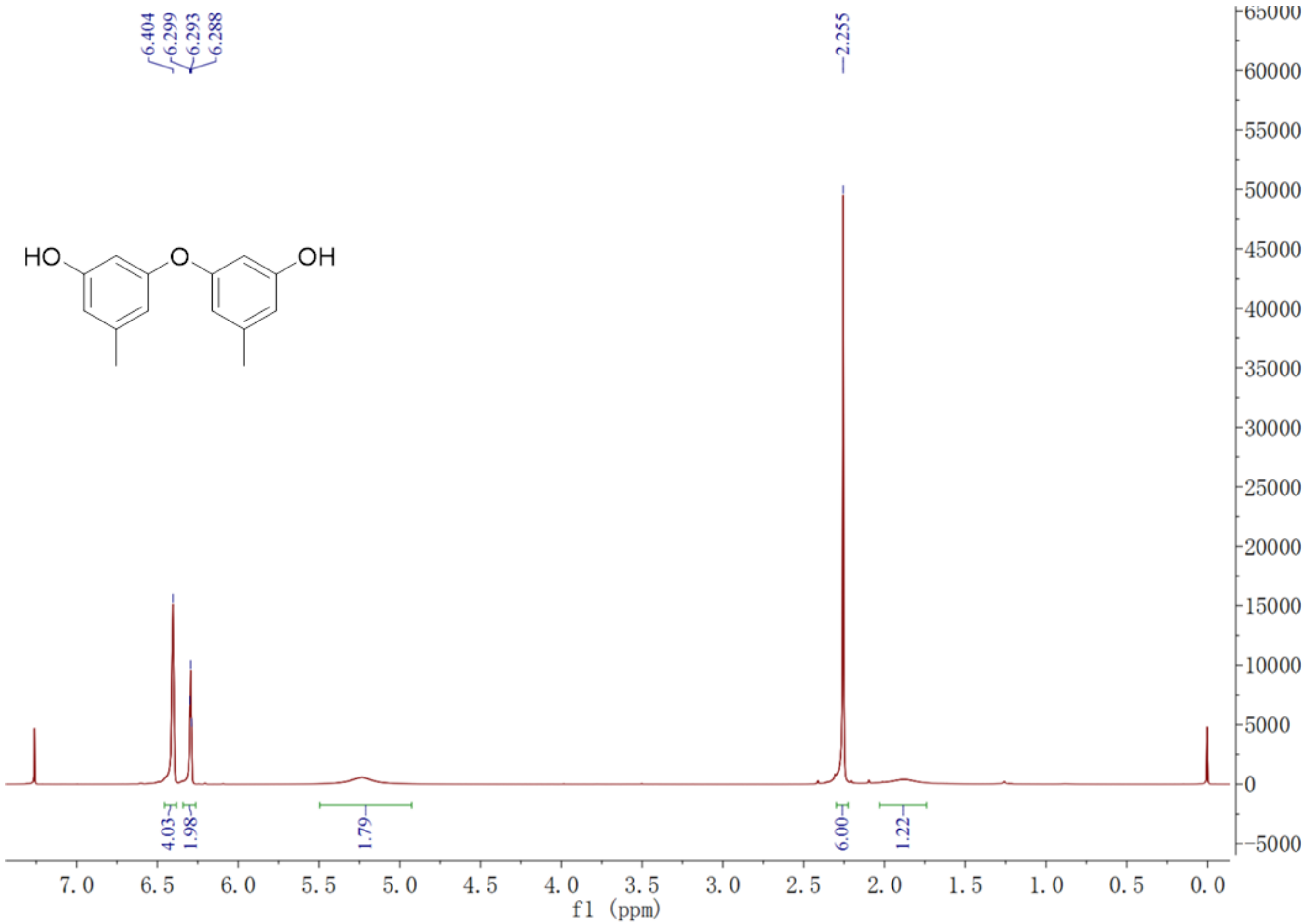

Figure S44. ^{13}C NMR spectrum of diorcinol (**7**) in $CDCl_3$ (100MHz)

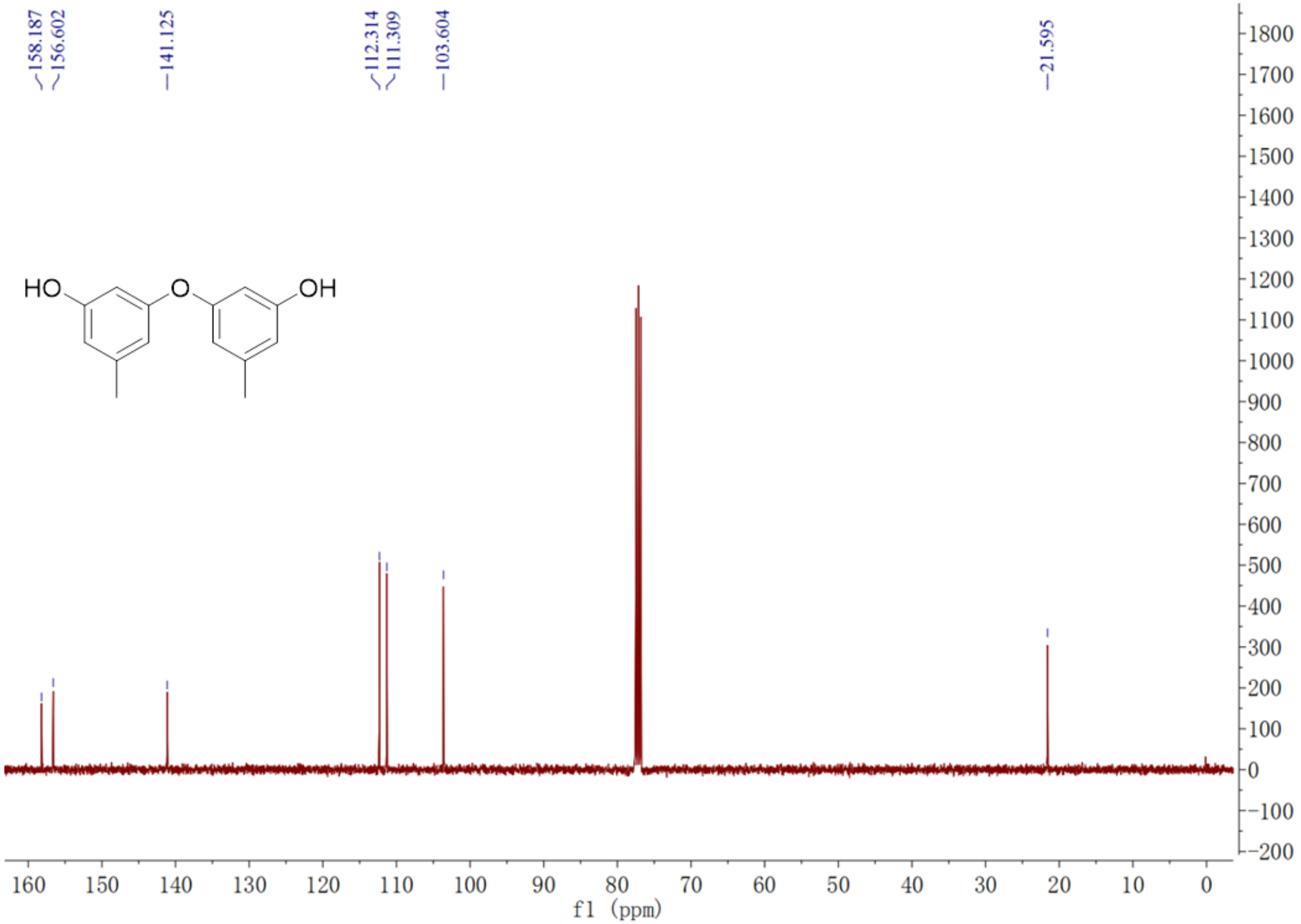

Figure S45. ^{1}H NMR spectrum of 3-O-methyldiorcinol (**8**) in $CDCl_3$ (400MHz)

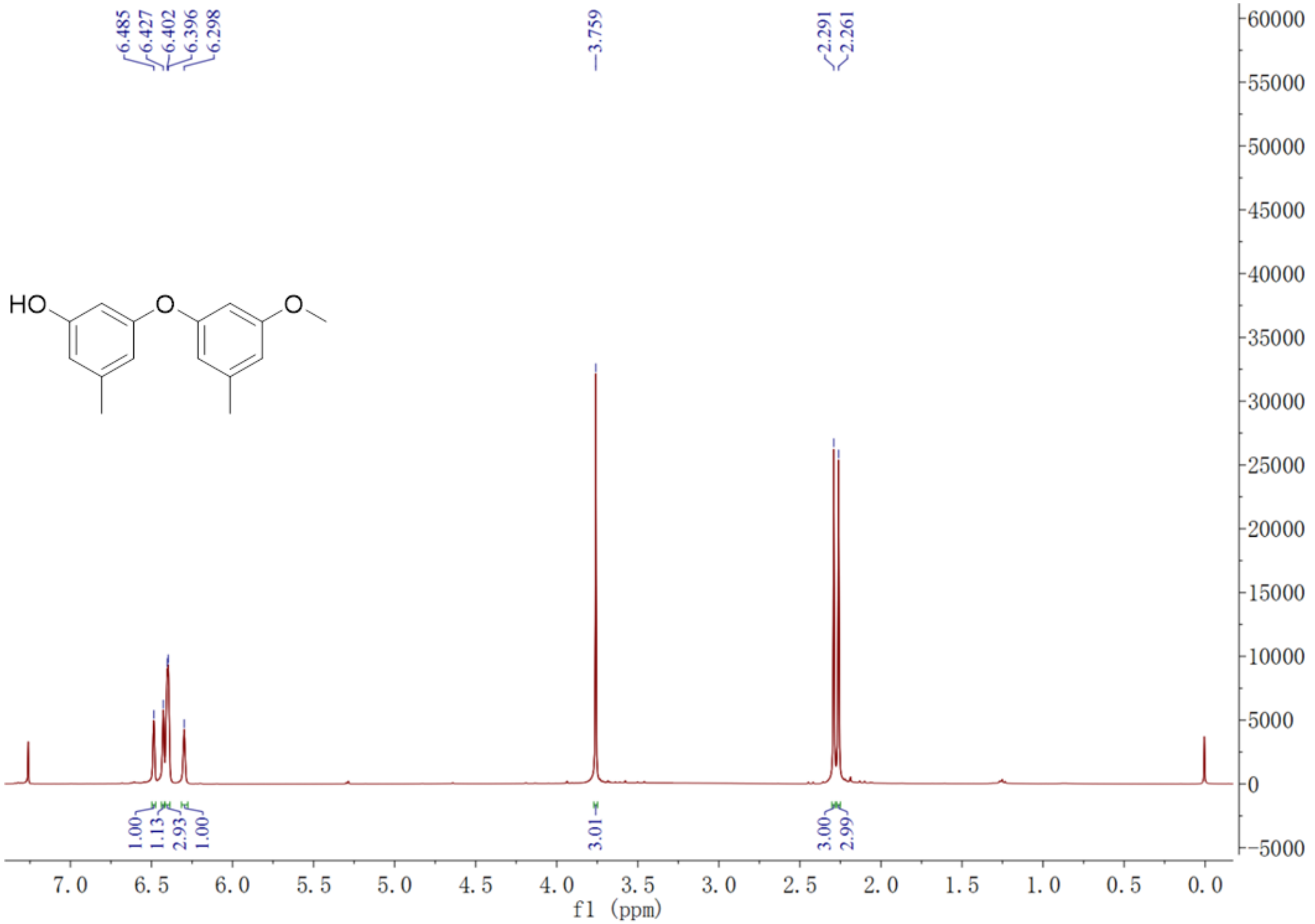

Figure S46. ^{13}C NMR spectrum of 3-O-methyldiorcinol (**8**) in $CDCl_3$ (100MHz)

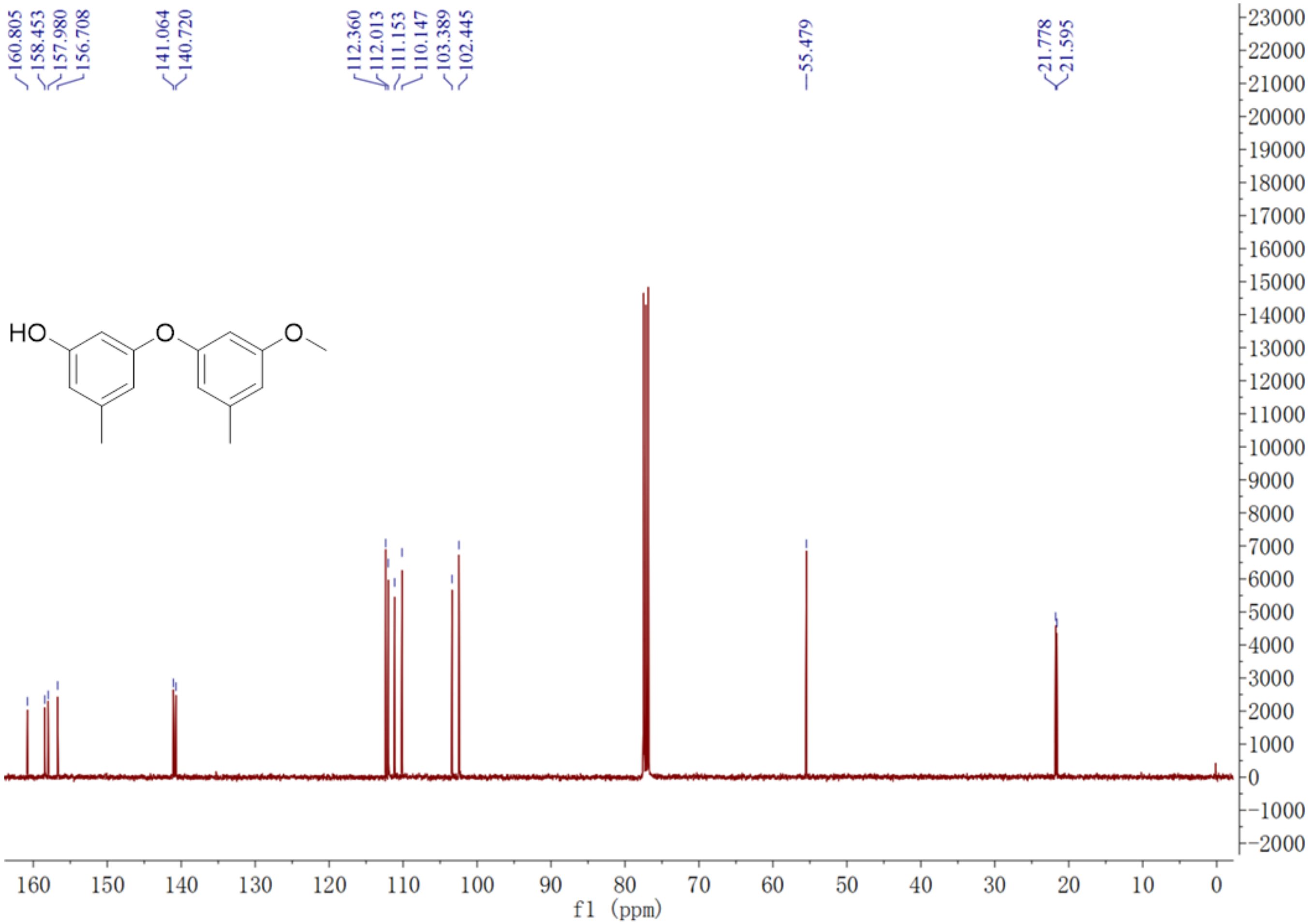

Figure.S47. ^{1}H NMR spectrum of 5,5'-oxybis(1-methoxy-3-methylbenzene) (**9**) in $CDCl_3$ (400MHz)

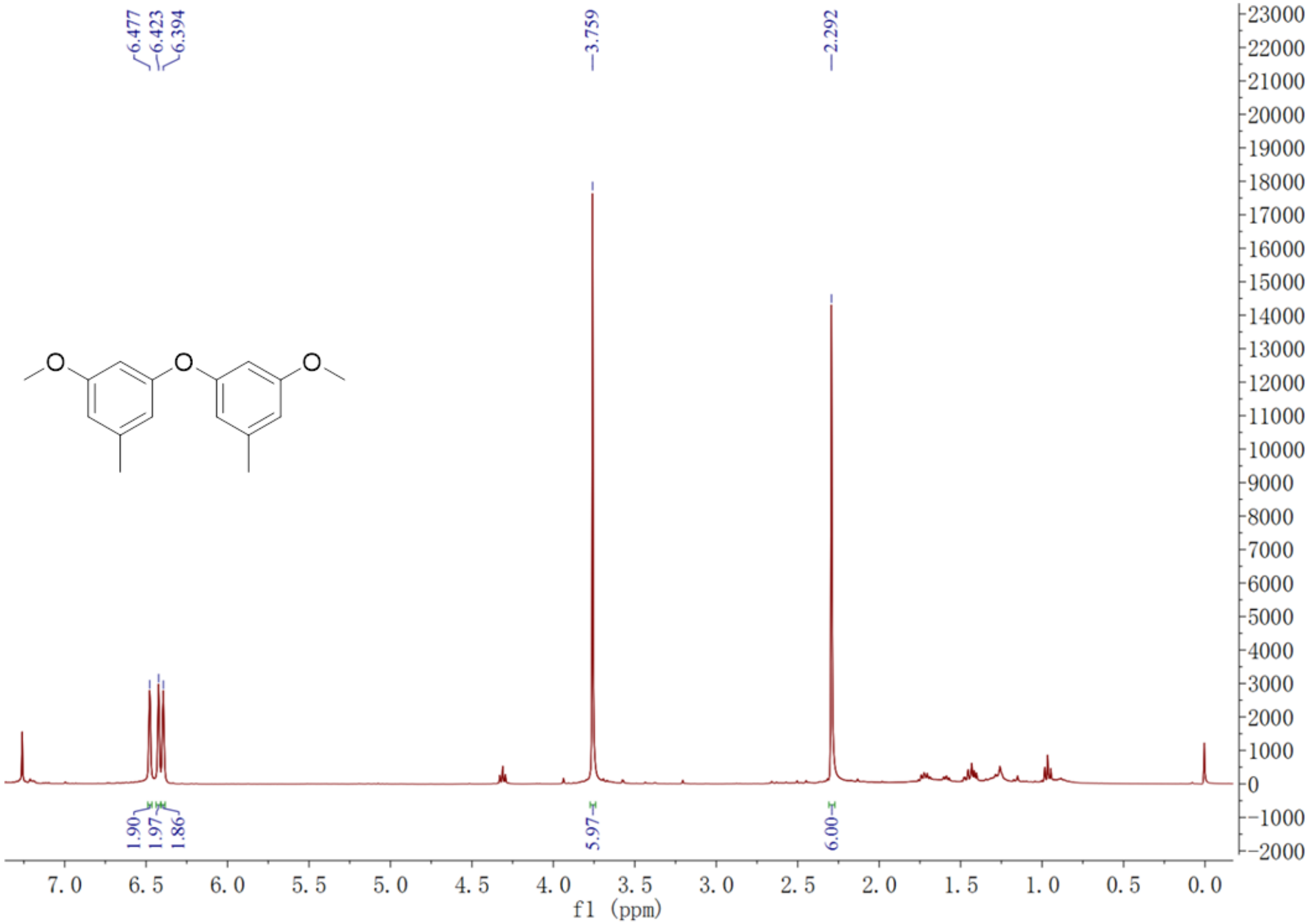

Figure S48. ^{13}C NMR spectrum of 5,5'-oxybis(1-methoxy-3-methylbenzene) (**9**) in $CDCl_3$ (100MHz)

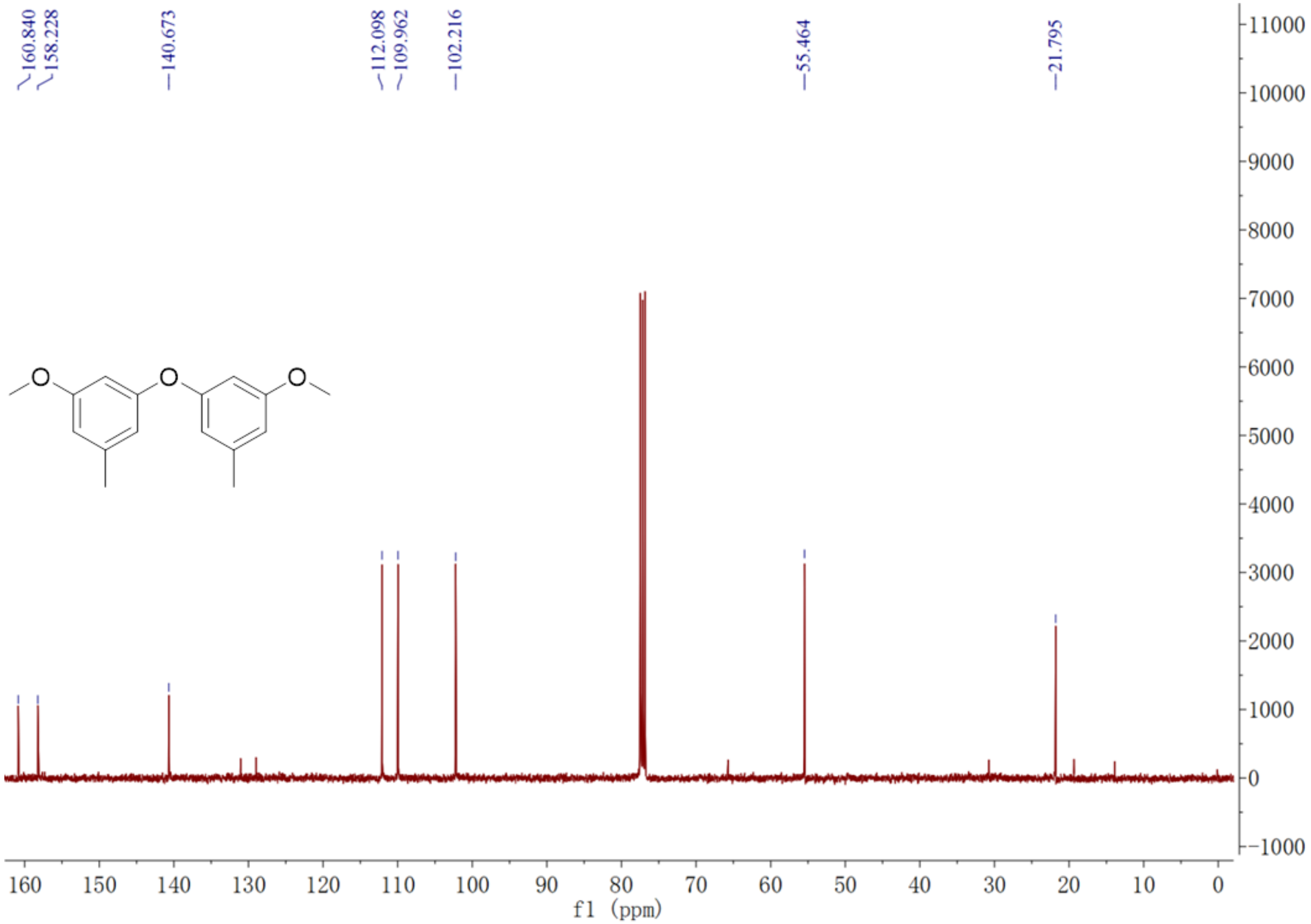

Figure S49. ^{1}H NMR spectrum of dibutyl phthalate (**10**) in $CDCl_3$ (400MHz)

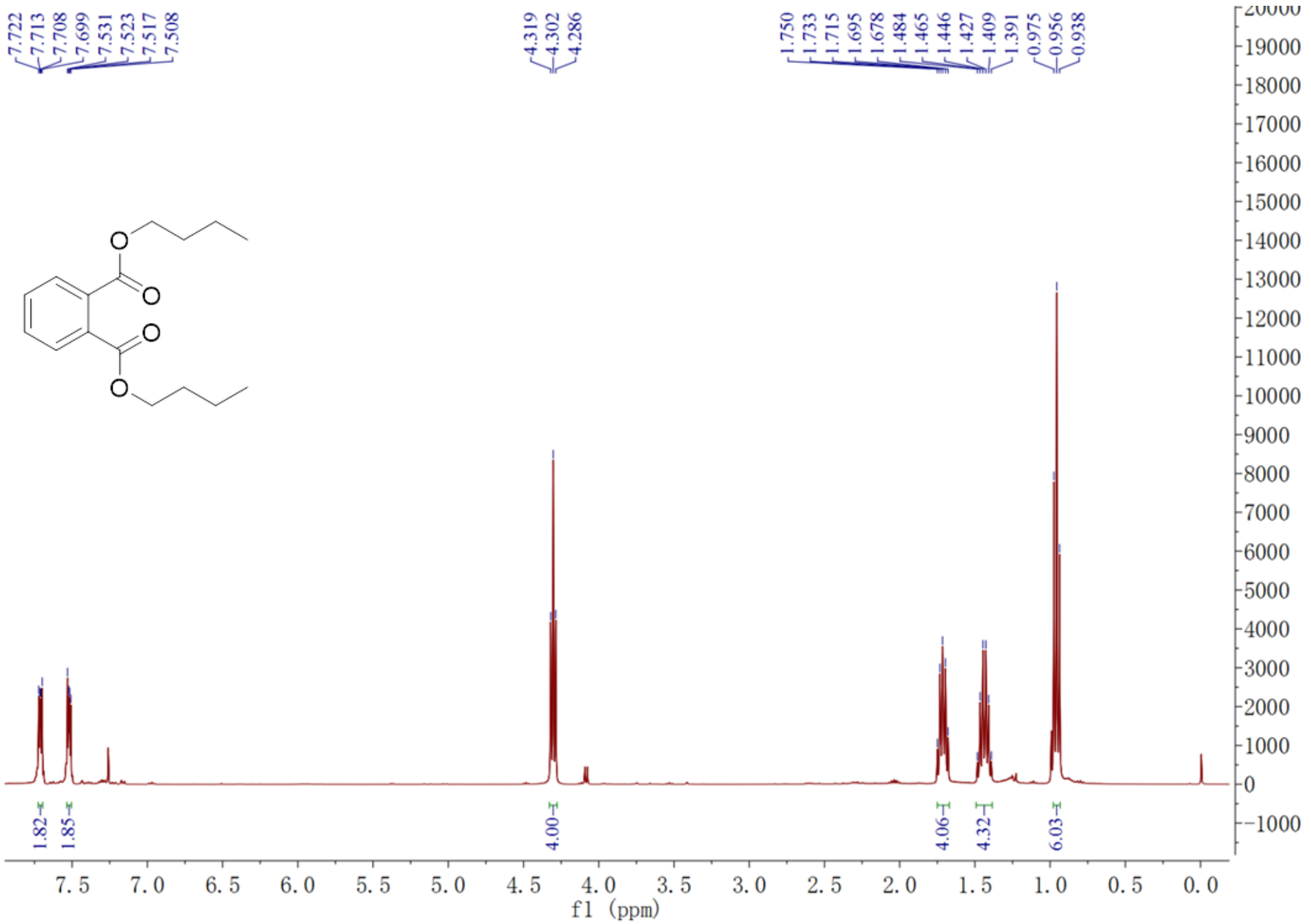

Figure S50. ^{13}C NMR spectrum of dibutyl phthalate (**10)** in $CDCl_3$ (100MHz)

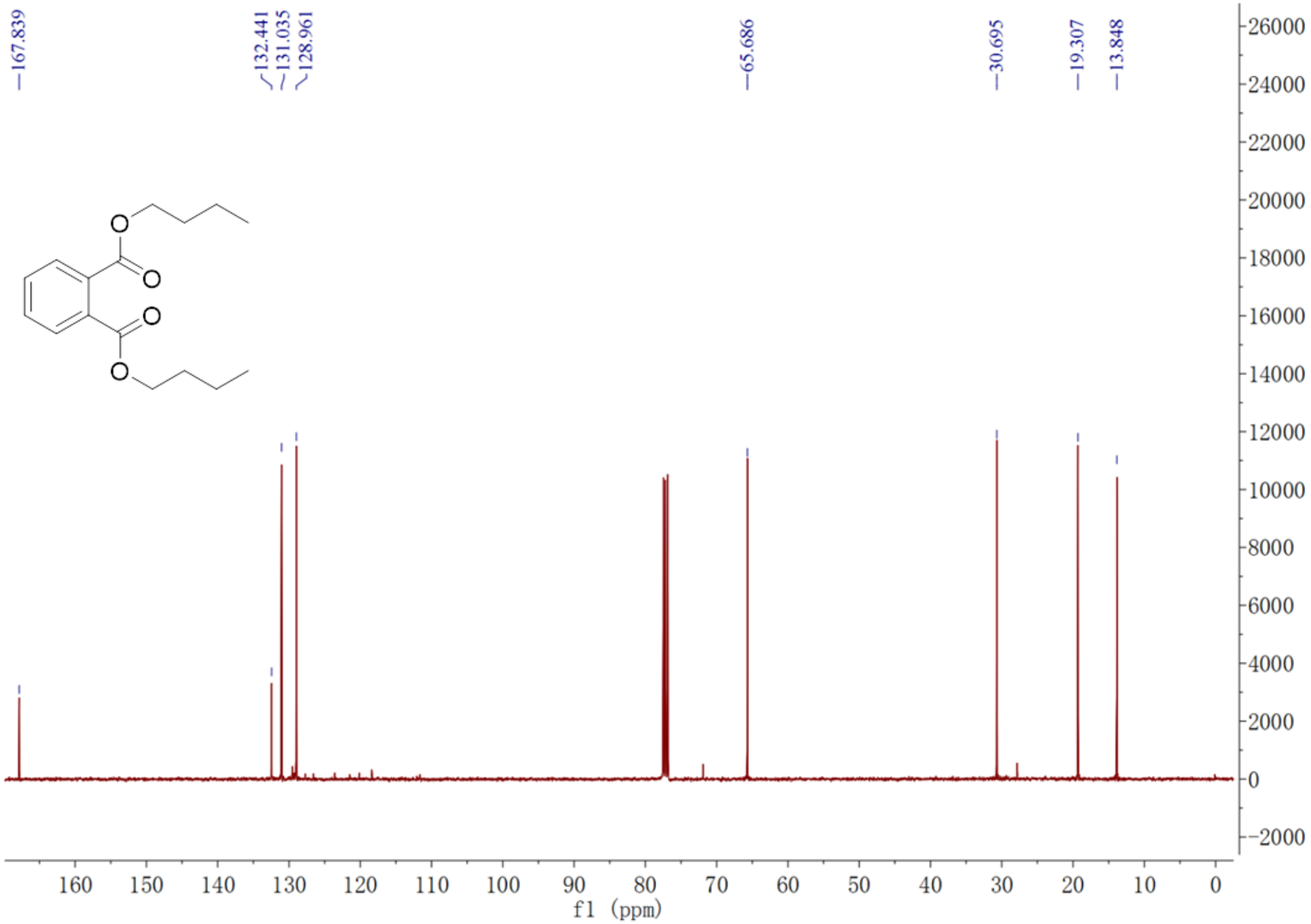

Figure S51. ^{1}H NMR spectrum of (2-ethylhexyl) phthalate (**11**) in $CDCl_3$ (400MHz)

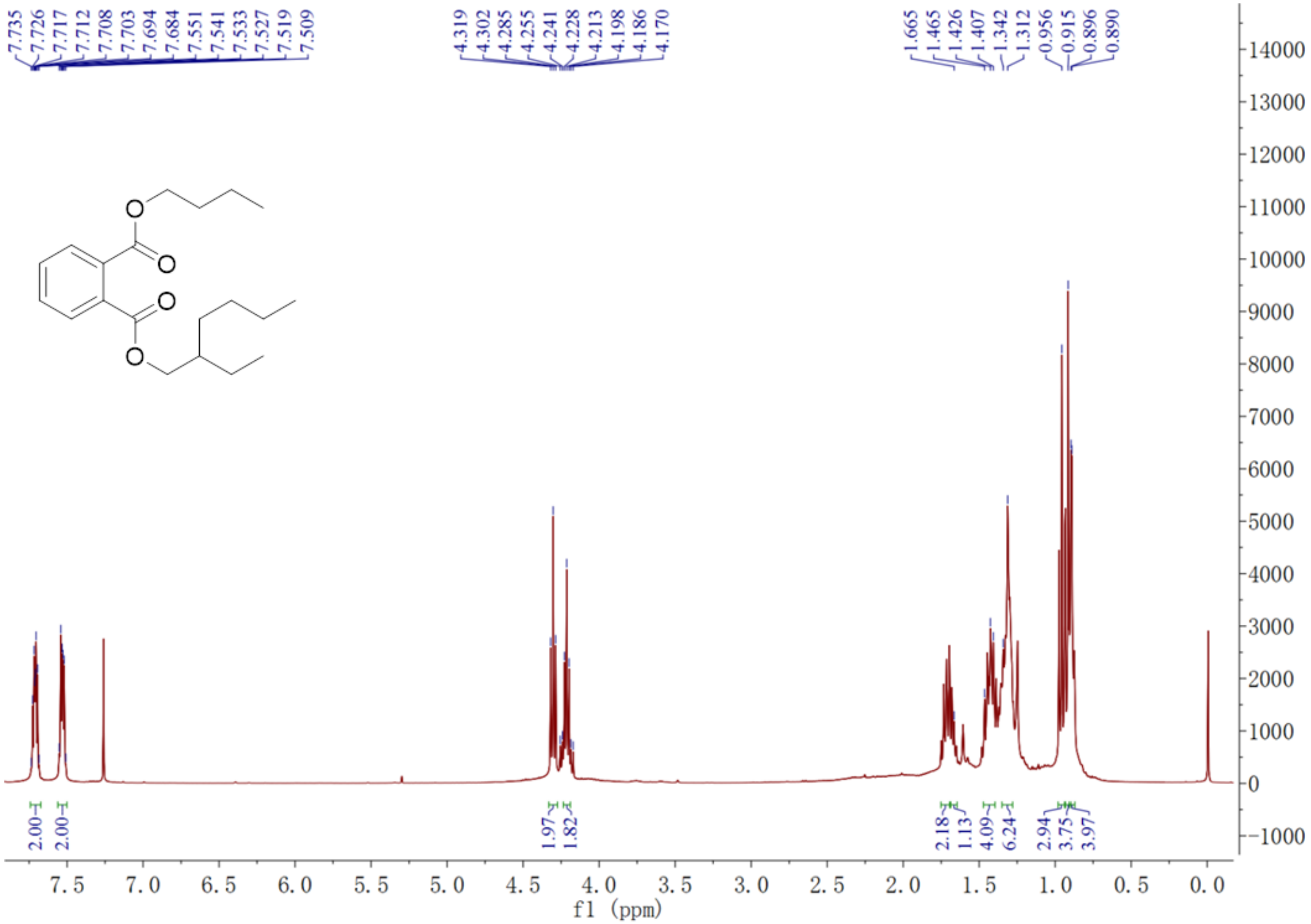

Figure S52. ^{13}C NMR spectrum of (2-ethylhexyl) phthalate (**11**) in $CDCl_3$ (100MHz)

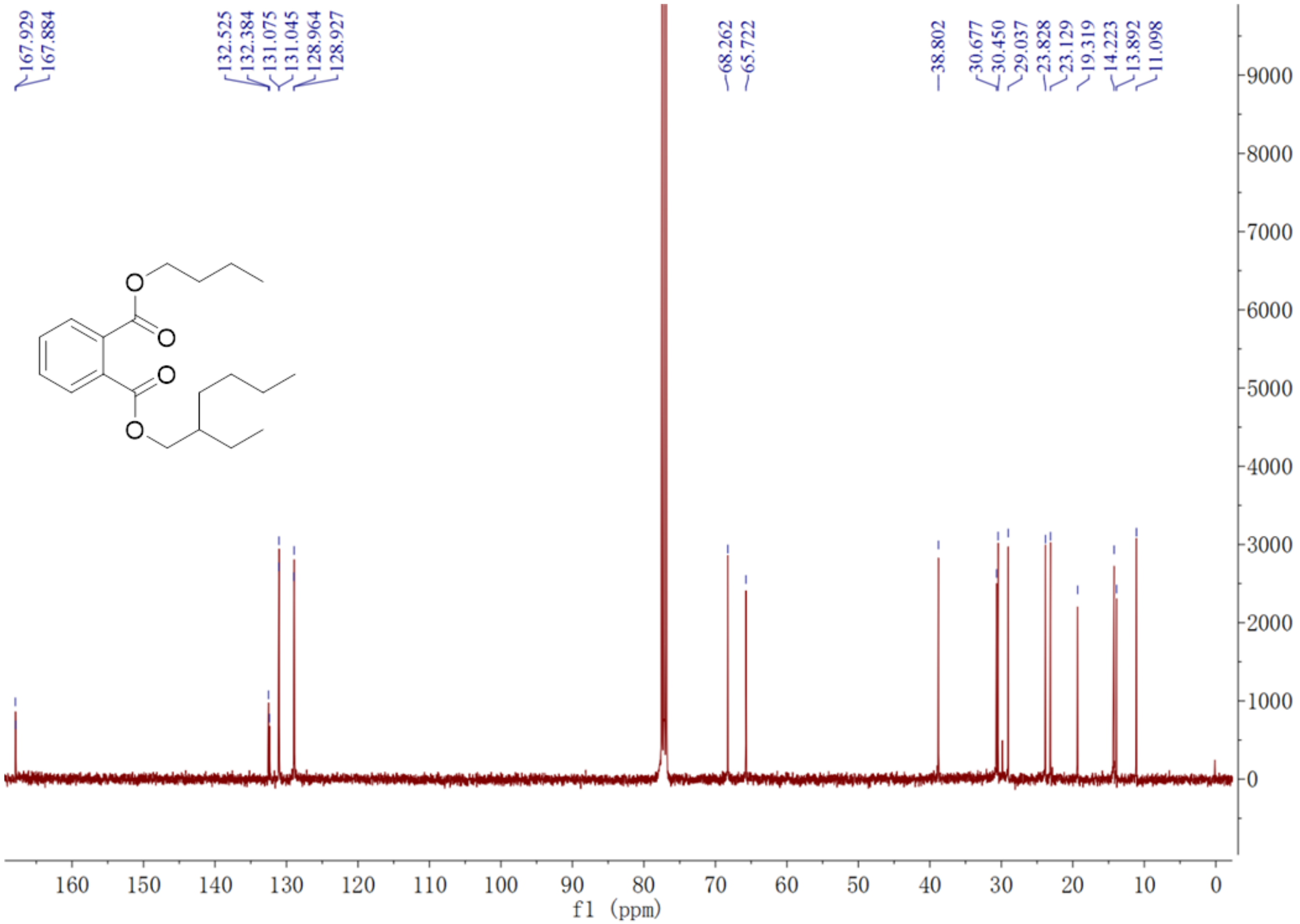

Figure S53. ^{1}H NMR spectrum of (2a*R*,5*R*,5a*R*,8*S*,8a*S*)-2,2,5,8-tetramethyldecahydro-2H-naphtho[1,8-bc]furan-5-ol (**12**) in $CDCl_3$ (400MHz)

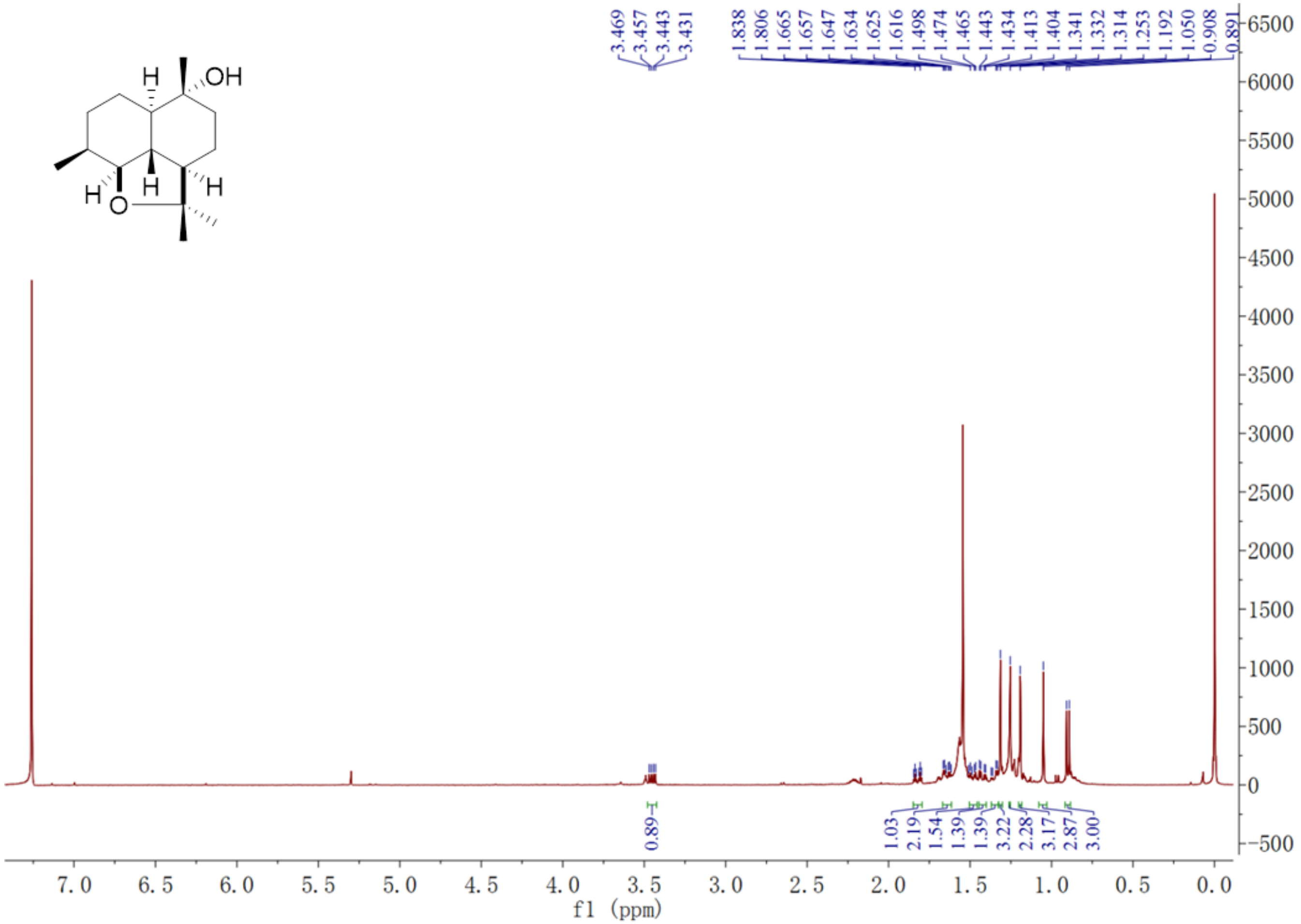

Figure S54. ^{13}C NMR spectrum of (2a*R*,5*R*,5a*R*,8*S*,8a*S*)-2,2,5,8-tetramethyldecahydro-2H-naphtho[1,8-bc]furan-5-ol (**12**) in $CDCl_3$ (100MHz)

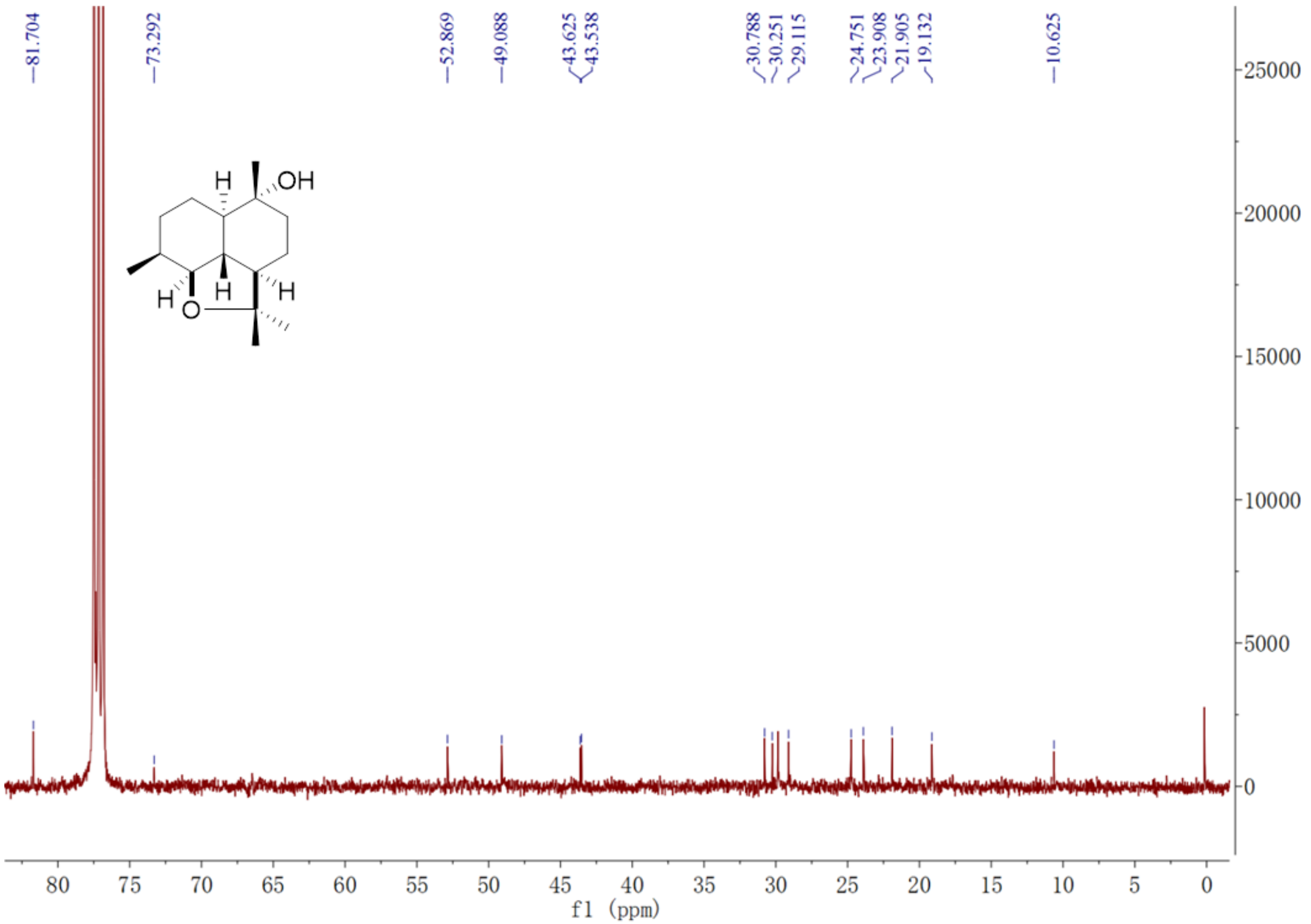

Figure S55 ^{1}H NMR spectrum of aspewentin A (**13**) in $CDCl_3$ (400MHz)

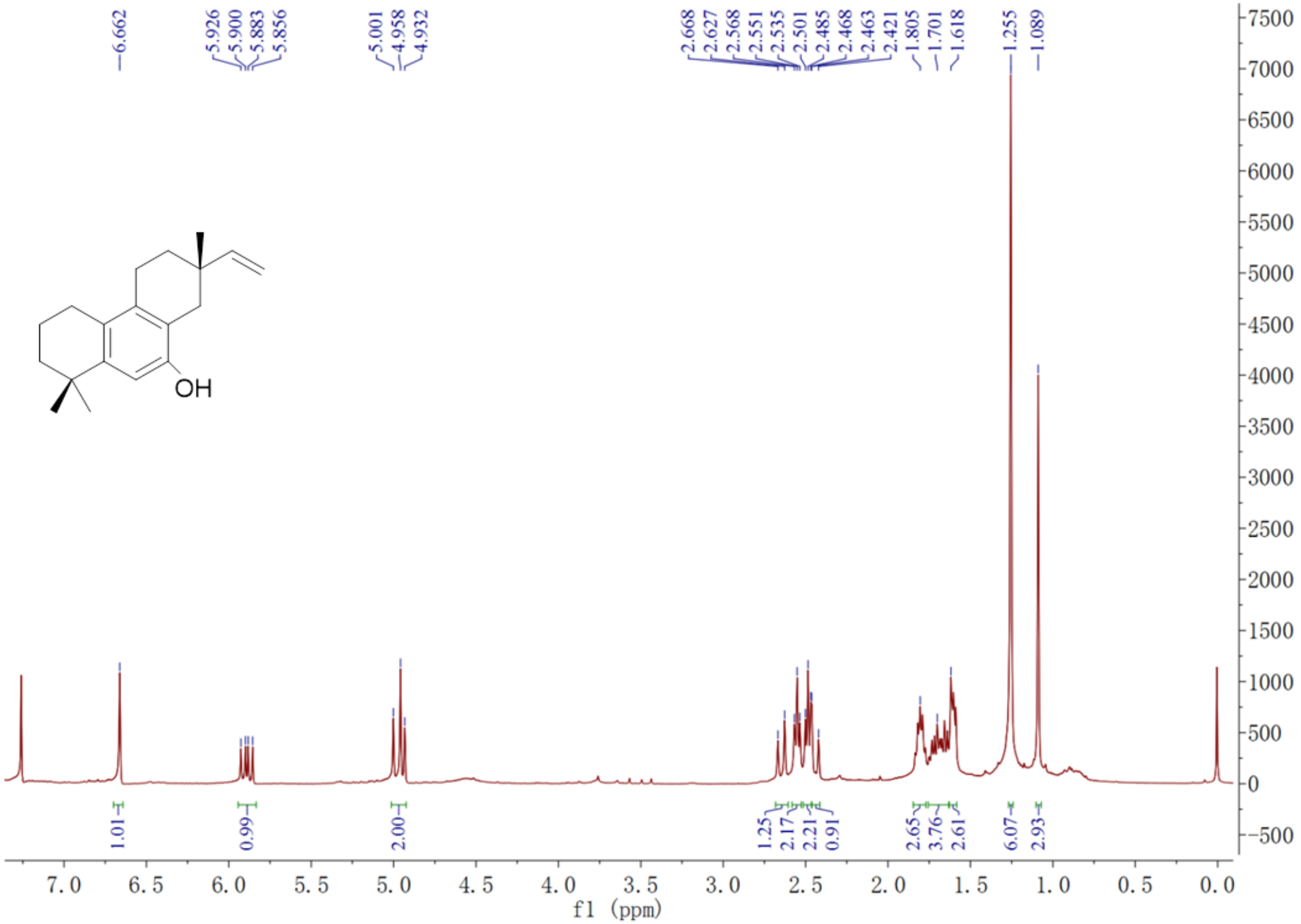

Figure S56. ^{13}C NMR spectrum of aspewentin A (**13**) in $CDCl_3$ (100MHz)

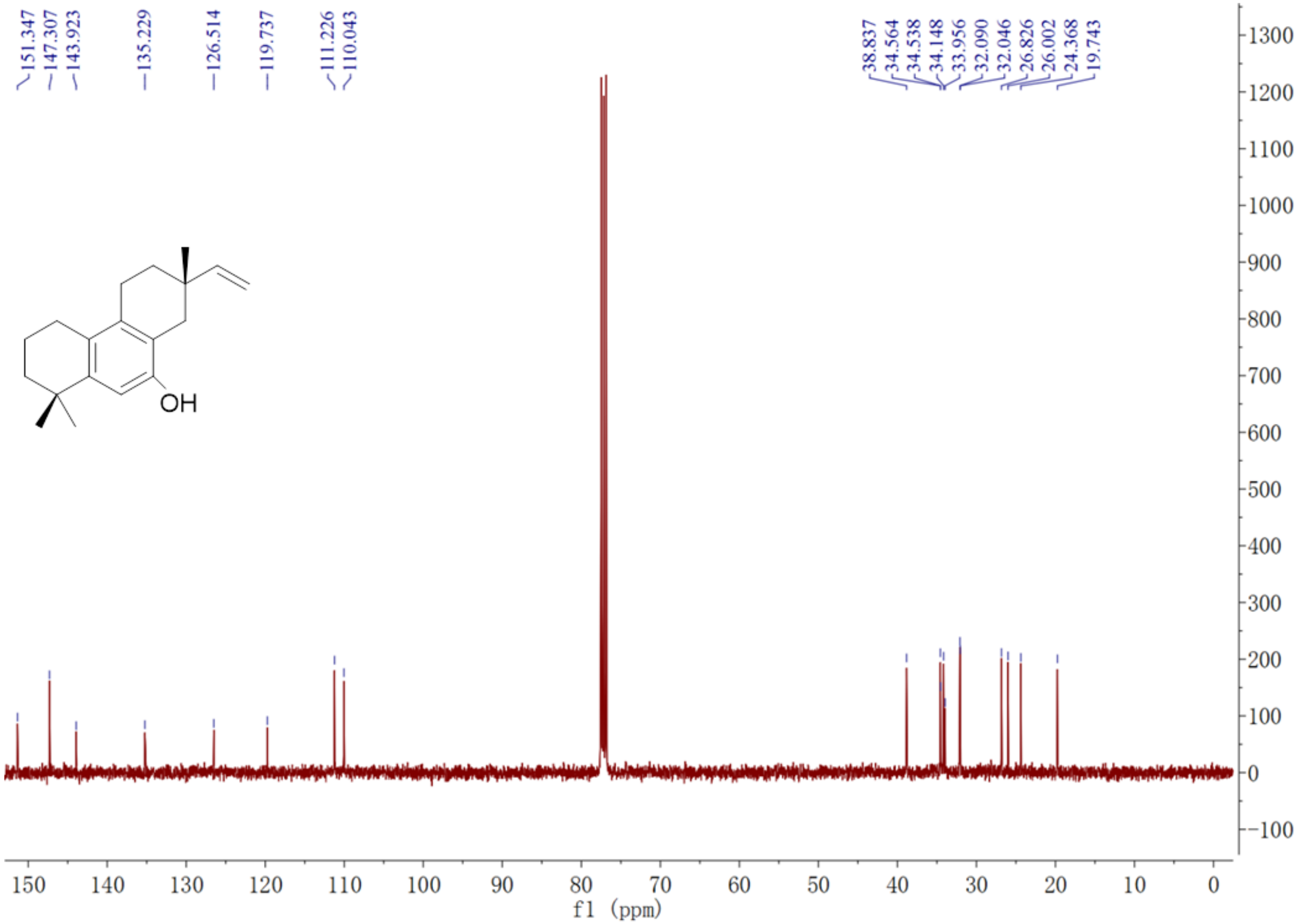

Figure S57. ^{1}H NMR spectrum of JBIR-03 (**14**) in $CDCl_3$ (400MHz)

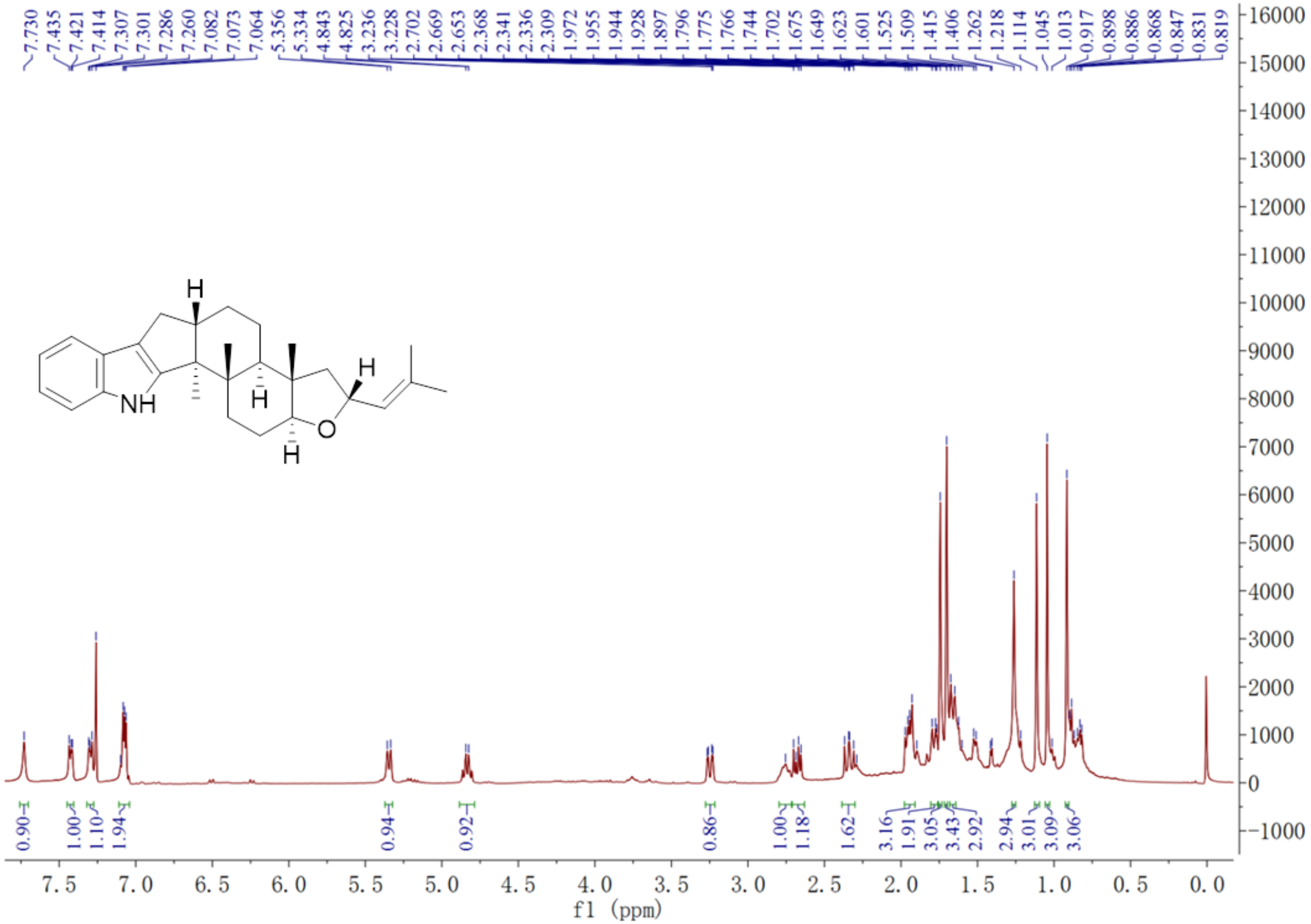

Figure S58. ^{13}C NMR spectrum of JBIR-03 (**14**) in $CDCl_3$ (100MHz)

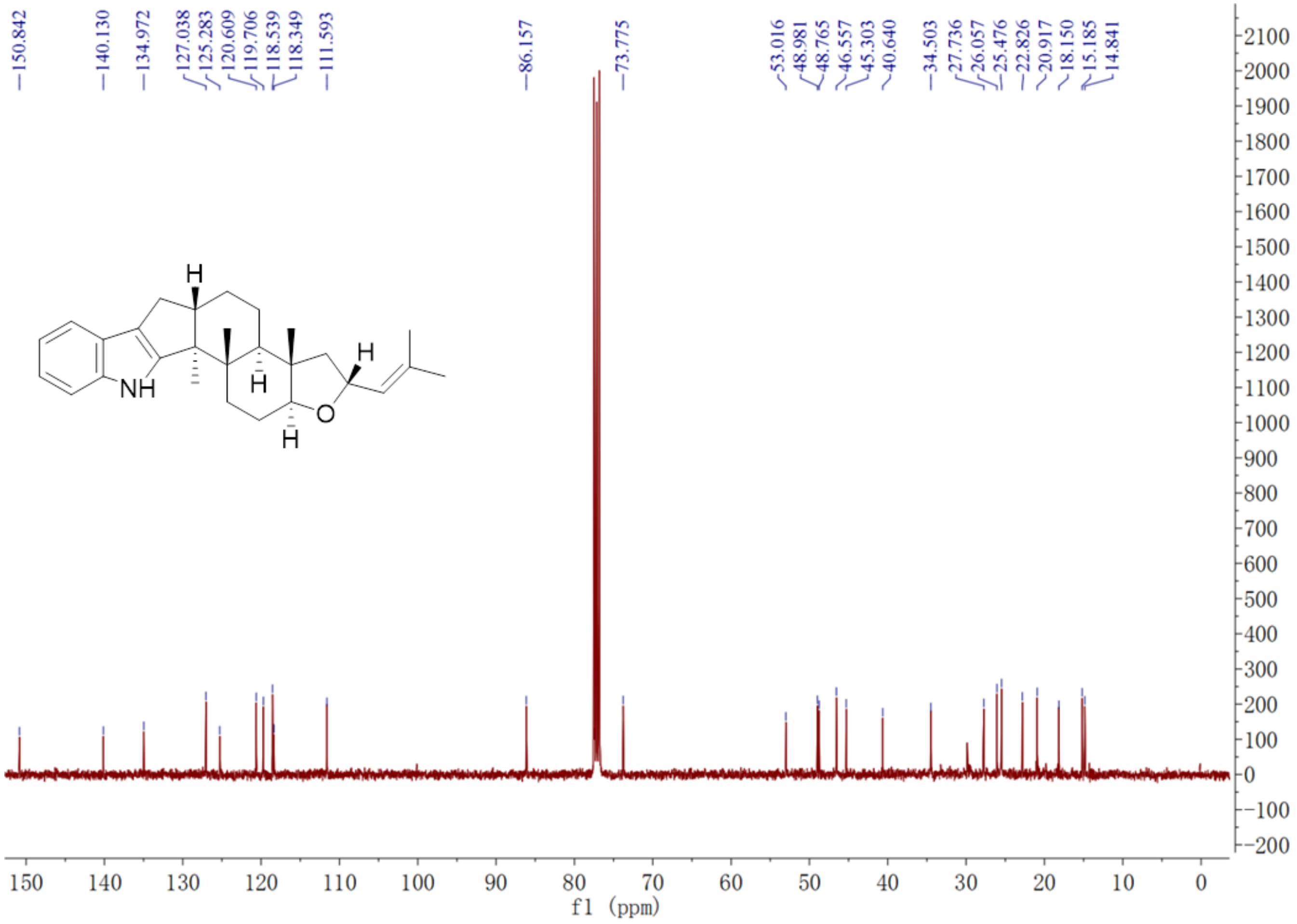

Figure S59. HR-ESI-MS spectrum of dichocerazine A (**15**)

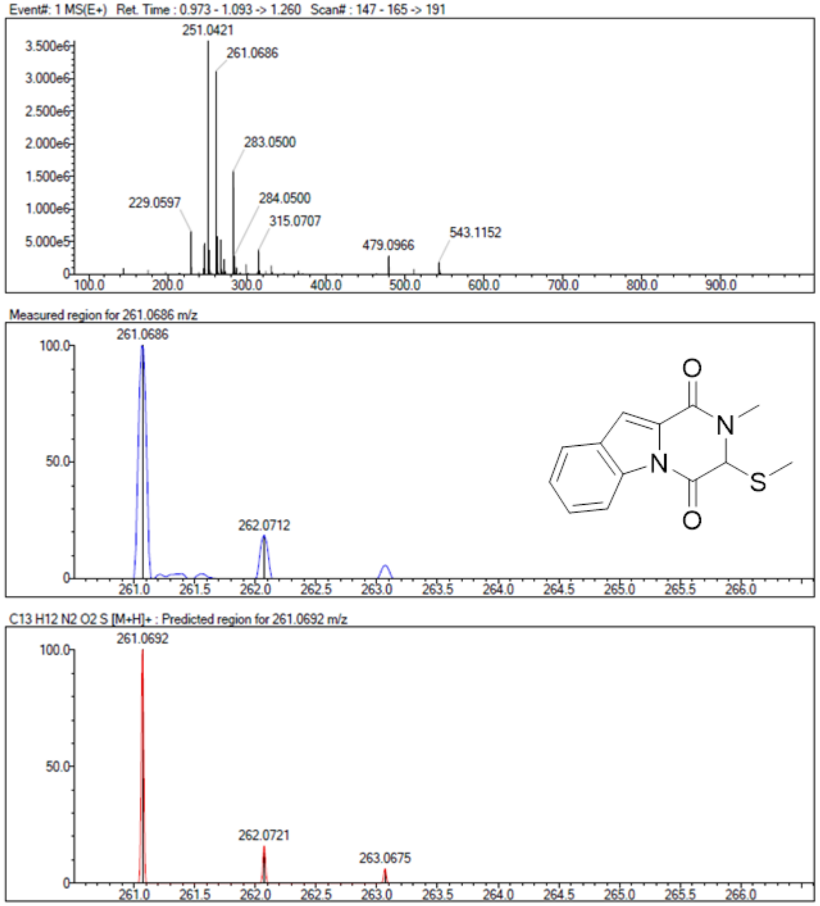

Rank	Score	Formula (M)	Ion	Meas. m/z	Pred. m/z	Df. (mDa)	Df. (ppm)	Iso	DBE
2	68.49	C13 H12 N2 O2 S	[M+H]+	261.0686	261.0692	-0.6	-2.30	70.79	9.0

Figure S60. ^{1}H NMR spectrum of dichocerazine A (**15**) in CDCl3 (400MHz)

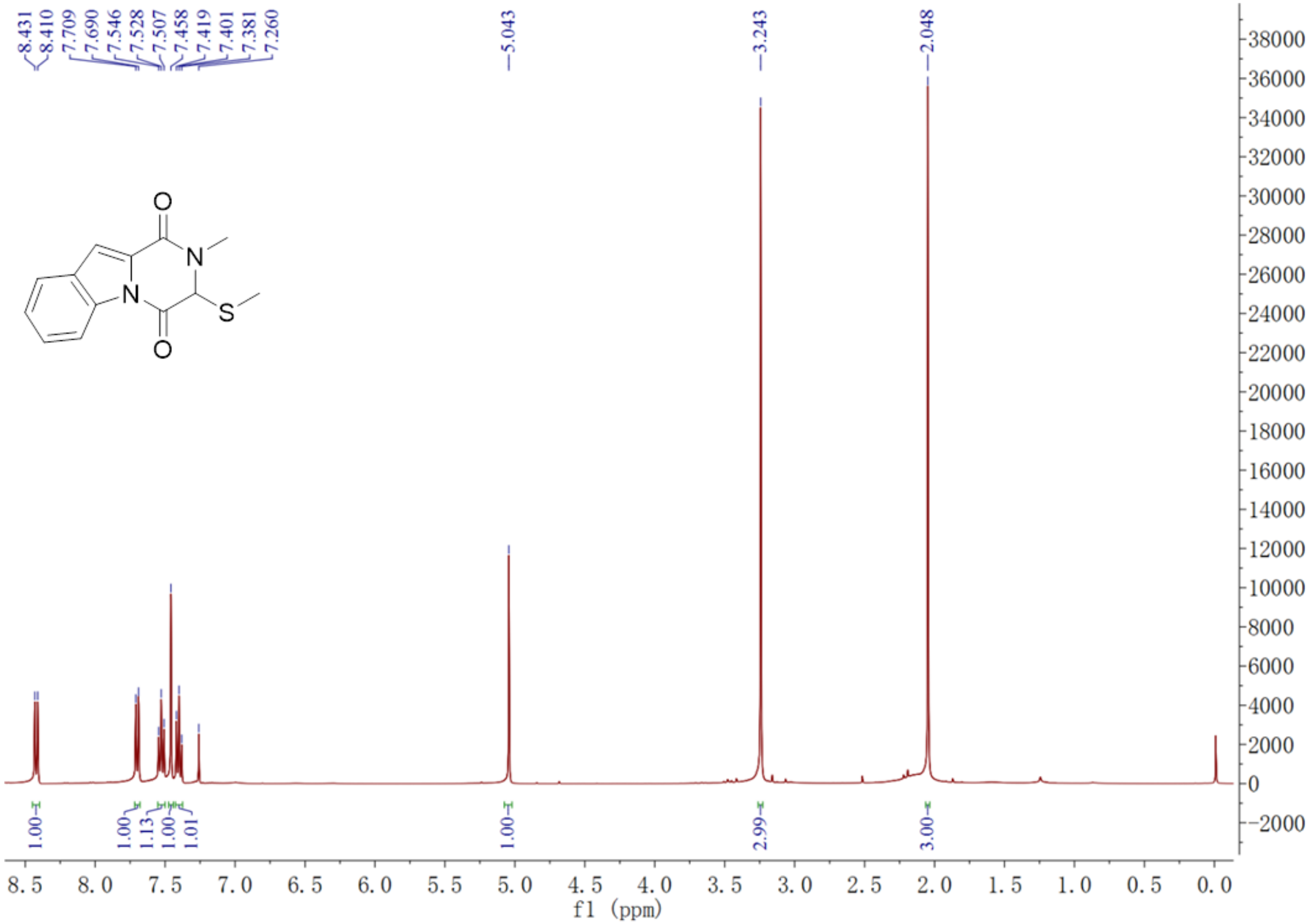

Figure S61. ^{13}C NMR spectrum of dichocerazine A (**15**) in CDCl3 (100MHz)

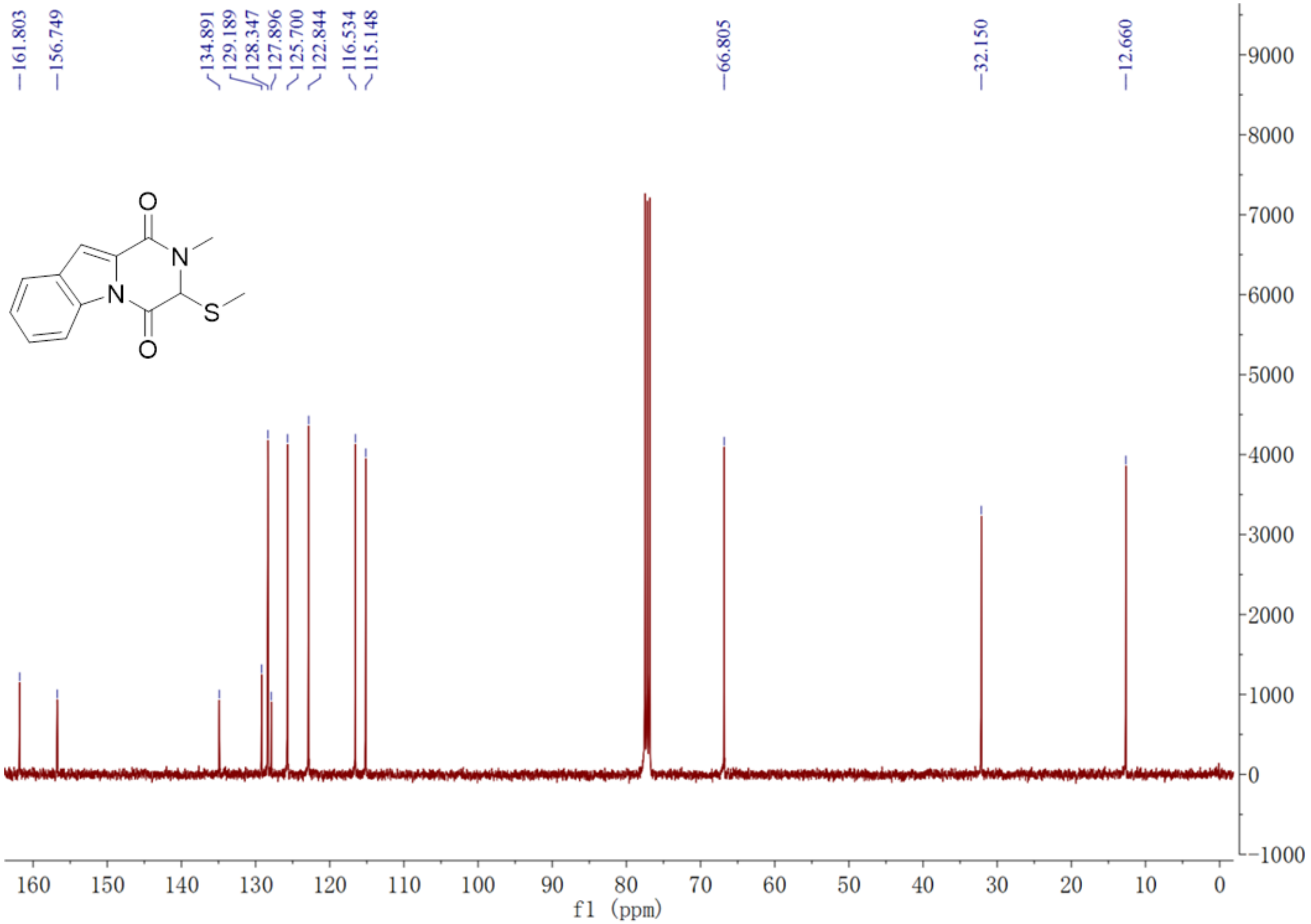

Figure S62. DEPT 135 spectrum of dichocerazine A (**15**) in CDCl3 (100MHz)

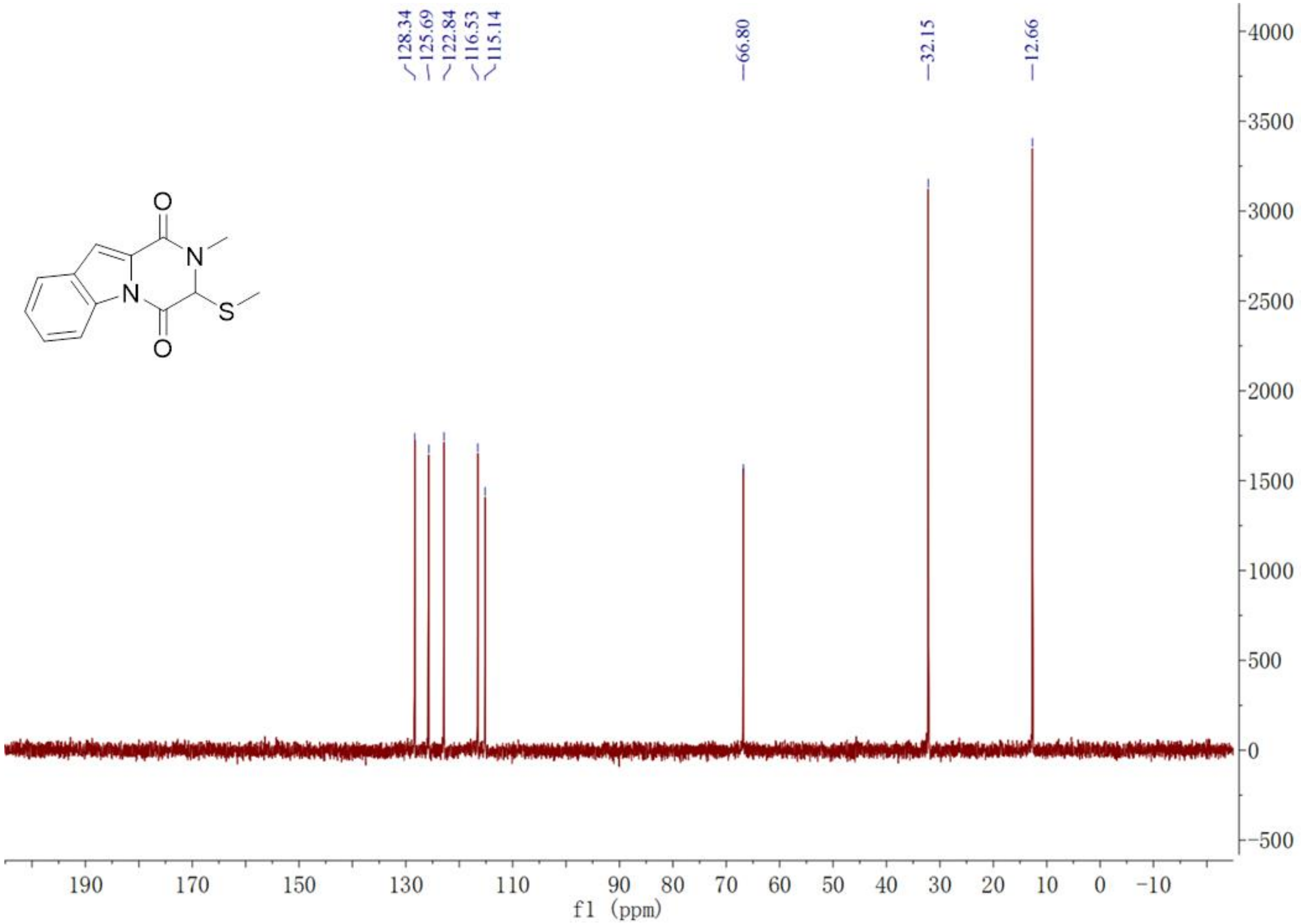

Figure S63. HMQC spectrum of dichocerazine A (**15**) in CDCl3

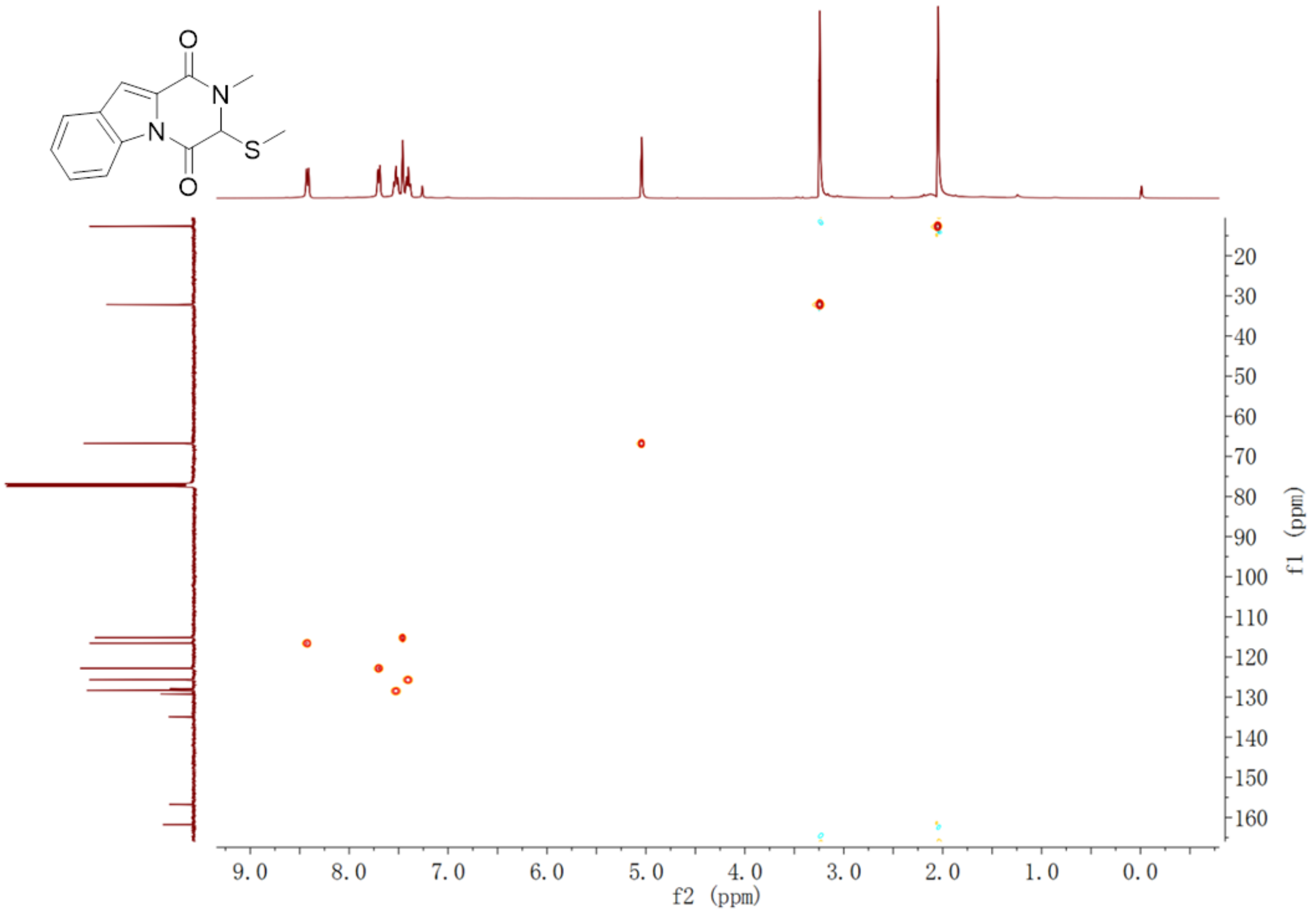

Figure S64. ^{1}H-^{1}H COSY spectrum of dichocerazine A (**15**) in CDCl3

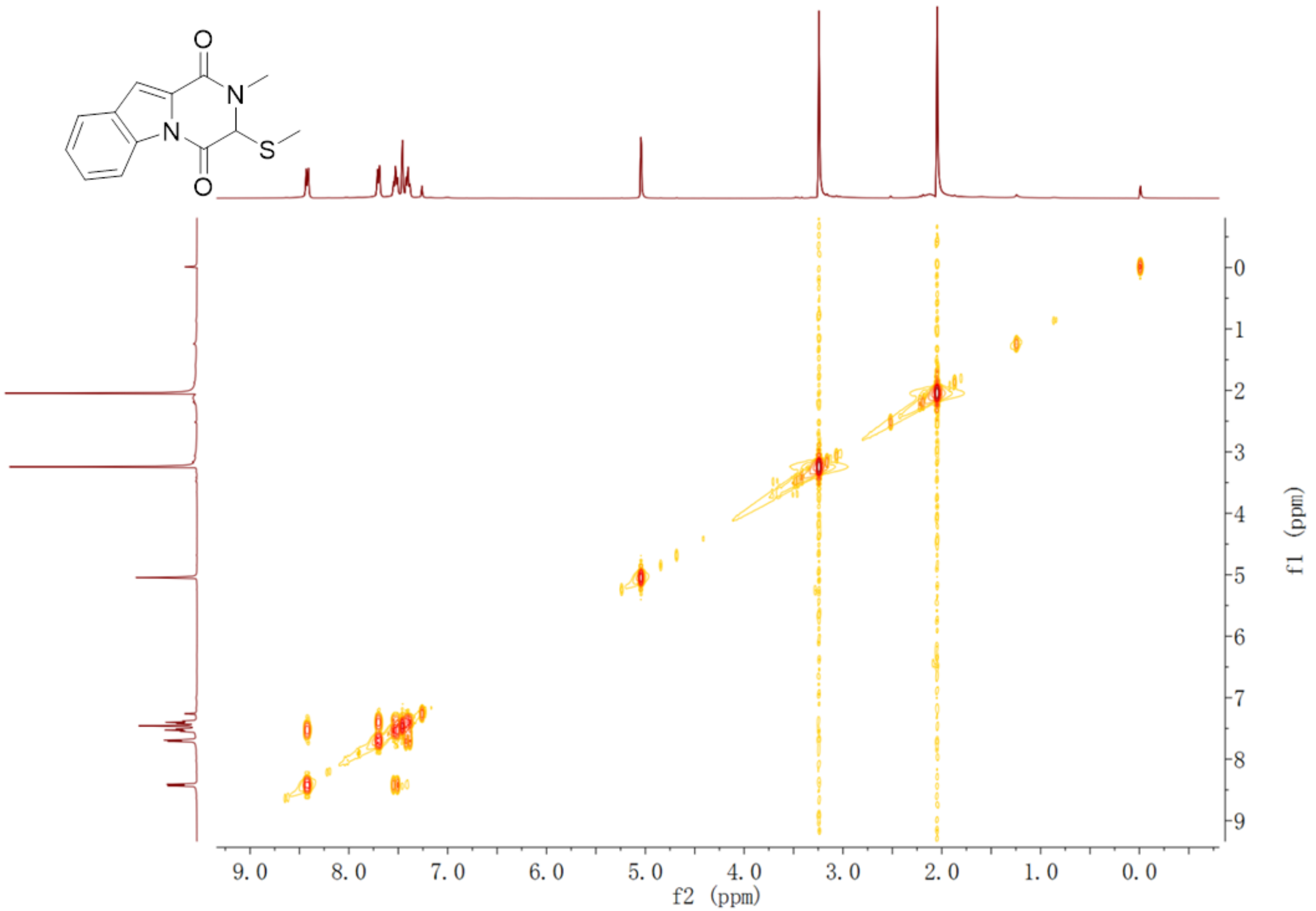

Figure S65. HMBC spectrum of dichocerazine A (**15**) in CDCl3

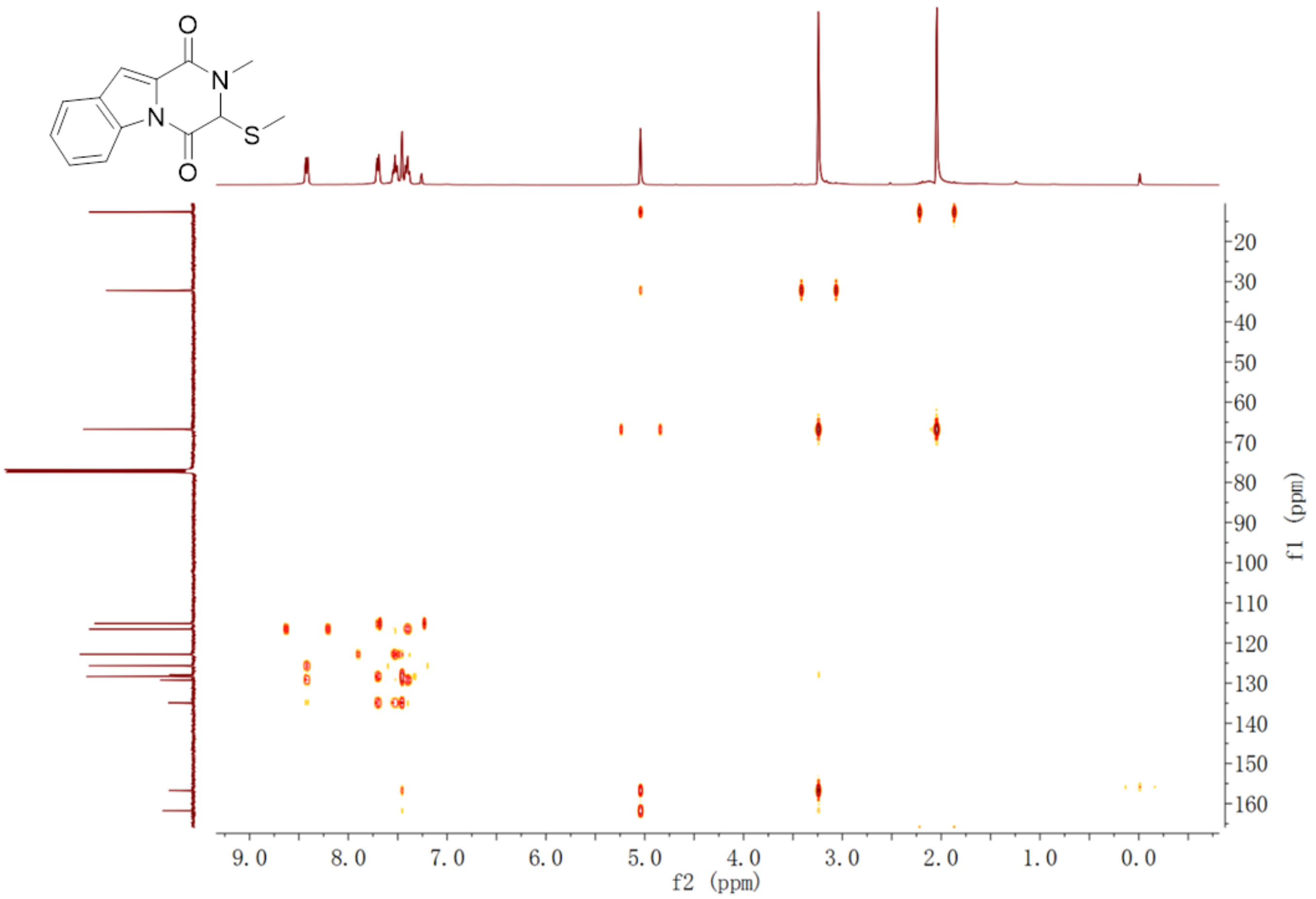

Figure S66. NOESY spectrum of dichocerazine A (**15**) in CDCl3

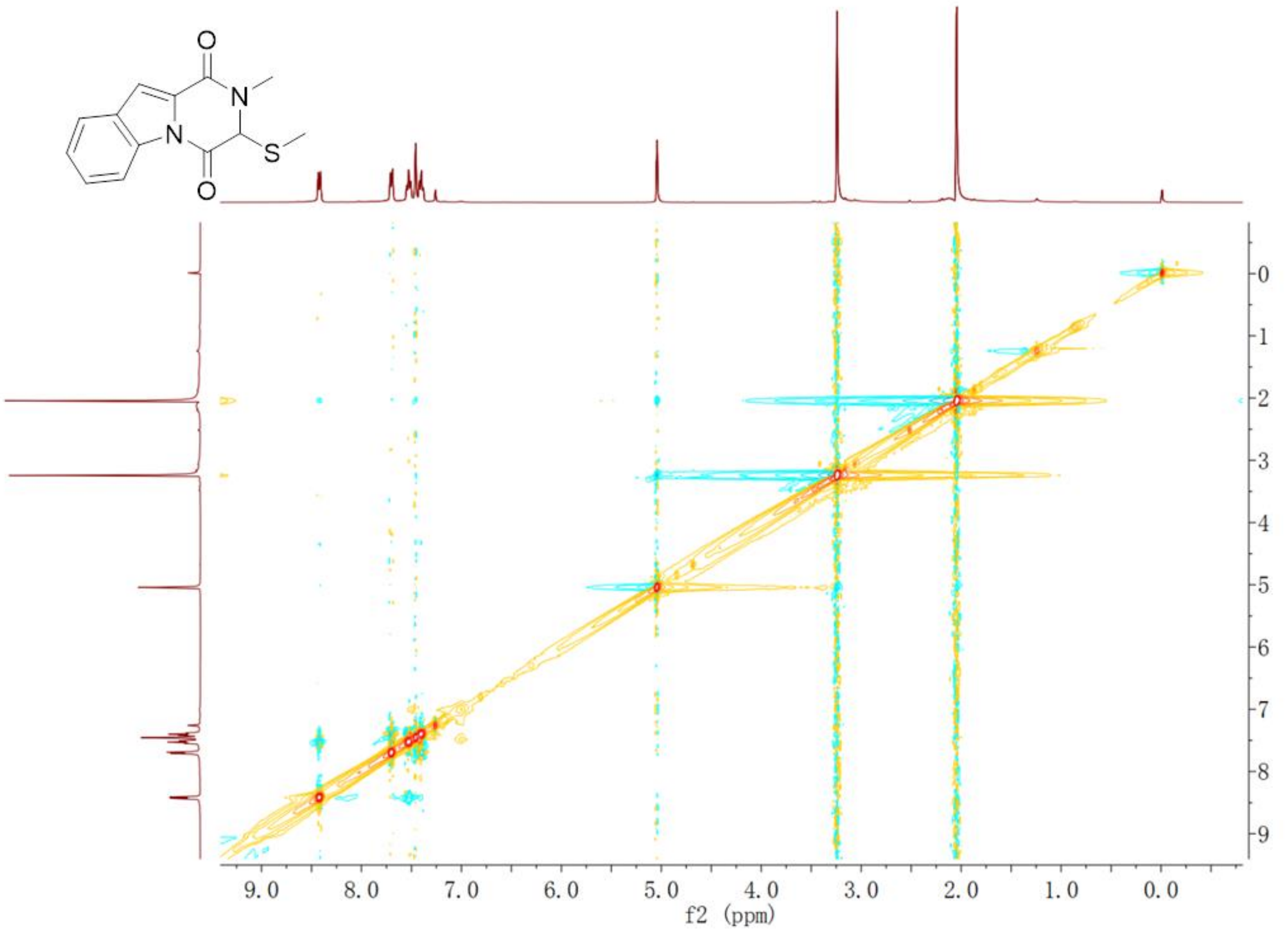

Figure S67. HR-ESI-MS spectrum of dichocerazine B (**16**)

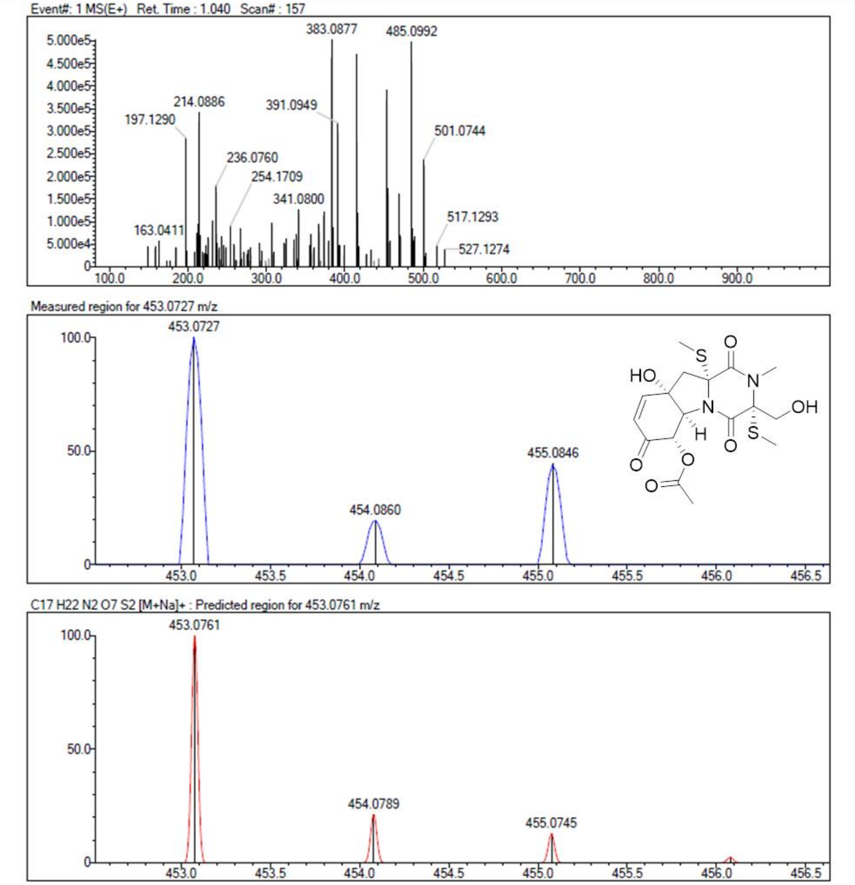

Rank	Score	Formula (M)	Ion	Meas. m/z	Pred. m/z	Df. (mDa)	Df. (ppm)	Iso	DBE
1	25.11	C17 H22 N2 O7 S2	[M+Na]+	453.0727	453.0761	-3.4	-7.50	38.64	8.0

Figure S68. ^{1}H NMR spectrum of dichocerazine B (**16**) in $CDCl_3$ (400MHz)

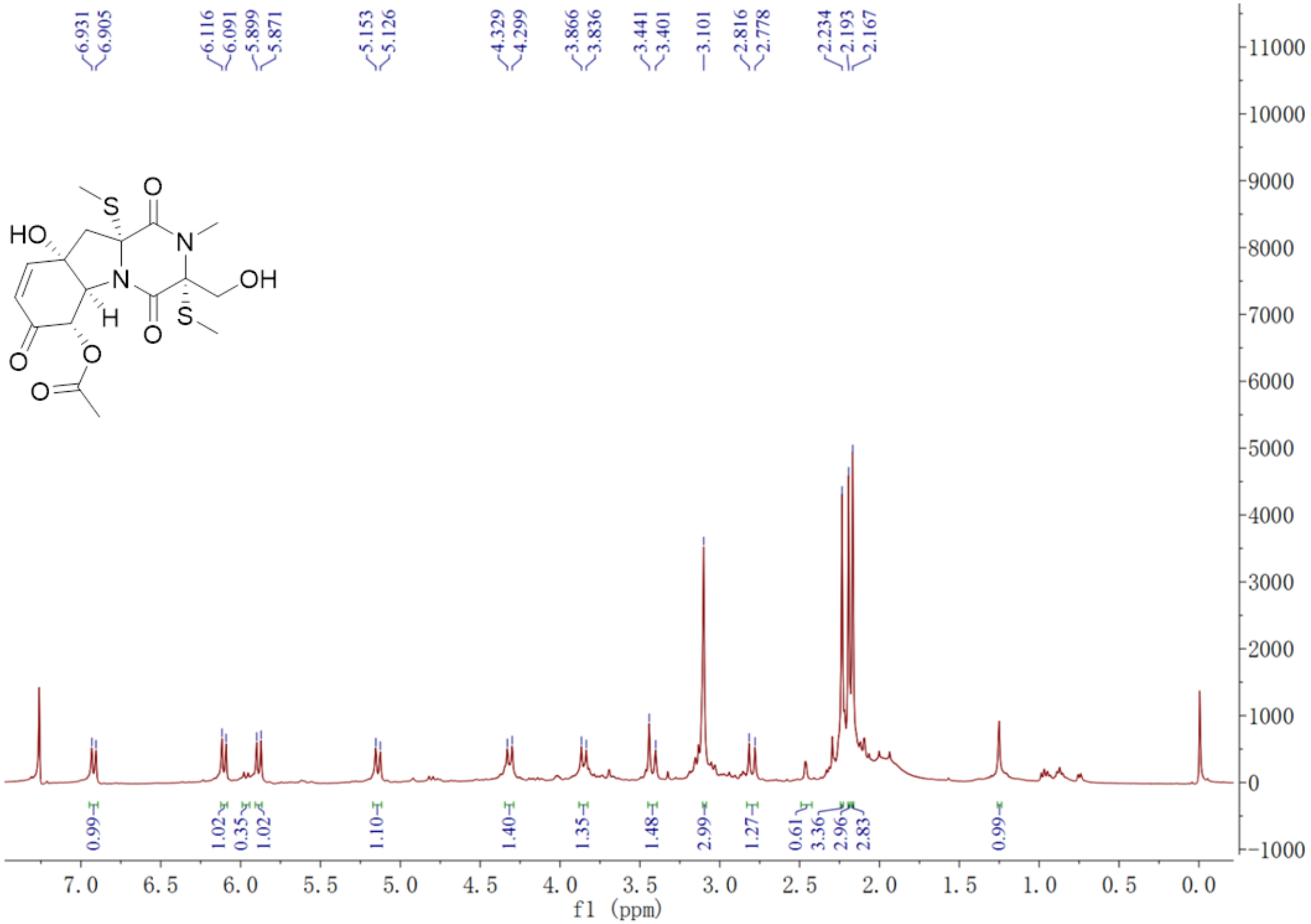

Figure S69 ^{13}C NMR spectrum of dichocerazine B (**16**) in $CDCl_3$ (100MHz)

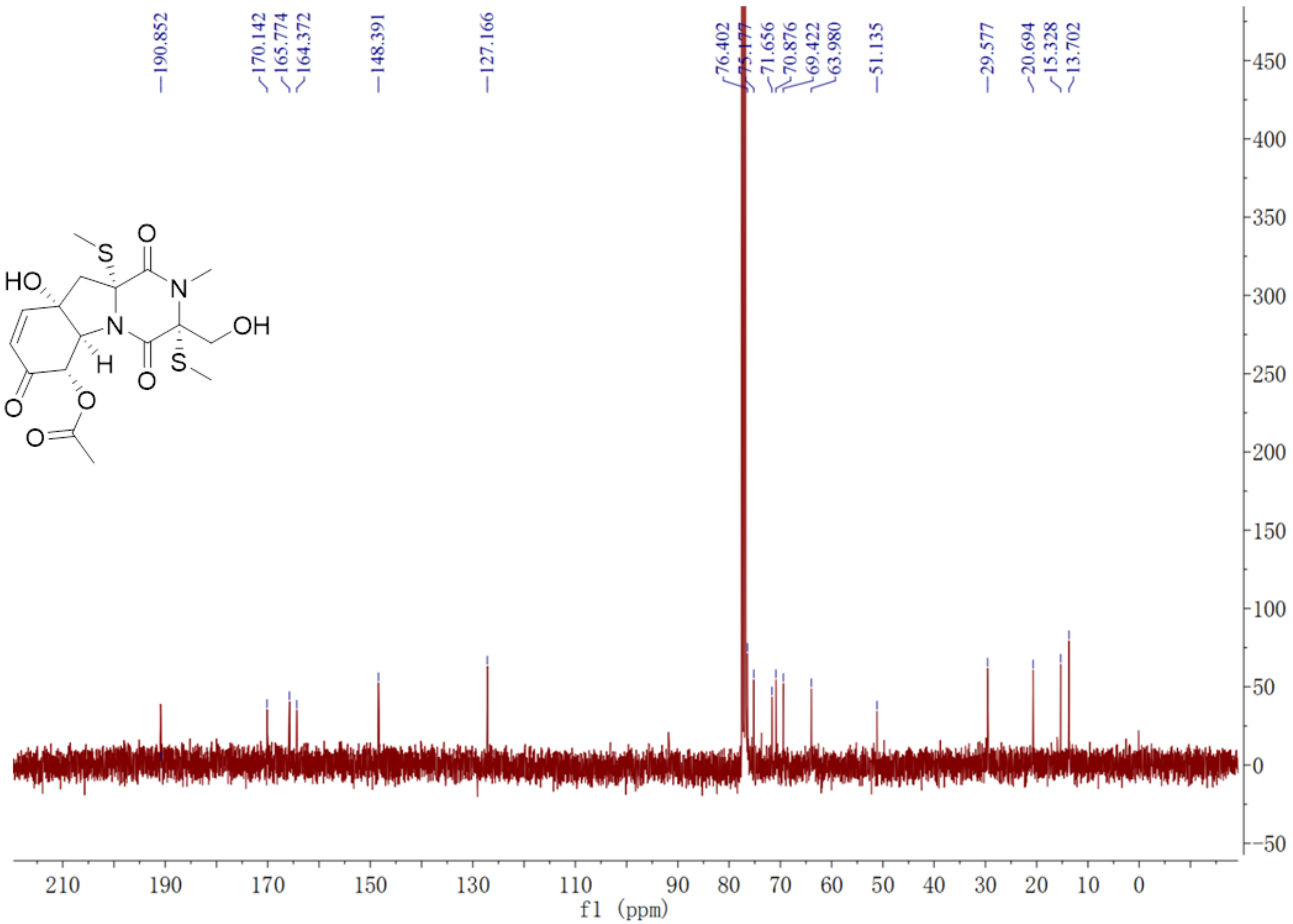

Figure S70. DEPT 135 spectrum of dichocerazine B (**16**) in CDCl3 (100MHz)

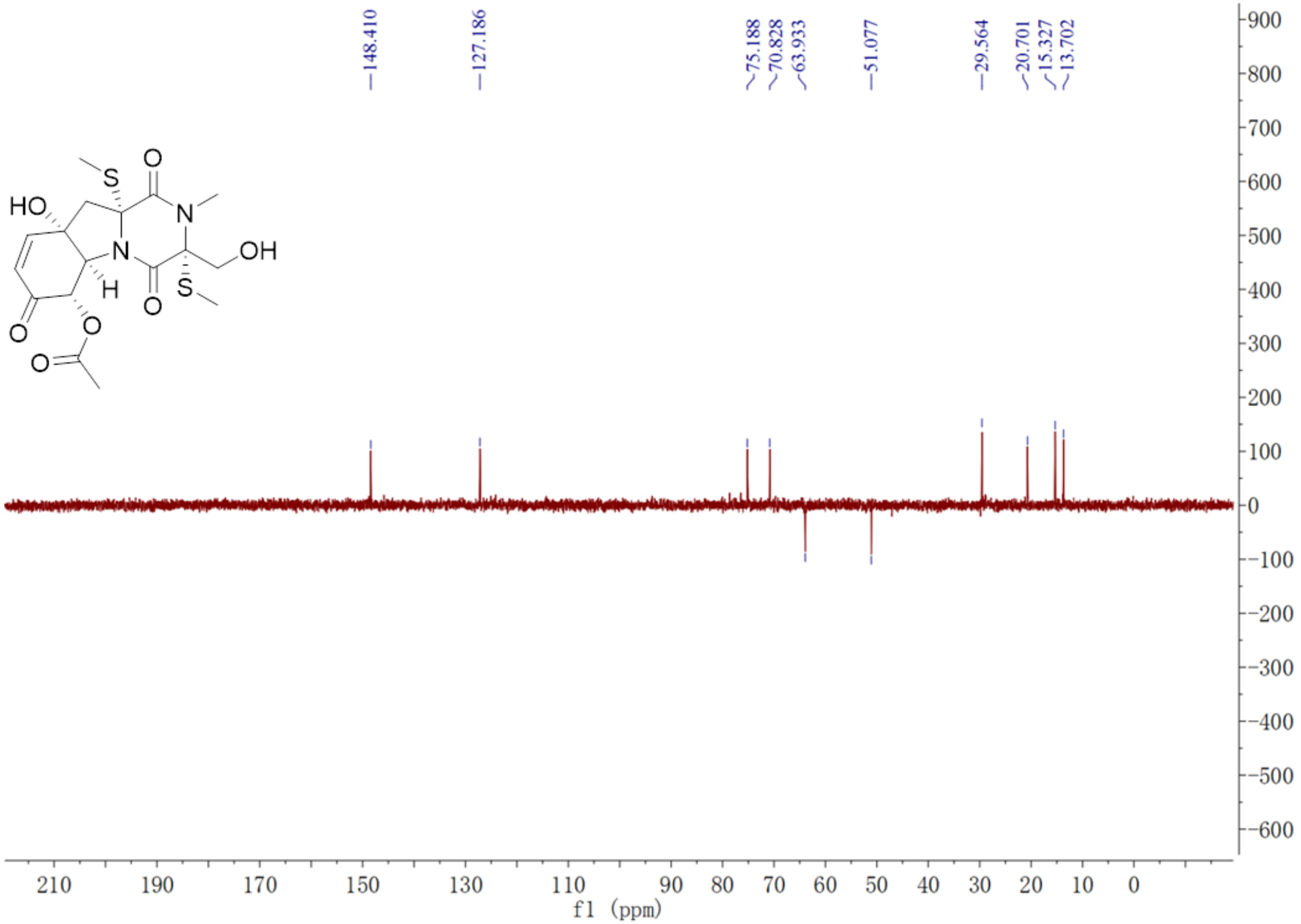

Figure S71. HMQC spectrum of dichocerazine B (**16**) in $CDCl_3$

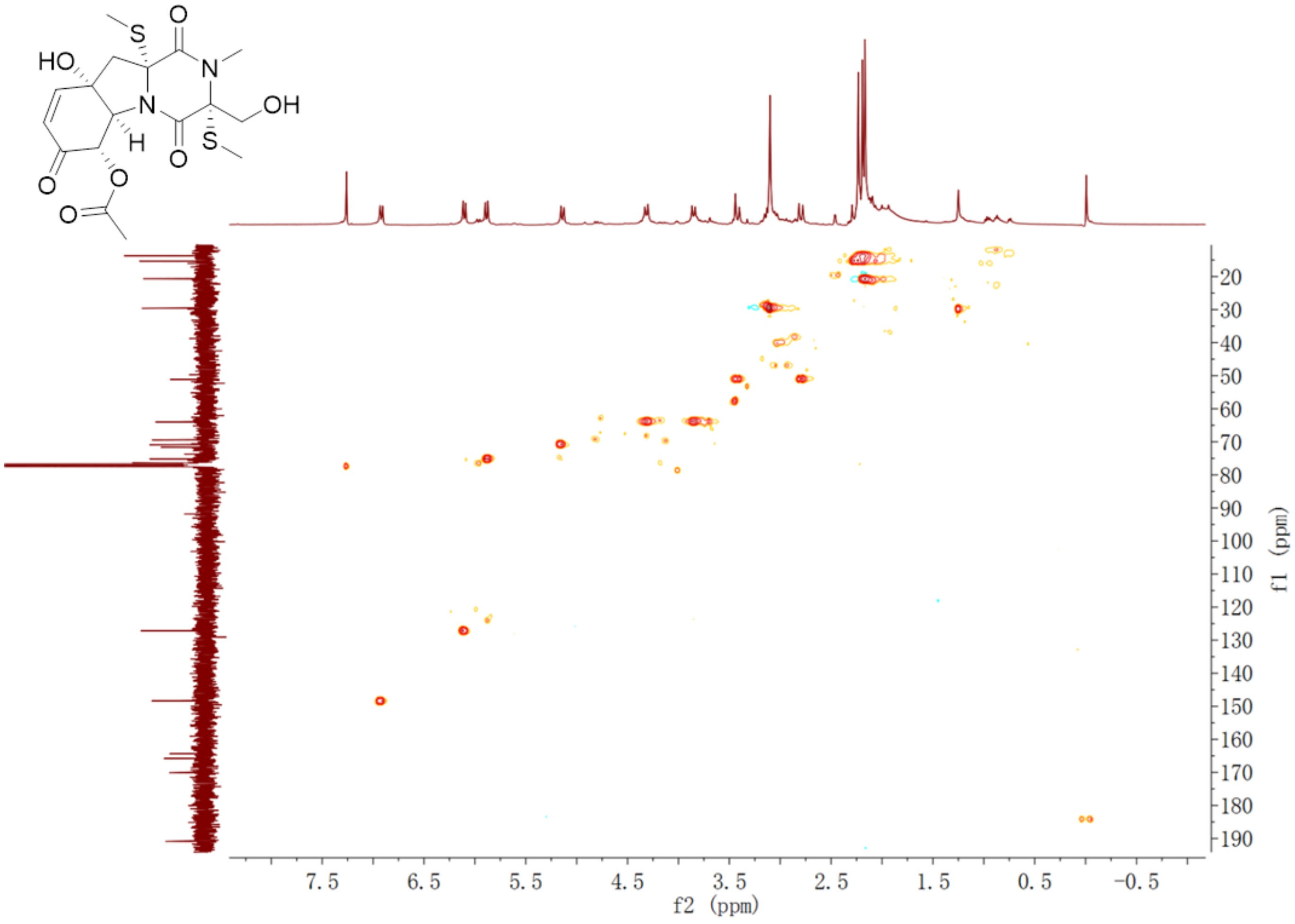

Figure S72. ^{1}H-^{1}H COSY spectrum of dichocerazine B (**16**) in $CDCl_3$

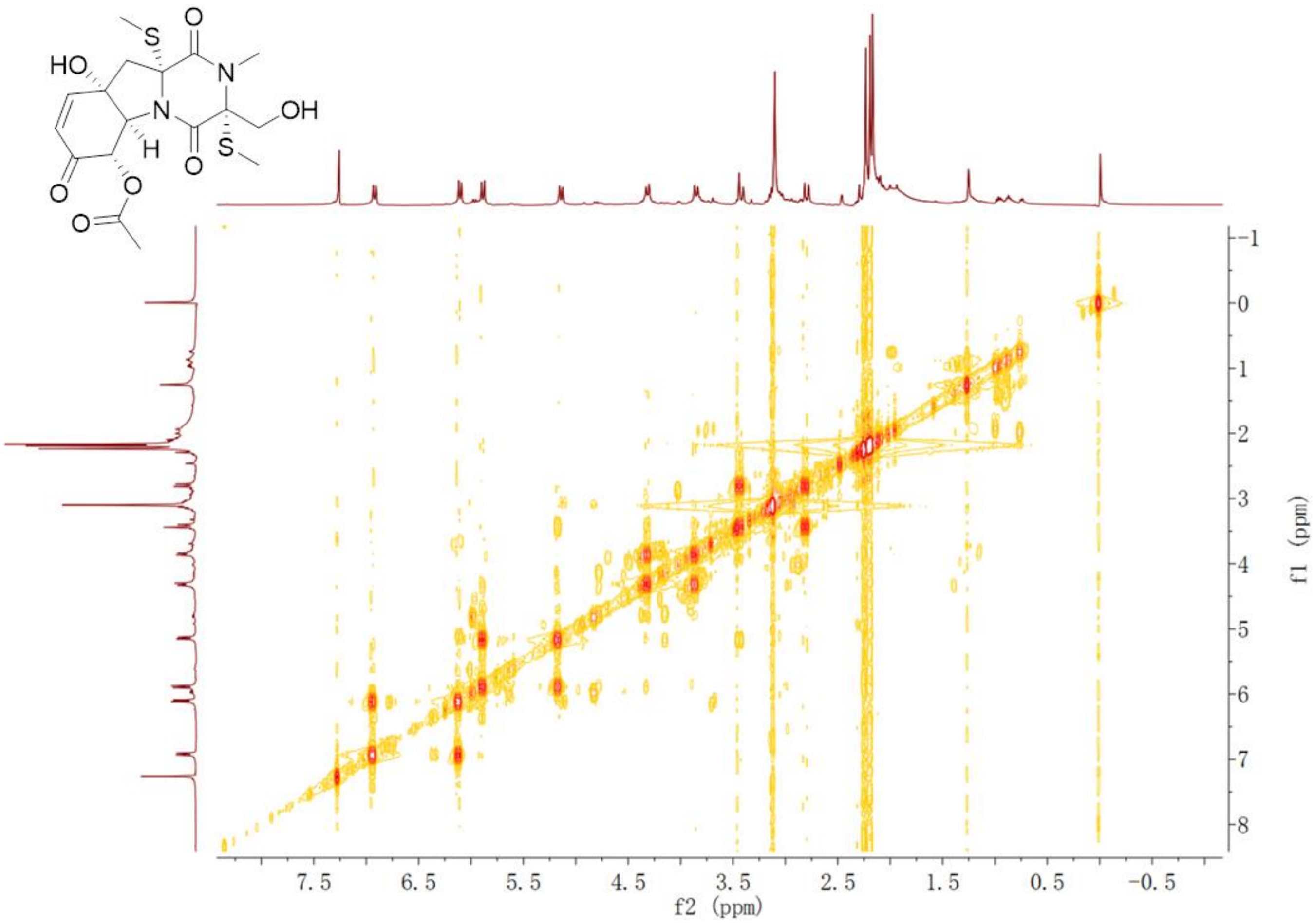

Figure S73. HMBC spectrum of dichocerazine B (**16**) in $CDCl_3$

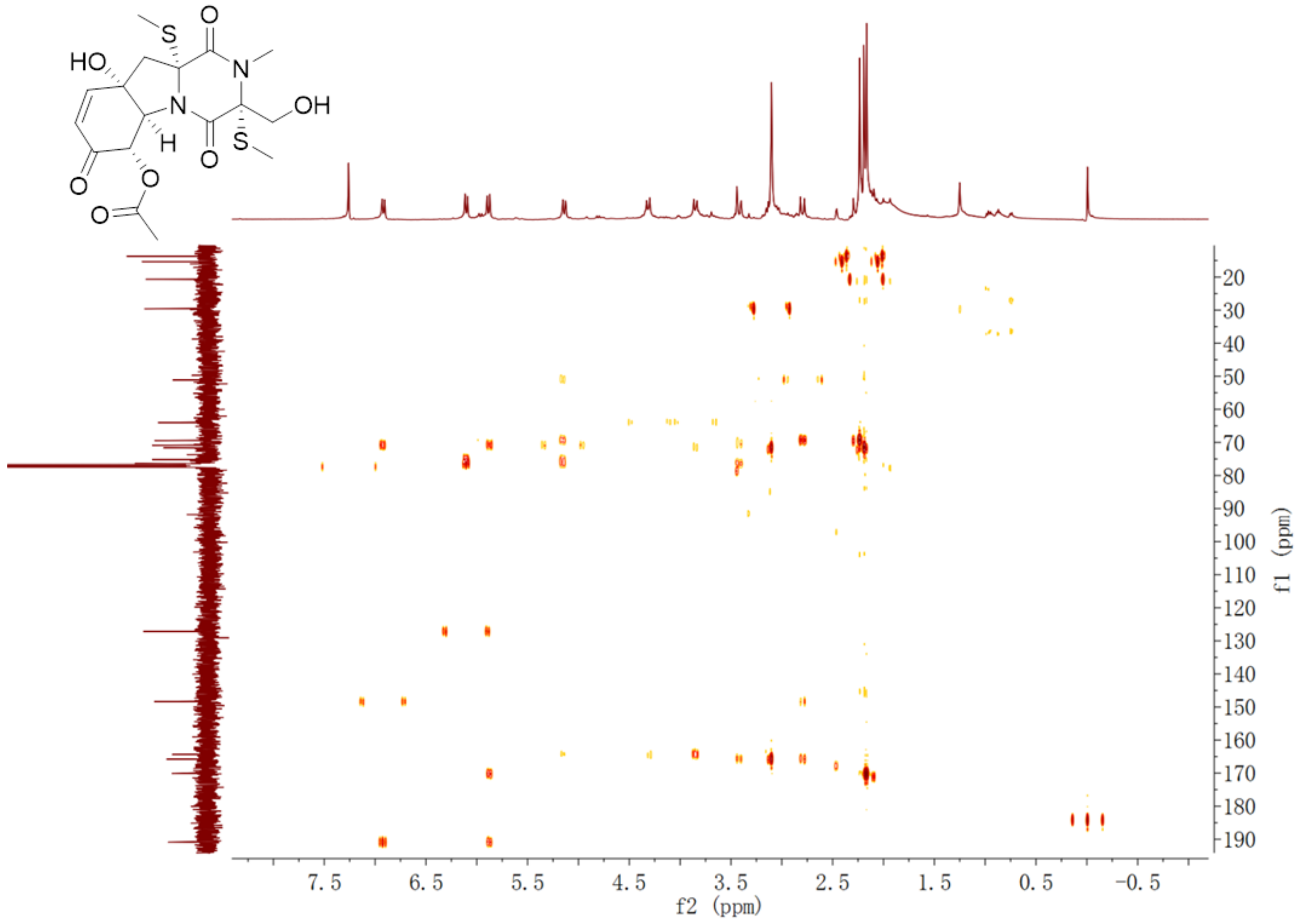

Figure S74. NOESY spectrum of dichocerazine B (**16**) in $CDCl_3$

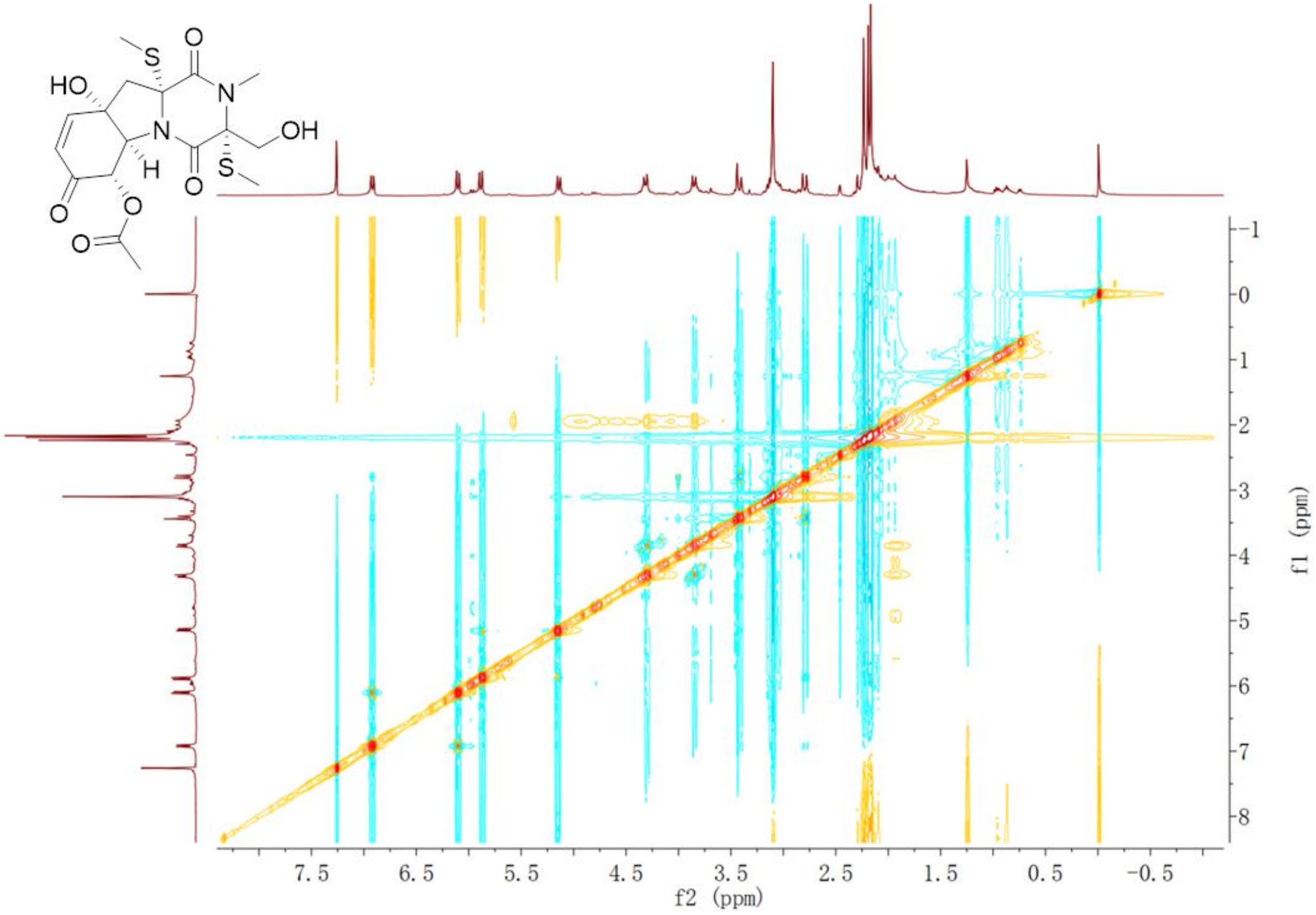

Figure S75. ^{1}H NMR spectrum of dichotocejpin A (**17**) in $CDCl_3$ (400MHz)

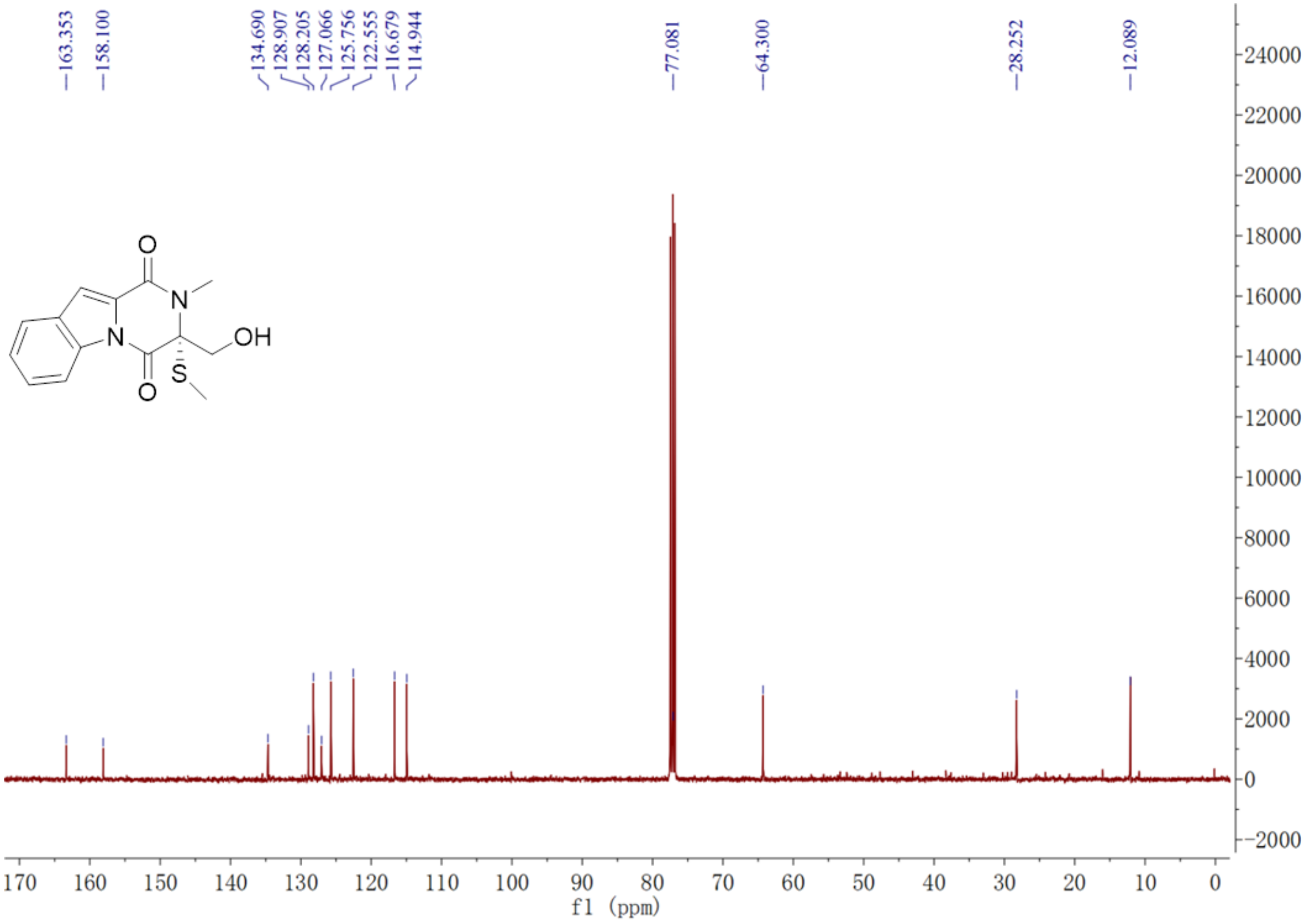

Figure S76. ^{13}C NMR spectrum of dichotocejpin A (**17**) in $CDCl_3$ (100MHz)

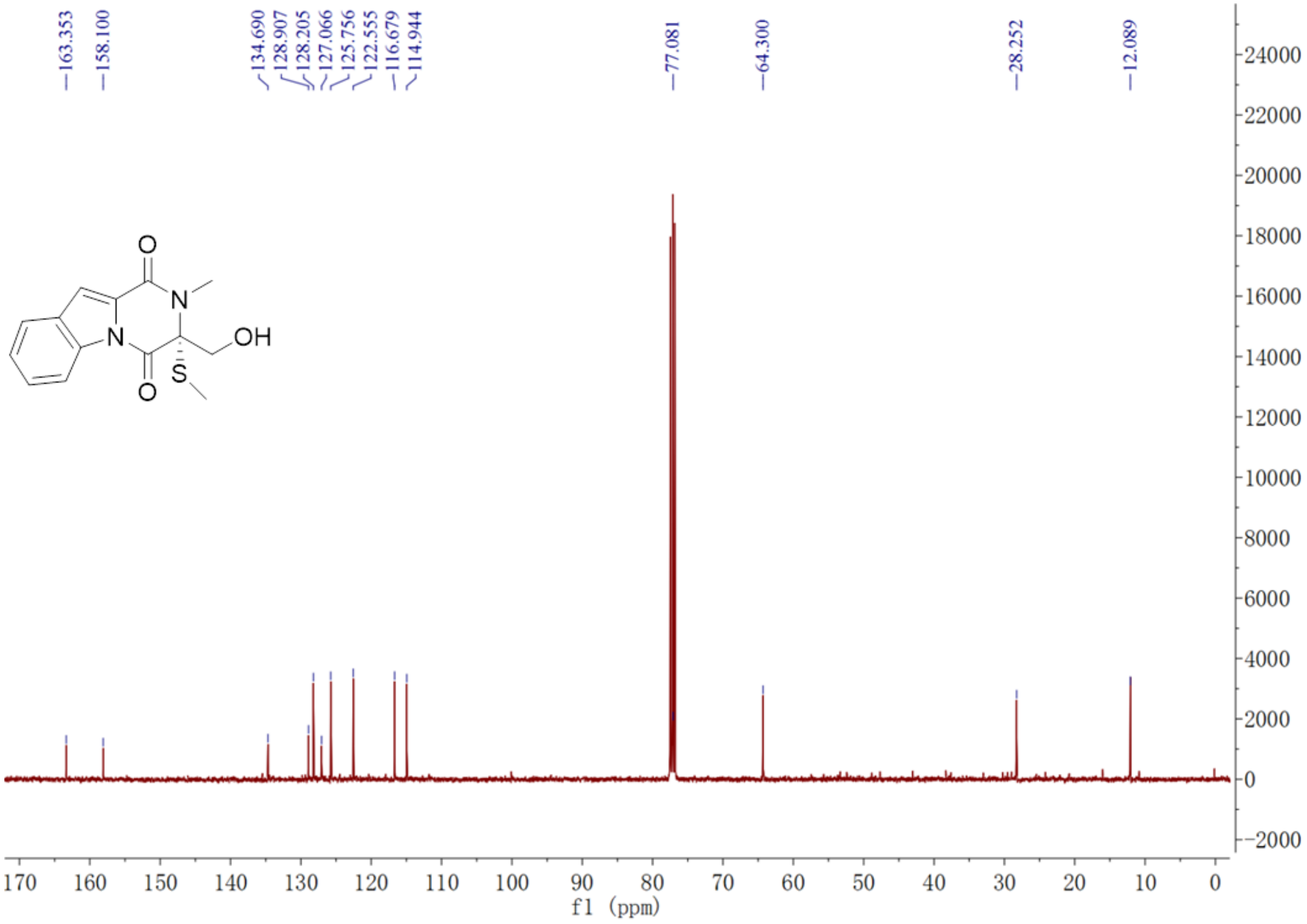

Figure S77. ^{1}H NMR spectrum of bisdethiobis (methylthio) gliotoxin (**18**) in $CDCl_3$ (400MHz)

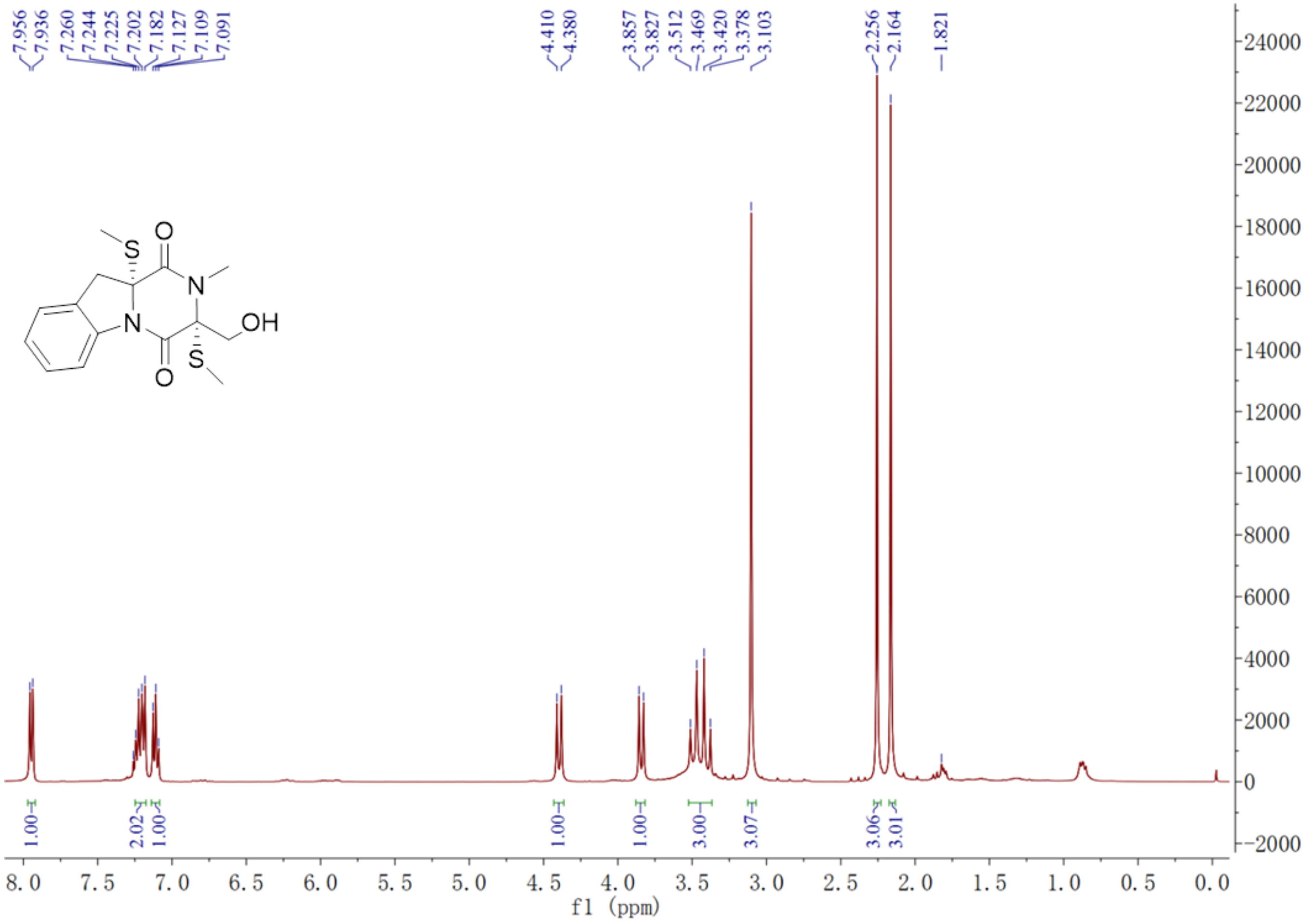

Figure S78. ^{13}C NMR spectrum of bisdethiobis (methylthio) gliotoxin (**18**) in $CDCl_3$ (100MHz)

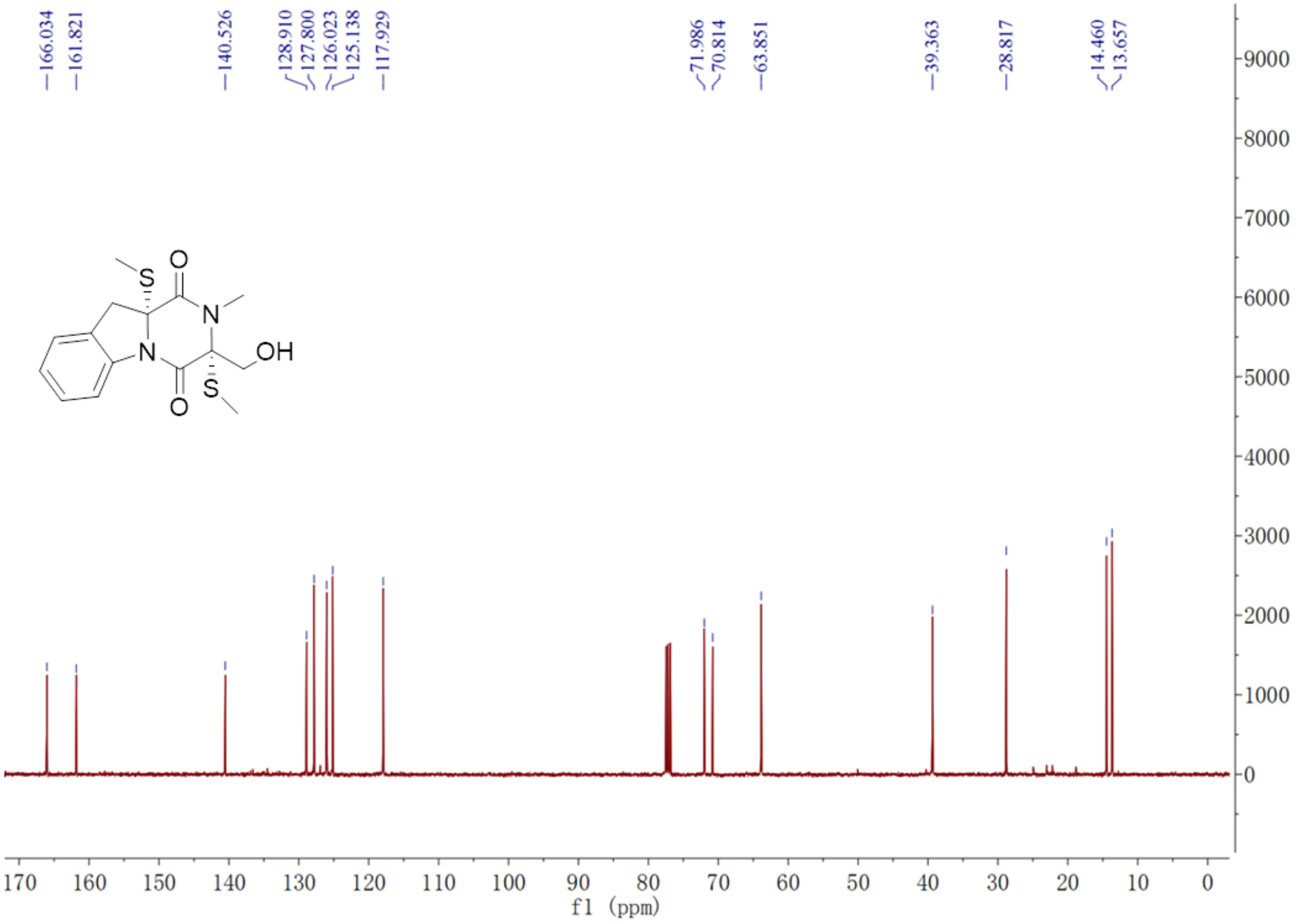

Figure S79. ^{1}H NMR spectrum of 6-acetylbis (methylthio) gliotoxin (**19**) in $CDCl_3$ (400MHz)

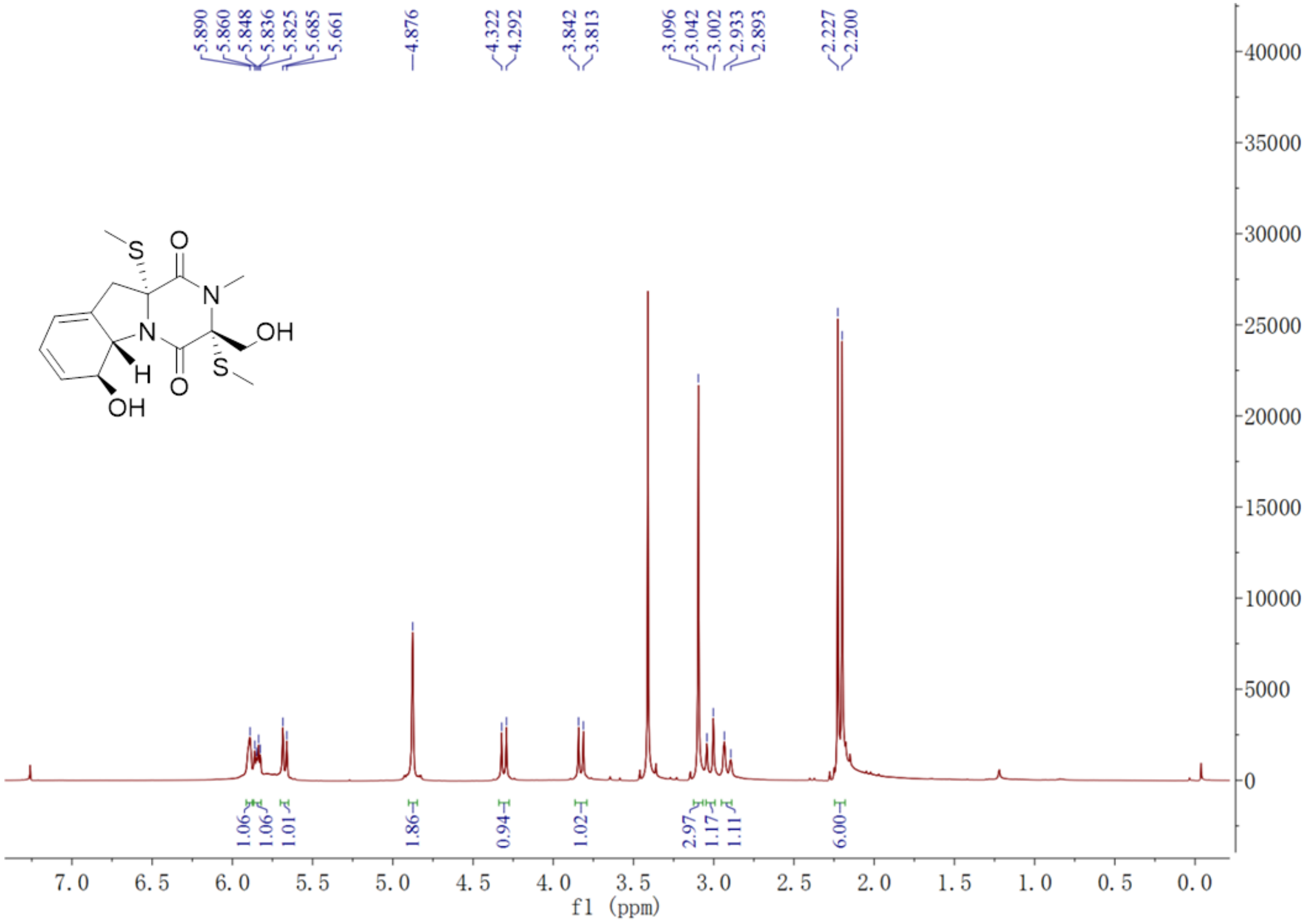

Figure S80. ^{13}C NMR spectrum of 6-acetylbis (methylthio) gliotoxin (**19**) in $CDCl_3$ (100MHz)

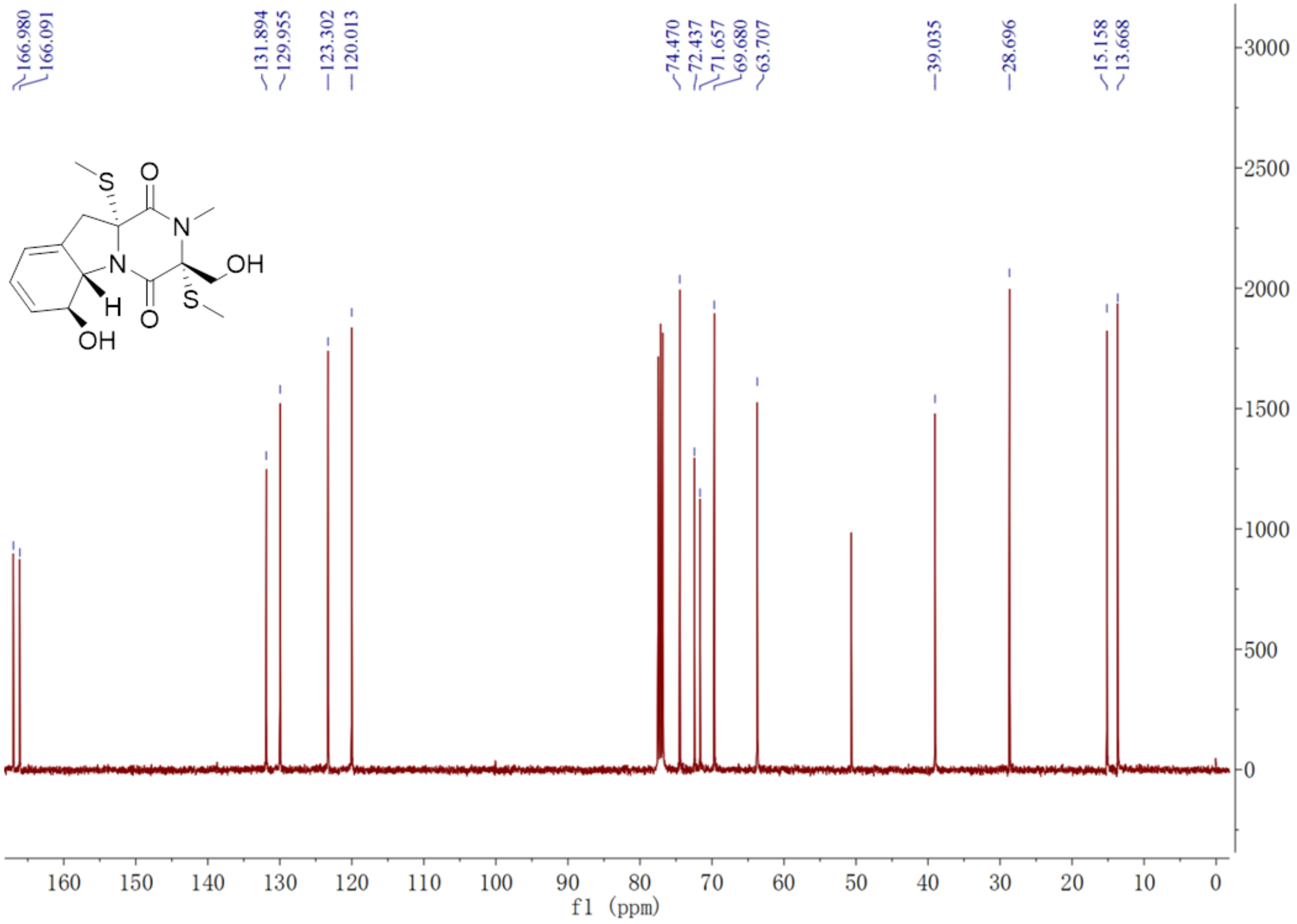

Figure S81. ^{1}H NMR spectrum of didehydrobisdethiobis (methylthio) gliotoxin (**20**) in $CDCl_3$ (400MHz)

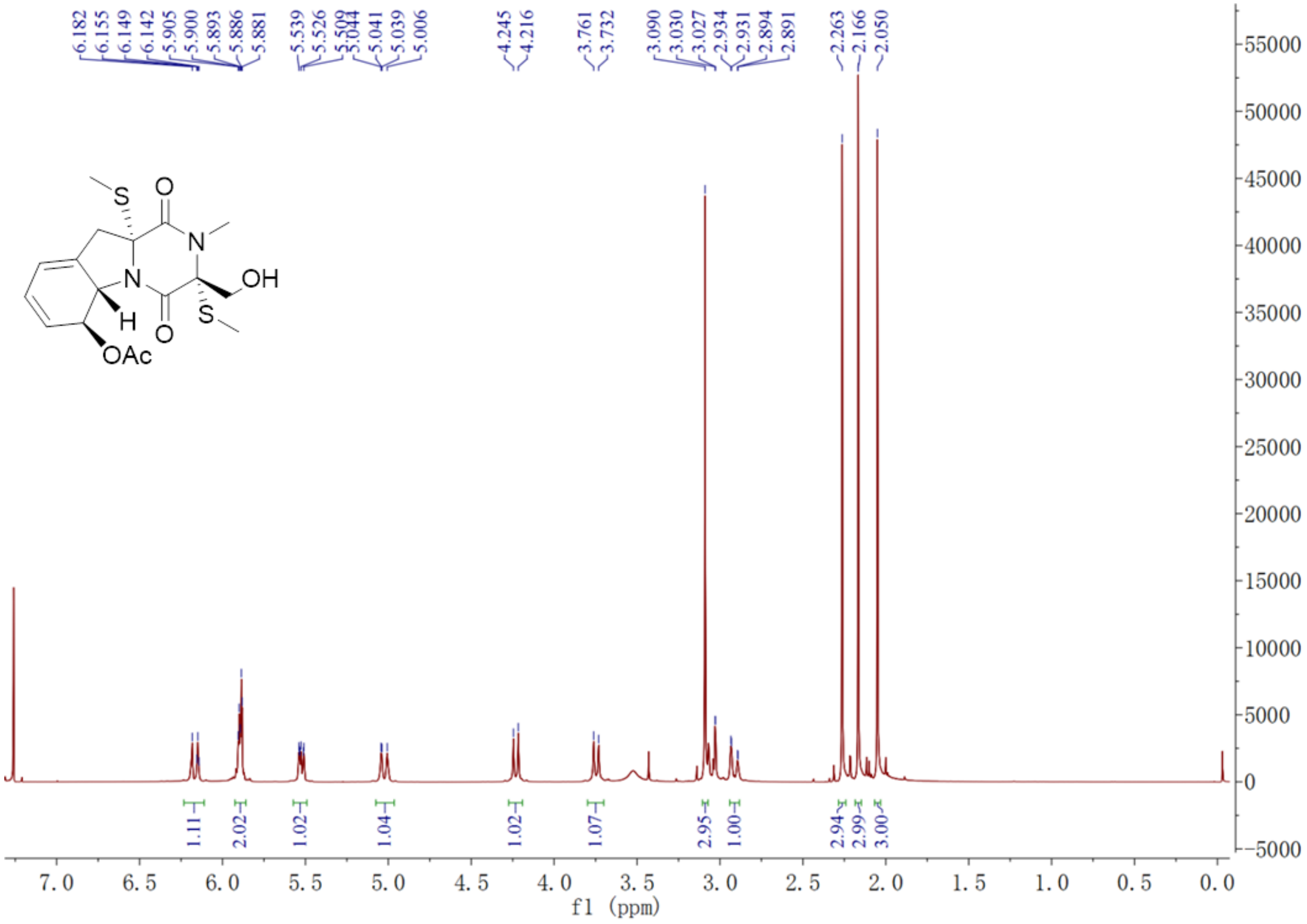

Figure S82. ^{13}C NMR spectrum of didehydrobisdethiobis (methylthio) gliotoxin (**20**) in $CDCl_3$ (100MHz)

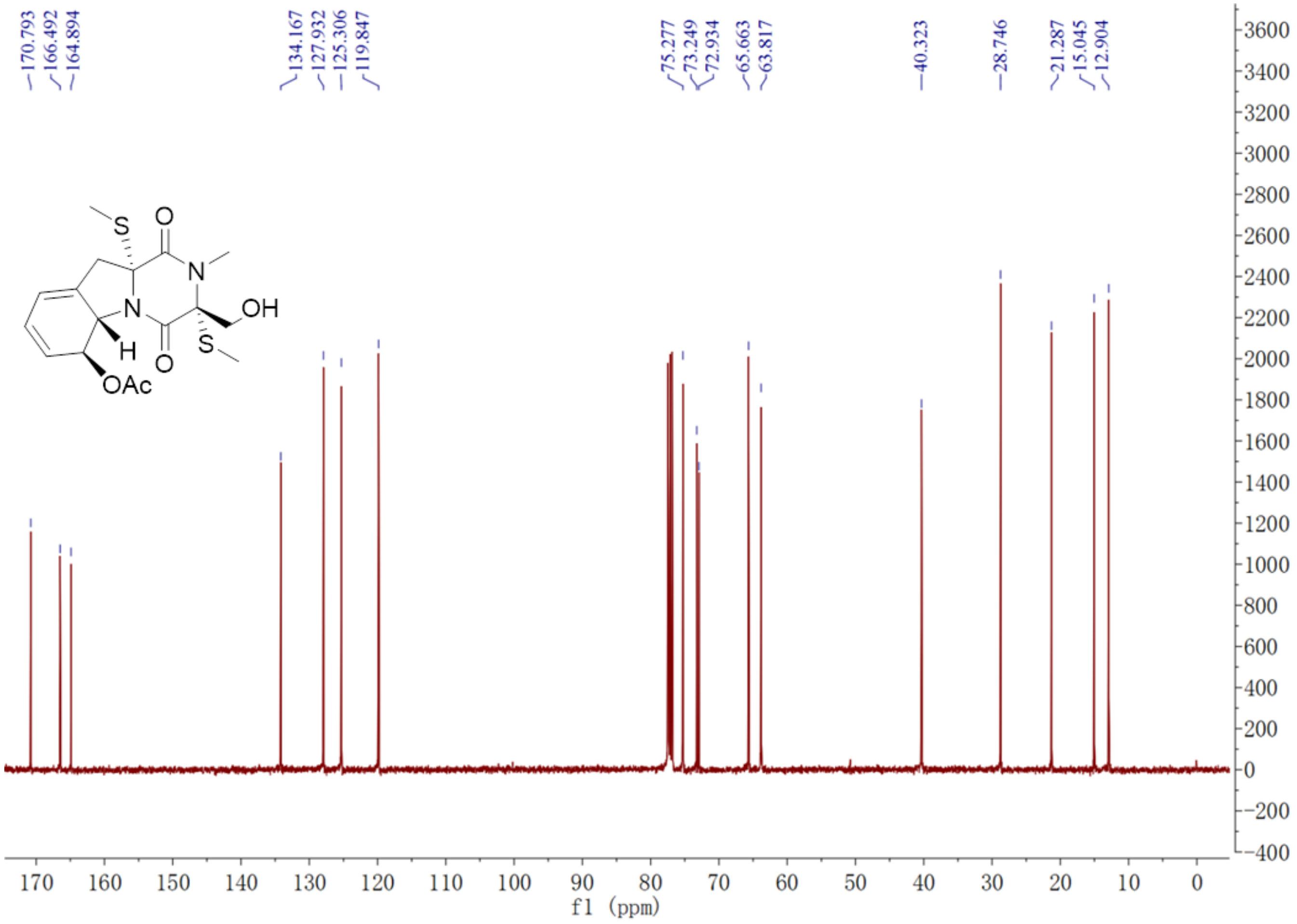

Figure S83. ^{1}H NMR spectrum of haematocin (**21**) in $CDCl_3$ (400MHz)

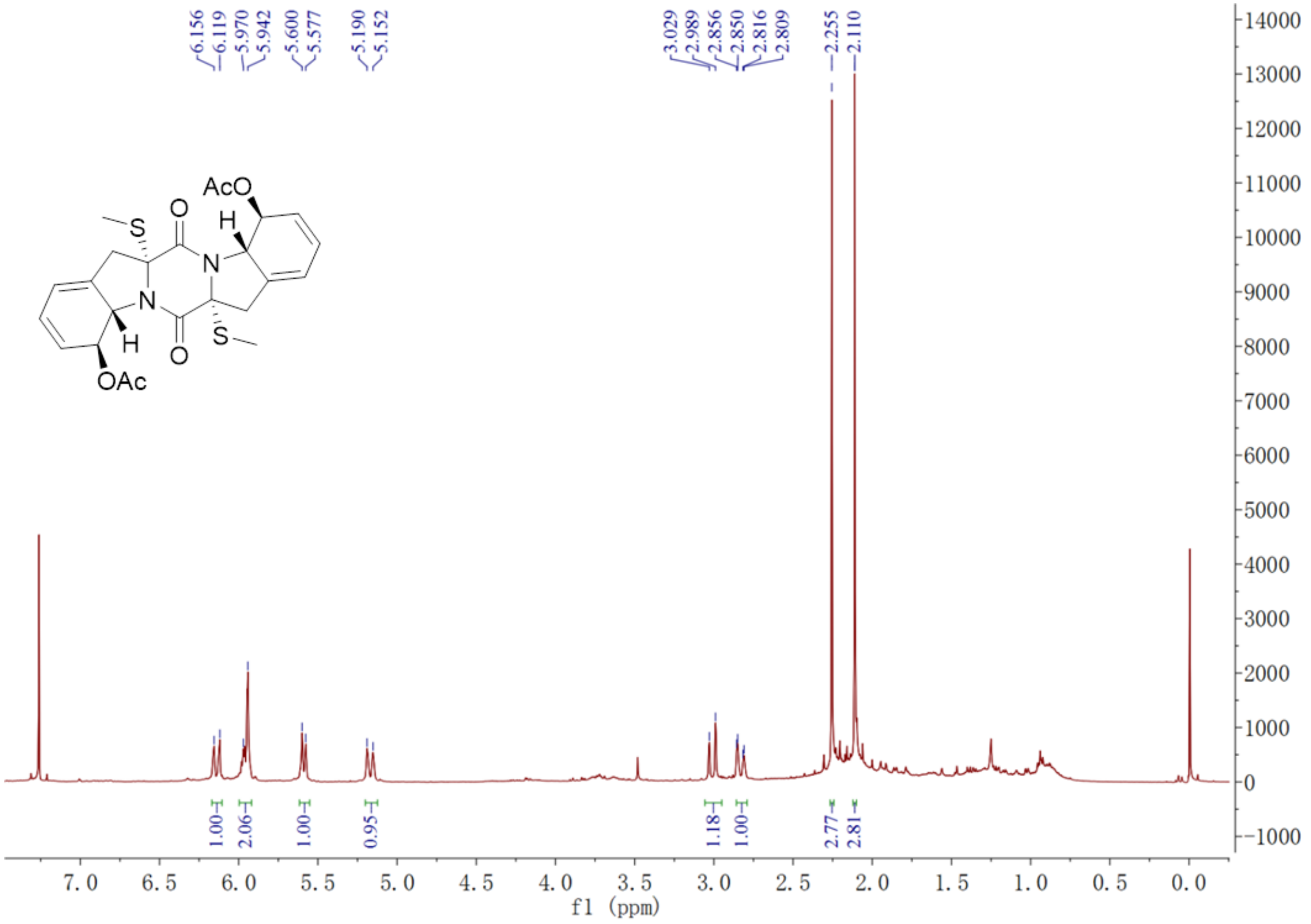

Figure S84. ^{13}C NMR spectrum of haematocin (**21**) in $CDCl_3$ (100MHz)

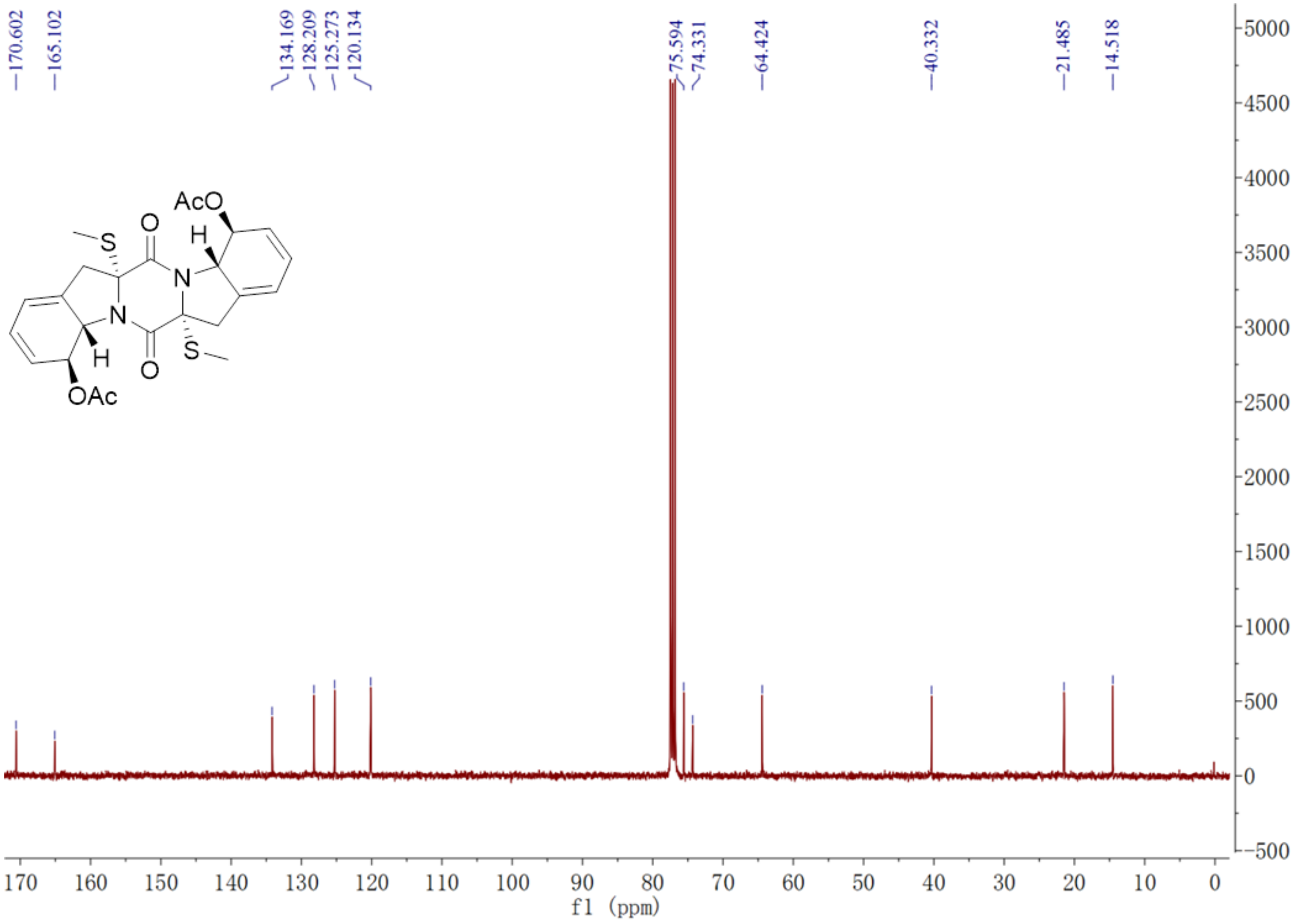

Figure S85. ^{1}H NMR spectrum of pityriacitrin (**22**) in Acetone-d_6 (400MHz)

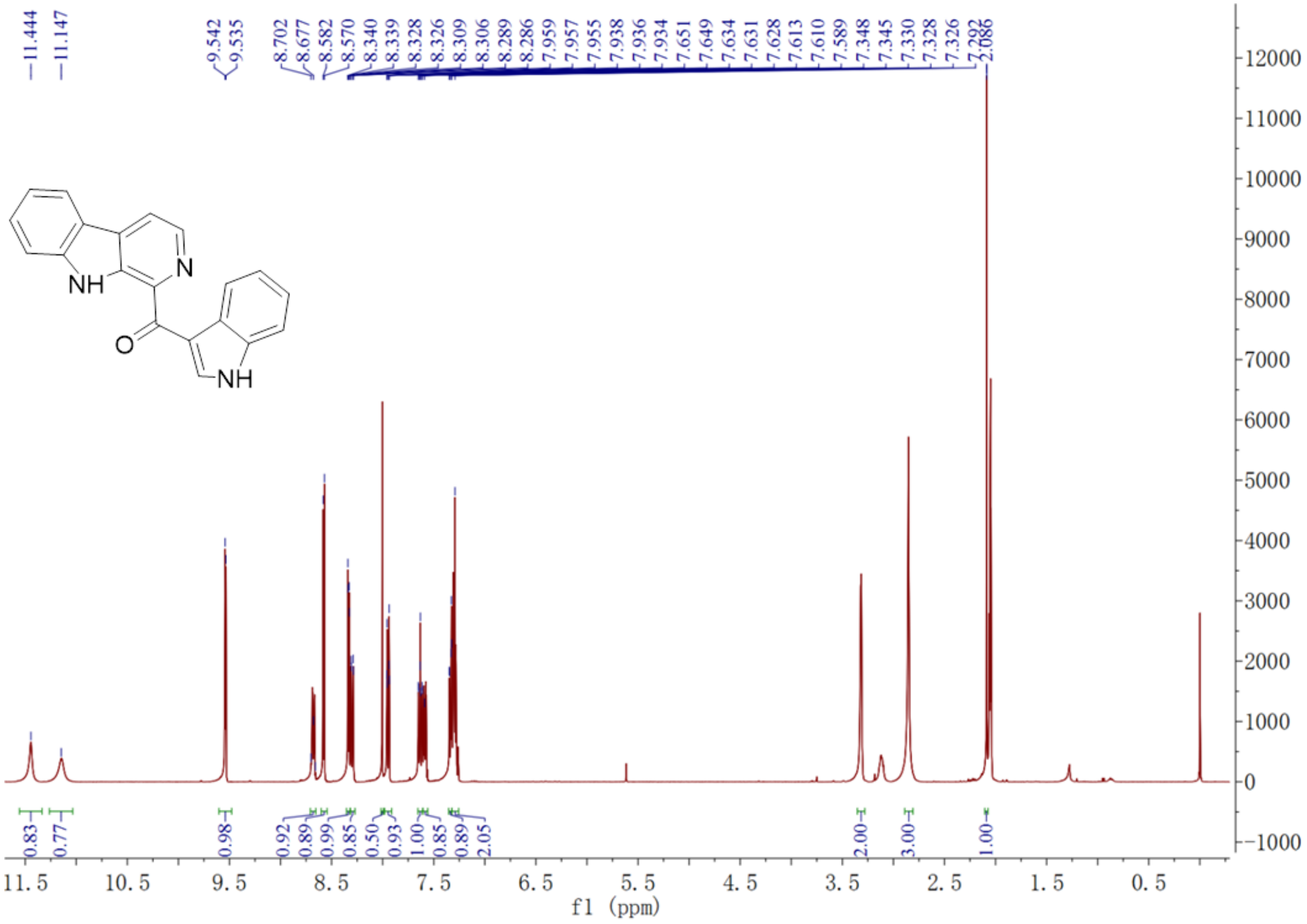

Figure S86. ^{13}C NMR spectrum of pityriacitrin (**22**) in Acetone-d_6 (100MHz)

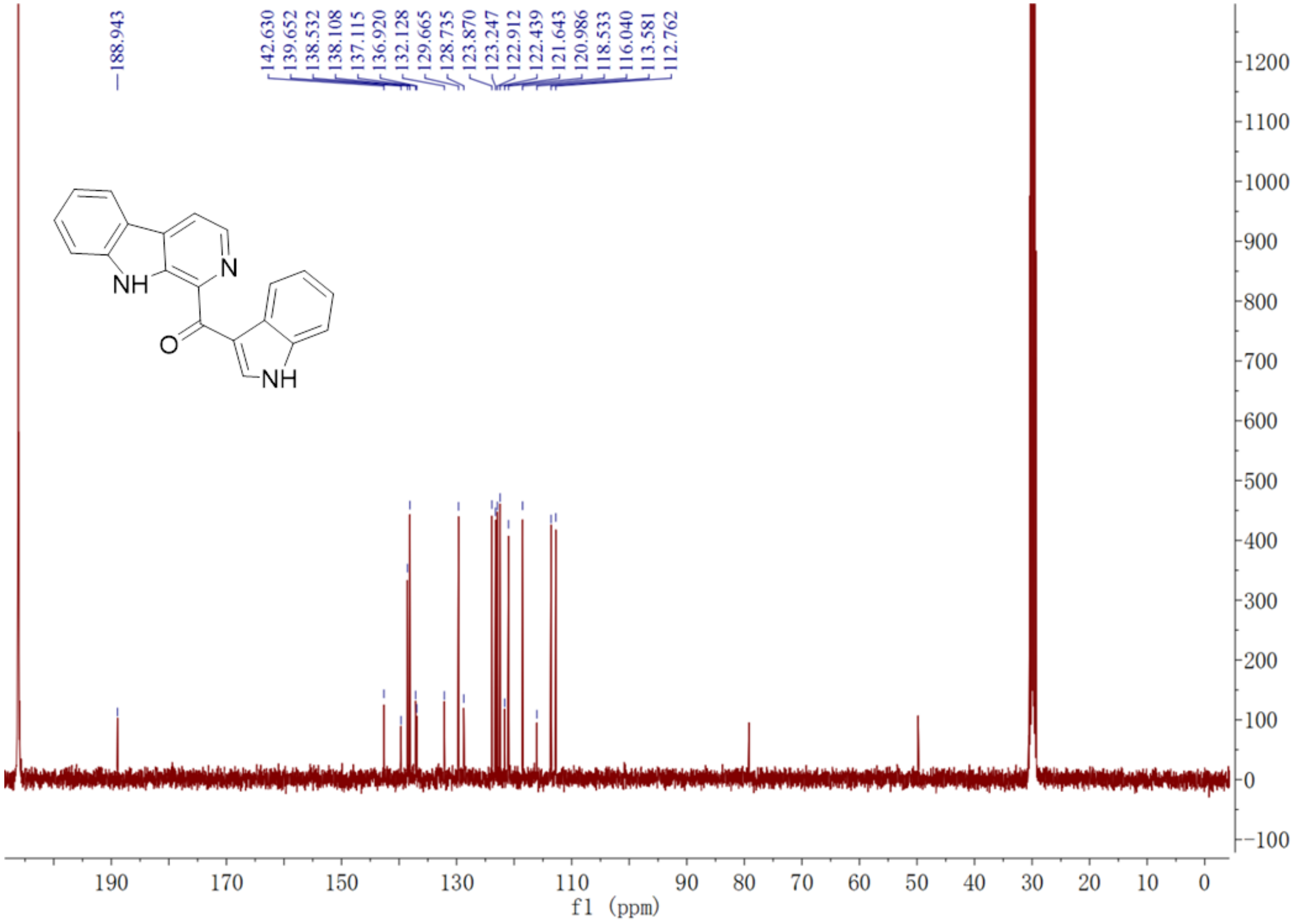

Figure S87. ^{1}H NMR spectrum of stellarine A (**23**) in DMSO-d_6 (400MHz)

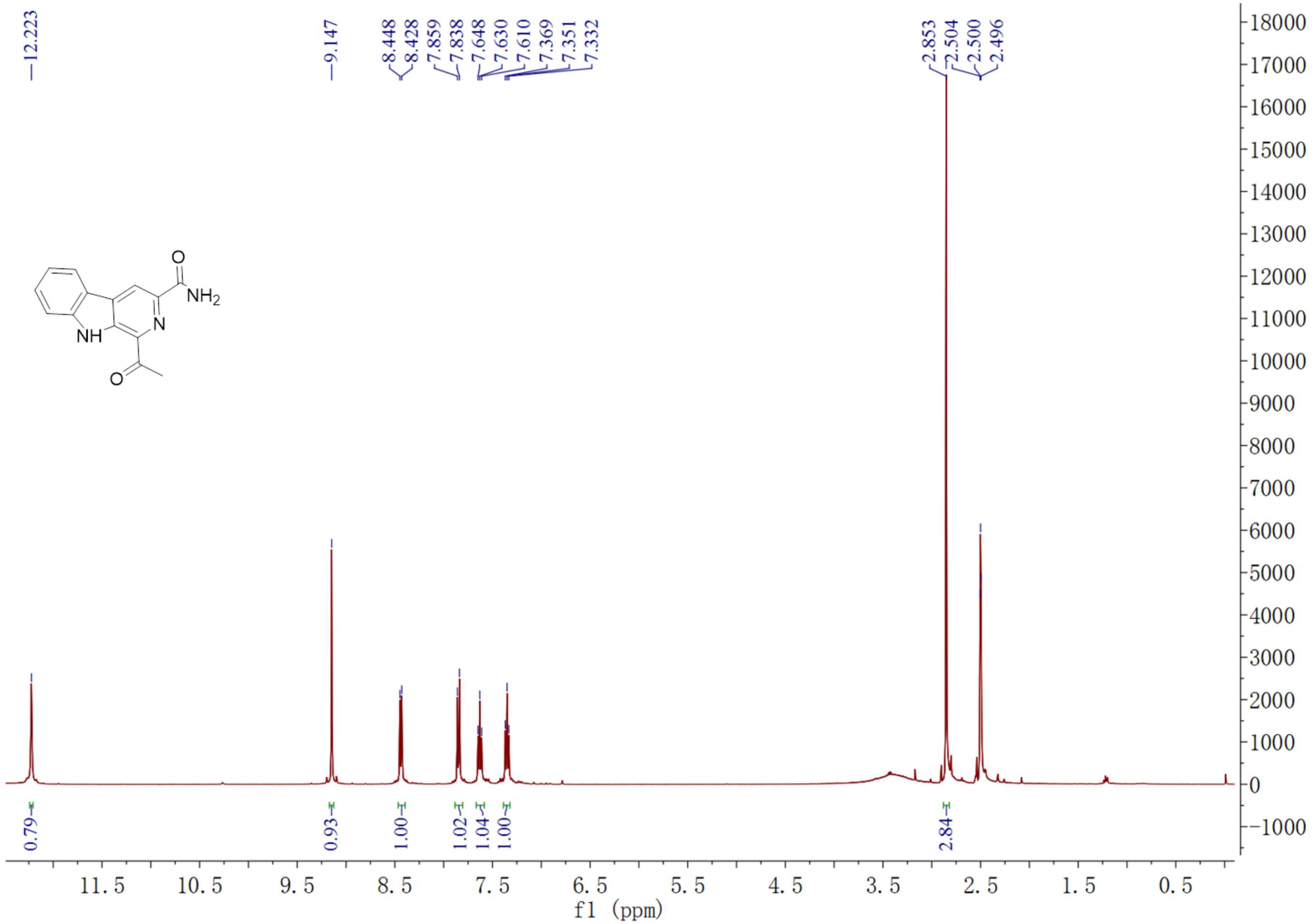

Figure S88. ^{13}C NMR spectrum of stellarine A (**23**) in DMSO-d_6 (100MHz)

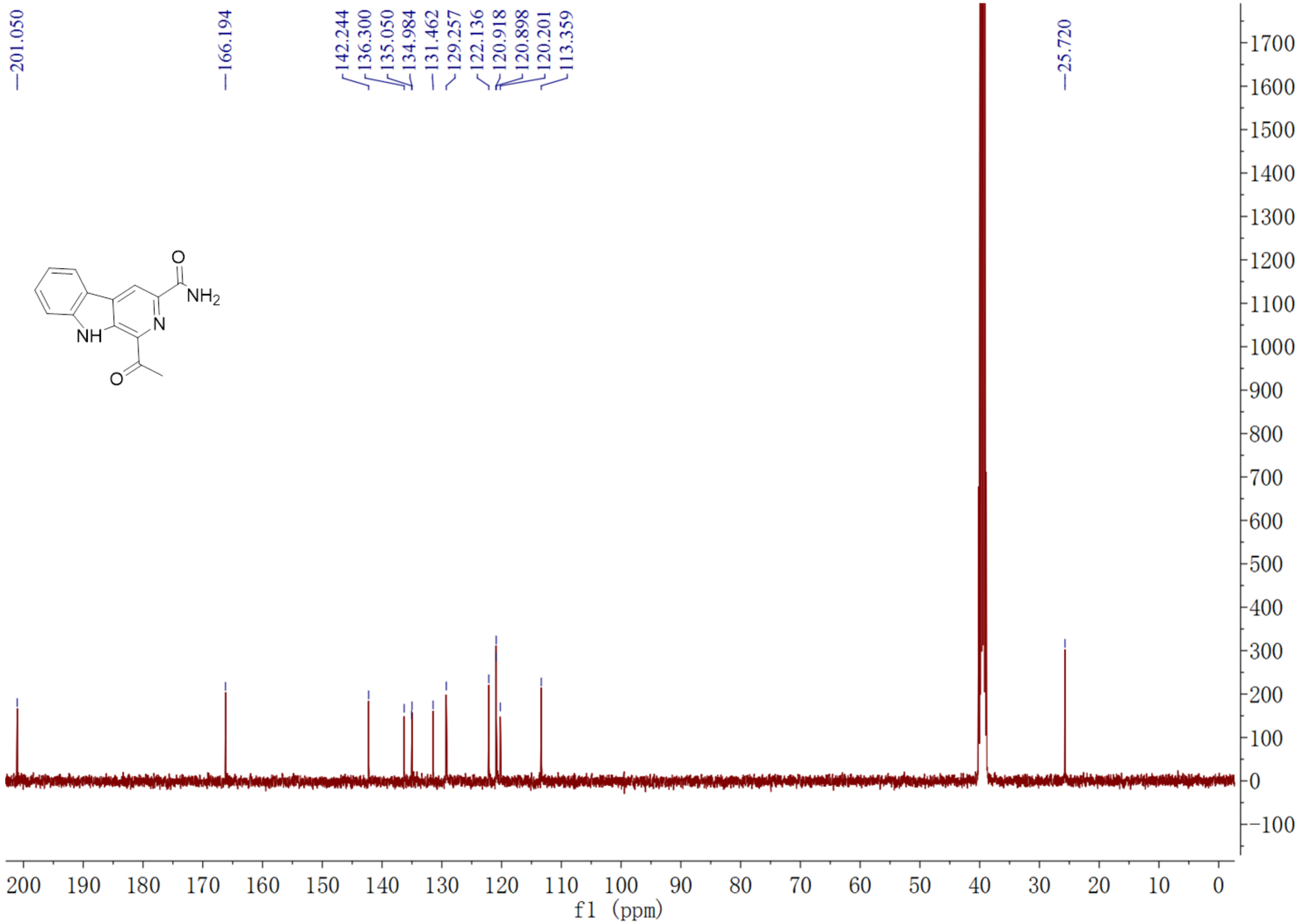

Figure S89. ^{1}H NMR spectrum of perlolyrine (**24**) in $CDCl_3$ (400MHz)

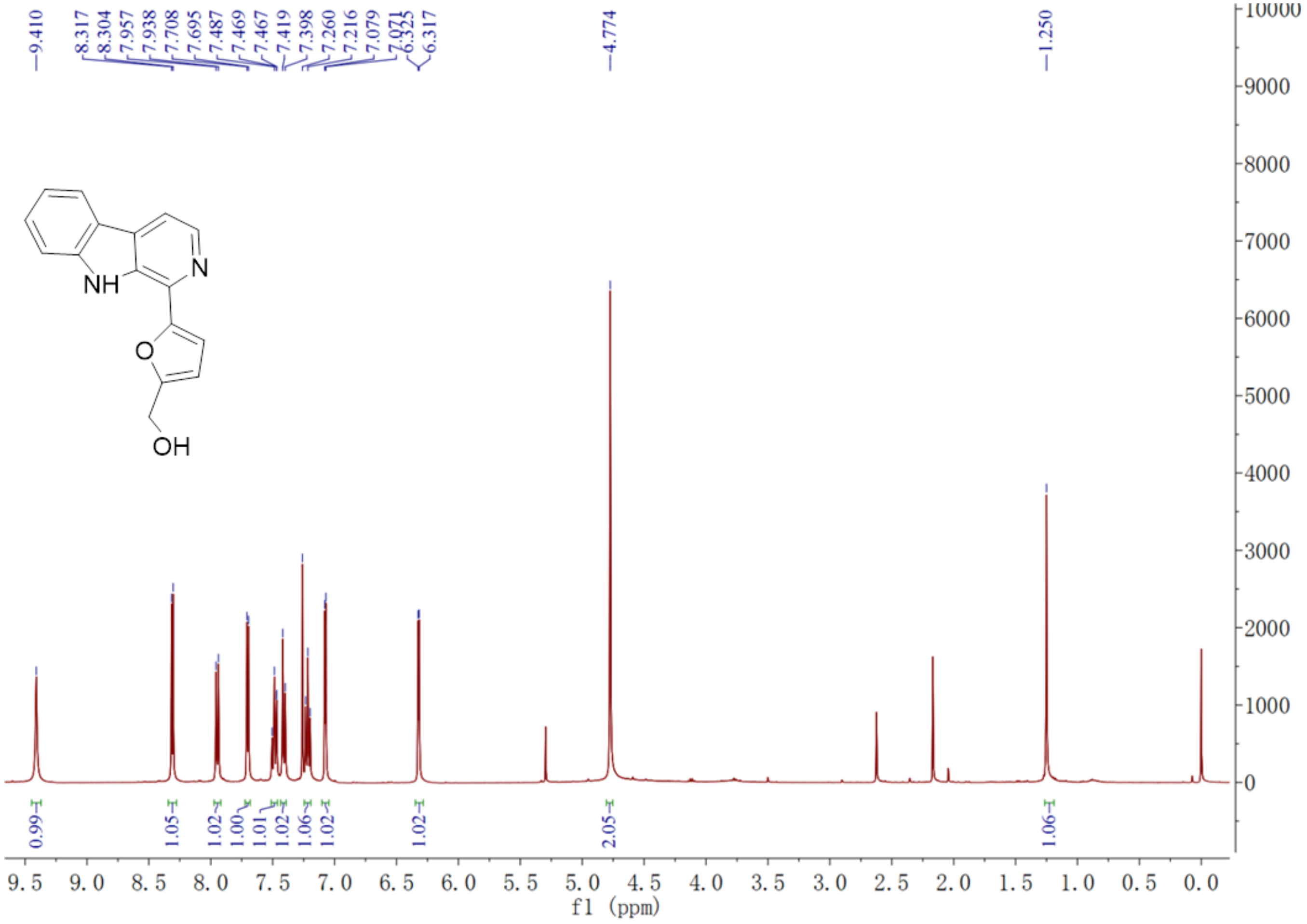

Figure S90. ^{13}C NMR spectrum of perlolyrine (**24**) in $CDCl_3$ (100MHz)

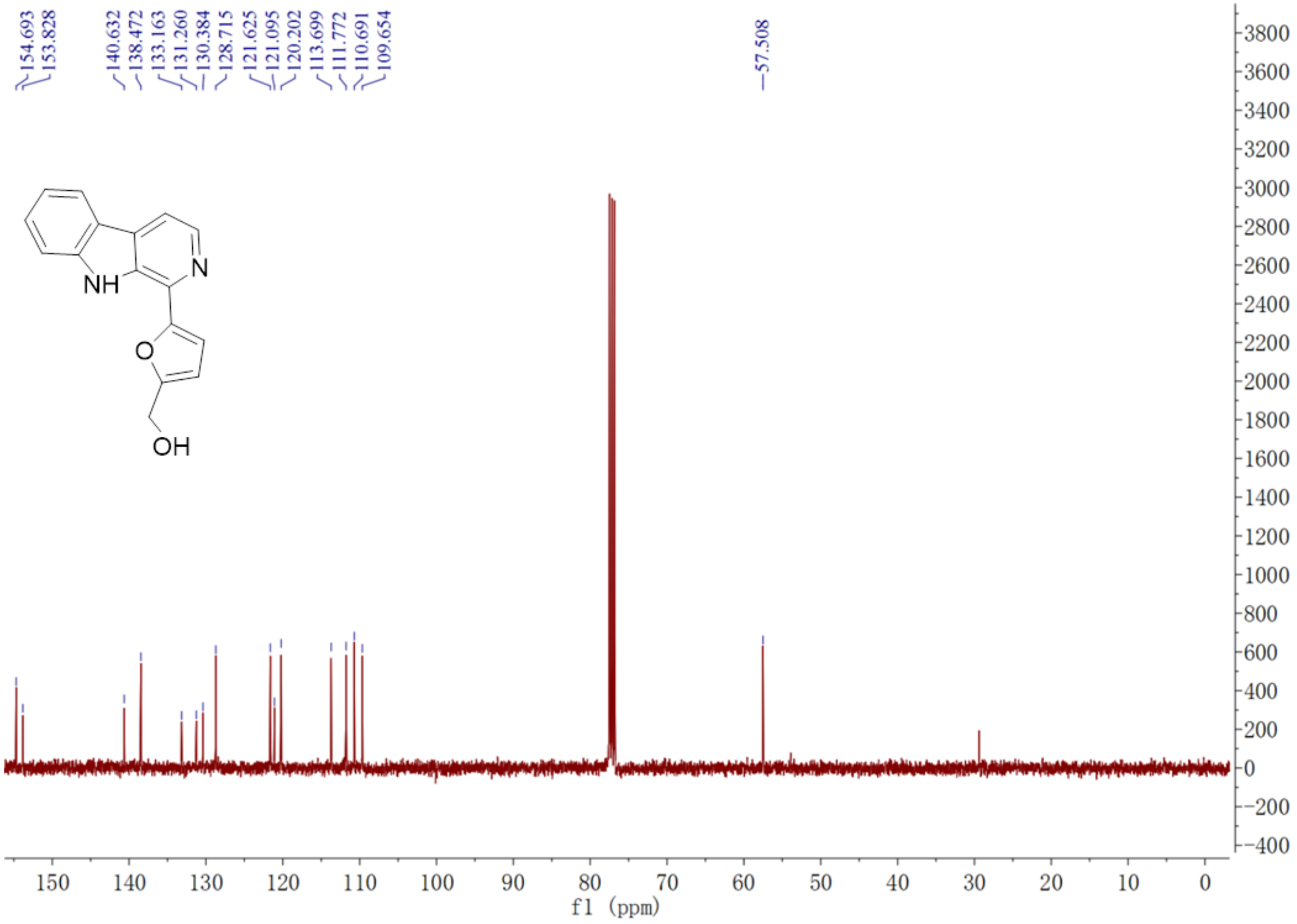

Figure S91. ^{1}H NMR spectrum of fiscalin C (**25**) in $CDCl_3$ (400MHz)

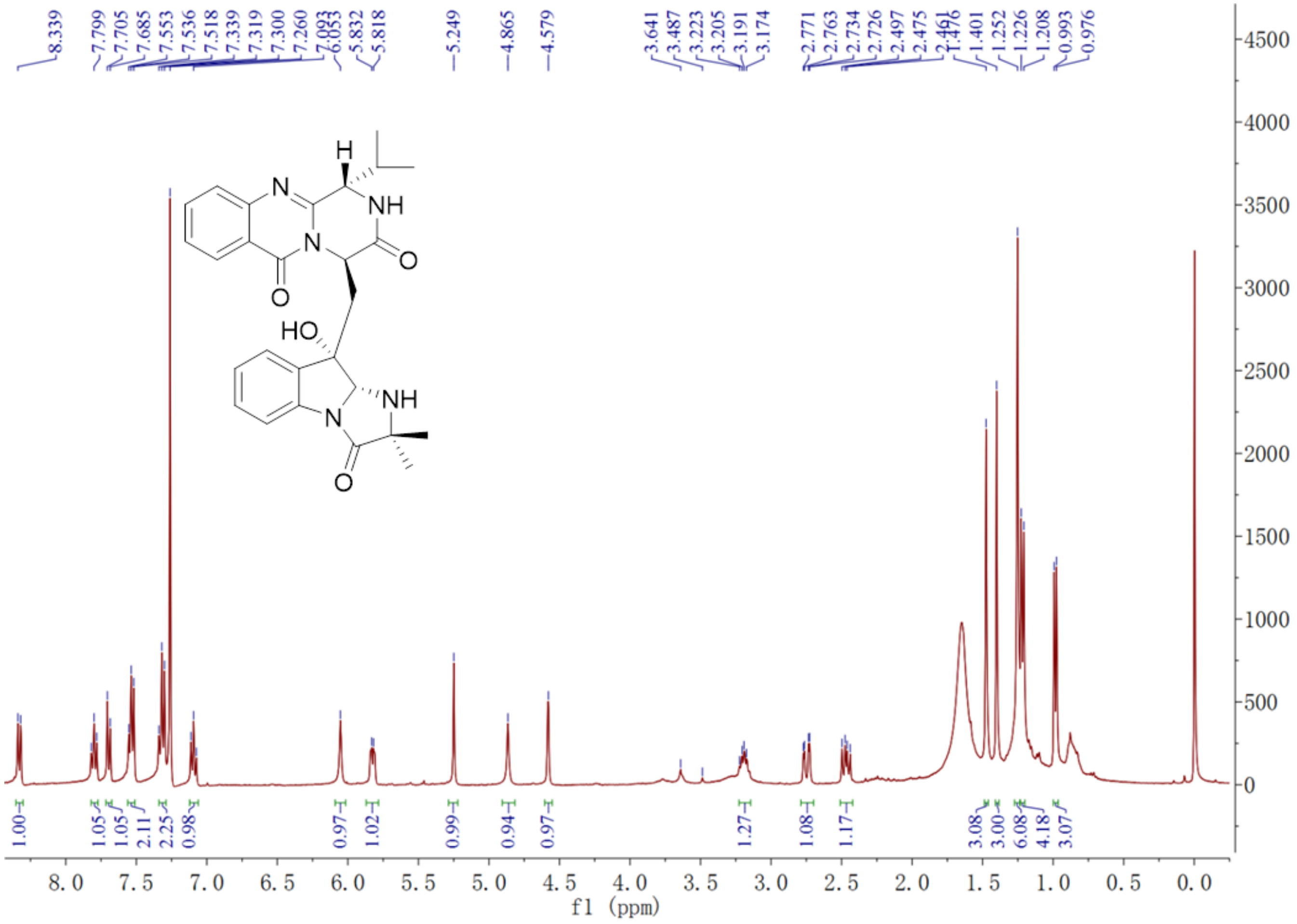

Figure S92. ^{13}C NMR spectrum of fiscalin C (**25**) in $CDCl_3$ (100MHz)

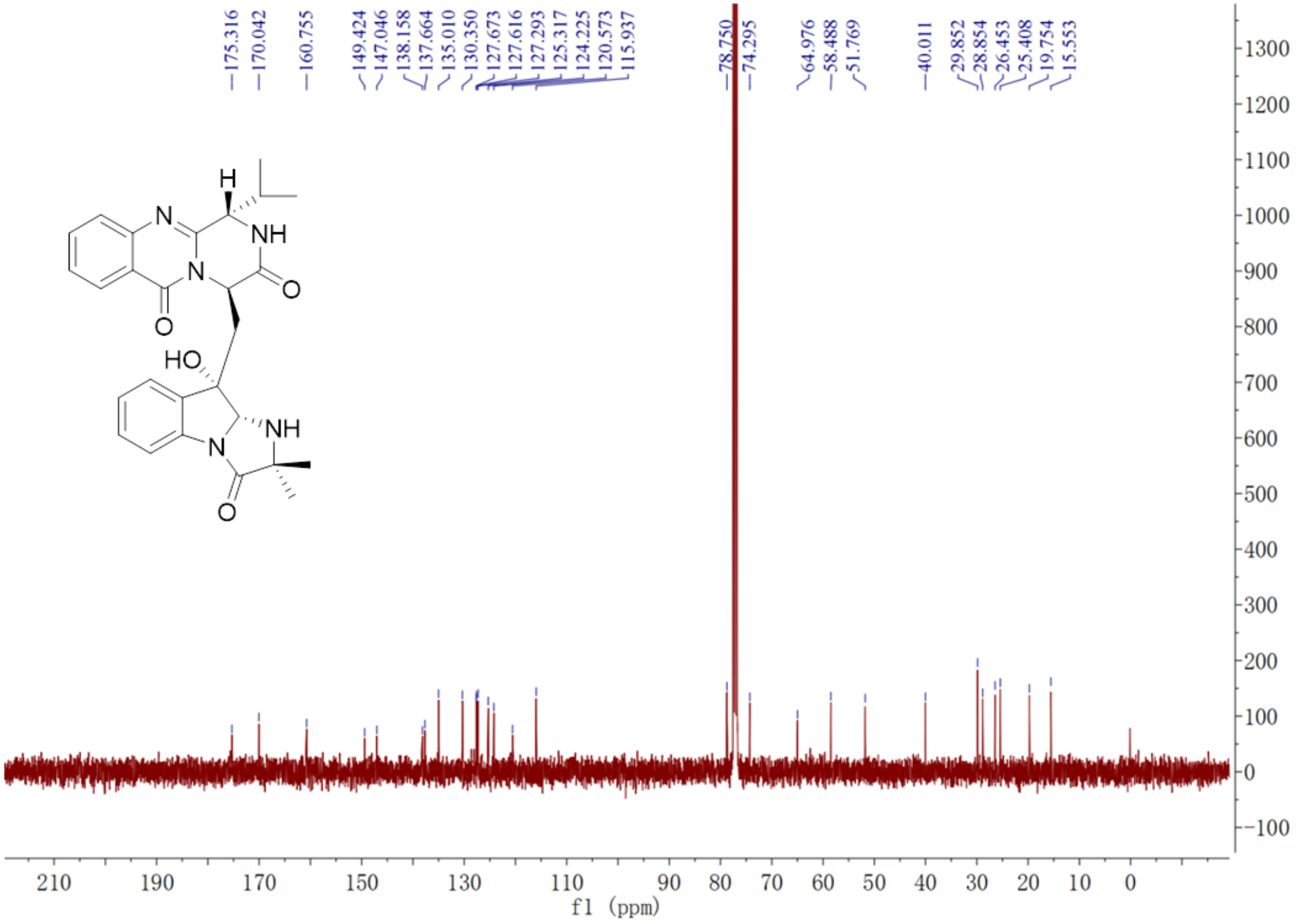

Figure S93. ^{1}H NMR spectrum of epi-fiscalin C (**26**) in $CDCl_3$ (400MHz)

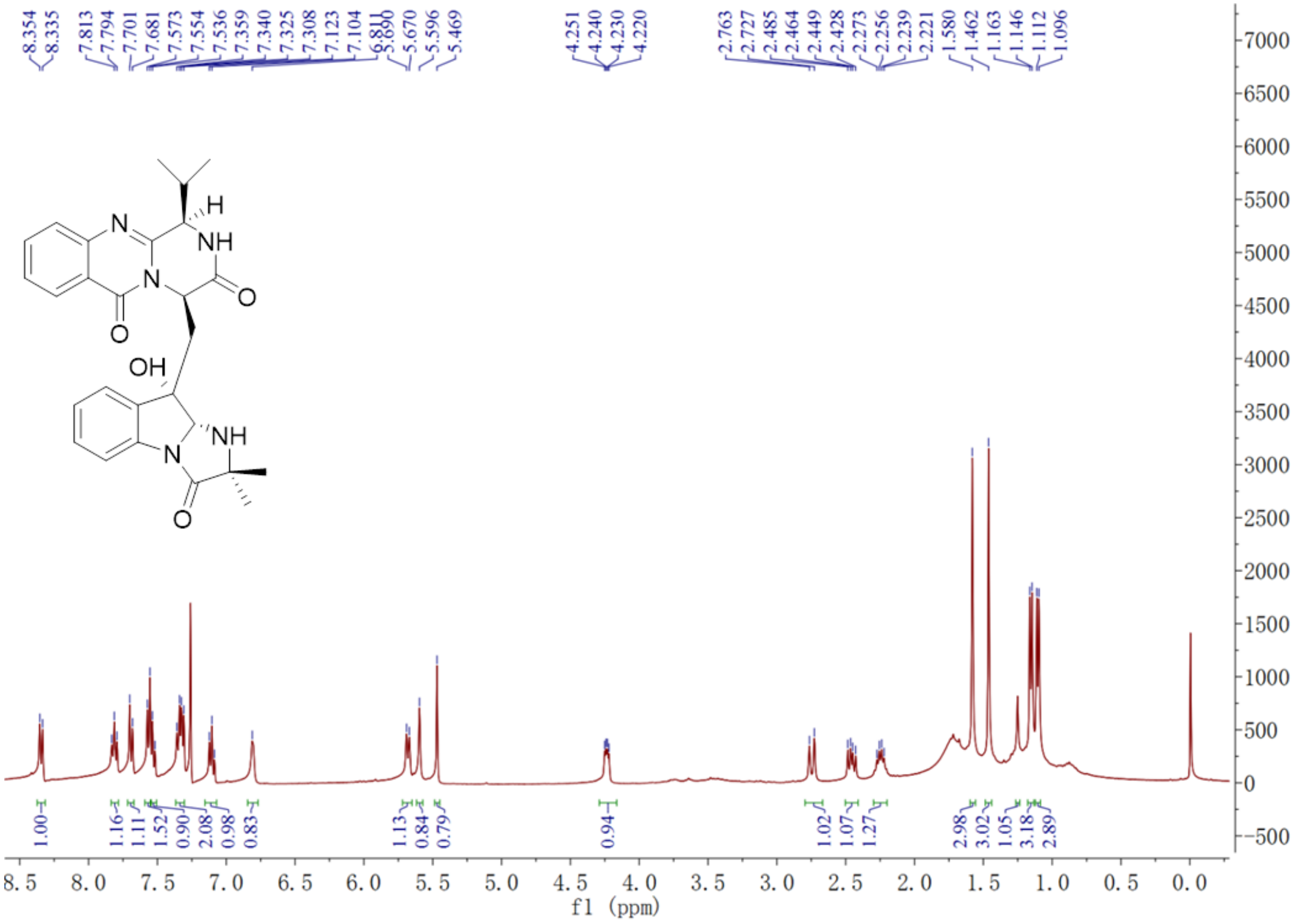

Figure S94. ^{13}C NMR spectrum of epi-fiscalin C (**26**) in $CDCl_3$ (100MHz)

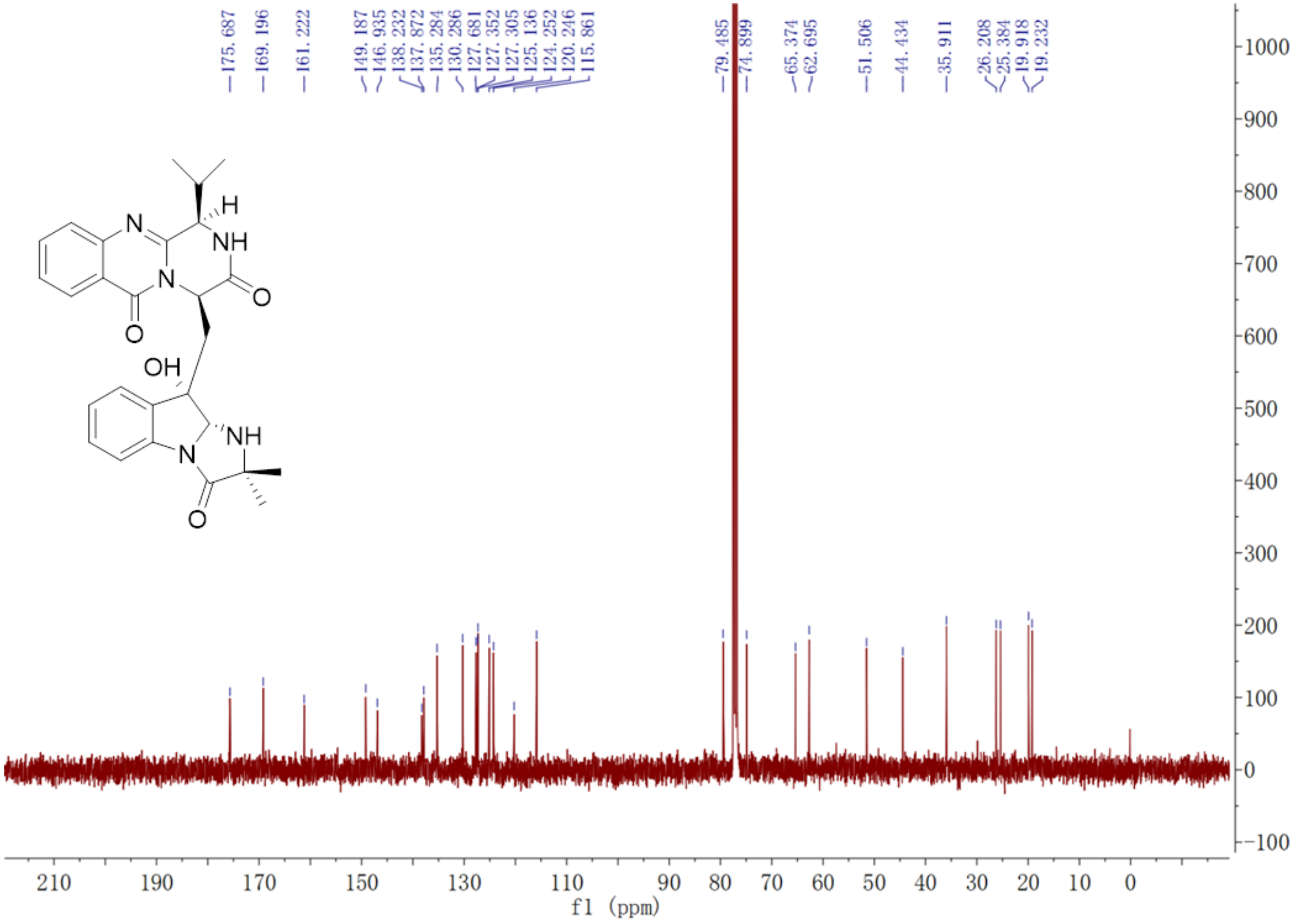

Figure S95. ^{1}H NMR spectrum of indolyl-3-acetic acid methyl ester (**27**) in $CDCl_3$ (400MHz)

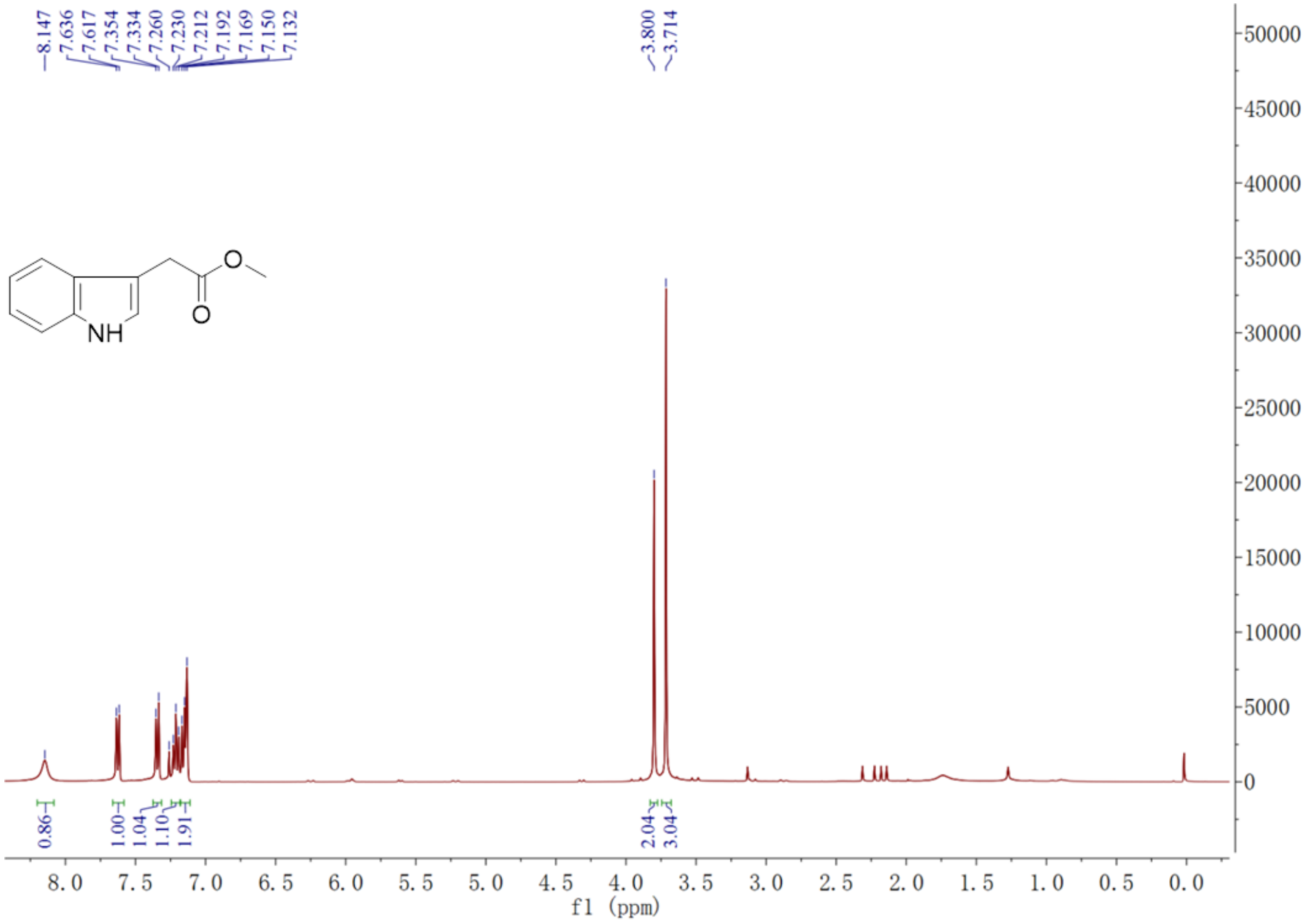

Figure S96. ^{13}C NMR spectrum of indolyl-3-acetic acid methyl ester (**27**) in $CDCl_3$ (100MHz)

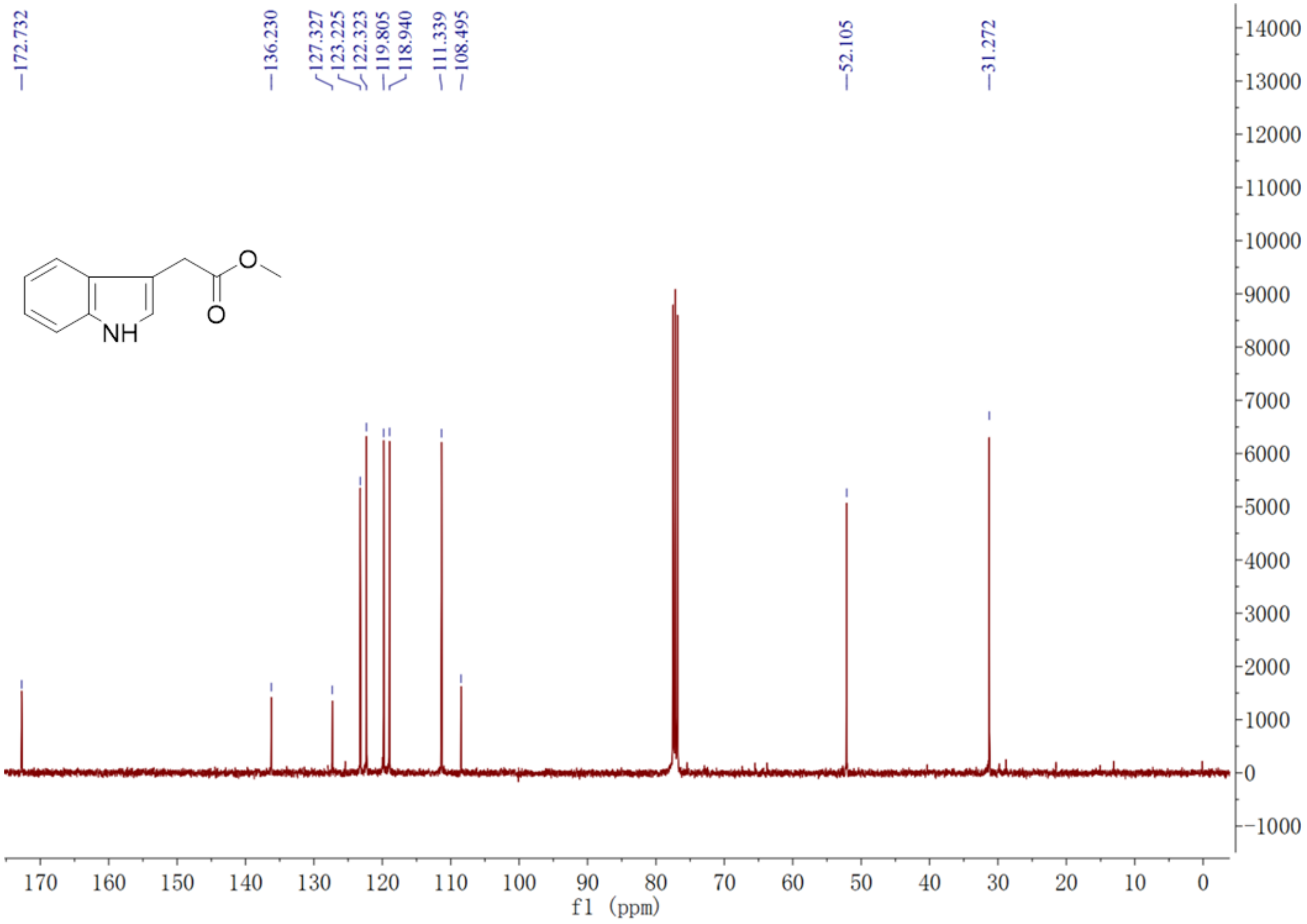

Figure S97. ^{1}H NMR spectrum of anthranilic acid (**28**) in Acetone-d_6 (400MHz)

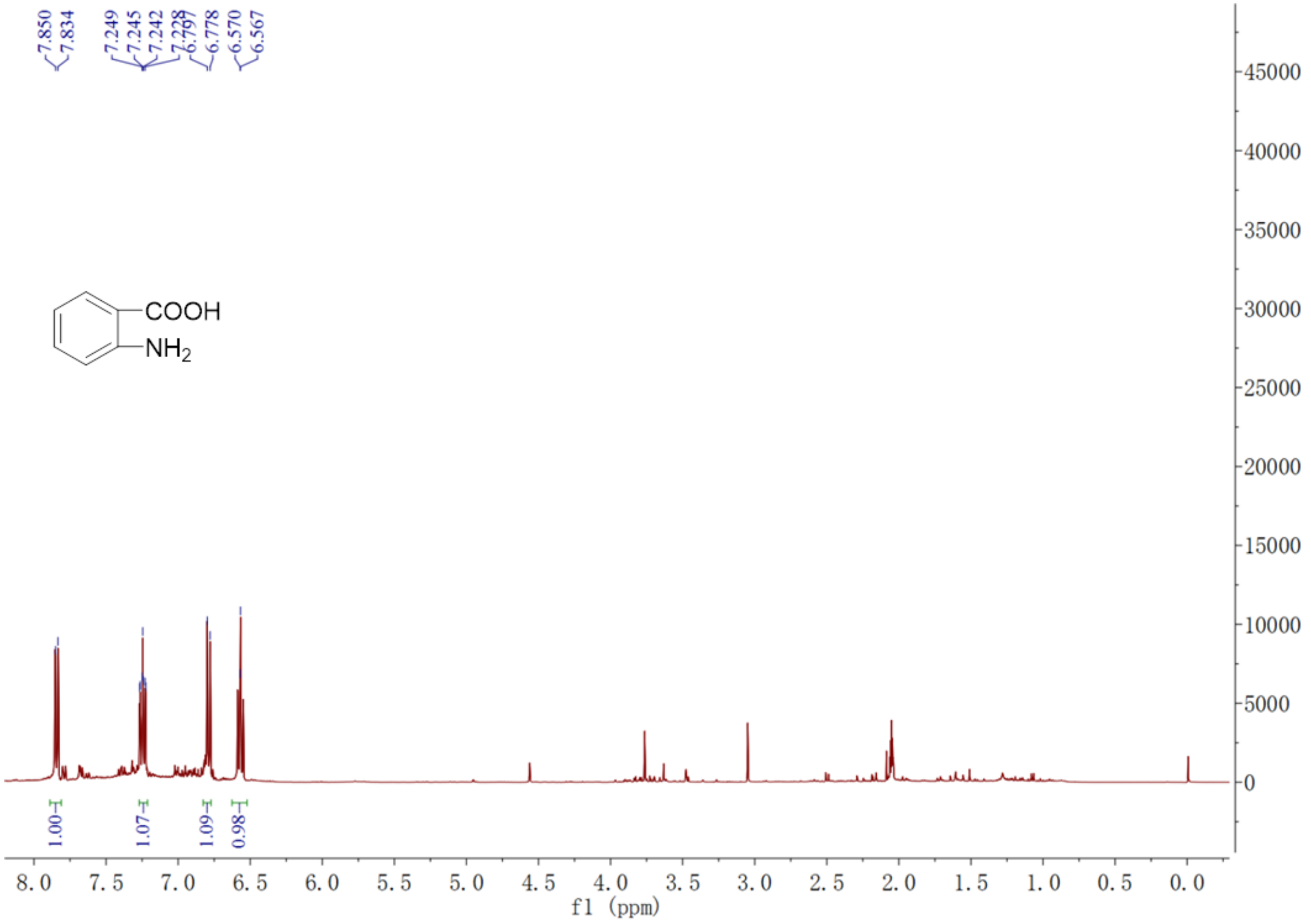

Figure S98. ^{13}C NMR spectrum of anthranilic acid (**28**) in Acetone-d_6 (100MHz)

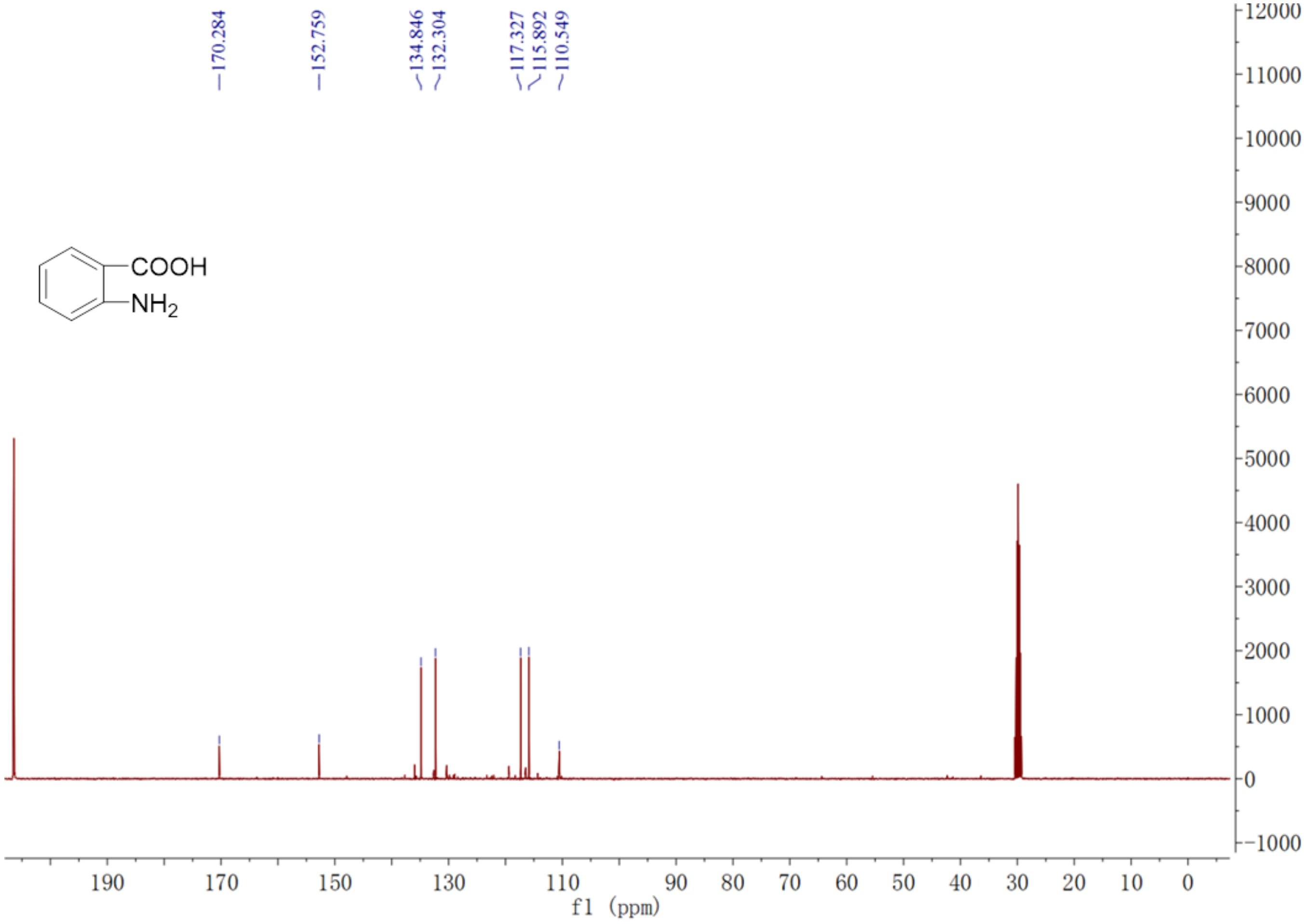

Supplement: Supplementary file 1 [file marinedrugs-15-00339-s001.pdf]
